# Latitudinal and bathymetrical species richness patterns in the NW Pacific and adjacent Arctic Ocean

**DOI:** 10.1038/s41598-019-45813-9

**Published:** 2019-06-26

**Authors:** Hanieh Saeedi, Mark J. Costello, Dan Warren, Angelika Brandt

**Affiliations:** 1 Department of Marine Zoology, Senckenberg Research Institute and Natural History Museum, Senckenberganlage 25, 60325 Frankfurt am Main, Germany; 2 FB 15 Biological Sciences, Institute for Ecology, Evolution and Diversity, Goethe University Frankfurt, Max-von-Laue-Str. 13, 60438 Frankfurt am Main, Germany; 3 OBIS data manager, Deep-Sea Node, Frankfurt am Main, Germany; 4 Institute of Marine Science, University of Auckland, Auckland, 1142 New Zealand; 5 Senckenberg Biodiversity and Climate Research Centre, Senckenberganlage 25, 60325 Frankfurt am Main, Germany

**Keywords:** Environmental sciences, Marine biology

## Abstract

Global scale analyses have recently revealed that the latitudinal gradient in marine species richness is bimodal, peaking at low-mid latitudes but with a dip at the equator; and that marine species richness decreases with depth in many taxa. However, these overall and independently studied patterns may conceal regional differences that help support or qualify the causes in these gradients. Here, we analysed both latitudinal and depth gradients of species richness in the NW Pacific and its adjacent Arctic Ocean. We analysed 324,916 distribution records of 17,414 species from 0 to 10,900 m depth, latitude 0 to 90°N, and longitude 100 to 180°N. Species richness per c. 50 000 km^2^ hexagonal cells was calculated as alpha (local average), gamma (regional total) and ES50 (estimated species for 50 records) per latitudinal band and depth interval. We found that average ES50 and gamma species richness decreased per 5° latitudinal bands and 100 m depth intervals. However, average ES50 per hexagon showed that the highest species richness peaked around depth 2,000 m where the highest total number of species recorded. Most (83%) species occurred in shallow depths (0 to 500 m). The area around Bohol Island in the Philippines had the highest alpha species richness (more than 8,000 species per 50,000 km^2^). Both alpha and gamma diversity trends increased from the equator to latitude 10°N, then further decreased, but reached another peak at higher latitudes. The latitudes 60–70°N had the lowest gamma and alpha diversity where there is almost no ocean in our study area. Model selection on Generalized Additive Models (GAMs) showed that the combined effects of all environmental predictors produced the best model driving species richness in both shallow and deep sea. The results thus support recent hypotheses that biodiversity, while highest in the tropics and coastal depths, is decreasing at the equator and decreases with depth below ~2000 m. While we do find the declines of species richness with latitude and depth that reflect temperature gradients, local scale richness proved poorly correlated with many environmental variables. This demonstrates that while regional scale patterns in species richness may be related to temperature, that local scale richness depends on a greater variety of variables.

## Introduction

The latitudinal and bathymetrical gradients of marine species richness have been widely studied at both regional^1–5^ and global scales^6–10^. Recent studies showed that the global latitudinal richness gradient in most marine species follows a bimodal pattern correlated with sea surface temperature^6,8,11–14^. That is, richness was highest in the tropics but it dips at the equator. In general, present marine species richness gradients decline from mid to high latitudes and from shallow to deep sea in many taxa^1,7,11,15–17^. However, diversity in some deep-sea taxa such as gastropods and nematodes increases from the continental shelf to the bathyal and abyssal zones due to increased environmental stability^18–20^. The deep sea is almost two-thirds of the Earth, and over 84% of the ocean area is deeper than 2,000 m^7,21,22^. In contrast to shallow waters, in the deep sea chemical energy and carbon flux mostly control the species diversity, and temperature does not predict variation in rarified diversity in many taxa (e.g., Bivalvia and Gastropoda)^22,23^. Climate change can alter deep-sea latitudinal diversity gradients, even at tropical latitudes^24^. The latitudinal gradients in species richness in the deep sea were generally present for the last 36 million years, but were weakened or absent during glacial periods^24^. Thus, considering both latitude and deep-sea gradients of marine species richness together in the same geographic region seems overdue.

Long-term global and more recent regional processes are likely to drive the marine species richness. For example, major historic events (e.g. glaciation and plate tectonics) and evolutionary processes such as origination, dispersal (range expansion), and extinction, are considered important driving factors shaping the current latitudinal patterns of richness of marine species^16,25–29^. Global climatic constraints resulting from plate tectonics modulate ocean circulation, resulting in changes in surface water characteristics as well as altering connectivity between populations^30^. In addition to continental drift and sea level change, recent latitudinal marine species richness analyses have considered light (as photosynthetically active radiation (PAR)), sea surface temperature, and habitat (e.g., continental shelf) in shaping the latitudinal gradients for shallow water marine species (e.g., bivalves and gastropods less than 200 m; recent and fossil marine zooplankton)^8,11,16,17,31–34^. Because light and temperature directly influence biomass and/or abundance, diversity may then increase as a result of secondary population dynamics and/or evolutionary processes^29,35,36^. Temperature, as a proxy for thermal energy, also enhances the utilization rate of chemical energy by organisms. Temperature may also influence diversity by allowing a greater range of energetic lifestyles at warmer temperatures (the metabolic niche hypothesis)^35,36^. Tropical warmer climates have thus increased metabolic scope and biodiversity by fostering greater population size and extinction resistance^29^. This allows more species to inhabit specialized niches as a result of greater available energy, and generates faster speciation and/or lower extinction rates^29,35^.

The tropical and subtropical areas of the west Pacific host the highest number of marine species worldwide^37–41^. They also have high topographic complexity, including large semi-enclosed seas, many islands, and deep-sea trenches. The regions high species richness may thus be due to high rates of speciation due to warm temperatures and repeated separations and reconnections of populations due to changing sea levels and continental drift. However, despite the high species richness and uniqueness of the NW Pacific, its latitudinal and depth gradients of marine species richness and their potential causes have not been studied. Here we show how species richness changes with latitude and depth, and consider potential explanatory factors including temperature, oxygen (dissolved and saturated), primary productivity, chlorophyll, current velocity, salinity, nitrate, ocean area, and sampling effort. We also considered the adjacent Arctic Ocean of the NW Pacific to discover how these patterns change towards the highest latitudes. If these latitudinal and depth gradients are largely temperature correlated it would suggest that other variables, including topographic complexity, had negligible influence on the evolution of the fauna.

## Methods

**Figure 1:**
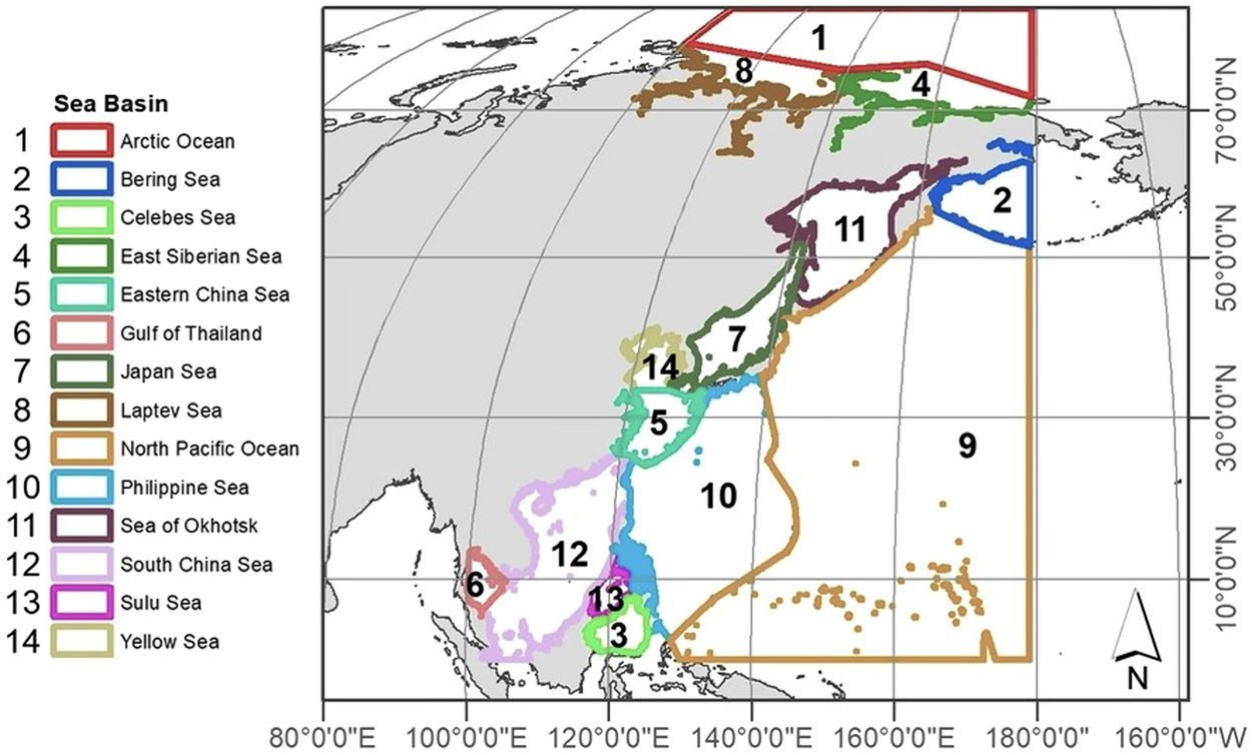
Study area located in the NW Pacific and adjacent Arctic Ocean (latitude: 0 to 90°N in latitude and longitude: 100 to 180°N) showing 14 Sea Basins with different colours. ArcMap 10.5.1 was used to create this figure (https://support.esri.com/en/products/desktop/arcgis-desktop/arcmap/10-5).

Our study area included the NW Pacific and its adjacent Arctic Ocean from latitude 0 to 90°N, and longitude 100 to 180°N including 14 sea basins (Fig. 1). All geographic distribution records were extracted from Ocean Biogeography Information System (OBIS) (www.iobis.org) and Global Biodiversity Information Facility (GBIF) (https://www.gbif.org) (for citations of the datasets used, please see SI, Table S1). The extracted data were merged and duplicates excluded. All species names were matched against the World Register of Marine Species^42^ and synonyms reconciled. Distribution records were manually checked for suitability, and dubious records were either corrected (e.g., reversing latitude and longitude fields, duplicate records) or removed (e.g., fossil records). The final dataset consisted of 324,916 distribution records of 17,414 marine species (1,792 families) from 0 to 10,900 m depth (SI, Fig. S1). Moreover, all the species were categorized to shallow-water and deep-sea benthic and pelagic groups (SI, Fig. S2). The statistical software R 3.4.4 and ArcMap 10.5.1 were used to analyse the data and plot the graphs.

We used three measures of species richness for each c. 50 000 km^2^ hexagonal cell, latitudinal band, and depth interval: (1) the total number of species per hexagon (alpha diversity); (2) the total number of species per latitudinal band (gamma diversity); and (3) the estimated richness per sampled hexagon using rarefaction (ES50). The rarefaction method was used to reduce the effect of sampling effort on species richness patterns by counting the number of species in a constant number of random samples. We considered each sample as a unique combination of date and location where one or more species were recorded. We used ES50 in the ‘vegan’ R package to estimate the species richness in 50 samples per hexagon^43^. Analyses considered samples at spatial scales of hexagons and 5° latitudinal bands. The former capture the heterogeneity in the underlying data best, but contain data collected by a variety of methods and sample sizes which are not necessarily comparable (e.g., plankton, whales, turtles, sharks, tracked animals, sediment cores, trawls, SI, Table S1). Hexagonal species richness analysis introduces considerable variability and bias between hexagons. The aggregation of samples into latitudinal bands aims to smooth out these effects because the number of species in a 5° band would tend to reach an asymptote.

We extracted environmental factors including average temperature (°C), dissolved oxygen (mol.m^−3^), primary productivity (g.m^−3^.d^−1^), chlorophyll (mg.m^−3^), current velocity (m^−1^), saturated oxygen (%), salinity (PSS), and nitrate (mol.m^−3^) for shallow water records (0–500 m); and average temperature, dissolved oxygen, chlorophyll, current velocity, salinity, and nitrate for deep-sea records (>500 m) from Bio-ORACLE (http://www.bio-oracle.org/)^44,45^ and Global Marine Environment Datasets (GMED) (only saturated oxygen) (http://gmed.auckland.ac.nz/)^46^. All the extracted environmental layers were at a 5 arcmin (c. 9.2 km) spatial resolution

We used generalized additive models (GAMs) to examine the impact of environmental predictors on number of species and ES50 on a per-hexagon basis. The mid-point of each c. 50 000 km^2^ hexagonal cell was calculated and collated with the spatial resolution of the environmental variables. Due to the high incidence of zeros in our species count data, models were built using the negative binomial error distribution. We fitted models via restricted maximum likelihood, using the automatic predictor selection implemented in the mgcv package^47^ to control the complexity of smooth terms. For each analysis we fitted an intercept-only model, which represented the null hypothesis that response variables were not explained by environment, spatial sampling bias, or spatial autocorrelation. For models built using number of species as the response variable, we used the total number of records for each locality as an estimate of sampling effort. Models using ES50 as a response variable excluded number of records as an explanatory variable, as ES50 calculations are themselves intended to control for sampling effort. To model the effects of spatial autocorrelation on predictor and response variables, we used a two-dimensional spherical spline on latitude and longitude of sampling sites^47^.

For models using number of species as a response variable, we fitted one model using only spatial sampling bias, one using only spatial autocorrelation, and one using both sampling bias and spatial autocorrelation. We also fitted a model for each environmental predictor separately, and one that represented the combined impacts of all environmental predictors. Models built using environmental predictors also included sampling effort and the effects of spatial autocorrelation. Candidate sets of models using ES50 as a response variable were the same as for number of species, except for the exclusion of sampling effort. The GAMs relating species richness and ES50 to environmental predictors on a per-hexagon basis, as well as tables presenting detailed model selection results, are in SI, S1 (Species Counts and Environment Based on Hexagonal Cells).

Generalizing per-hexagon data to 5° latitudinal bands substantially reduced sample size, which limited our ability to fit complex functional responses. Therefore, for this data, we fitted generalized linear models (GLMs) using a Poisson error distribution, and used number of records per band to control for differences in sampling effort. Per-hexagon and 5° models were evaluated using the small sample size corrected Akaike Information Criterion (AIC^48^), a statistical method used to choose models with optimal fit to the data while controlling for over-parameterization^49,50^. The models with lower AIC scores are those that demonstrated a better compromise between model fit and model complexity. A difference in AIC value (deltaAIC) of less than two is considered to be inconclusive when comparing models. GLMs relating species richness and ES50 values to environmental predictors are given in SI, S2 (Species Counts and Environment Based on 5° Latitudinal Bands) and Fig. S4.

## Results

### Species richness

**Figure 2:**
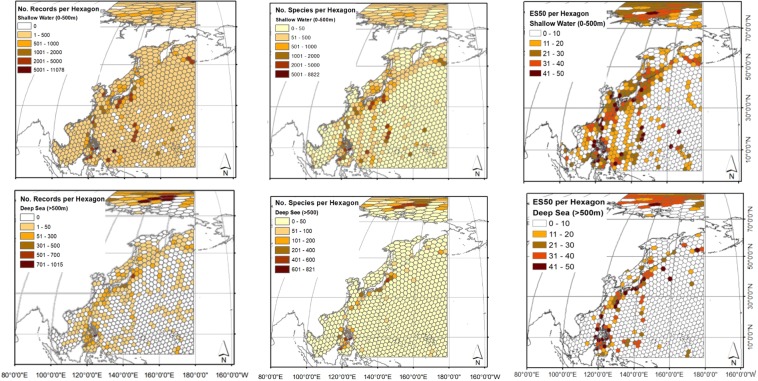
Total number of records, species, and ES50 calculated per c. 50 000 km^2^ hexagonal cells in depths above and below 500 m. ArcMap 10.5.1 was used to create this figure (https://support.esri.com/en/products/desktop/arcgis-desktop/arcmap/10-5).

Most records in the study area were in shallow depths, and only 8% of the hexagons had no reported samples (Figs 2 and S2). Of these, almost all (7% of hexagons) were in the tropical and subtropical NW Pacific (between 0 to 30°N). In contrast, for the deep sea, 62% of the hexagons had no data. The highest sampling effort in both shallow (11,000 records) and deep sea (1,000 records below 500 m) in the study area was in the Philippines around Bohol Island (10.05°N, 124°E; ocean area: 3,000 km^2^) (Fig. 2).

The highest alpha species richness (shallow water: 8,800 species; deep sea: 800 species) was in the Philippines around Bohol Island (Fig. 2). The ES50 species richness index ranges from 0 to 50 and accounts for the effect of the number of samples. The highest ES50 (49) was also observed in Bohol Island in shallow water. However, the highest ES50 (48) in the deep sea was recorded around Luzon Island (16.14°N, 122.40°E; ocean area: ~1,250 km^2^) in the Philippines. In general, the Bering, Japan, Philippine, Sulu and Celebes Seas, including some areas of subtropical NW Pacific Ocean, had the highest alpha species richness in the shallow and deep NW Pacific. The Arctic Ocean also had high alpha species richness, with some values of ES50 > 40.

**Figure 3:**
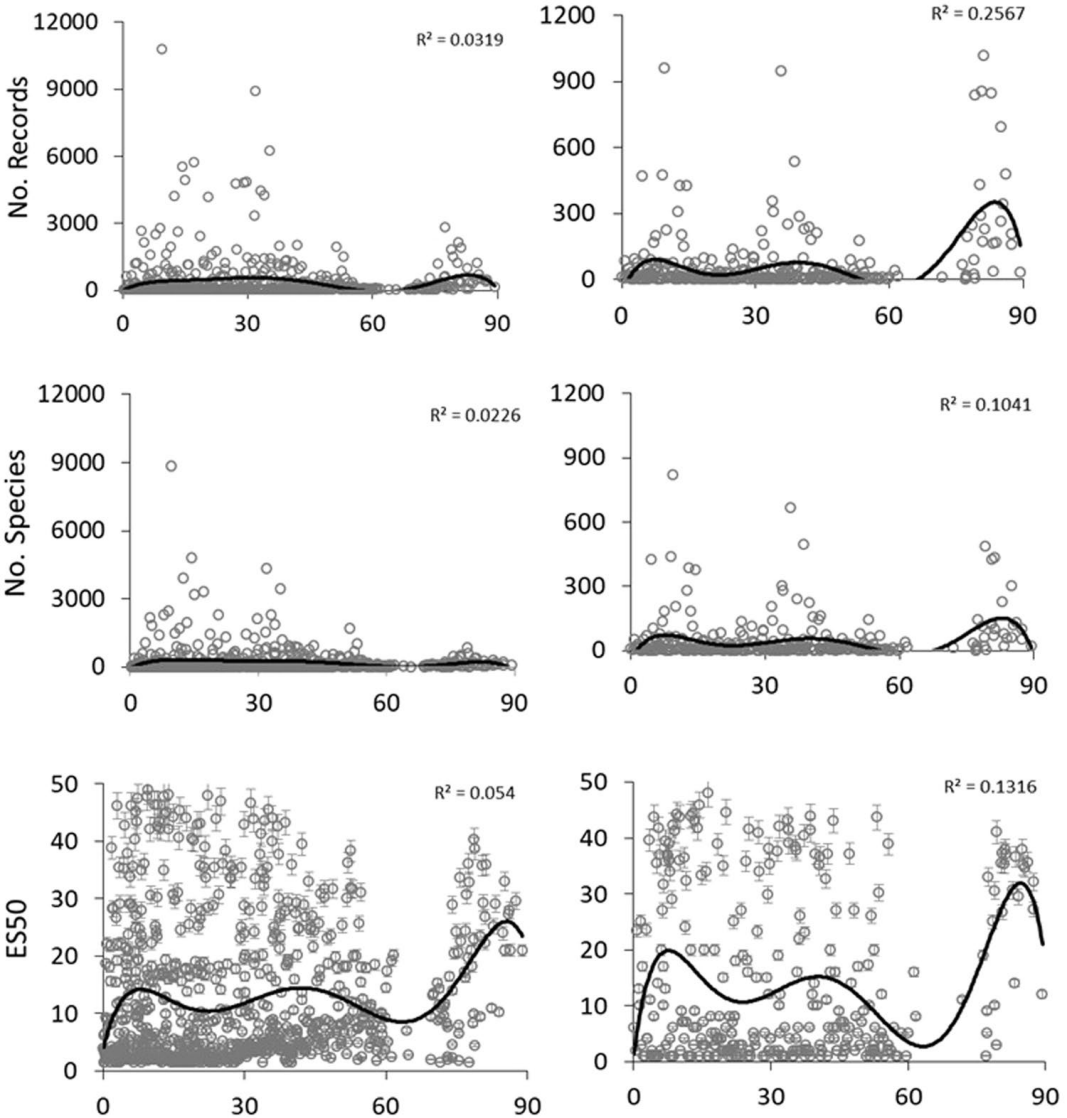
The number of sampling records, alpha species richness (number of species per hexagon), and ES50 ± SE calculated per hexagon against latitude for both shallow water (0–500 m) and deep sea (>500 m) species. Solid lines shows order 6 polynomial trend lines regression trends over the latitude.

**Figure 4:**
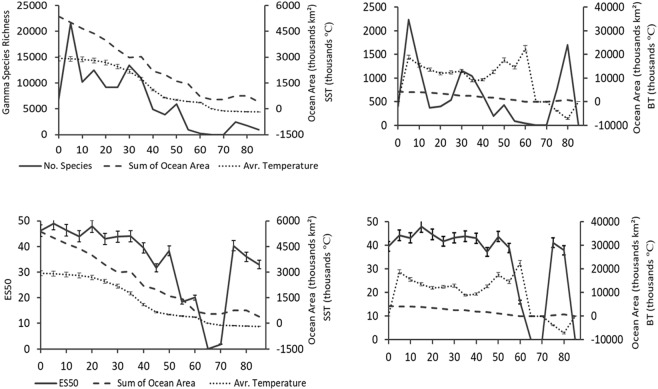
Gamma species richness (number of species per 5° latitudinal band), average ES50 ± SE, average sea surface (SST) and bottom (BT) temperatures (10^4^ ± SE), and ocean area against 5° latitudinal bands.

Latitudinal gradients in number of records, alpha species richness, gamma richness, and ES50, increased from the equator to latitudes 5 or 10°N, then decreased, and further reached another peak at the highest latitudes (75 to 90°N) (Figs 3 and 4). Latitudes 5 to 10°N hosted the highest species richness and ES50 which was mostly belonged to the Philippine Sea (average sea surface temperature = 28 °C and bottom temperature = 15 to 18 °C) (Figs 3 and 4). That further peaks in alpha and gamma richness were not seen in average ES50 which indicates that those peaks were due to the number of samples (Fig. 4). The latitude 60 to 70°N had the lowest species richness and ES50, as this is the smallest ocean area in this region (about 500,000 km^2^). Apart from this decrease related to ocean area, ES50 was very similar across latitudes (Fig. 4).

**Figure 5:**
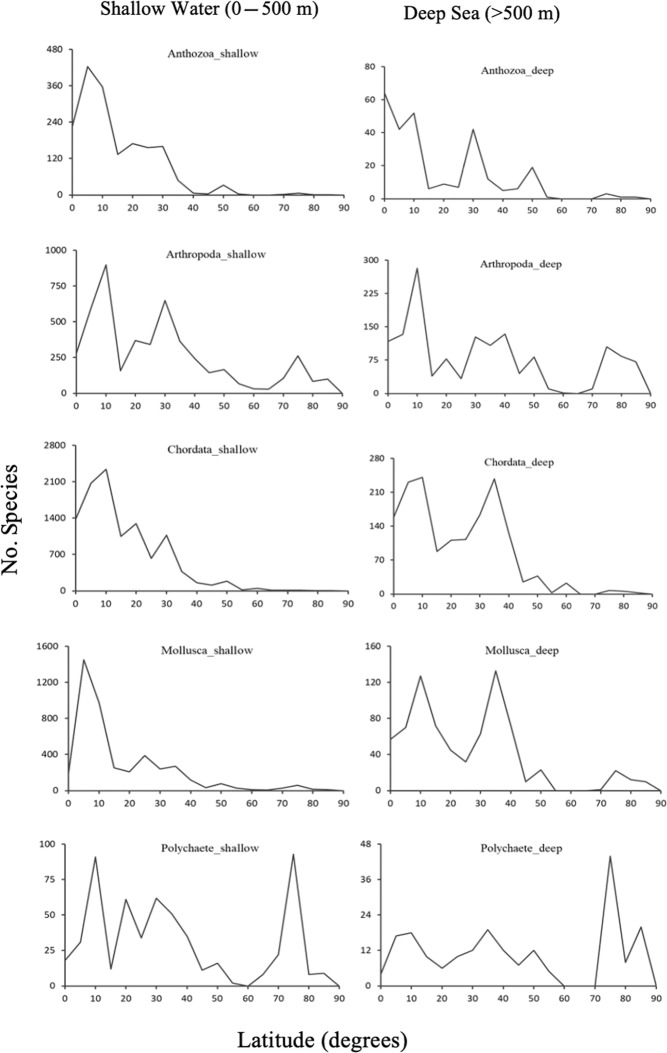
Total number of species (Gamma species richness) against latitude for five selected taxa.

Both shallow-water and deep-sea Anthozoa and Chordata had their highest gamma species richness in the subtropical areas from 5 to 10°N (Fig. 5). For Mollusca, highest shallow-water species richness was around 5°N and from 30 to 40°N in deep sea. Surprisingly, both shallow-water and deep-sea Polychaeta had their highest gamma species richness in the Arctic from 70 to 80°N, but not the NW Pacific Ocean. Most of the distribution records for higher taxa were observed between latitude 20 to 40°N (SI, Fig. S3). Mollusca, Porifera, and Bryozoa were mostly distributed around 10° latitude. Cnidarians, Pisces, and crustaceans had their higher distributions from latitude 20 to 30°N.

### Environmental variables

For shallow waters, the model using salinity as the sole environmental predictor for number of species had the lowest AIC value, followed closely by the model with all environmental predictors (deltaAIC of 0.32) (SI, S1). Model selection tables for the response variables are included in SI, S1. Several other models were within deltaAIC < 2 from the best model, including models using temperature, productivity, chlorophyll, current, and oxygen saturation. However, with a deltaAIC of only 1.8 between the top model and the one containing only spatial autocorrelation, none of these results is particularly strong. Considering ES50 for shallow communities yields stronger results; the model with all environmental predictors had the lowest AIC value, with a deltaAIC > 25 between it and the model containing only spatial autocorrelation. A model for ES50 in shallow waters that contained only the effects of productivity had a deltaAIC of 0.06 when compared to the one with all environmental predictors, indicating that much of the predictive power of the environment for determining ES50 in shallow waters likely comes from the effects of productivity. No other model for ES50 in shallow waters had a deltaAIC < 2 when compared to the top model.

In deep waters, both species richness and ES50 per hexagon were correlated with the environmental predictors. For both response variables, the model with the lowest AIC was the one containing all environmental predictors, comparing those models to models with only spatial autocorrelation results in a deltaAIC of 10.0 for number of species and 6.7 for ES50. Much of the explanatory power in these models seems to come from a combination of salinity, temperature, and dissolved oxygen; additional models with deltaAIC < 2 included salinity and temperature as predictors for number of species and oxygen as a predictor of ES50. The difference in deviance explained by all models containing environmental predictors compared to models containing only spatial autocorrelation is not large. However, this does not indicate that these predictors are not important; many of the predictors show substantial spatial autocorrelation, and as such the effects of the two-dimensional smoother fit to latitude and longitude may tend to assume some of the predictive power of the environmental predictors.

Much of the deviance in ES50 and species richness across 5° latitudinal bands was explained by sampling effort (number of records), but model selection nevertheless shows significant effects of the environmental predictors in some cases (SI, S2 and Fig. S4). Model selection tables for the response variables are included in SI, S2. Salinity was the best predictor of number of species in shallow water, but the intercept-only model was within 0.42 deltaAIC of the top model for ES50 in shallow water, indicating little explanatory power of the environment. In deep-water communities the story is similarly ambiguous when looking over 5° latitudinal bands; current was the top predictor of species counts in deep waters, while nitrate was the top predictor of ES50. We do caution that the severely reduced sample size and lack of explicit spatial autocorrelation terms in these models likely makes these results less reliable than those for individual hexagons.

### Depth

**Figure 6:**
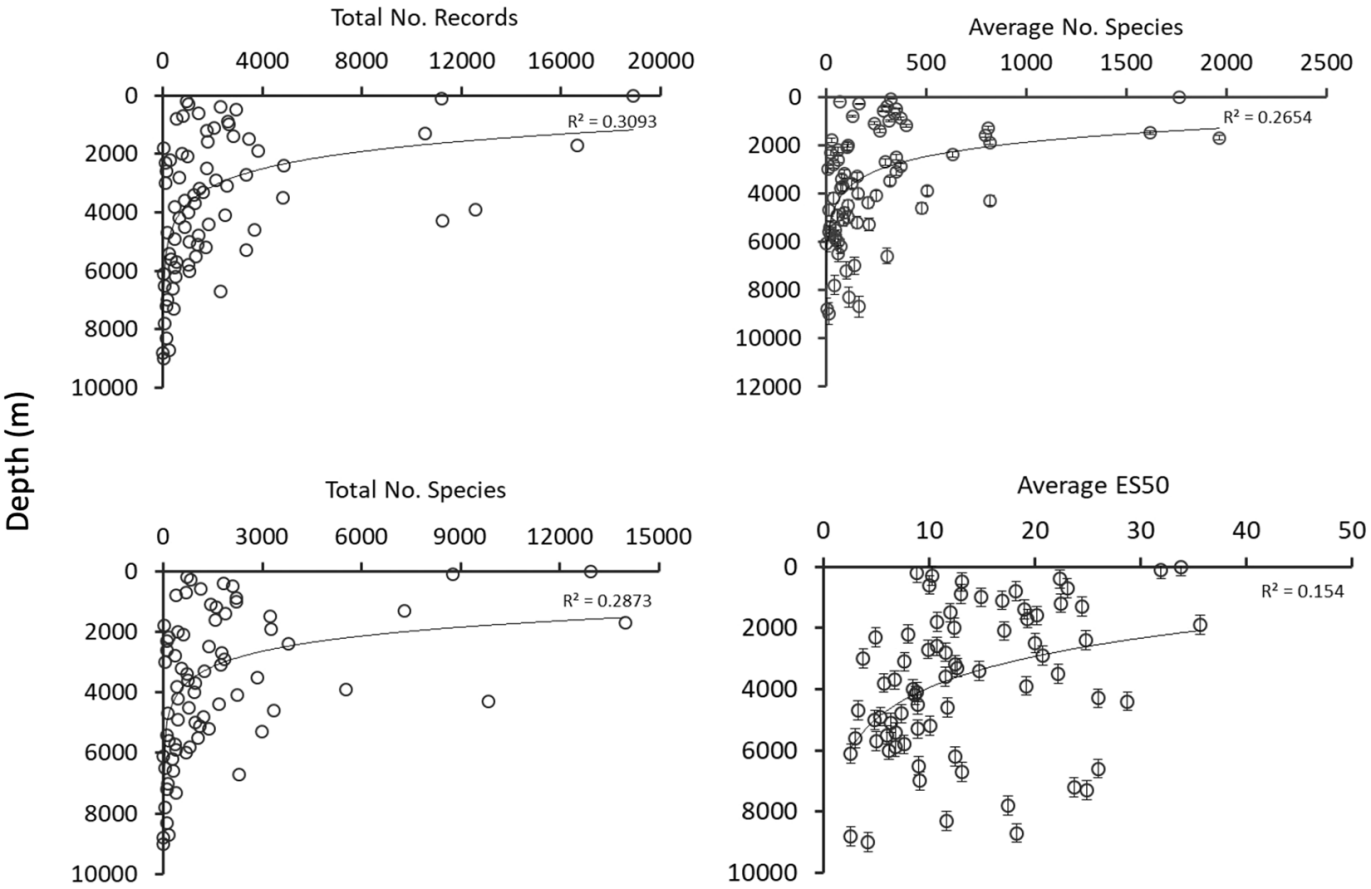
The relationship of sampling effort (total number of records), alpha species richness (average number of species), gamma species richness (total number of species), and average ES50 per hexagon per 100 depth intervals. The solid lines show the logarithmic trends.

From a total number of 324,916 species distribution records, 83% were from shallower than 500 m: 0–50 m = 48,809; 50–100 m = 10,869; 100–200 m = 10,670; 200–500 m = 23,538; and for > 500 m there were 22,104 records (7%). All the species were divided into four groups including shallow water and deep-sea pelagic and shallow water and deep-sea benthic species. A total number of 352,969 distribution records (the number of records here are higher than stated before because some species were grouped in both pelagic and benthic, or/and shallow water and deep sea categories) of four groups were mapped (SI, Fig. S1). About half of all the records (174,182 records) belonged to shallow-water pelagic species and only ~3% (12,176 records) were classified as deep-sea pelagic species. About 21% (73,282 records) and 26% of (93,329 records) the records belonged to shallow-water and deep-sea benthic species, respectively. The total number of records per hexagon per 100 m depth intervals was highest from 0 to 500 m among all depths, and then decreased sharply with depth (Fig. 6).

In the deep sea, sampling effort generally decreased with depth in 100 m depth intervals with the exception of two peaks around 2,000 and 4,000 m. Average ES50, alpha, and gamma species richness per hexagon decreased with depth (100 m depth intervals) and had the highest values from 0 to 2,000 m (Fig. 6).

## Discussion

There were eight times less data available for the deep sea than coastal depths in our study area. Nevertheless, the data still showed a decline in species richness with depth when adjusted for sampling effort (ES50). Similarly, near coast areas had greater richness as ES50 than offshore areas (Fig. 2).

The most species rich area in the world ocean is the Coral Triangle, which includes the Philippine Sea (latitudes from ~5 to 35°N)^7,30,51,52^. This is the most species-rich region for bivalves^8,37,38,40,53^, bony fish^54,55^, sharks^56^, crustaceans^57^, ascidians^58^, anemones and corals^52,59^, benthic marine algae^60^, endemic fish^61^ and other marine organisms^62^. Our data support these findings. We found that the Philippine Sea around Bohol Island (10.05°N, 124°E) had the highest alpha species richness of both the shallow and deep NW Pacific. This area is where the Philippine Sea has its highest number of islands and is a meeting point for the species rich Sulu, South China, and Celebes Seas. Even when corrected for sampling effort, this area and Luzon Island (16.14°N, 122.40°E) had the highest expected species richness in shallow water and deep sea, respectively. However, specific taxa might show different diversity patterns. For example, Ophiuroidea species richness in continental shelf to upper-slope areas peaks at tropical Indo-west Pacific and Caribbean (0–30°) latitudes, following the water temperature^22^. In contrast, deep-sea ophiuroid species show maximum richness at higher latitudes (30–50°), in regions close to continental margins where carbon export flux is high^22^.

Many coral reef groups reach their greatest diversity in the Coral Triangle. In addition to higher coral reef diversity in this area^30,63^, there are more reasons why the Coral Triangle is so species rich. Being tropical it has not suffered from glaciation driven extinctions. The tropics have higher rates of potential speciation due to warm temperatures increasing mutation rates and decreasing generation times (see Costello and Chaudhary 2017 for a recent review). Warmer sea surface temperatures, high productivity, and habitat availability and heterogeneity are likely important factors responsible for high tropical shallow species richness^6,8,39,64^. Our richest spots for species were in the Philippine Sea with a mean SST from 24 to 28 °C and ~20 °C in winter^6,8,64^. The NW Pacific is relatively rich in nutrients from land and rainfall, enabling high productivity, large population sizes, and intra-specific competition that can drive speciation. The many islands and deep-sea areas create a diversity of bathymetric and oceanographic conditions to which species can evolve to adapt, and past fluctuations in sea level will have isolated populations in coastal areas that have subsequently become reconnected. In addition to present and past environmental conditions, this region serves as a meeting point for the biota of Asia and Australia, and the Indian and Pacific Oceans, including species that may have evolved in Australia as part of Gondwanaland^30,65^. Although the area has never been glaciated, it was definitely affected by Quaternary sea level cycles which could create a diversity pump mechanism, as a result of the relationship between Pleistocene sea level changes and the complex geography of the area, or as a result of accumulation, reflecting the exceptional environmental features of the region^30^.

While looking at our data set in isolation, one may consider that the lower richness at the equator (0 to 5°N) was an artefact of sampling bias. However, a recent review has shown that marine species richness does decrease at the equator, where the highest sea temperatures occur, across almost all taxa^11^. Thus the dip in richness may be due to thermal stress above an annual average SST of 28 °C. For example, bivalve larvae (e.g., *Mytilopsis leucophaeata*) have higher embryo and larval mortality in temperatures above 28 °C^8,66,67^, and annual average sea surface temperature was above 28 °C (29 to 30 °C) at the equatorial western Pacific from 1870 to 2005^68^.

Considering the best model fit, both hexagon-based and 5° latitudinal band models showed that ES50 was the better estimate of species richness in both shallow and deep sea. We also concluded that the best model explaining species richness in the shallow and deep sea was when all the environmental factors were considered rather than single variables. In other words, no single variable explained the pattern in species richness for either hexagons or latitudinal bands. Alpha diversity (species richness per hexagon), and gamma diversity (species richness per 5° latitudinal band), showed different regression levels and outcomes in the model outputs. However, the high spatial heterogeneity (hexagons) resulted in poor correlations between species richness and environmental conditions. While clearer correlation trend lines were found when data were aggregated into 5° latitudinal bands, the reduced sample size limited the statistical outcome of the results.

Sampling effort and bias are important factors to consider in interpreting species richness patterns. Indeed, studies on global latitudinal species richness gradients showed that alpha, and to a lesser extent gamma, species richness patterns were affected by sampling effort^8,11^. Our comparison of alpha, gamma, and ES50 (to account for sampling bias) showed that all measures still peaked between 5 to 10°N in both shallow and deep sea (Fig. 3). However, ES50 also showed high values in the Arctic Ocean, not indicated by alpha and gamma species richness. Average ES50, alpha, and gamma species richness decreased with depth below the lower continental slope in the study area, but ES50 richness trend was not decreased as sharp as alpha and gamma species richness (Fig. 6). Some bathymetrical peaks of species richness in the Arctic and lower-slope have been well documented by other studies^20,69,70^.

We found that the NW Pacific conforms to the recent global findings of species declining with latitude and depth, such that most species occur in tropical coastal depths^7^. Furthermore, the dip in richness observed by Chaudhary *et al*. (2016, 2017) is also present from 0–5° latitude. By calculating alpha, gamma, and ES50 against latitude and depth, we showed that using different diversity indices may influence perceived patterns of species richness over large spatial scales.

## Supplementary information

SI

## Data Availability

The dataset will be available upon the request.
