# Supplementary material for: Latitudinal and bathymetrical species richness patterns in the NW Pacific and adjacent Arctic Ocean
Source: Sci Rep. 2019 Jun 26;9:9303. doi: 10.1038/s41598-019-45813-9 (PMC6594967; doi:10.1038/s41598-019-45813-9)

Latitudinal and bathymetrical species richness patterns in the NW Pacific and adjacent Arctic Ocean

Hanieh Saeedi [1,2,3*], Mark J. Costello [4], Dan Warren [5], Angelika Brandt [1,2]

[1*] Senckenberg Research Institute and Museum, Senckenberganlage 25, 60325 Frankfurt am Main, Germany

[2*] Goethe-University of Frankfurt, FB 15, Institute for Ecology, Evolution and Diversity, Max-von-Laue-Str. 13, 60439 Frankfurt am Main, Germany

[3*] OBIS data manager, Deep-Sea Node, Frankfurt am Main, Germany

[4] Institute of Marine Science, University of Auckland, Auckland 1142, New Zealand

[5] Senckenberg Biodiversity and Climate Research Centre, Senckenberganlage 25, 60325 Frankfurt am Main, Germany

Table S1. Datasets used in this study. The first 145 datasets were extracted from GBIF and the remaining 204 from OBIS. Each is ranked by number of records per dataset.

Dataset title as provided in GBIF or OBIS	Number of Records
NMNH Extant Specimen Records	56000
Fish specimens of Kagoshima University Museum	34000
The molluscs collection (IM) of the Muséum national d'Histoire naturelle (MNHN - Paris)	15000
Zooplankton Eastern Arctic Ocean Polarstern, 1995-1998	12000
CAS Invertebrate Zoology (IZ)	11000
Archives of the Arctic Seas Zooplankton	10000
NOAA Deep Sea Corals Research and Technology Program	7000
Arctic Ocean Diversity	6000
Field Museum of Natural History (Zoology) Fish Collection	6000
Arctic benthic invertebrate collection of the Zoological Institute of the Russian Academy of Science	5000
Queensland Museum provider for OZCAM	5000
Continuous Plankton Recorder Dataset (SAHFOS)	4000
Mollusca collection of National Museum of Nature and Science	4000
Diveboard - Scuba diving citizen science observations	3000
Fish collection of National Museum of Nature and Science	3000
Fishbase	3000
Invertebrates Collection of the Swedish Museum of Natural History	2000
LACM Vertebrate Collection	2000
Ophiuroidea collections of the Zoological Institute Russian Academy of Sciences	2000
Vulnerable marine ecosystems in the South Pacific Ocean region	2000
Composition and distribution of the biomass of zooplankton in the central Arctic Basin 1975, 1976, 1977	1000
Crustacea Collection of Natural History Museum and Institute, Chiba	1000
Fish Collection of Hokkaido University	1000
SIO Marine Vertebrate Collection	1000
Structures and Nutrition Requirements of Macrozoobenthic Communities in the area of the Lomonossov Ridge, 1995-1998	1000
The fishes collection (IC) of the Muséum national d'Histoire naturelle (MNHN - Paris)	1000
UWFC Ichthyology Collection	1000
Museum of Comparative Zoology, Harvard University	863
Diversity of the Indo-Pacific (DIPnet)	859
Mollusca specimens of Toyama Science Museum	858
Biological observations from the Dana Expedition Reports	625
Occurrence records of southern African aquatic biodiversity	584

Museums Victoria provider for OZCAM	566
Natural History Museum (London) Collection Specimens	530
COMARGIS: Information System on Continental Margin Ecosystems	503
RBINS DaRWIN	481
Bernice P. Bishop Museum	475
The echinoderm collection (IE) of the Muséum national d'Histoire naturelle (MNHN - Paris)	469
IPOE_Benthos_Steffens	454
Marine invertabrate(ECHINODERMATA) specimen database of Osaka Museum of Natural History	424
Fish Collection of Natural History Museum and Institute, Chiba	385
Australian Institute of Marine Science (AIMS) - Surveys of Octocorals communities, benthic cover and environmental factors on coral reefs of Palau (2003 - 2005)	376
Vertebrate Zoology Division - Ichthyology, Yale Peabody Museum	343
KUBI Ichthyology Tissue Collection	322
The crustaceans collection (IU) of the Muséum national d'Histoire naturelle (MNHN - Paris)	318
Scleractinia specimens of Kuroshio Biological Research Foundation	294
Arctic Marine Fish Museum Specimens	245
Invertebrate Zoology Division, Yale Peabody Museum	224
Mollusca specimens of Wakayama Prefectural Museum of Natural History	223
Planktic foraminifera abundance of Hole 7-62A	212
Marine Invertebrata specimen database of Osaka Museum of Natutal History	206
Gorgonacea specimens of Kuroshio Biological Research Foundation	197
Zoological Museum Amsterdam, University of Amsterdam (NL) - Bryozoa	188
Australian Institute of Marine Science (AIMS) - Surveys of Octocoral communities, benthic cover and environmental factors on coral reefs of Hong Kong. (1999)	173
Crustacea specimens of Ryukyu University Museum (Fujukan)	157
Natural History Museum Rotterdam (NL) - Mollusca collection	150
Fish Collection of Coastal Branch of Natural History Museum and Institute, Chiba	140
KUBI Ichthyology Collection	134
Echinodermata specimens of Kuroshio Biological Research Foundation	124
Molluscus specimens of Toyohashi Museum of Natural History	107
Galathea II, Danish Deep Sea Expedition 1950-52	106
Kamptozoa collection of National Museum of Nature and Science	99
UF FLMNH Ichthyology	97
Biological Reference Collections ICM CSIC	89
Invertebrates (Type Specimens) of the Swedish Museum of Natural History	74
Macrozoobenthos composition, abundance and biomass in the Arctic Ocean along a transect between Svalbard and the Makarov Basin 1991	69
Catalogue of the type specimens of sea stars (Asteroidea, Echinodermata) from research collections of the Zoological Institute, Russian Academy of Sciences	68
Relative abundance of planktonic foraminifera in Pliocene and Pleistocene sediments of DSDP Hole 32-310 from Hess Rise, Central North Pacific (Table II)	60
Mollusca specimens of Kuroshio Biological Research Foundation	57
Foraminifera abundance of Hole 57-438A	52
Field Museum of Natural History (Zoology) Invertebrate Collection	51
UAM Invertebrate Collection (Arctos)	48
Foraminifera abundance of Hole 61-462	40
Oregon State Ichthyology Collection	38
Biodiversity Research and Teaching Collections - TCWC Vertebrates	32
Distribution data of Arctic species of genus Microporella and Pseudoflustra gathered from museum collections	31
Royal BC Museum - Invertebrates Collection	29
CSIRO Ichthyology provider for OZCAM	28
Five new species of the damselfish genus Chromis (Perciformes: Labroidei: Pomacentridae) from deep coral reefs in the tropical western Pacific	28
Ifremer BIOCEAN database (Deep Sea Benthic Fauna)	26
(Table T17) Planktonic foraminifera stratigraphy of ODP Hole 198-1211A, Zones P14-P18	22

Códigos de barras de la vida en peces y zooplancton de México	22
Catalogue of the type specimens of Pogonophora (Annelida; seu Polychaeta: Siboglinidae) from research collections of the Zoological Institute, Russian Academy of Sciences	21
Range table from planktonic foraminifers in ODP Hole 145-883B	18
orstom_macassar_corindon_1980	17
Type Collection of the Seto Marine Biological Laboratory, Kyoto University	17
(Table T12) Planktonic foraminifera stratigraphy of ODP Hole 198-1210A, Zones P14-P18	15
Foraminifera abundance of Hole 57-438	14
Ostracoda abundance in DSDP Leg 87 holes	14
Antarctic Jellyfish	12
Foraminifera abundance of Hole 57-440B	12
Gunma Museum of Natural History, Molluscan Specimen	12
NIWA Invertebrate Type Collection	11
Catálogo de los equinodermos recientes de México (Fase II)	10
The cnidarians collection (IK) of the Muséum national d'Histoire naturelle (MNHN - Paris)	10
Crustacean specimens of Kitakyushu Museum of Natural History & Human History	9
Planktic foraminifera abundance of Hole 32-305	8
(Table 8) Sample mass, sand fraction and relative abundances of planktonic foraminifera in ODP Holes 127-794A and 127-197B	7
Chaetognatha collection of National Museum of Nature and Science	7
DMNS Marine Invertebrate Collection (Arctos)	7
Planktic foraminifera abundance of Hole 20-200	7
UAM Fish Collection (Arctos)	7
(Table 11) Relative frequencies of planktonic foraminifers and cluster arrangement of ODP Holes 127-794A and 127-197B	6
(Table 5) Range chart of benthic and planktonic foraminifera of ODP Site 127-797	5
Foraminifera abundance of Hole 57-440A	5
Foraminifera abundance of Hole 59-448A	5
Naturalis National Natural History Museum (NL) – Invertebrate specimens from marine expeditions	5
Planktic foraminifera abundance of Hole 6-47B	5
Sizing Ocean Giants	5
(Table 2) Median abundances of macrobenthos in surface sediments	4
Cnidaria Collection of the Seto Marine Biological Laboratory, Kyoto University	4
Echinoderm specimens of Ryukyu University Museum (Fujukan)	4
Foraminifera abundance of Hole 57-440	4
Planktic foraminifera abundance of Hole 60-458	4
Pycnogonidea specimens of Kuroshio Biological Research Foundation	4
Scarabaeoidea y colección de tipos de la colección entomológica del Centro de Estudios en Zoología, Universidad de Guadalajara	4
Atlantic Reference Centre Museum of Canadian Atlantic Organisms - Invertebrates and Fishes Data	3
Deep-water azooxanthellate Scleractinia from Vanuatu, and Wallis and Futuna Islands	3
MNA - Sezione di Genova - (Marine Biological Samples)	3
Northern Territory Museum and Art Gallery provider for OZCAM	3
Sipuncula collection of National Museum of Nature and Science	3
BMSM Bailey-Matthews National Shell Museum	2
Flatworm specimens of Ryukyu University Museum (Fujukan)	2
Fortalecimiento de las colecciones de ECOSUR. Primera fase	2
Luzonichthys seaver, a new species of Anthiinae (Perciformes, Serranidae) from Pohnpei, Micronesia	2
Megafauna of the UKSRL exploration contract area and eastern Clarion-Clipperton Zone in the Pacific Ocean: Annelida, Arthropoda, Bryozoa, Chordata, Ctenophora, Mollusca	2
National Whale and Dolphin Sightings and Strandings Database	2
Planktic foraminifera abundance of Hole 20-199	2
Southern Ocean Ostracoda (except Halocypridina)	2
(Table 3) Range chart of benthic and planktonic foraminifera of ODP Site 127-795	1
ARGOS Satellite Tracking of animals	1
Arthropoda Collection of the Seto Marine Biological Laboratory, Kyoto University	1

Biodiversity Research and Teaching Collections - TCWC Marine Invertebrates	1
Canadian Museum of Nature - Fish Collection (OBIS Canada)	1
CNIN/Triatominos (Hemiptera-Heteroptera-Reduviidae)	1
Foraminifera abundance of Hole 59-448	1
In situ observations of Stygiomedusa gigantea	1
Marine biological observation data from coastal and offshore surveys around New Zealand	1
Megafauna of the UKSRL exploration contract area and eastern Clarion-Clipperton Zone in the Pacific Ocean: Echinodermata	1
Nemertean specimens of Ryukyu University Museum (Fujukan)	1
Planktic foraminifera abundance of Hole 20-200A	1
Planktic foraminifera abundance of Hole 7-62	1
Range table from planktonic foraminifers in ODP Hole 145-883C	1
UCM Fish Collection (Arctos)	1
World Ocean Database 2009	160507
JODC Dataset	88277
CRED Rapid Ecological Assessments of Fish Belt Transect Surveys and Fish Stationary Point Count Surveys in the Pacific Ocean 2000-2010	81325
Fishbase occurrences hosted by GBIF-Sweden	48941
Asia-Pacific Dataset	36482
CRED Rapid Ecological Assessment of Invertebrate in the Pacific Ocean	34904
NMNH Invertebrate Zoology Collections	33851
CRED Rapid Ecological Assessments of Coral Population in the Pacific Ocean 2007-2010	29181
NCOS1959_Crustacea	25522
Marine Biological Sample Database, JAMSTEC	23906
Biodiversity Research Museum, Academia Sinica, Taiwan	19833
NMNH Vertebrate Zoology Fishes Collections	18436
KOBIS database	16433
NCOS1959_Mollusca	15905
University of Florida Museum of Natural History Invertebrate Zoology Collection	12697
Hexacorallians of the World	10747
North Pacific Groundfish Observer	10228
Ichthyology Collection - Royal Ontario Museum	9607
Fish specimens	9503
CRED Rapid Ecological Assessment of Benthic Habitat Cover in the Pacific Ocean 2005-2010	9329
Survey data of tidal flats on the Monitoring sites 1000 project, BDCJ	7883
NCOS	7859
NCOS1959_Echinodermata	7714
Continuous Plankton Recorder Dataset (SAHFOS) - Pacific Zooplankton	7176
Benthic species from the tropical Pacific surrounding New Caledonia	6762
CRED Towed-Diver Fish Biomass Surveys in the Pacific Ocean 2000-2010	5177
Academy of Natural Sciences OBIS Mollusc Database	4926
Historical distribution of whales shown by logbook records 1785-1913	4120
Ichtyologie	3752
Taiwan bottom trawl survey	3529
obis_mbmcas_scs_i	2860
PANGAEA - Data from various sources	2694
Bishop Museum Data (OBIS distribution)	2674
obis_mbmcas_ys_i	2562
TOPP Summary of SSM-derived Telemetry	2412
Arctic Species Trend Index (ASTI) : Marine	2331
obis_mbmcas_ys_ii	2271
BOLD Public Fish Data	2097
ECOCEAN Whale Shark Photo-identification Library	2020
Land-based observations of cetaceans in the waters of Beringa Island in 2007-2009	1960
CMarZ (Census of Marine Zooplankton)-Asia Database	1892
obis_mbmcas_scs_ii	1846

IPHC seabird survey 2002-2011	1680
NaGISA Project	1639
PANGAEA - data from Archive of Ocean Data (ArcOD)	1588
Type locality distributions from the World Register of Marine Species	1507
SeamountsOnline (Seamount Biota)	1428
Collection Bryozoa SMF	1426
PANGAEA - Data from marine animal ecology @ AWI	1271
obis_iocas_ecs_i	1211
National Taiwan Museum	1063
IndOBIS, Indian Ocean Node of OBIS	1059
obis_iocas_esc_ii	1057
Diversity and community structure of pelagic cnidarians in south-east Asian tropical marginal Seas	1010
CeDAMar database for benthic biological sampling on the abyssal plains	967
ZINRAS_Arctic_Benthos	952
Taxonomically comprehensive assessment of biodiversity of animal plankton throughout the world ocean	927
Arctic Ostracode database 2010	809
NMFS Turtle Tracking (aggregated per 1-degree cell)	737
Cnidaria Anthozoa: Azooxanthellate Scleractinia from the Philippine and Indonesian regions	716
PANGAEA - Data from quaternary environment of the Eurasian North (QUEEN)	701
Project for Reef Fish Monitoring	686
Yangtze finless porpoise in Yangtze River, China 2006	640
asean_fish_occurrences_literature	630
PANGAEA - Data from the Ocean Drilling Program (ODP)	600
Survey data of rocky shores on the Monitoring sites 1000 project, BDCJ	585
JAMSTEC_MULTISPLASH2006_SagamiBay_Siphonophora	553
MICROBIS database	504
South African Institute for Aquatic Biodiversity - Fish Collection	468
Meiobenthos and nematodes from the continental shelf of the Laptev Sea	448
CRED Rapid Ecological Assessments of Coral Health and Disease in the Pacific Ocean 2005-2008	445
Wildbook for Whale Sharks	421
Australian Institute of Marine Science for Zooplankton community structure in Nearshore waters of the Great Barrier Reef.	397
CephBase	392
Senckenberg's collection management system	377
Australian Institute of Marine Science - Surveys of Octocorals communities, benthic cover and environmental factors on coral reefs of Palau.	376
Loggerhead turtle movement off the coast of Taiwan (aggregated per 1-degree cell)	360
iziko South African Museum - Shark Collection	351
TOPP Albatrosses 2002-06 (aggregated per 1-degree cell)	304
Echinoderm collection	274
DFO Pacific IOS zooplankton database - Zooplankton samples collected during cruises to the Canadian Arctic between 1987 and 2000	265
Fish Species Observed in the Hawaii Exclusive Economic Zone from the 1750s through 2003.	252
ChEssBase	242
PANGAEA - Data from the Deep Sea Drilling Project (DSDP)	235
asean_fish_occurrences_museums	209
Ocean Genome Resource	206
Zoobenthos data from different sources (collected and extracted from literature), personal archive of G.V. Murina (IBSS)	191
Australian Institute of Marine Science - Surveys of Octocoral communities, benthic cover and environmental factors on coral reefs of Hong Kong.	173
Tern Island Albatrosses - 1999 (aggregated per 1-degree cell)	160
BOLD Marine Invertebrate Data	159
SWFSC juvenile loggerhead sea turtle tracking 2002-2005 (aggregated per 1-degree cell)	153
NPPSD Short-tailed Albatross Sightings	142

IndOBIS Dataset (36001-38000)	140
Nematodes of the central Arctic Ocean	140
Lamont-Doherty/LGL/NSF cruises	121
IndOBIS Dataset (34001-36000)	117
East London Museum	114
Satellite telemetry of King Eiders from northern Alaska 2002-2009 (aggregated per 1-degree cell)	114
IndOBIS Dataset (32001-34000)	113
Distribution of small cetaceans in the nearshore waters of Sarawak, East Malaysia	112
Cold water corals	107
TOPP Fish (TOPP)	107
Green sea turtle satellite tracking in the Nansha and Penghu Archipelagos 2000-2005 (aggregated per 1-degree cell)	106
IndOBIS Dataset (14001-16000)	106
Tern Island Albatrosses - 1998 (aggregated per 1-degree cell)	105
Deep-sea Meiobenthos	96
IndOBIS Dataset (12001-14000)	96
Collection Cnidaria SMF	95
HMAP Dataset 21: Gulf of Thailand cephalopod fisheries	93
Pacific Turtle Tracks: Grupo Tortuguero (aggregated per 1-degree cell)	92
Cetacean observations in the waters of Karaginsky Island and Beringa Island in 2009	78
IndOBIS Dataset (16001-18000)	65
MV Ichthyology	62
Vietnam Sea Turtle Tracking Project (aggregated per 1-degree cell)	59
Cetacean observations in Avacha Gulf 2005-2009	53
Catalogue of Squat Lobsters	51
IndOBIS Dataset (6001-8000)	50
Northern Elephant Seals Post-Molting 2016 (aggregated per 1-degree cell)	48
IndOBIS Dataset (30001-32000)	46
Satellite tracked green turtles in the South China Sea 1993-1994 (aggregated per 1-degree cell)	46
Circumpolar Seabird Monitoring Plan	45
Records from the NIWA AllSeaBio database	45
Identification of Important Turtle Areas for green turtles in the Sulu Sulawesi Marine Ecoregion (aggregated per 1-degree cell)	43
IndOBIS Dataset (38001-40000)	43
IndOBIS Dataset (10001-12000)	41
IndOBIS Dataset (28001-30000)	39
Shallow-water fauna of Sagami Bay	37
IndOBIS Dataset (8001-10000)	34
Global distribution of Manta and Mobula rays - A citizen science project based upon a survey of divers	33
Cetacean survey in Balabac Strait, Philippines	32
IndOBIS Dataset (4001-6000)	30
IndOBIS Dataset (18001-20000)	29
Northern elephant seals post-molting 2014 (aggregated per 1-degree cell)	26
IndOBIS Dataset (20001-22000)	25
Continuous Plankton Recorder Dataset (SAHFOS) - Pacific Phytoplankton	22
iziko South African Museum - Fish Collection	22
Naturalis National Natural History Museum (NL) - Crustacea Decapoda	21
IndOBIS Dataset	20
CNMI Sea Turtle Program satellite tracked green turtles 2011 (aggregated per 1-degree cell)	19
Piai Island Green Sea Turtle Tracking (aggregated per 1-degree cell)	19
Cetacean observations in the Indian Ocean Sanctuary and the South China Sea, Mauritius to the Philippines, April 1999	18
Sangalaki Green Turtles Tracking (aggregated per 1-degree cell)	18
Australian Institute of Marine Science - Bioresources Library	17
Holothuroidea distribution data from: Deep-sea fauna of European seas - an annotated species check-list of benthic invertebrates living deeper than 2000 m in the seas bordering Europe	16

IPHC Opportunistic Albatross Obs 1998-2002	16
MIRC Vessel Winter Surveys 2011	16
MV Marine Invertebrates	16
TOPP northern elephant seal (Mirounga angustirostris) ARGOS satellite tracking	16
ZooGene A DNA Sequence Database for Calanoid Copepods and Euphausiids	16
Scleractinia of Eastern Australia - AIMS Monograph Series - John (Charlie) Veron	15
IndOBIS Dataset (24001-26000)	13
iziko South African Museum - Crustacean Collection	13
IndOBIS Dataset (26001-28000)	12
Satellite Tracking of Hawksbill Turtles in Melaka, Malaysia (aggregated per 1-degree cell)	12
CRED REA Algal Quadrate Images in the Pacific Ocean 2002-2008	11
Distribution of Hydrothermal Alvinocaridid Shrimps	11
Biocean	10
Identification of secondary foraging grounds for green turtles as they depart Mantanani, Malaysia (aggregated per 1-degree cell)	10
IndOBIS Dataset (22001-24000)	9
IndOBIS Dataset (48001-50000)	9
IndOBIS Dataset (50001-52000)	9
MIRC Guam Marine Species Monitoring Winter-Spring Surveys 2012	9
South Western Pacific Regional OBIS Data Specify Subset	9
Collection Porifera SMF	8
MIRC Saipan Marine Species Monitoring Winter-Spring Surveys 2012	8
UK Royal Navy Marine Mammal Observations	8
Biological observations from the Discovery Investigations 1925-1952	7
Catches or strandings of Balaenoptera omurai	7
IndOBIS Dataset (90001-92000)	7
Extensive sympatry, cryptic diversity and introgression throughout the Indo-Pacific distribution of two coral species complexes	6
Naturalis Invertebrate specimens from marine expeditions	6
NMML 2002-2010 Bering Sea Shelf Cetacean Survey	6
Ophiuroidea distribution data from: Deep-sea fauna of European seas - an annotated species check-list of benthic invertebrates living deeper than 2000 m in the seas bordering Europe	6
Palau Marine Turtle Conservation and Monitoring Program (aggregated per 1-degree cell)	6
Aerial sightings of beluga in the Russian Arctic seas 2010	5
Collection Polychaeta SMF	5
IndOBIS Dataset (40001-42000)	5
Taxonomic Information System for the Belgian coastal area	5
USAKA Turtle Release Program (aggregated per 1-degree cell)	5
Whimbrel Tracking in the Americas (aggregated per 1-degree cell)	5
Satellite tracking project, Pulau Banyak, Aceh, Sumatra, Indonesia (aggregated per 1-degree cell)	4
Satellite Tracking Project-Marinelife Alliance (aggregated per 1-degree cell)	4
Australian Museum	3
Marine apostome ciliates	3
Northern elephant seals post-molting 2015 MALES (aggregated per 1-degree cell)	3
Tracking on Magnifying Olive ridley Journey in Kaironi beach, Papua-Indonesia (aggregated per 1-degree cell)	3
Bryozoa distribution data from: Deep-sea fauna of European seas - an annotated species check-list of benthic invertebrates living deeper than 2000 m in the seas bordering Europe	2
Collection Copepoda SMF	2
Crinoidea distribution data from: Deep-sea fauna of European seas - an annotated species check-list of benthic invertebrates living deeper than 2000 m in the seas bordering Europe	2
Cascadia Research Blue Whale Photo IDs for US West Coast, 1972-2004	1
Cirripedia distribution data from: Deep-sea fauna of European seas - an annotated species check-list of benthic invertebrates living deeper than 2000 m in the seas bordering Europe	1
Deep-Sea Hydrothermal Vent Endemic Brachyuran Family Bythograeidae	1
Derawan Green Turtles Tracking (aggregated per 1-degree cell)	1

Echinoidea distribution data from: Deep-sea fauna of European seas - an annotated species check-list of benthic invertebrates living deeper than 2000 m in the seas bordering Europe	1
Ecsenius niue, new species of blenniid fish, and new distribution records for other species in the Opsifrontalis species group	1
iziko South African Museum - Marine Mammal Collection	1
iziko South African Museum - Mollusc Collection	1
Mysida and Lophogastrida distribution data from: Deep-sea fauna of European seas - an annotated species check-list of benthic invertebrates living deeper than 2000 m in the seas bordering Europe	1
Pycnogonida distribution data from: Deep-sea fauna of European seas - an annotated species check-list of benthic invertebrates living deeper than 2000 m in the seas bordering Europe	1
SAM Herpetology	1
SOMBASE BIOCONSTRUCTORS	1
The Azooxanthellate Scleractinia (Coelenterata: Anthozoa) of Australia	1
Tulane University Museum of Natural History	1
USGS Alaska Science Center Polar Bear Maternal Dens	1

Supplementary Figures

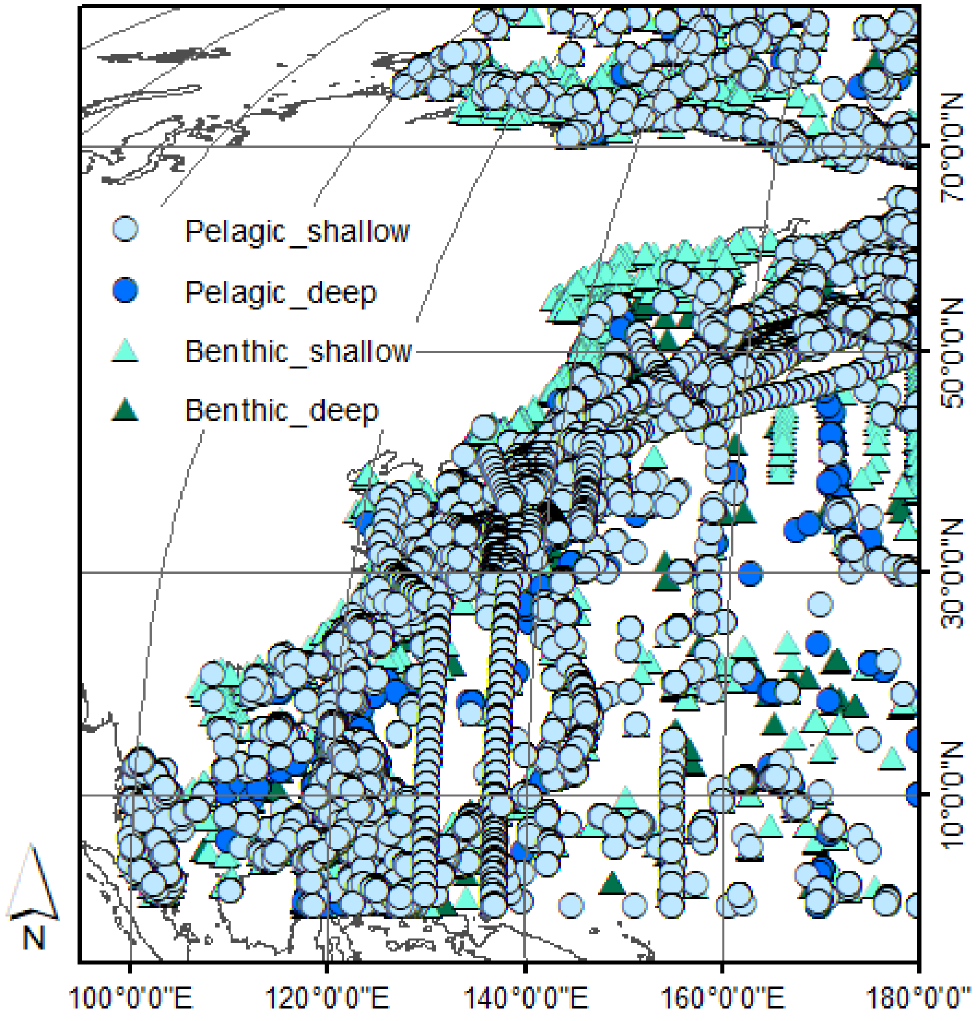

Fig. S1. Distribution map of pelagic and benthic shallow water (0-500m) and deep-sea (deeper than 500m) species in the NW Pacific and adjacent Arctic Ocean. In total 352,969 distribution records of four groups were mapped.

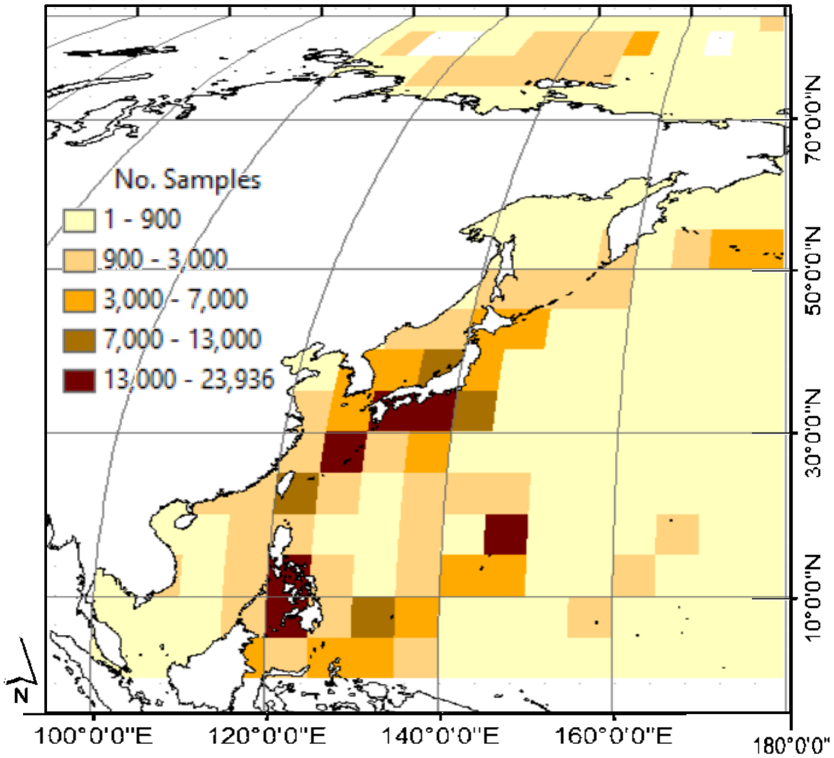

Fig. S2. Number of records in each 5˚N latitude-longitude cell of the NW Pacific and adjacent Arctic Ocean. The Japan, East China, Philippine Seas had the highest number of records; from 13,000 to around 24,000 records.

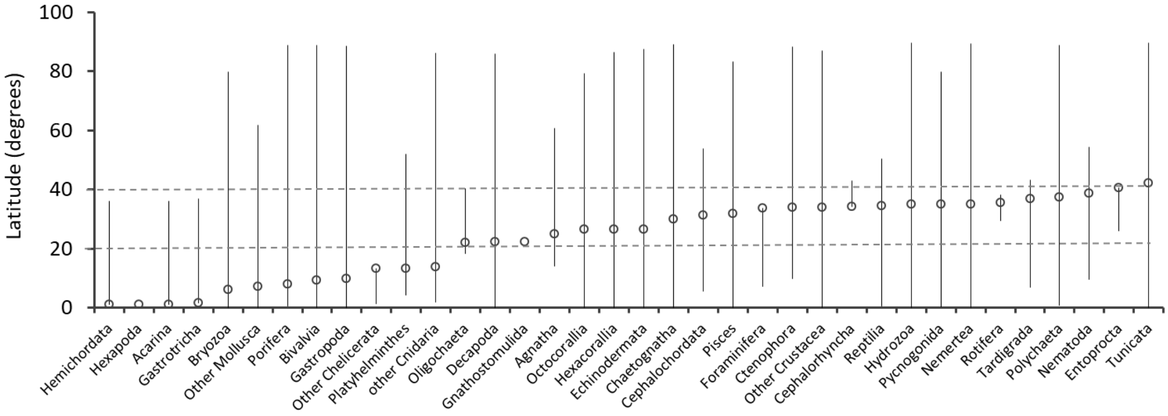

Fig. S3. Latitudinal ranges of higher taxa. The hollow circles show the mode of the distribution records, and indicator of sampling effort (not species richness), in each taxon.

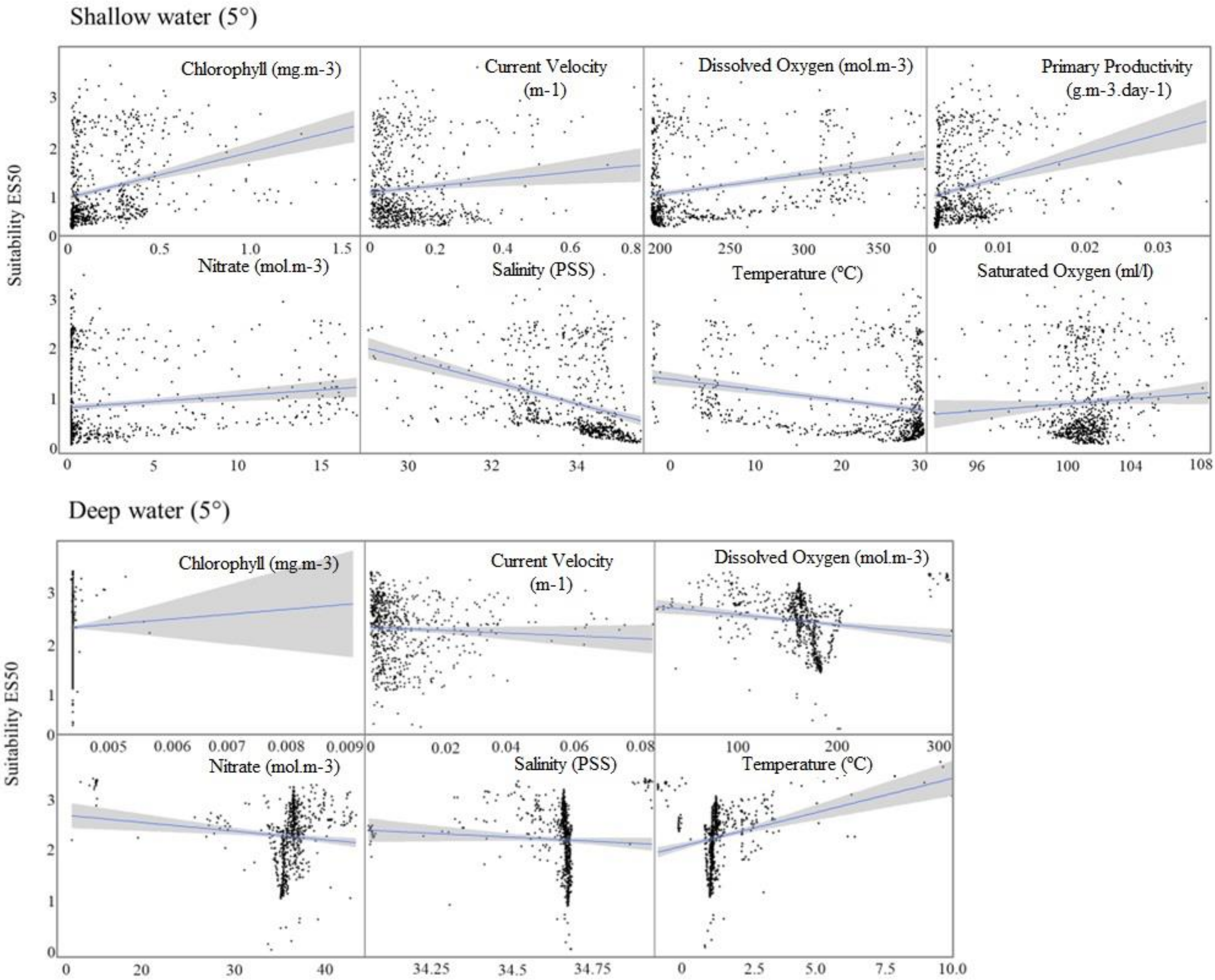

Fig. S4. Correlation matrix between suitability ES50 (suitable responses of ES50 to the environmental predictor variables) and environmental factors used in GAMs models for both shallow water (0-500 m) (top) and deep sea (>500 m) (bottom) species.

S1. Species Counts and Environment Based on Hexagons.

Setup

Shallow water

Deep water

Species Counts and Environment

Setup

Shallow water

Data load-in and visualization

First we're going to load in our data and then trim the data frame down to just the columns we need.

```
shallow <- read.csv("shallow.csv")
analysis.cols <- c("lat", "lon", "area", "num.records", "num.species", "ES50", "dis
solved.oxygen", "temp", "primary.productivity", "chlorophyll", "current", "o2.satur
ate", "salinity", "nitrate")
shallow <- shallow[,analysis.cols]
shallow <- shallow[complete.cases(shallow),]
corrplot(cor(shallow))
```

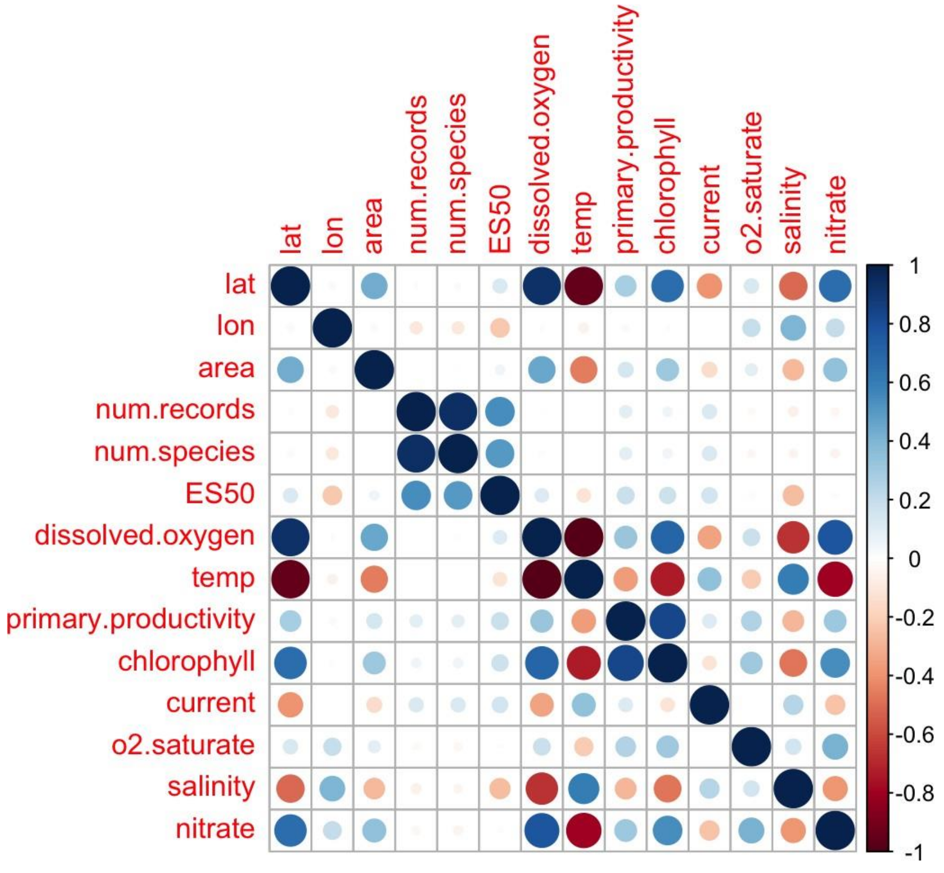

GAMs for number of species, shallow water

We're going to develop a number of GAMs here. The "intercept" GAM represents the fit of a model that assumes no relationship between the species counts and the environment and no spatial autocorrelation. The "latlon" model represents the fit of a model that fits spatial autocorrelation, but no environmental effects. The "env" model represents the combined effects of all environmental predictors, and the remainder of the models ("temp", "oxygen", etc.) estimate the effects of a single predictor at a time.

```
shallow.numsp.intercept <- gam(num.species ~ 1, data = shallow, family = "nb", meth
od = "REML", select = TRUE)
gam.check(shallow.numsp.intercept)
```

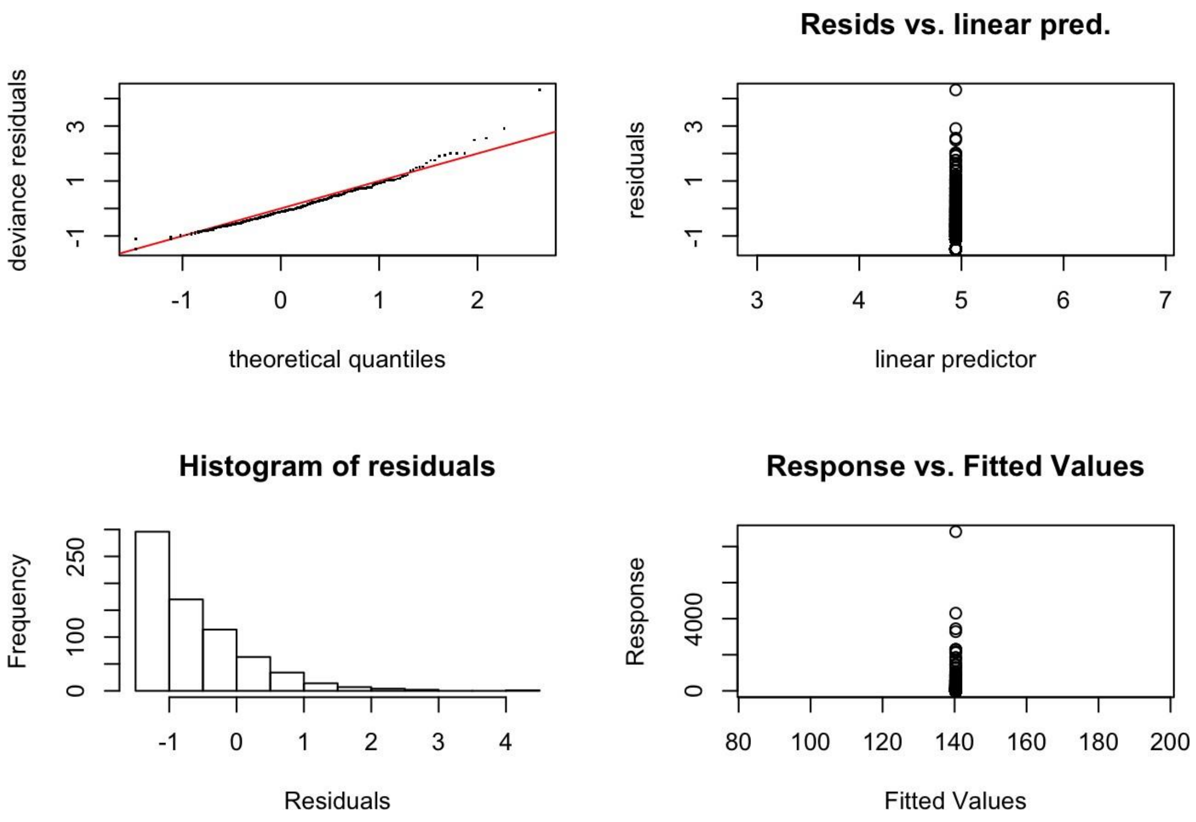


```
##
## Method: REML   Optimizer: outer newton
## full convergence after 5 iterations.
## Gradient range [2.33879e-06,2.33879e-06]
## (score 3116.071 & scale 1).
## Hessian positive definite, eigenvalue range [382.9675,382.9675].
## Model rank =  1 / 1
```

```
summary(shallow.numsp.intercept)
```

```
##
## Family: Negative Binomial(0.161)
## Link function: log
##
## Formula:
## num.species ~ 1
##
## Parametric coefficients:
##                 Estimate Std. Error z value Pr(>|z|)
## (Intercept)      4.94395    0.09386   52.67   <2e-16 ***
## ---
## Signif. codes:  0 '***' 0.001 '**' 0.01 '*' 0.05 '.' 0.1 ' ' 1
##
##
## R-sq.(adj) =      0   Deviance explained = 1.56e-09%
## -REML = 3116.1  Scale est. = 1         n = 705
```

```
shallow.numsp.numrec <- gam(num.species ~ s(num.records), data = shallow, family =
"nb", method = "REML", select = TRUE)
gam.check(shallow.numsp.numrec)
```

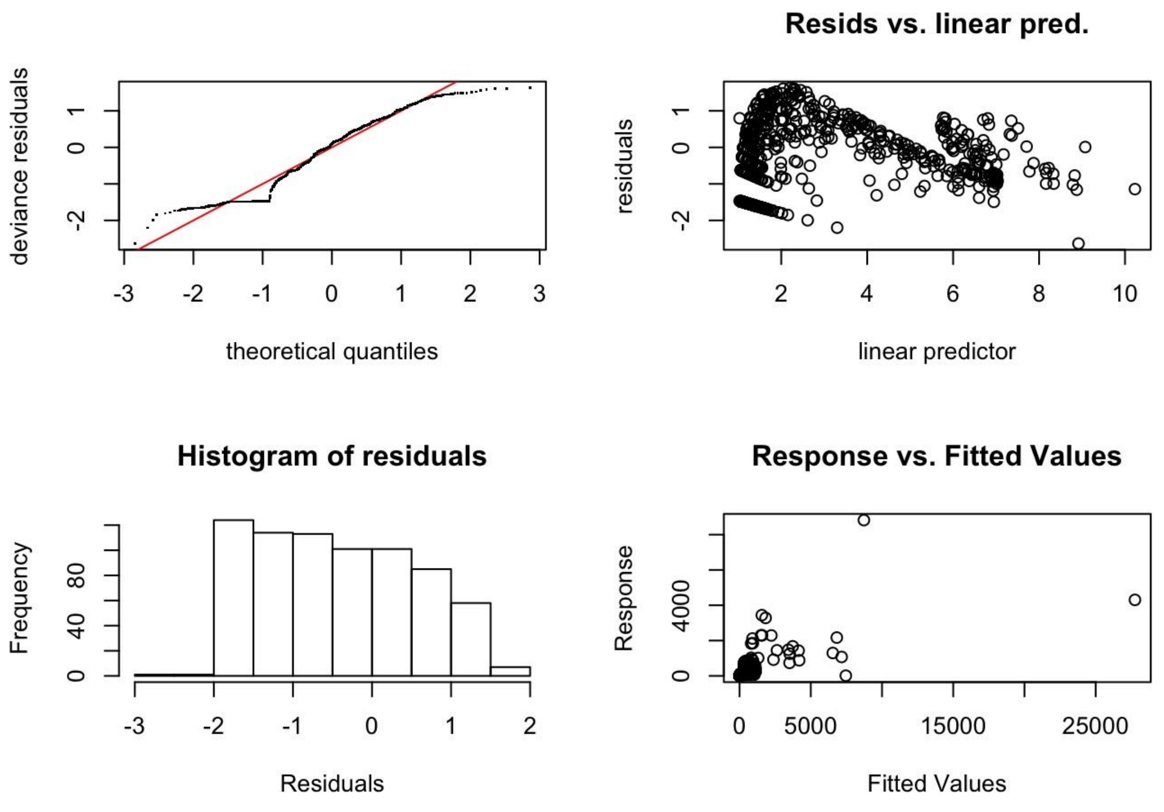

```
##
## Method: REML   Optimizer: outer newton
## full convergence after 9 iterations.
## Gradient range [-3.306909e-05,0.0006015526]
## (score 2711.477 & scale 1).
## Hessian positive definite, eigenvalue range [0.4966568,212.487].
## Model rank =  10 / 10
##
## Basis dimension (k) checking results. Low p-value (k-index<1) may
## indicate that k is too low, especially if edf is close to k'.
##
##                    k'  edf k-index p-value
## s(num.records) 9.00 8.86    0.43  <2e-16 ***
## ---
## Signif. codes:  0 '***' 0.001 '**' 0.01 '*' 0.05 '.' 0.1 ' ' 1
```

```
summary(shallow.numsp.numrec)
```

```
##
## Family: Negative Binomial(0.641)
## Link function: log
##
## Formula:
## num.species ~ s(num.records)
##
## Parametric coefficients:
##             Estimate Std. Error z value Pr(>|z|)
## (Intercept)  2.75008    0.04959   55.46   <2e-16 ***
## ---
## Signif. codes:  0 '***' 0.001 '**' 0.01 '*' 0.05 '.' 0.1 ' ' 1
##
## Approximate significance of smooth terms:
##                  edf Ref.df Chi.sq  p-value
## s(num.records) 8.855      9   1964  <2e-16 ***
## ---
## Signif. codes:  0 '***' 0.001 '**' 0.01 '*' 0.05 '.' 0.1 ' ' 1
##
## R-sq.(adj) =  -3.39   Deviance explained =  71%
## -REML = 2711.5 Scale est. = 1         n = 705
```

```
shallow.numsp.latlon <- gam(num.species ~ s(lat, lon, bs = "sos") + s(num.records), data =
shallow, family = "nb", method = "REML", select = TRUE)
gam.check(shallow.numsp.latlon)
```

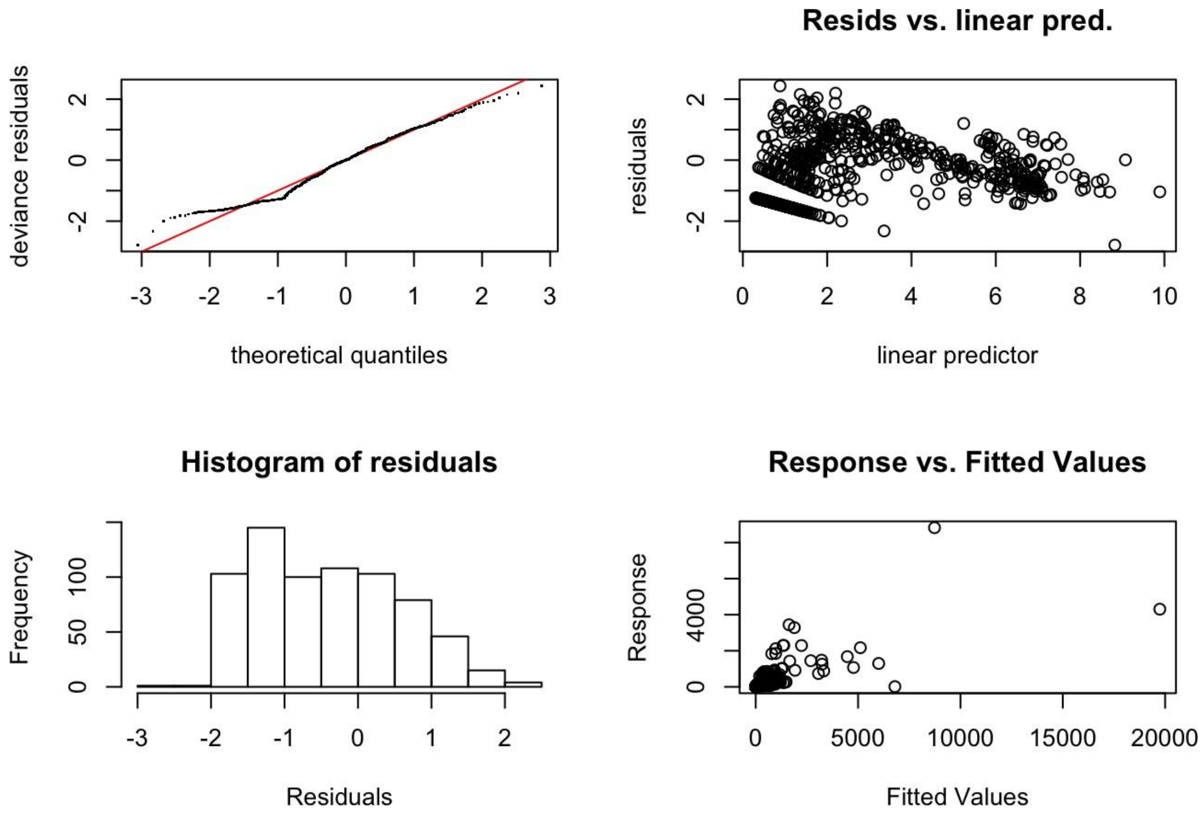


```
##
## Method: REML   Optimizer: outer newton
## full convergence after 12 iterations.
## Gradient range [-9.356918e-05,0.001657861]
## (score 2690.814 & scale 1).
## Hessian positive definite, eigenvalue range [0.4975741,193.3047].
## Model rank =  59 / 59
##
## Basis dimension (k) checking results. Low p-value (k-index<1) may
## indicate that k is too low, especially if edf is close to k'.
##
##                   k'   edf k-index p-value
## s(lat,lon)     49.00 12.82    0.74  <2e-16 ***
## s(num.records)  9.00  8.84    0.48  <2e-16 ***
## ---
## Signif. codes:  0 '***' 0.001 '**' 0.01 '*' 0.05 '.' 0.1 ' ' 1
```

```
summary(shallow.numsp.latlon)
```

```
##
## Family: Negative Binomial(0.73)
## Link function: log
##
## Formula:
## num.species ~ s(lat, lon, bs = "sos") + s(num.records)
##
## Parametric coefficients:
##             Estimate Std. Error z value Pr(>|z|)
## (Intercept)   2.6640     0.0474    56.2   <2e-16 ***
## ---
## Signif. codes:  0 '***' 0.001 '**' 0.01 '*' 0.05 '.' 0.1 ' ' 1
##
## Approximate significance of smooth terms:
##                   edf Ref.df   Chi.sq  p-value
## s(lat,lon)     12.824     49    66.67 1.85e-12 ***
## s(num.records)  8.844      9  1474.16  < 2e-16 ***
## ---
## Signif. codes:  0 '***' 0.001 '**' 0.01 '*' 0.05 '.' 0.1 ' ' 1
##
## R-sq.(adj) =  -1.26   Deviance explained = 74.5%
## -REML = 2690.8  Scale est. = 1         n = 705
```

```
shallow.numsp.temp <- gam(num.species ~ s(lat, lon, bs = "sos") + s(num.records) +
s(temp), data = shallow, family = "nb", method = "REML", select = TRUE)
gam.check(shallow.numsp.temp)
```

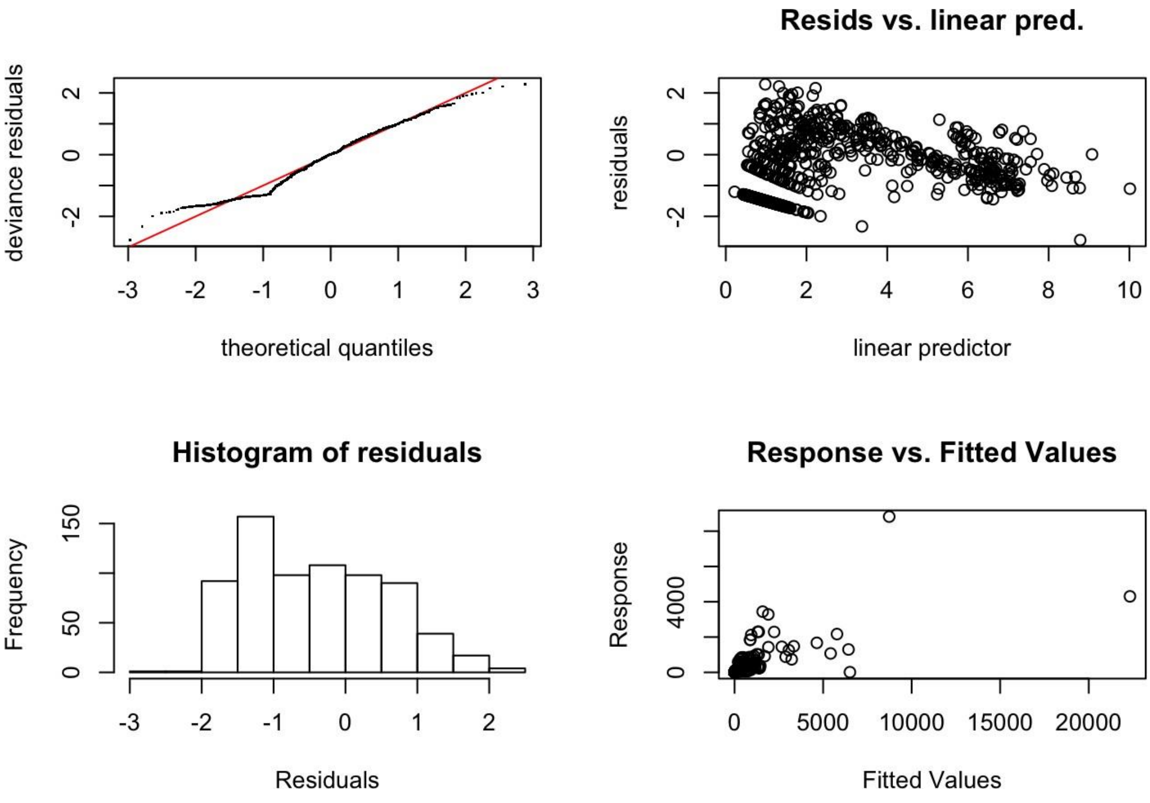

```
##
## Method: REML   Optimizer: outer newton
## full convergence after 12 iterations.
## Gradient range [-9.719285e-05,0.0007917026]
## (score 2689.112 & scale 1).
## Hessian positive definite, eigenvalue range [9.717277e-05,195.1371].
## Model rank =  68 / 68
##
## Basis dimension (k) checking results. Low p-value (k-index<1) may
## indicate that k is too low, especially if edf is close to k'.
##
##                    k'   edf k-index p-value
## s(lat,lon)      49.00  7.53    0.74  <2e-16 ***
## s(num.records)   9.00  8.84    0.48  <2e-16 ***
## s(temp)          9.00  2.15    0.89    0.88
## ---
## Signif. codes:  0 '***' 0.001 '**' 0.01 '*' 0.05 '.' 0.1 ' ' 1
```

```
summary(shallow.numsp.temp)
```

```
##
## Family: Negative Binomial(0.727)
## Link function: log
##
## Formula:
## num.species ~ s(lat, lon, bs = "sos") + s(num.records) + s(temp)
##
## Parametric coefficients:
##             Estimate Std. Error z value Pr(>|z|)
## (Intercept)  2.67036    0.04745   56.28   <2e-16 ***
## ---
## Signif. codes:  0 '***' 0.001 '**' 0.01 '*' 0.05 '.' 0.1 ' ' 1
##
## Approximate significance of smooth terms:
##                  edf Ref.df   Chi.sq  p-value
## s(lat,lon)     7.533     49   34.065 6.73e-08 ***
## s(num.records) 8.840      9 1555.054  < 2e-16 ***
## s(temp)        2.147      9    9.394 2.54e-05 ***
## ---
## Signif. codes:  0 '***' 0.001 '**' 0.01 '*' 0.05 '.' 0.1 ' ' 1
##
## R-sq.(adj) =  -1.82   Deviance explained = 74.2%
## -REML = 2689.1  Scale est. = 1         n = 705
```

```
shallow.numsp.oxygen <- gam(num.species ~ s(lat, lon, bs = "sos") + s(num.records)
 + s(dissolved.oxygen), data = shallow, family = "nb", method = "REML", select = T
RUE)
gam.check(shallow.numsp.oxygen)
```

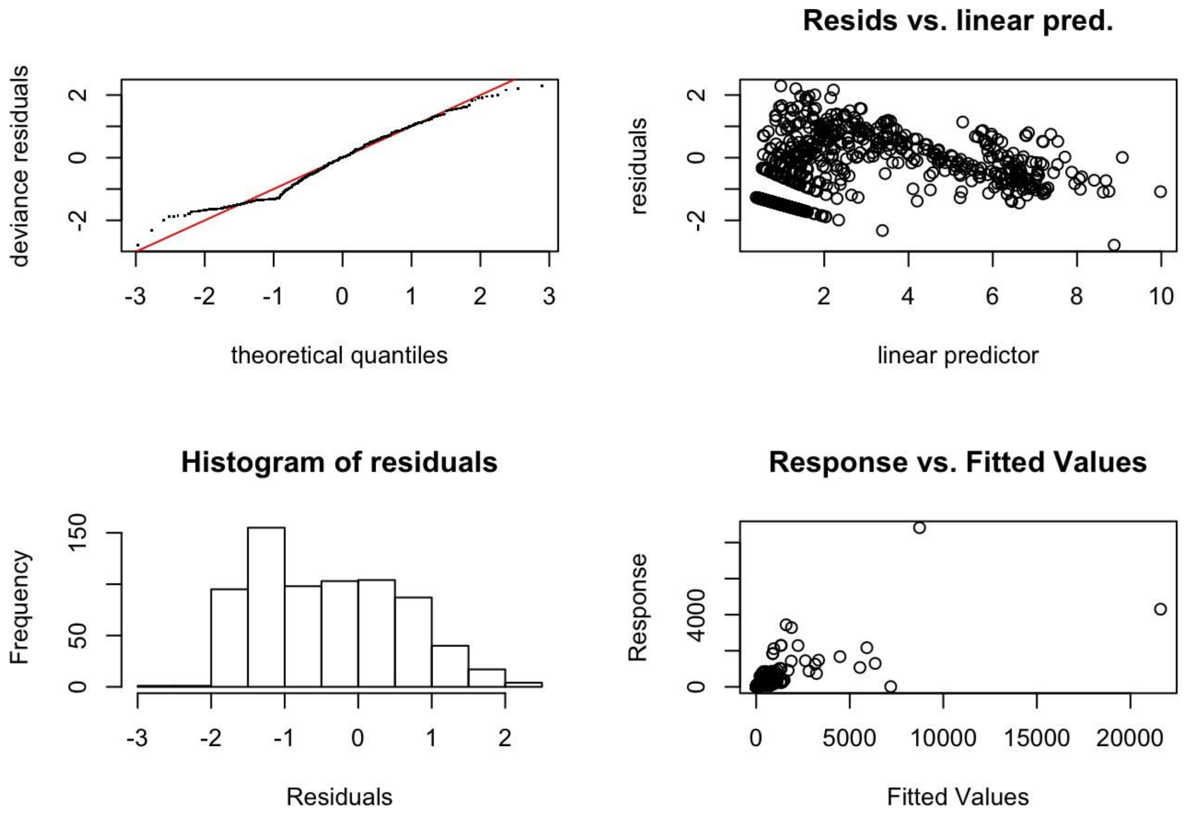


```
##
## Method: REML   Optimizer: outer newton
## full convergence after 17 iterations.
## Gradient range [-0.0001833293,0.001605755]
## (score 2689.23 & scale 1).
## Hessian positive definite, eigenvalue range [0.0001832544,195.7092].
## Model rank =  68 / 68
##
## Basis dimension (k) checking results. Low p-value (k-index<1) may
## indicate that k is too low, especially if edf is close to k'.
##
##                       k'  edf k-index p-value
## s(lat,lon)         49.00 7.40    0.74   0.005 **
## s(num.records)      9.00 8.84    0.48  <2e-16 ***
## s(dissolved.oxygen) 9.00 1.98    0.84   0.495
## ---
## Signif. codes:  0 '***' 0.001 '**' 0.01 '*' 0.05 '.' 0.1 ' ' 1
```

```
summary(shallow.numsp.oxygen)
```

```
##
## Family: Negative Binomial(0.725)
## Link function: log
##
## Formula:
## num.species ~ s(lat, lon, bs = "sos") + s(num.records) + s(dissolved.oxygen)
##
## Parametric coefficients:
##             Estimate Std. Error z value Pr(>|z|)
## (Intercept)   2.6720     0.0475   56.26   <2e-16 ***
## ---
## Signif. codes:  0 '***' 0.001 '**' 0.01 '*' 0.05 '.' 0.1 ' ' 1
##
## Approximate significance of smooth terms:
##                       edf Ref.df   Chi.sq  p-value
## s(lat,lon)          7.395     49   34.122 5.08e-08 ***
## s(num.records)      8.839      9 1554.175  < 2e-16 ***
## s(dissolved.oxygen) 1.977      9    9.037 2.38e-05 ***
## ---
## Signif. codes:  0 '***' 0.001 '**' 0.01 '*' 0.05 '.' 0.1 ' ' 1
##
## R-sq.(adj) =  -1.72   Deviance explained = 74.2%
## -REML = 2689.2  Scale est. = 1         n = 705
```

```
shallow.numsp.prod <- gam(num.species ~ s(lat, lon, bs = "sos") + s(num.records) + s(primary.productivity), data = shallow, family = "nb", method = "REML", select = TRUE)
gam.check(shallow.numsp.prod)
```

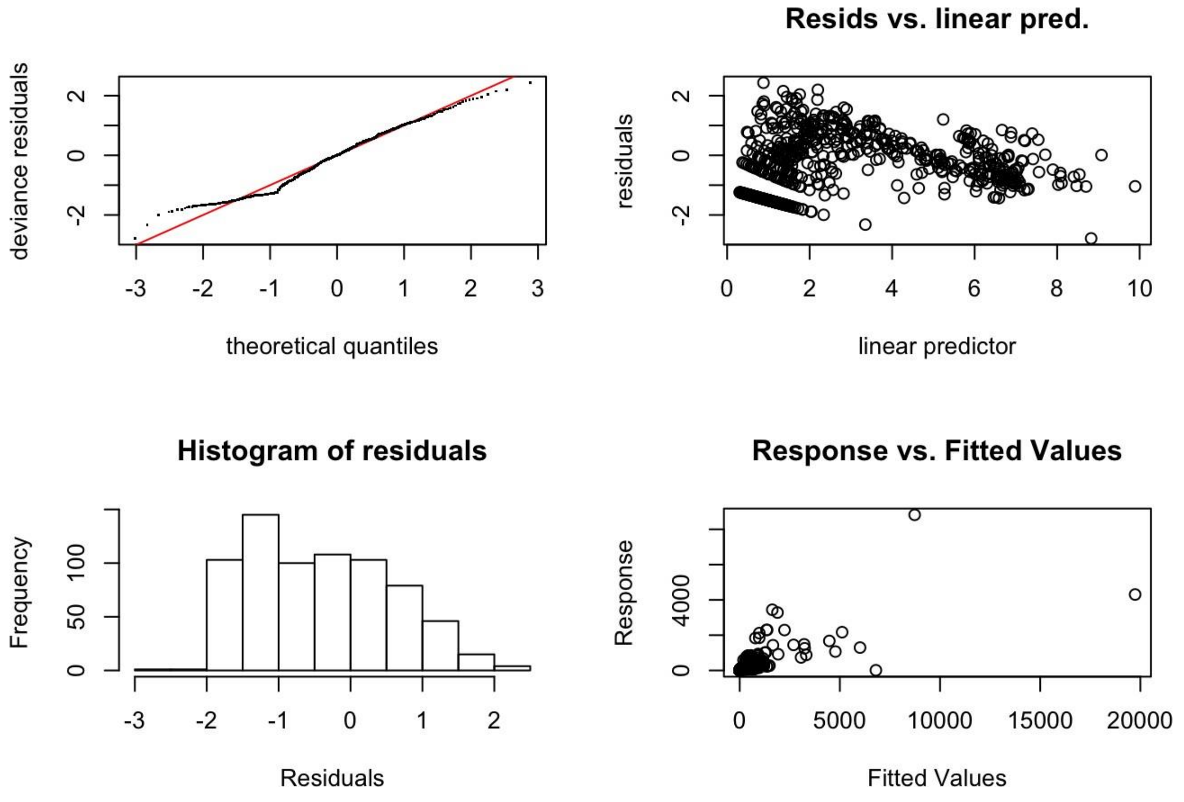

```
##
## Method: REML   Optimizer: outer newton
## full convergence after 17 iterations.
## Gradient range [-0.0002133841,0.001419792]
## (score 2690.815 & scale 1).
## Hessian positive definite, eigenvalue range [0.0001387295,193.3042].
## Model rank =  68 / 68
##
## Basis dimension (k) checking results. Low p-value (k-index<1) may
## indicate that k is too low, especially if edf is close to k'.
##
##                                k'      edf k-index p-value
## s(lat,lon)                49.00000 12.82396    0.74  <2e-16 ***
## s(num.records)             9.00000  8.84415    0.48  <2e-16 ***
## s(primary.productivity)    9.00000  0.00125    0.83    0.31
## ---
## Signif. codes:  0 '***' 0.001 '**' 0.01 '*' 0.05 '.' 0.1 ' ' 1
```

```
summary(shallow.numsp.prod)
```

```
##
## Family: Negative Binomial(0.73)
## Link function: log
##
## Formula:
## num.species ~ s(lat, lon, bs = "sos") + s(num.records) + s(primary.productivity)
##
## Parametric coefficients:
##             Estimate Std. Error z value Pr(>|z|)
## (Intercept)   2.6640     0.0474    56.2   <2e-16 ***
## ---
## Signif. codes:  0 '***' 0.001 '**' 0.01 '*' 0.05 '.' 0.1 ' ' 1
##
## Approximate significance of smooth terms:
##                               edf Ref.df    Chi.sq  p-value
## s(lat,lon)              12.823963     49    66.663 1.85e-12 ***
## s(num.records)           8.844153      9  1474.147  < 2e-16 ***
## s(primary.productivity)  0.001252      9     0.001    0.424
## ---
## Signif. codes:  0 '***' 0.001 '**' 0.01 '*' 0.05 '.' 0.1 ' ' 1
##
## R-sq.(adj) =  -1.26   Deviance explained = 74.5%
## -REML = 2690.8  Scale est. = 1         n = 705
```

```
shallow.numsp.chlorophyll <- gam(num.species ~ s(lat, lon, bs = "sos") + s(num.records)
+ s(chlorophyll), data = shallow, family = "nb", method = "REML", select = TRUE)
gam.check(shallow.numsp.chlorophyll)
```

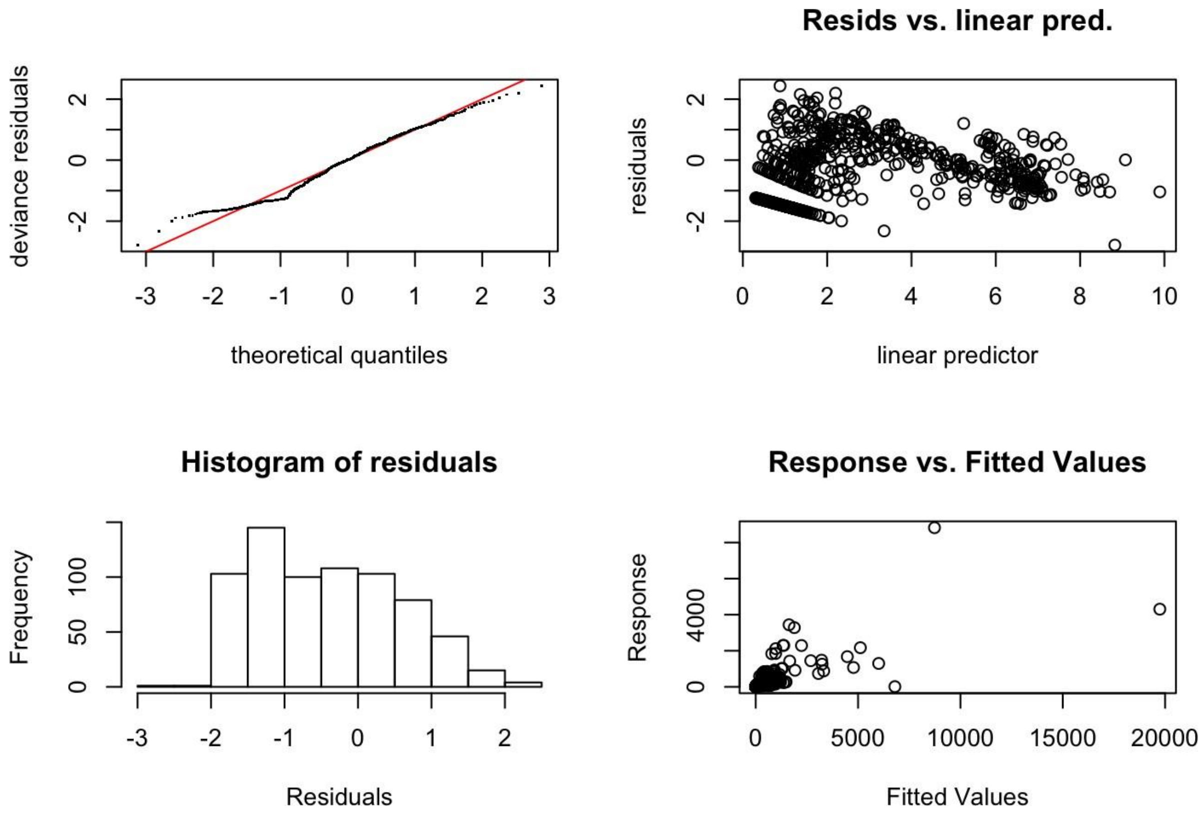


```
##
## Method: REML   Optimizer: outer newton
## full convergence after 17 iterations.
## Gradient range [-0.0001761506,0.0009546675]
## (score 2690.814 & scale 1).
## Hessian positive definite, eigenvalue range [0.0001271656,193.3044].
## Model rank =  68 / 68
##
## Basis dimension (k) checking results. Low p-value (k-index<1) may
## indicate that k is too low, especially if edf is close to k'.
##
##                         k'      edf k-index p-value
## s(lat,lon)        49.00000 12.82359    0.74  <2e-16 ***
## s(num.records)     9.00000  8.84416    0.48  <2e-16 ***
## s(chlorophyll)     9.00000  0.00143    0.79    0.05 *
## ---
## Signif. codes:  0 '***' 0.001 '**' 0.01 '*' 0.05 '.' 0.1 ' ' 1
```

```
summary(shallow.numsp.chlorophyll)
```

```
##
## Family: Negative Binomial(0.73)
## Link function: log
##
## Formula:
## num.species ~ s(lat, lon, bs = "sos") +  s(num.records) +  s(chlorophyll)
##
## Parametric coefficients:
##                  Estimate Std. Error z value Pr(>|z|)
## (Intercept)      2.6640     0.0474     56.2    <2e-16 ***
## ---
## Signif. codes:  0 '***' 0.001 '**' 0.01 '*' 0.05 '.' 0.1 ' ' 1
##
## Approximate significance of smooth terms:
##                         edf  Ref.df     Chi.sq   p-value
## s(lat,lon)        12.823594       49     66.641  1.86e-12 ***
## s(num.records)     8.844155        9   1474.164   < 2e-16 ***
## s(chlorophyll)     0.001435        9      0.001     0.375
## ---
## Signif. codes:  0 '***' 0.001 '**' 0.01 '*' 0.05 '.' 0.1 ' ' 1
##
## R-sq.(adj) =   -1.26     Deviance explained = 74.5%
## -REML = 2690.8  Scale est. = 1              n = 705
```

```
shallow.numsp.current <- gam(num.species ~ s(lat, lon, bs = "sos") + s(num.records)
+  s(current), data = shallow, family = "nb", method = "REML", select = TRUE)
gam.check(shallow.numsp.current)
```

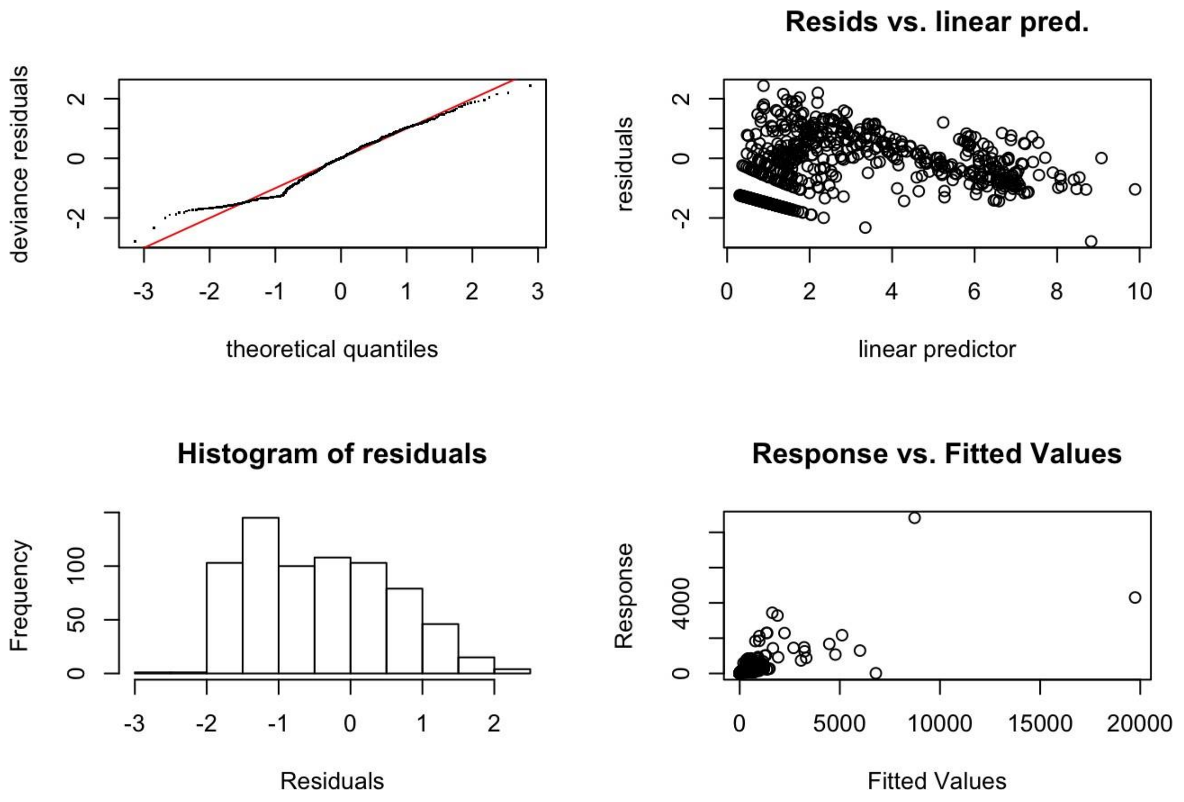

```
##
## Method: REML   Optimizer: outer newton
## full convergence after 12 iterations.
## Gradient range [-0.0002338866,0.001733544]
## (score 2690.815 & scale 1).
## Hessian positive definite, eigenvalue range [0.0001043007,193.3042].
## Model rank =  68 / 68
##
## Basis dimension (k) checking results. Low p-value (k-index<1) may
## indicate that k is too low, especially if edf is close to k'.
##
##                         k'      edf k-index p-value
## s(lat,lon)        49.00000 12.82367    0.74  <2e-16 ***
## s(num.records)     9.00000  8.84415    0.48  <2e-16 ***
## s(current)         9.00000  0.00189    0.88    0.82
## ---
## Signif. codes:  0 '***' 0.001 '**' 0.01 '*' 0.05 '.' 0.1 ' ' 1
```

```
summary(shallow.numsp.current)
```

```
##
## Family: Negative Binomial(0.73)
## Link function: log
##
## Formula:
## num.species ~ s(lat, lon, bs = "sos") + s(num.records) + s(current)
##
## Parametric coefficients:
##             Estimate Std. Error z value Pr(>|z|)
## (Intercept)   2.6640     0.0474    56.2   <2e-16 ***
## ---
## Signif. codes:  0 '***' 0.001 '**' 0.01 '*' 0.05 '.' 0.1 ' ' 1
##
## Approximate significance of smooth terms:
##                      edf Ref.df   Chi.sq  p-value
## s(lat,lon)     12.823667     49   66.655 1.86e-12 ***
## s(num.records)  8.844152      9 1474.107  < 2e-16 ***
## s(current)      0.001885      9    0.001    0.517
## ---
## Signif. codes:  0 '***' 0.001 '**' 0.01 '*' 0.05 '.' 0.1 ' ' 1
##
## R-sq.(adj) =  -1.26   Deviance explained = 74.5%
## -REML = 2690.8  Scale est. = 1         n = 705
```

```
shallow.numsp.o2.saturate <- gam(num.species ~ s(lat, lon, bs = "sos") + s(num.reco rds)
+  s(o2.saturate), data = shallow, family = "nb", method = "REML", select = TRUE)
gam.check(shallow.numsp.o2.saturate)
```

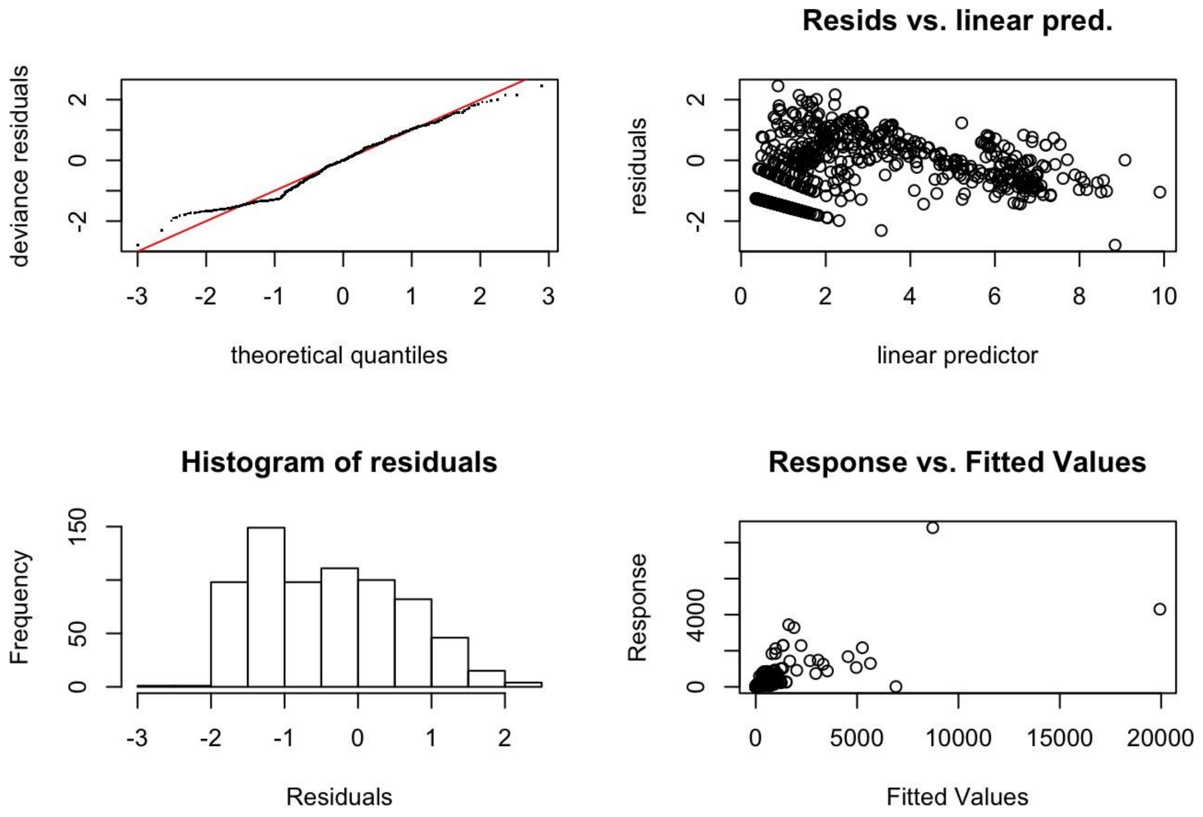


```
##
## Method: REML   Optimizer: outer newton
## full convergence after 12 iterations.
## Gradient range [-0.0001888787,0.001738329]
## (score 2690.688 & scale 1).
## Hessian positive definite, eigenvalue range [0.0001887718,193.1974].
## Model rank =  68 / 68
##
## Basis dimension (k) checking results. Low p-value (k-index<1) may
## indicate that k is too low, especially if edf is close to k'.
##
##                     k'    edf k-index p-value
## s(lat,lon)      49.000 12.358    0.74  <2e-16 ***
## s(num.records)   9.000  8.844    0.48  <2e-16 ***
## s(o2.saturate)   9.000  0.617    0.86    0.65
## ---
## Signif. codes:  0 '***' 0.001 '**' 0.01 '*' 0.05 '.' 0.1 ' ' 1
```

```
summary(shallow.numsp.o2.saturate)
```

```
##
## Family: Negative Binomial(0.73)
## Link function: log
##
## Formula:
## num.species ~ s(lat, lon, bs = "sos") + s(num.records) + s(o2.saturate)
##
## Parametric coefficients:
##             Estimate Std. Error z value Pr(>|z|)
## (Intercept)  2.66391    0.04739   56.21   <2e-16 ***
## ---
## Signif. codes:  0 '***' 0.001 '**' 0.01 '*' 0.05 '.' 0.1 ' ' 1
##
## Approximate significance of smooth terms:
##                    edf Ref.df   Chi.sq p-value
## s(lat,lon)     12.3585     49   63.474 3.3e-12 ***
## s(num.records)  8.8438      9 1480.231 < 2e-16 ***
## s(o2.saturate)  0.6173      9    1.012    0.15
## ---
## Signif. codes:  0 '***' 0.001 '**' 0.01 '*' 0.05 '.' 0.1 ' ' 1
##
## R-sq.(adj) =  -1.31   Deviance explained = 74.5%
## -REML = 2690.7  Scale est. = 1         n = 705
```

```
shallow.numsp.salinity <- gam(num.species ~ s(lat, lon, bs = "sos") + s(num.record
s) +  s(salinity), data = shallow, family = "nb", method = "REML", select = TRUE)
gam.check(shallow.numsp.salinity)
```

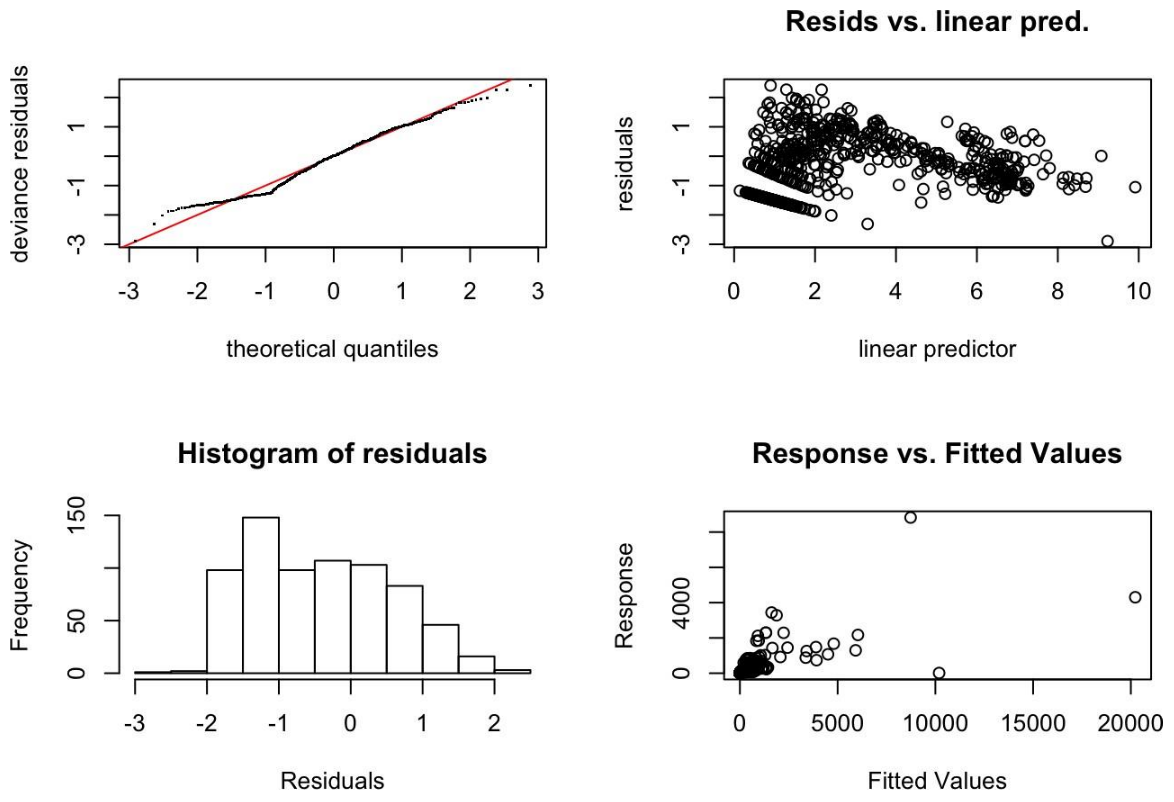

```
##
## Method: REML   Optimizer: outer newton
## full convergence after 12 iterations.
## Gradient range [-0.0001653638,0.001587915]
## (score 2689.424 & scale 1).
## Hessian positive definite, eigenvalue range [0.002622196,193.1189].
## Model rank =  68 / 68
##
## Basis dimension (k) checking results. Low p-value (k-index<1) may
## indicate that k is too low, especially if edf is close to k'.
##
##                    k'   edf k-index p-value
## s(lat,lon)      49.00 10.73    0.75  <2e-16 ***
## s(num.records)   9.00  8.84    0.48  <2e-16 ***
## s(salinity)      9.00  1.72    0.82     0.1
## ---
## Signif. codes:  0 '***' 0.001 '**' 0.01 '*' 0.05 '.' 0.1 ' ' 1
```

```
summary(shallow.numsp.salinity)
```

```
##
## Family: Negative Binomial(0.732)
## Link function: log
##
## Formula:
## num.species ~ s(lat, lon, bs = "sos") + s(num.records) + s(salinity)
##
## Parametric coefficients:
##             Estimate Std. Error z value Pr(>|z|)
## (Intercept)  2.66279    0.04735   56.23   <2e-16 ***
## ---
## Signif. codes:  0 '***' 0.001 '**' 0.01 '*' 0.05 '.' 0.1 ' ' 1
##
## Approximate significance of smooth terms:
##                   edf Ref.df   Chi.sq  p-value
## s(lat,lon)     10.728     49   28.168 0.000165 ***
## s(num.records)  8.843      9 1493.268  < 2e-16 ***
## s(salinity)     1.716      9    6.143 0.004691 **
## ---
## Signif. codes:  0 '***' 0.001 '**' 0.01 '*' 0.05 '.' 0.1 ' ' 1
##
## R-sq.(adj) =  -1.76   Deviance explained = 74.5%
## -REML = 2689.4  Scale est. = 1         n = 705
```

```
shallow.numsp.nitrate <- gam(num.species ~ s(lat, lon, bs = "sos") + s(num.records)
+  s(nitrate), data = shallow, family = "nb", method = "REML", select = TRUE)
gam.check(shallow.numsp.nitrate)
```

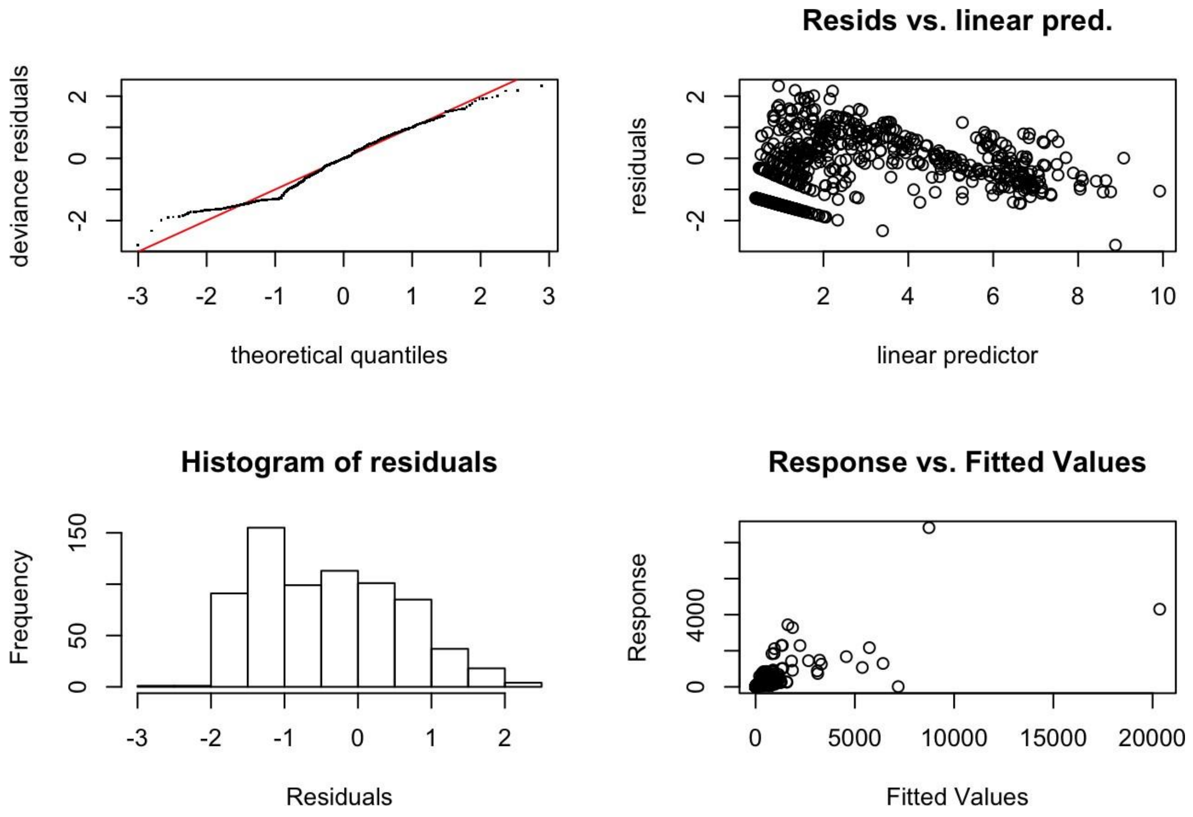


```
##
## Method: REML   Optimizer: outer newton
## full convergence after 12 iterations.
## Gradient range [-0.0001123949,0.001608861]
## (score 2689.498 & scale 1).
## Hessian positive definite, eigenvalue range [0.0001123446,195.7503].
## Model rank =  68 / 68
##
## Basis dimension (k) checking results. Low p-value (k-index<1) may
## indicate that k is too low, especially if edf is close to k'.
##
##                   k'  edf k-index p-value
## s(lat,lon)     49.00 8.68    0.74  <2e-16 ***
## s(num.records)  9.00 8.84    0.48  <2e-16 ***
## s(nitrate)      9.00 1.44    0.81    0.08 .
## ---
## Signif. codes:  0 '***' 0.001 '**' 0.01 '*' 0.05 '.' 0.1 ' ' 1
```

```
summary(shallow.numsp.nitrate)
```

```
##
## Family: Negative Binomial(0.725)
## Link function: log
##
## Formula:
## num.species ~ s(lat, lon, bs = "sos") + s(num.records) + s(nitrate)
##
## Parametric coefficients:
##              Estimate Std. Error z value Pr(>|z|)
## (Intercept)   2.67060    0.04751   56.21   <2e-16 ***
## ---
## Signif. codes:  0 '***' 0.001 '**' 0.01 '*' 0.05 '.' 0.1 ' ' 1
##
## Approximate significance of smooth terms:
##                  edf Ref.df    Chi.sq  p-value
## s(lat,lon)     8.683     49   42.889 3.87e-09 ***
## s(num.records) 8.844      9 1554.037  < 2e-16 ***
## s(nitrate)     1.442      9    6.317   0.0026 **
## ---
## Signif. codes:  0 '***' 0.001 '**' 0.01 '*' 0.05 '.' 0.1 ' ' 1
##
## R-sq.(adj) =  -1.48   Deviance explained = 74.2%
## -REML = 2689.5  Scale est. = 1         n = 705
```

```
shallow.numsp.env <- gam(num.species ~ s(lat, lon, bs = "sos") + s(num.records) + s
(temp) + s(dissolved.oxygen) + s(primary.productivity) + s(chlorophyll) + s(curren
t) + s(o2.saturate) + s(salinity) + s(nitrate), data = shallow, family = "nb", meth od =
"REML", select = TRUE)
gam.check(shallow.numsp.env)
```

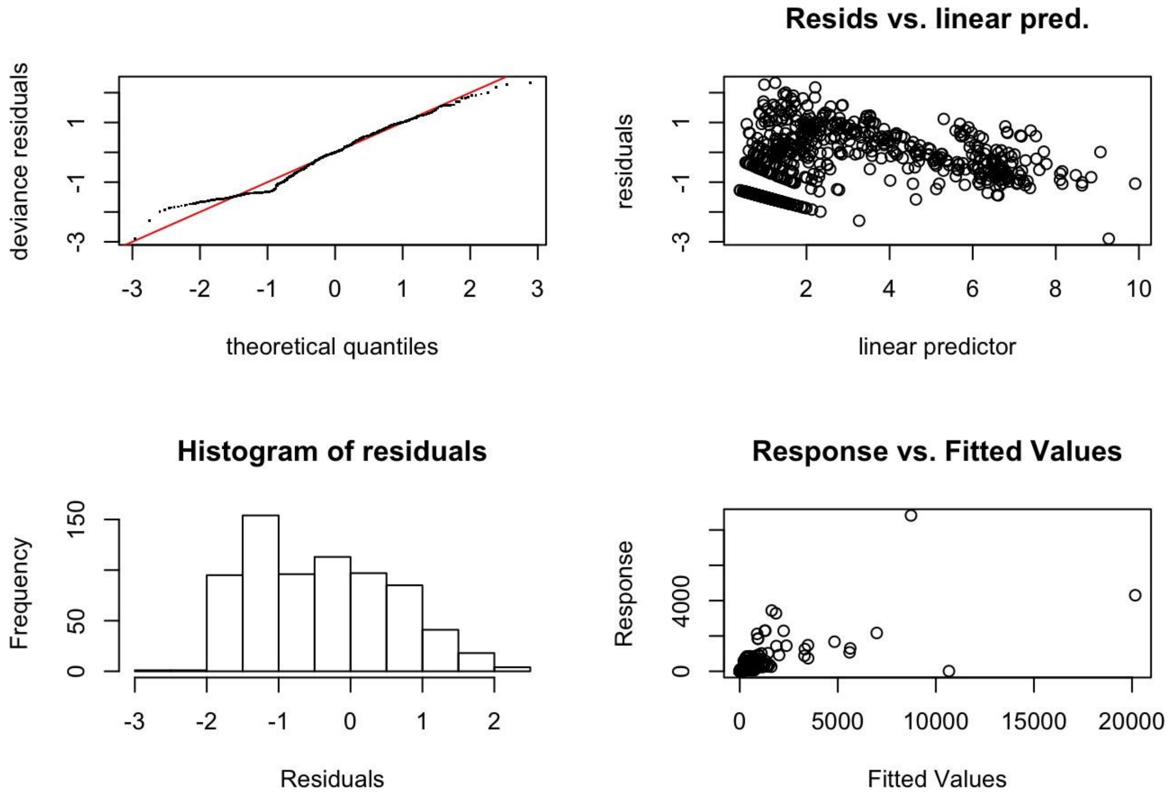

```
##
## Method: REML   Optimizer: outer newton
## full convergence after 19 iterations.
## Gradient range [-0.0002419147,0.0007436145]
## (score 2687.494 & scale 1).
## eigenvalue range [-0.0001766961,195.6325].
## Model rank =  131 / 131
##
## Basis dimension (k) checking results. Low p-value (k-index<1) may
## indicate that k is too low, especially if edf is close to k'.
##
##                                  k'      edf k-index p-value
## s(lat,lon)                4.90e+01 5.38e+00    0.74  <2e-16 ***
## s(num.records)            9.00e+00 8.84e+00    0.48  <2e-16 ***
## s(temp)                   9.00e+00 2.09e-03    0.89   0.850
## s(dissolved.oxygen)       9.00e+00 6.69e-01    0.84   0.395
## s(primary.productivity) 9.00e+00 1.59e-03    0.83   0.315
## s(chlorophyll)            9.00e+00 2.01e-03    0.80   0.050 *
## s(current)                9.00e+00 8.63e-04    0.88   0.790
## s(o2.saturate)            9.00e+00 8.60e-01    0.86   0.650
## s(salinity)               9.00e+00 8.51e-01    0.82   0.205
## s(nitrate)                9.00e+00 1.40e+00    0.80   0.065 .
## ---
## Signif. codes:  0 '***' 0.001 '**' 0.01 '*' 0.05 '.' 0.1 ' ' 1
```

```
summary(shallow.numsp.env)
```

```
##
## Family: Negative Binomial(0.728)
## Link function: log
##
## Formula:
## num.species ~ s(lat, lon, bs = "sos") + s(num.records) + s(temp) +
##       s(dissolved.oxygen) +  s(primary.productivity)  +  s(chlorophyll)  +
##       s(current) + s(o2.saturate) + s(salinity) +  s(nitrate)
##
## Parametric coefficients:
##                  Estimate Std. Error z value Pr(>|z|)
##  (Intercept)   2.66998     0.04741    56.32   <2e-16 ***
## ---
## Signif. codes:  0 '***' 0.001 '**' 0.01 '*' 0.05 '.' 0.1 ' ' 1
##
## Approximate significance of smooth terms:
##                                 edf  Ref.df    Chi.sq   p-value
## s(lat,lon)                 5.3823776      49    14.832  0.000545  ***
## s(num.records)             8.8397292       9  1609.506   < 2e-16  ***
## s(temp)                    0.0020898       9     0.001  0.143235
## s(dissolved.oxygen)        0.6685280       9     0.942  0.069083  .
## s(primary.productivity) 0.0015915         9     0.001  0.350595
## s(chlorophyll)             0.0020123       9     0.003  0.203619
## s(current)                 0.0008629       9     0.000  0.704874
## s(o2.saturate)             0.8599729       9     1.733  0.107133
## s(salinity)                0.8513896       9     5.520  0.002008  **
## s(nitrate)                 1.4007490       9     5.087  0.007049  **
## ---
## Signif. codes:  0 '***' 0.001 '**' 0.01 '*' 0.05 '.' 0.1 ' ' 1
##
##  R-sq.(adj) =   -1.85     Deviance explained = 74.3%
## -REML = 2687.5Scale  est.  =  1               n = 705
```

Model selection for number of species, shallow water

Here we use AIC to compare models for goodness of fit while penalizing for overparameterization. Models are ranked in order of AIC score, with lowest scoring model (model with the best compromise between fit and complexity) first in the list. The delta AIC represents how much the AIC score for each model differs from the top model, an can be used as an estimate of the relative support for each model. A delta AIC of 2 is considered to be potentially a significantly better fit, with higher delta AICs between models indicating increasingly larger differences in model fit while correcting for the number of parameters.

```
shallow.numsp.models <- list(shallow.numsp.intercept = shallow.numsp.intercept,
                              shallow.numsp.numrec = shallow.numsp.numrec,
                         shallow.numsp.latlon = shallow.numsp.latlon,
                         shallow.numsp.temp = shallow.numsp.temp,
                         shallow.numsp.oxygen = shallow.numsp.oxygen,
                         shallow.numsp.prod = shallow.numsp.prod,
                         shallow.numsp.chlorophyll = shallow.numsp.chlorophyll,
                         shallow.numsp.current = shallow.numsp.current,
                         shallow.numsp.o2.saturate = shallow.numsp.o2.saturate,
                         shallow.numsp.salinity = shallow.numsp.salinity,
                         shallow.numsp.nitrate = shallow.numsp.nitrate,
                         shallow.numsp.env = shallow.numsp.env)
shallow.numsp.aic.df <- data.frame(Model = names(shallow.numsp.models),
                              AIC = sapply(shallow.numsp.models, function(x) x$aic),
                             akaike.weights(sapply(shallow.numsp.models, function(x)
x$aic)))

shallow.numsp.aic.df <- shallow.numsp.aic.df[order(shallow.numsp.aic.df$AIC),]
shallow.numsp.aic.df$Cumulative.Weight <- cumsum(shallow.numsp.aic.df$weights)

kable(shallow.numsp.aic.df, row.names = FALSE)
```

Model	AIC	deltaAIC	rel.LL	weights	Cumulative.Weight
shallow.numsp.salinity	5318.205	0.0000000	1.0000000	0.2104098	0.2104098
shallow.numsp.env	5318.527	0.3222579	0.8511823	0.1790971	0.3895069
shallow.numsp.temp	5319.780	1.5747971	0.4550270	0.0957421	0.4852491
shallow.numsp.latlon	5320.046	1.8404896	0.3984215	0.0838318	0.5690808
shallow.numsp.prod	5320.047	1.8412992	0.3982602	0.0837979	0.6528787
shallow.numsp.chlorophyll	5320.047	1.8414643	0.3982274	0.0837909	0.7366696
shallow.numsp.current	5320.048	1.8427285	0.3979757	0.0837380	0.8204076
shallow.numsp.o2.saturate	5320.201	1.9957224	0.3686671	0.0775712	0.8979788
shallow.numsp.oxygen	5320.817	2.6119011	0.2709149	0.0570032	0.9549820
shallow.numsp.nitrate	5321.289	3.0839880	0.2139540	0.0450180	1.0000000
shallow.numsp.numrec	5374.309	56.1033173	0.0000000	0.0000000	1.0000000
shallow.numsp.intercept	6231.247	913.0422064	0.0000000	0.0000000	1.0000000

```
write.csv(shallow.numsp.aic.df, file = "shallow.numsp.aic.csv")
```

Here we see that the model containing salinity is the best fit, but many other models are almost as good. It is particularly noteworthy that the model containing only the effects of spatial autocorrelation (the “latlon” model) has a delta AIC of < 2 compared to the best-performing model. However, we know that latitude is highly correlated with several of the environmental predictors. That being the case, it is hard to determine which, if any, environmental predictors are affecting the number of species in shallow water. T

Plots for number of species, shallow water

Here we plot the effects of each environmental predictor from its respective single-variable model.

```
qplot(shallow$temp, predict(shallow.numsp.temp, shallow)) + geom_smooth(method = "g
am")
```

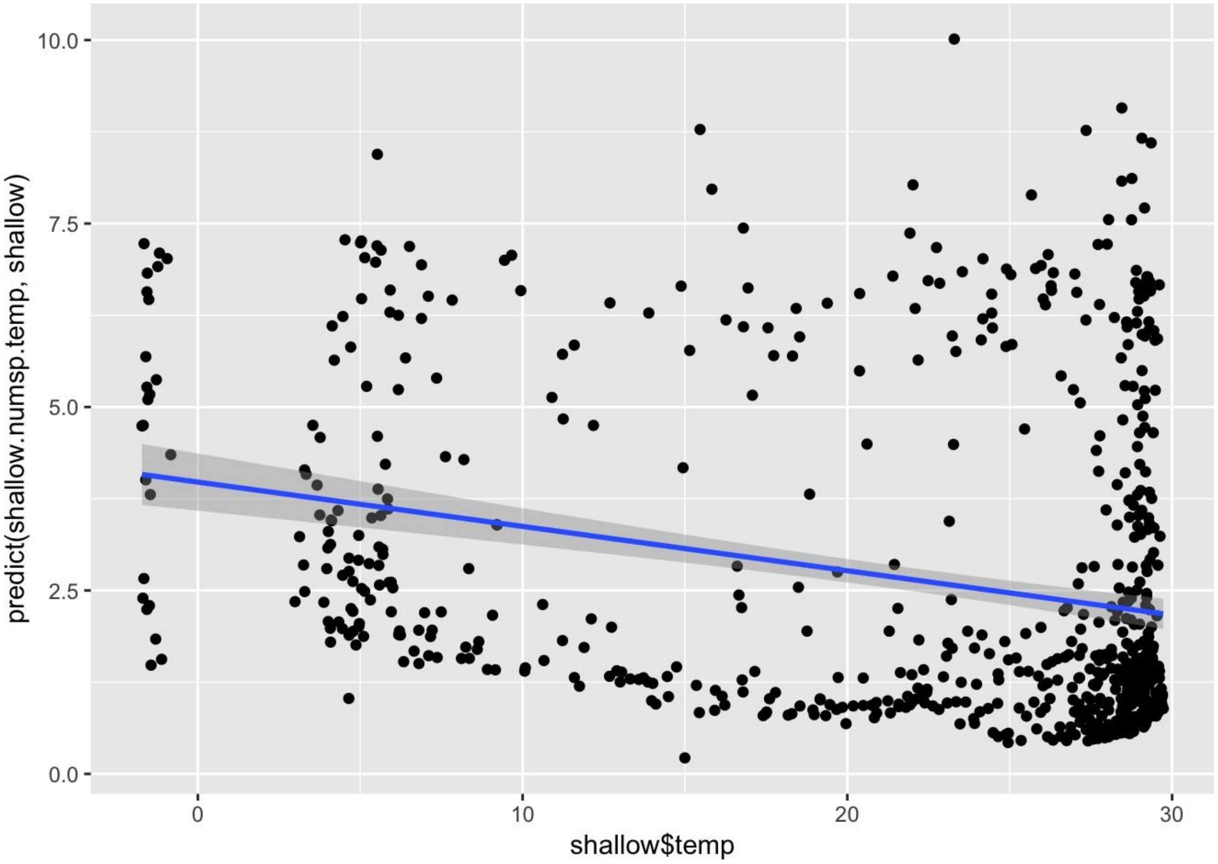


```
qplot(shallow$primary.productivity, predict(shallow.numsp.prod, shallow)) + geom_sm
ooth(method = "gam")
```

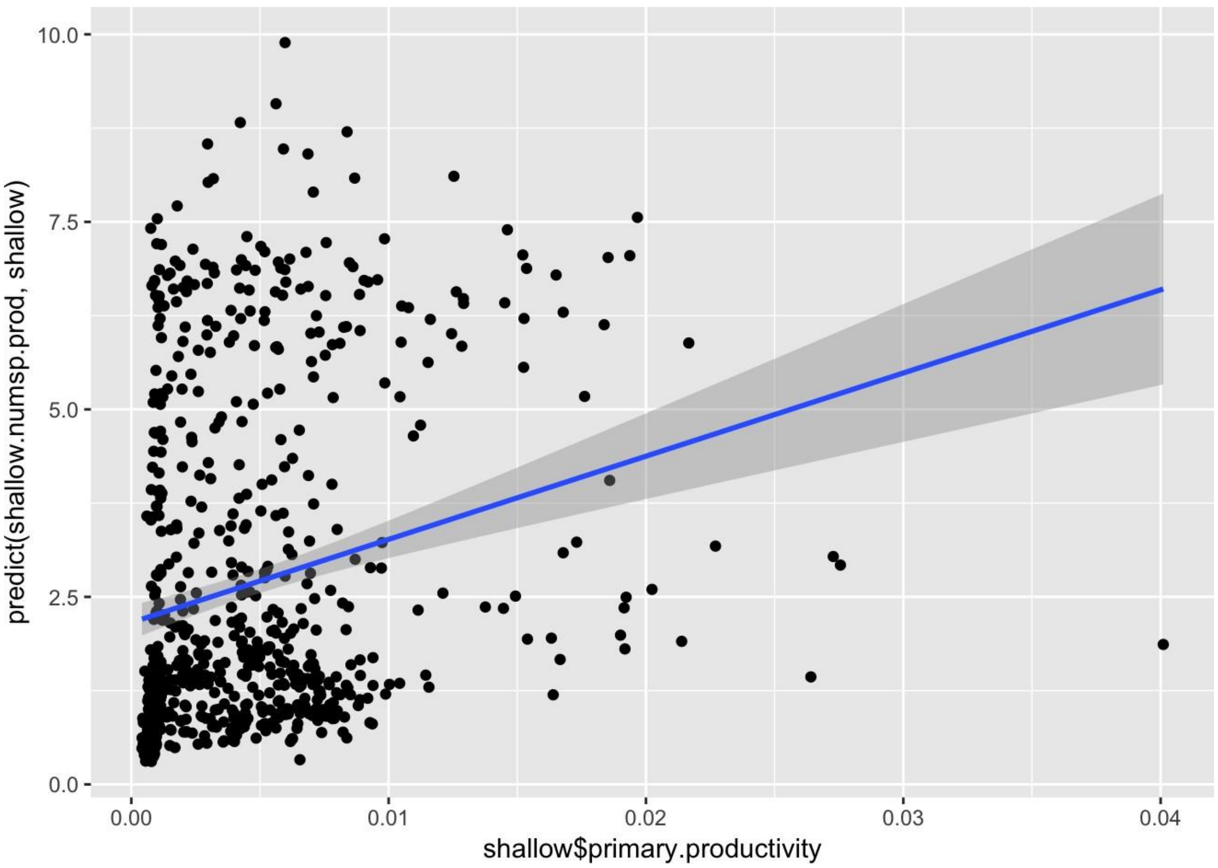


```
qplot(shallow$dissolved.oxygen, predict(shallow.numsp.oxygen, shallow)) + geom_smoo
th(method = "gam")
```

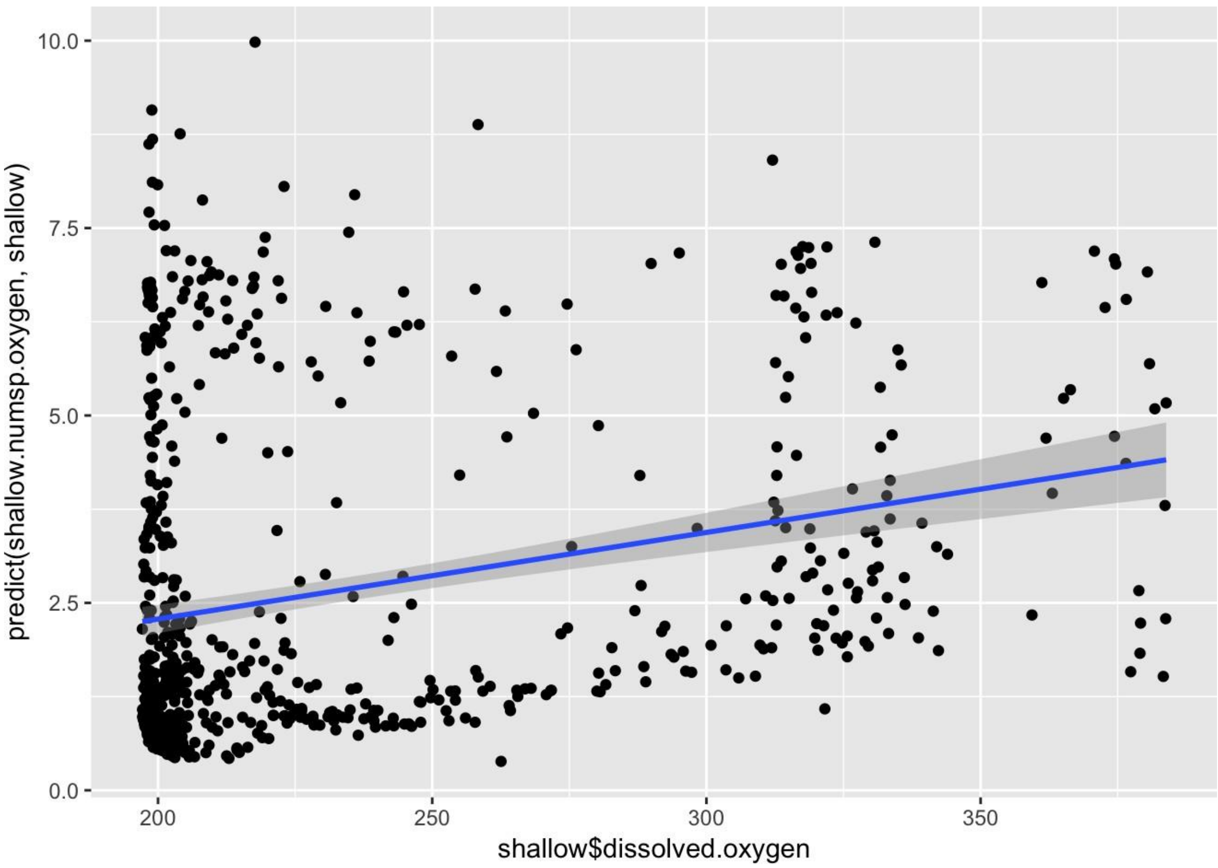


```
qplot(shallow$chlorophyll, predict(shallow.numsp.chlorophyll, shallow)) + geom_smoo
th(method = "gam")
```

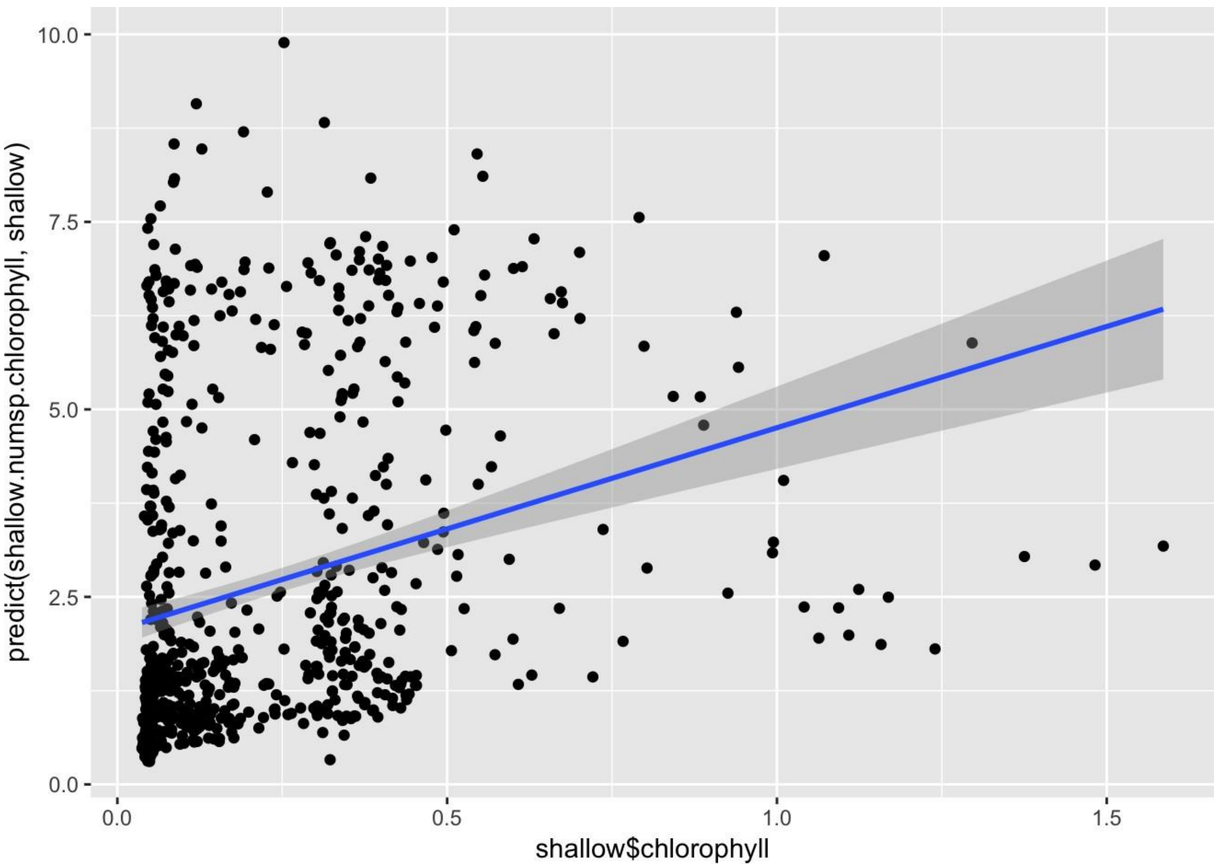


```
qplot(shallow$current, predict(shallow.numsp.current, shallow)) + geom_smooth(metho
d = "gam")
```

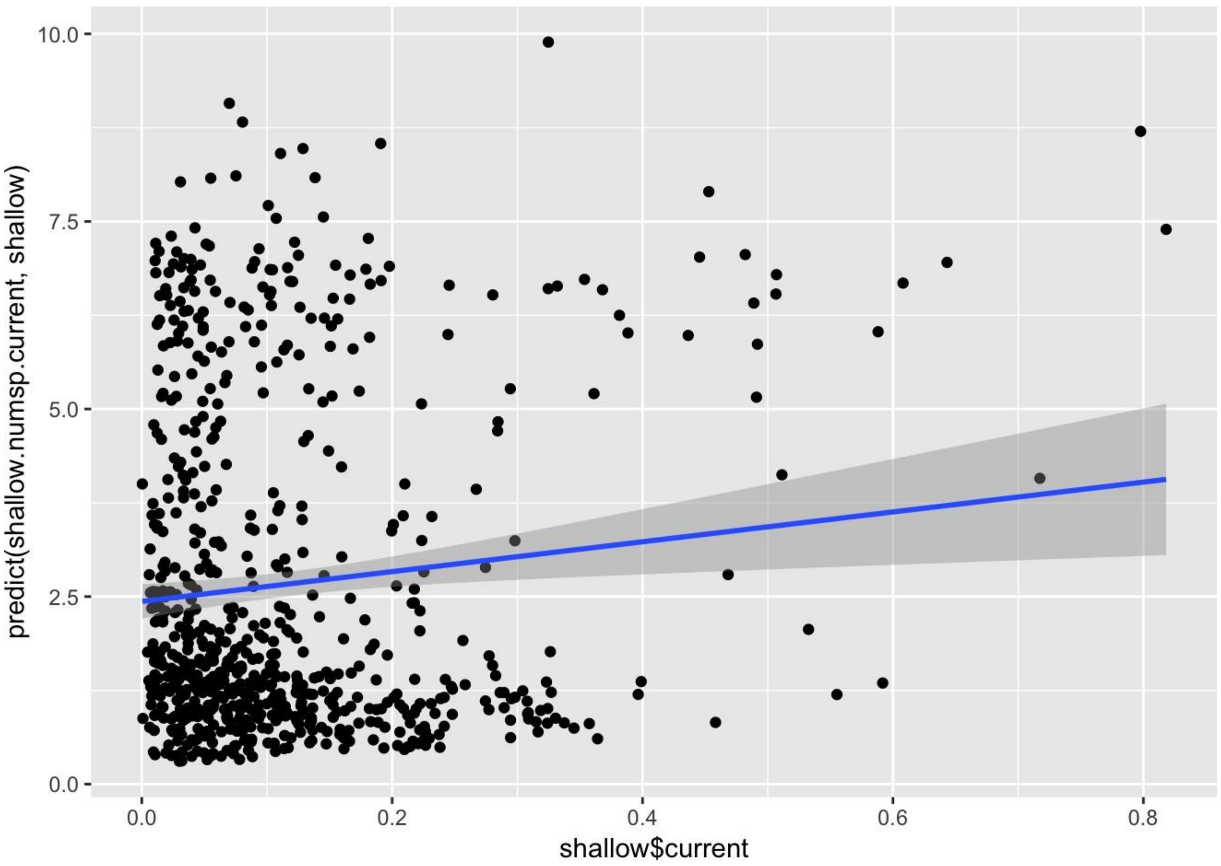


```
qplot(shallow$o2.saturate, predict(shallow.numsp.o2.saturate, shallow)) + geom_smoo
th(method = "gam")
```

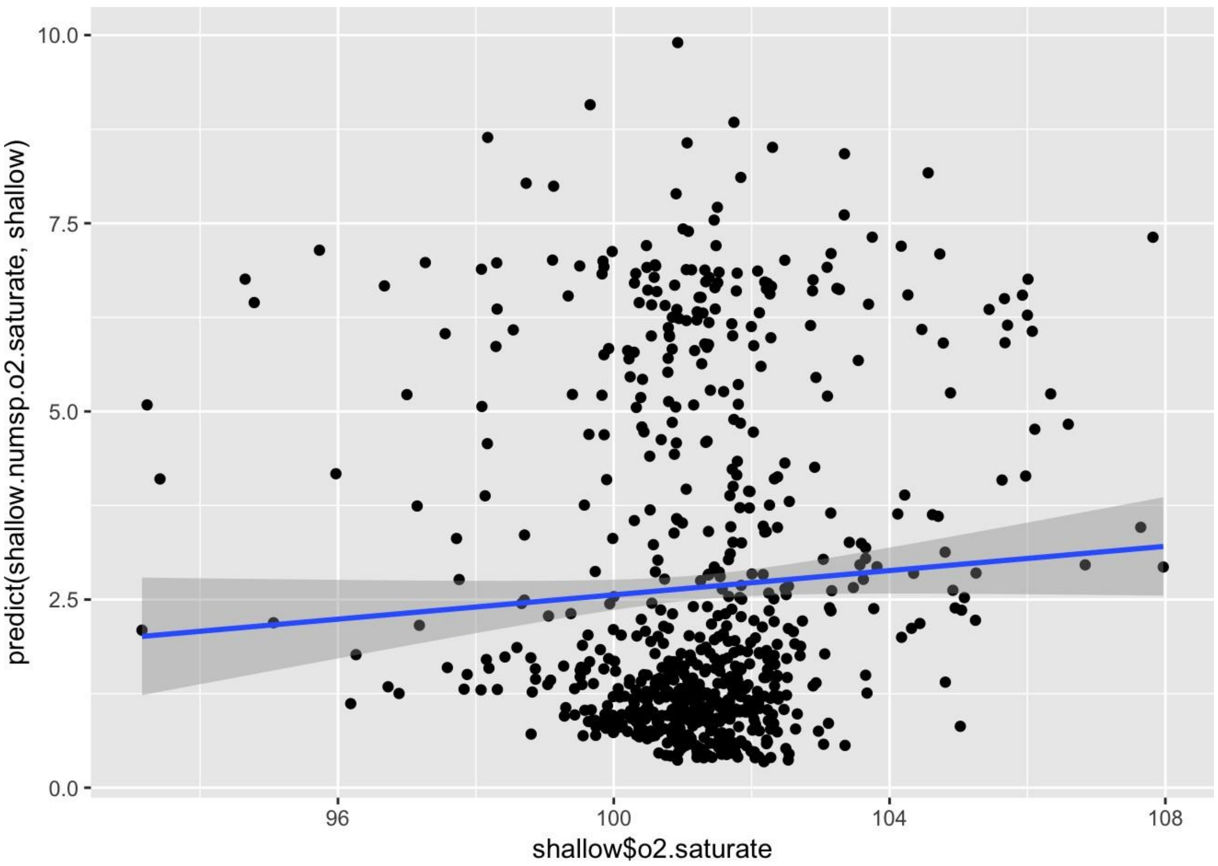


```
qplot(shallow$salinity, predict(shallow.numsp.salinity, shallow)) + geom_smooth(met
hod = "gam")
```

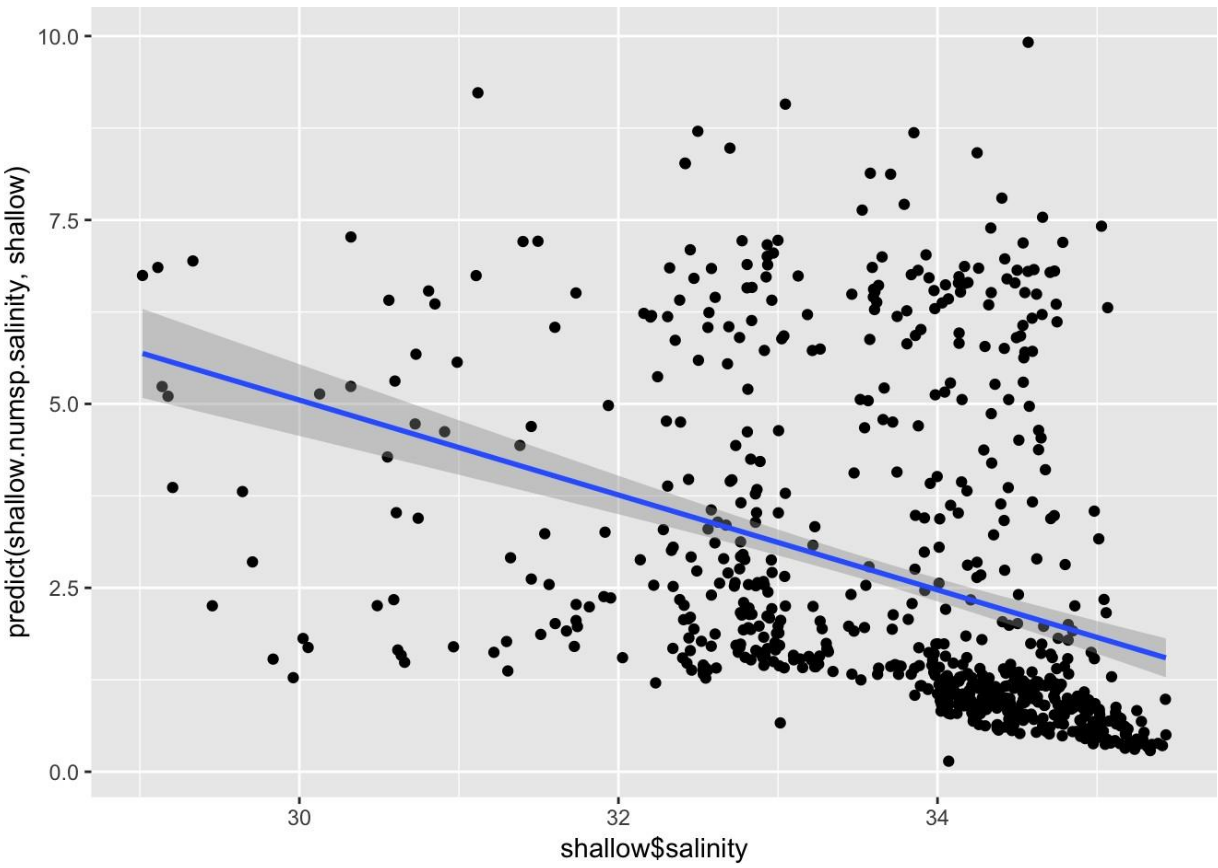


```
qplot(shallow$nitrate, predict(shallow.numsp.nitrate, shallow)) + geom_smooth(metho
d = "gam")
```

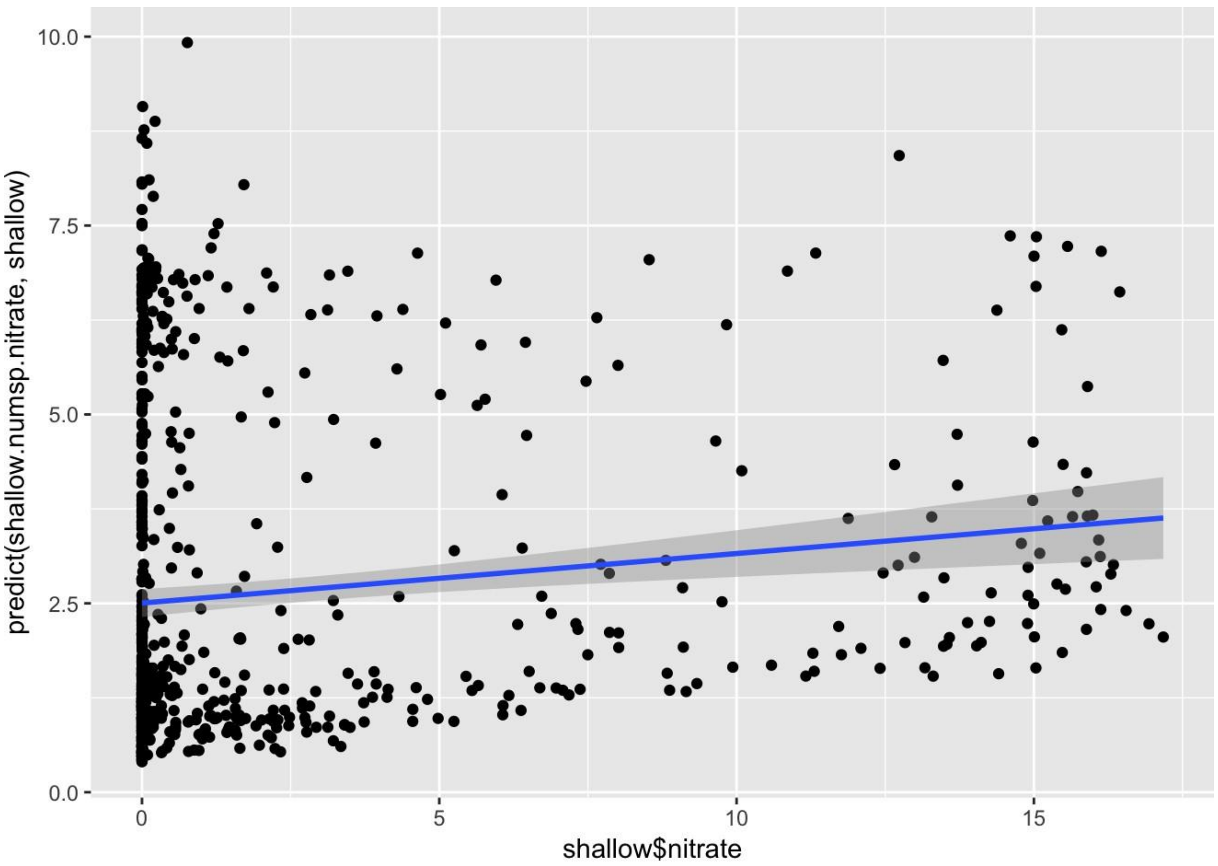

GAMs for ES50, shallow water

These models are identical to the above, except that the response variable is the species richness estimated from the rarefaction curves. These are meant to be estimates of species richness if all sites were sampled equally. Since these measurements are intended to take sampling effort into account, we exclude the “no.records” term for these models.

```
shallow.es50.intercept <- gam(ES50 ~ 1, data = shallow, family = "nb", method = "RE
ML", select = TRUE)
gam.check(shallow.es50.intercept)
```

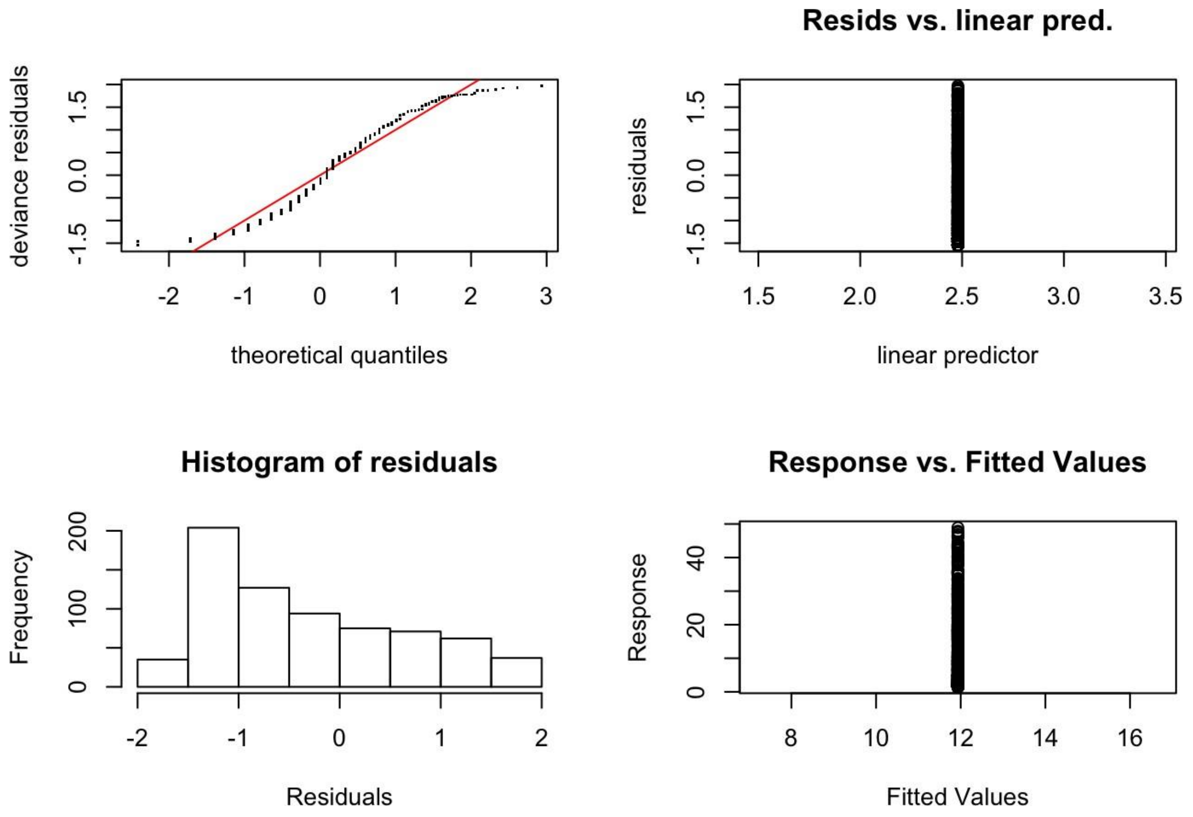


```
##
## Method: REML   Optimizer: outer newton
## full convergence after 3 iterations.
## Gradient range [5.595699e-09,5.595699e-09]
## (score 2476.994 & scale 1).
## Hessian positive definite, eigenvalue range [335.1861,335.1861].
## Model rank =  1 / 1
```

```
summary(shallow.es50.intercept)
```

```
##
## Family: Negative Binomial(1.231)
## Link function: log
##
## Formula:
## ES50 ~ 1
##
## Parametric coefficients:
##             Estimate Std. Error z value Pr(>|z|)
## (Intercept)  2.47939    0.03566   69.53   <2e-16 ***
## ---
## Signif. codes:  0 '***' 0.001 '**' 0.01 '*' 0.05 '.' 0.1 ' ' 1
##
##
## R-sq.(adj) =      0   Deviance explained = 3.56e-08%
## -REML =   2477  Scale est. = 1         n = 705
```

```
shallow.es50.latlon <- gam(ES50 ~ s(lat, lon, bs = "sos") , data = shallow, family
 = "nb", method = "REML", select = TRUE)
gam.check(shallow.es50.latlon)
```

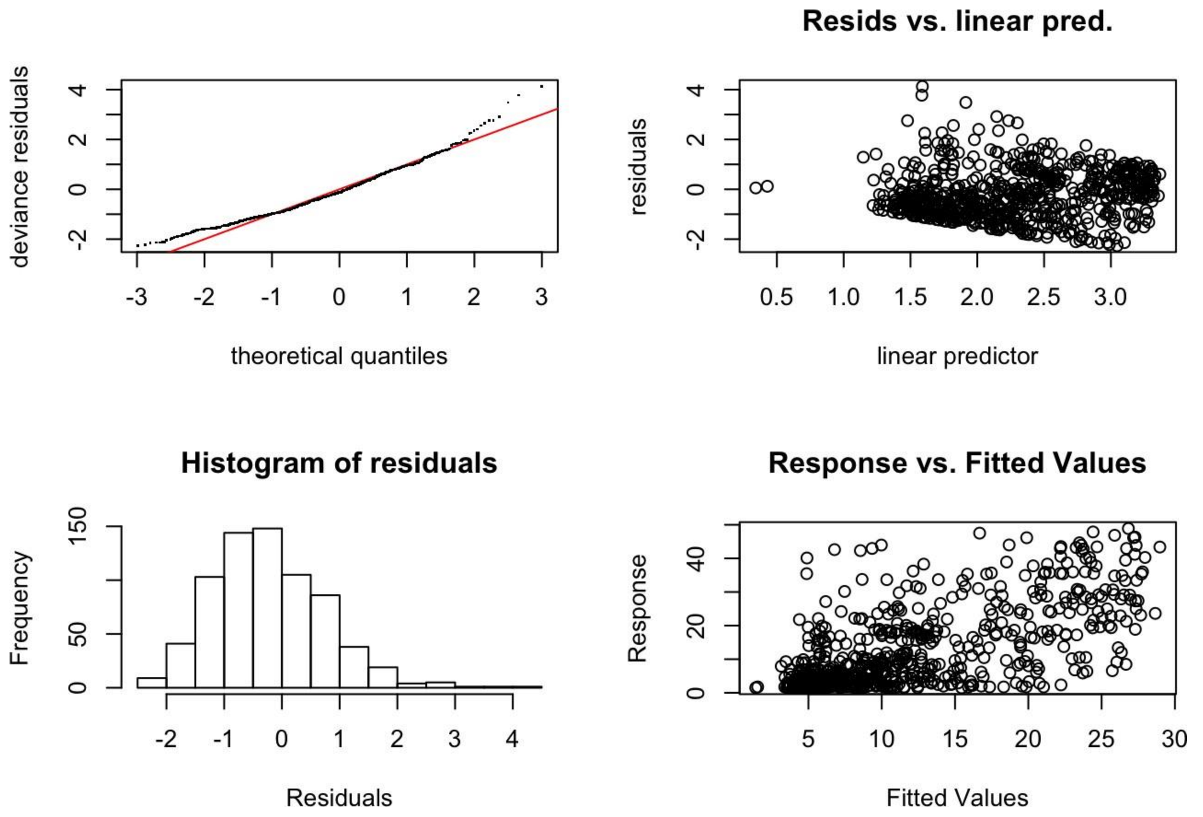


```
##
## Method: REML   Optimizer: outer newton
## full convergence after 3 iterations.
## Gradient range [-1.636573e-06,1.452781e-05]
## (score 2341.56 & scale 1).
## Hessian positive definite, eigenvalue range [9.722589,234.1502].
## Model rank =  50 / 50
##
## Basis dimension (k) checking results. Low p-value (k-index<1) may
## indicate that k is too low, especially if edf is close to k'.
##
##                k'  edf k-index p-value
## s(lat,lon) 49.0 34.2    0.84  <2e-16 ***
## ---
## Signif. codes:  0 '***' 0.001 '**' 0.01 '*' 0.05 '.' 0.1 ' ' 1
```

```
summary(shallow.es50.latlon)
```

```
##
## Family: Negative Binomial(2.106)
## Link function: log
##
## Formula:
## ES50 ~ s(lat, lon, bs = "sos")
##
## Parametric coefficients:
##              Estimate Std. Error z value Pr(>|z|)
## (Intercept)   2.28253    0.02904   78.59   <2e-16 ***
## ---
## Signif. codes:  0 '***' 0.001 '**' 0.01 '*' 0.05 '.' 0.1 ' ' 1
##
## Approximate significance of smooth terms:
##               edf Ref.df Chi.sq p-value
## s(lat,lon) 34.23     49  436.6  <2e-16 ***
## ---
## Signif. codes:  0 '***' 0.001 '**' 0.01 '*' 0.05 '.' 0.1 ' ' 1
##
## R-sq.(adj) =  0.415   Deviance explained =   42%
## -REML = 2341.6  Scale est. = 1         n = 705
```

```
shallow.es50.temp <- gam(ES50 ~ s(lat, lon, bs = "sos") + s(temp), data = shallow,
family = "nb", method = "REML", select = TRUE)
gam.check(shallow.es50.temp)
```

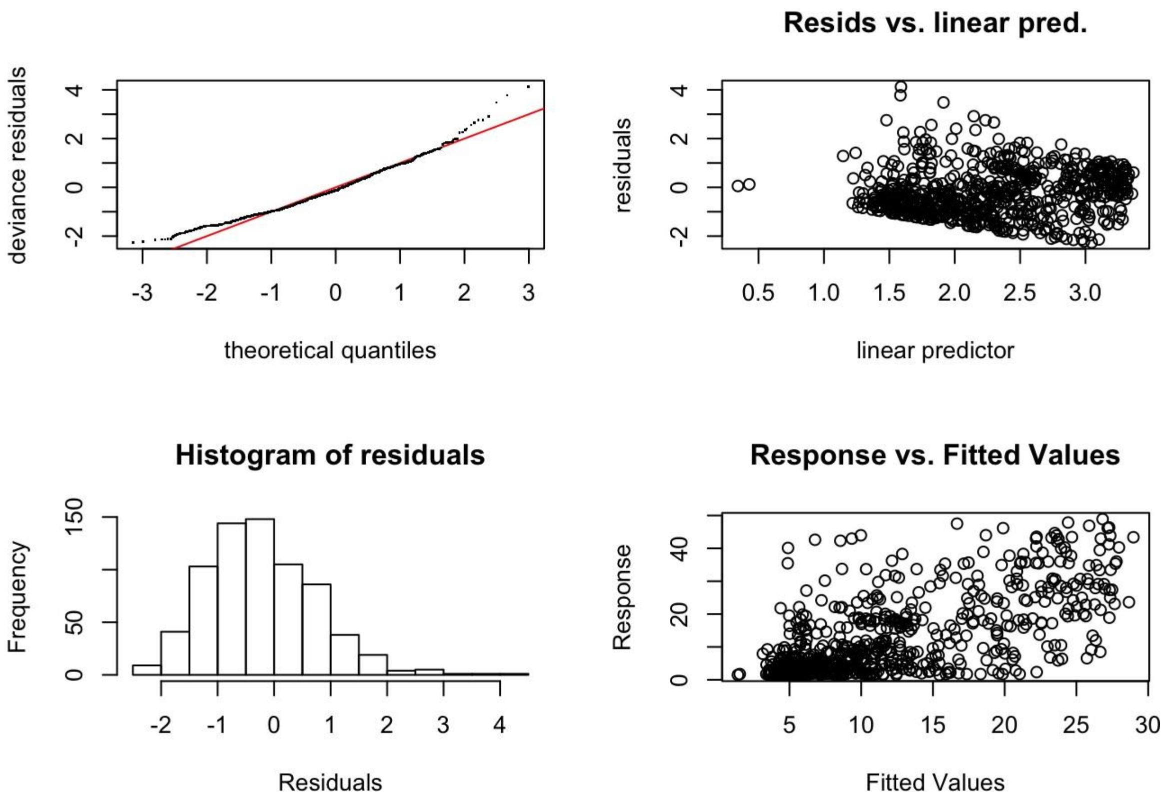

```
##
## Method: REML   Optimizer: outer newton
## full convergence after 6 iterations.
## Gradient range [-0.0007373449,0.0003134109]
## (score 2341.561 & scale 1).
## Hessian positive definite, eigenvalue range [1.390536e-05,234.1496].
## Model rank =  59 / 59
##
## Basis dimension (k) checking results. Low p-value (k-index<1) may
## indicate that k is too low, especially if edf is close to k'.
##
##                   k'      edf k-index p-value
## s(lat,lon)  49.00000 34.23233    0.84  <2e-16 ***
## s(temp)      9.00000  0.00244    0.97    0.74
## ---
## Signif. codes:  0 '***' 0.001 '**' 0.01 '*' 0.05 '.' 0.1 ' ' 1
```

```
summary(shallow.es50.temp)
```

```
##
## Family: Negative Binomial(2.106)
## Link function: log
##
## Formula:
## ES50 ~ s(lat, lon, bs = "sos") + s(temp)
##
## Parametric coefficients:
##             Estimate Std. Error z value Pr(>|z|)
## (Intercept)  2.28253    0.02904   78.59   <2e-16 ***
## ---
## Signif. codes:  0 '***' 0.001 '**' 0.01 '*' 0.05 '.' 0.1 ' ' 1
##
## Approximate significance of smooth terms:
##                  edf Ref.df  Chi.sq p-value
## s(lat,lon) 34.232332     49 434.804  <2e-16 ***
## s(temp)     0.002443      9   0.001   0.511
## ---
## Signif. codes:  0 '***' 0.001 '**' 0.01 '*' 0.05 '.' 0.1 ' ' 1
##
## R-sq.(adj) =  0.415   Deviance explained =   42%
## -REML = 2341.6  Scale est. = 1         n = 705
```

```
shallow.es50.oxygen <- gam(ES50 ~ s(lat, lon, bs= "sos") + s(dissolved.oxygen),
data = shallow, family = "nb", method = "REML", select = TRUE)
gam.check(shallow.es50.oxygen)
```

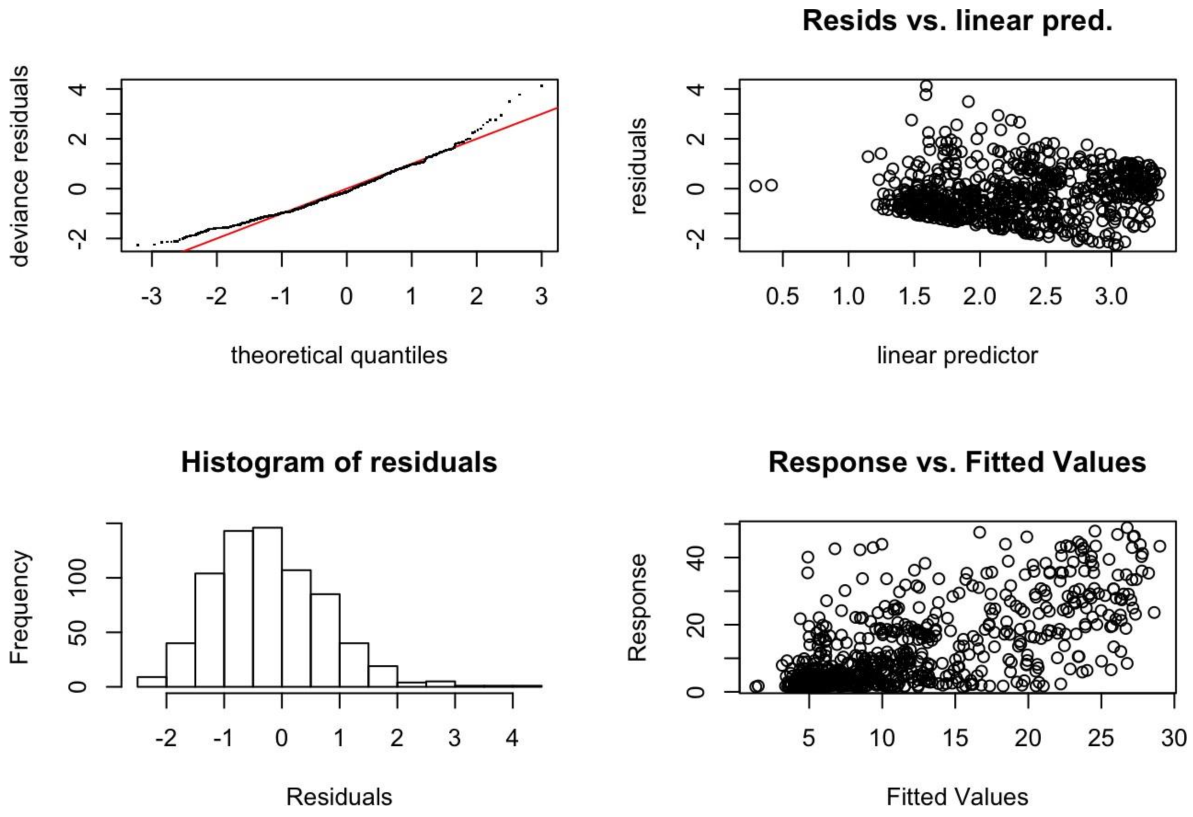


```
##
## Method: REML   Optimizer: outer newton
## full convergence after 3 iterations.
## Gradient range [-0.0002385339,0.0001233648]
## (score 2341.47 & scale 1).
## Hessian positive definite, eigenvalue range [0.0002383868,233.6167].
## Model rank =  59 / 59
##
## Basis dimension (k) checking results. Low p-value (k-index<1) may
## indicate that k is too low, especially if edf is close to k'.
##
##                         k'    edf k-index p-value
## s(lat,lon)          49.000 33.884    0.84  <2e-16 ***
## s(dissolved.oxygen)  9.000  0.809    0.95     0.5
## ---
## Signif. codes:  0 '***' 0.001 '**' 0.01 '*' 0.05 '.' 0.1 ' ' 1
```

```
summary(shallow.es50.oxygen)
```

```
##
## Family: Negative Binomial(2.109)
## Link function: log
##
## Formula:
## ES50  ~ s(lat, lon, bs = "sos") + s(dissolved.oxygen)
##
## Parametric coefficients:
##                  Estimate Std. Error z value  Pr(>|z|)
##  (Intercept)    2.28207      0.02903     78.62     <2e-16 ***
## ---
## Signif. codes:  0 '***' 0.001 '**' 0.01 '*' 0.05 '.' 0.1 ' ' 1
##
## Approximate significance of smooth terms:
##                                edf  Ref.df    Chi.sq  p-value
## s(lat,lon)                33.884        49 416.405   <2e-16 ***
## s(dissolved.oxygen)    0.809          9      1.138   0.0899 .
## ---
## Signif. codes:  0 '***' 0.001 '**' 0.01 '*' 0.05 '.' 0.1 ' ' 1
##
## R-sq.(adj) =  0.418    Deviance explained = 42.1%
## -REML = 2341.5  Scale est. = 1            n = 705
```

```
shallow.es50.prod <- gam(ES50 ~ s(lat, lon, bs = "sos") + s(primary.productivit
y), data = shallow, family = "nb", method = "REML", select = TRUE)
gam.check(shallow.es50.prod)
```

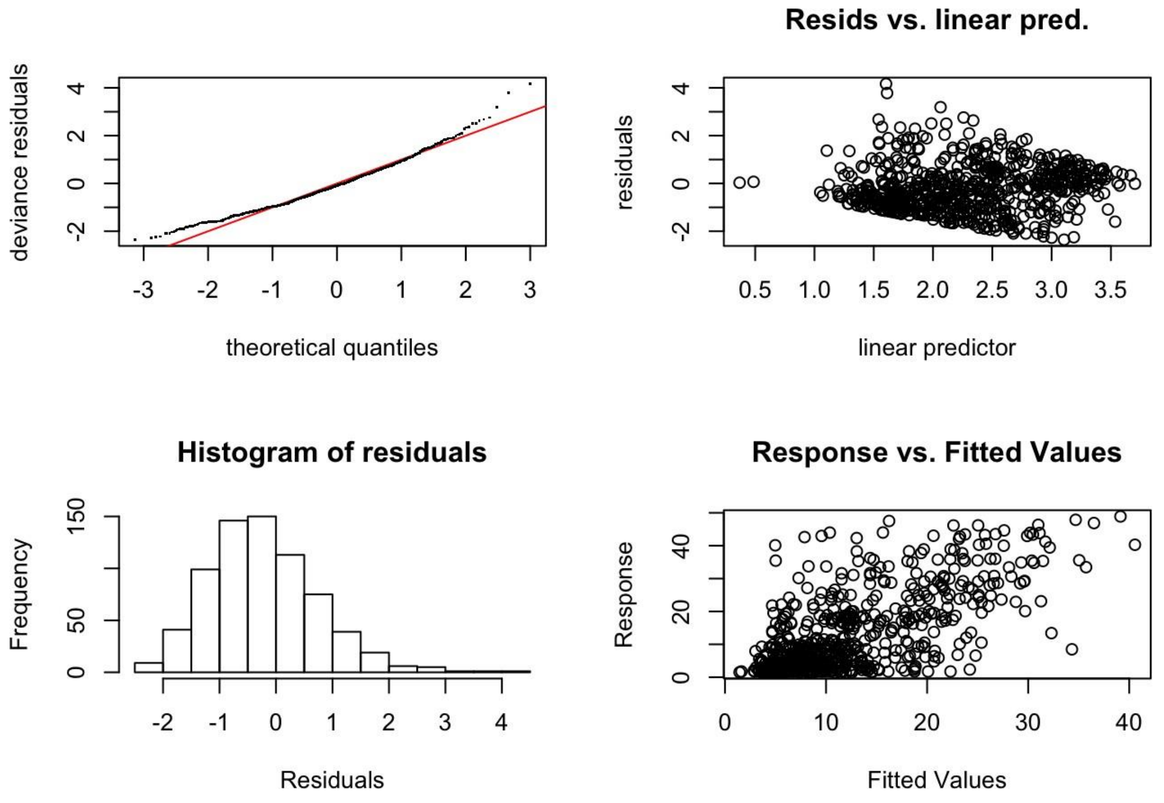

```
##
## Method: REML   Optimizer: outer newton
## full convergence after 5 iterations.
## Gradient range [-1.662754e-06,3.139754e-06]
## (score 2331.933 & scale 1).
## Hessian positive definite, eigenvalue range [0.1293296,226.2702].
## Model rank =  59 / 59
##
## Basis dimension (k) checking results. Low p-value (k-index<1) may
## indicate that k is too low, especially if edf is close to k'.
##
##                             k'   edf k-index p-value
## s(lat,lon)               49.00 34.19    0.85  <2e-16 ***
## s(primary.productivity)  9.00  3.37    0.99    0.85
## ---
## Signif. codes:  0 '***' 0.001 '**' 0.01 '*' 0.05 '.' 0.1 ' ' 1
```

```
summary(shallow.es50.prod)
```

```
##
## Family: Negative Binomial(2.209)
## Link function: log
##
## Formula:
## ES50 ~ s(lat, lon, bs = "sos") + s(primary.productivity)
##
## Parametric coefficients:
##             Estimate Std. Error z value Pr(>|z|)
## (Intercept)  2.27083    0.02856   79.52   <2e-16 ***
## ---
## Signif. codes:  0 '***' 0.001 '**' 0.01 '*' 0.05 '.' 0.1 ' ' 1
##
## Approximate significance of smooth terms:
##                            edf Ref.df Chi.sq  p-value
## s(lat,lon)               34.19     49 422.70  < 2e-16 ***
## s(primary.productivity)   3.37      9  27.48 2.04e-07 ***
## ---
## Signif. codes:  0 '***' 0.001 '**' 0.01 '*' 0.05 '.' 0.1 ' ' 1
##
## R-sq.(adj) =  0.455   Deviance explained = 44.6%
## -REML = 2331.9  Scale est. = 1         n = 705
```

```
shallow.es50.chlorophyll <- gam(ES50 ~ s(lat, lon, bs = "sos") + s(chlorophyll),
data = shallow, family = "nb", method = "REML", select = TRUE)
gam.check(shallow.es50.chlorophyll)
```

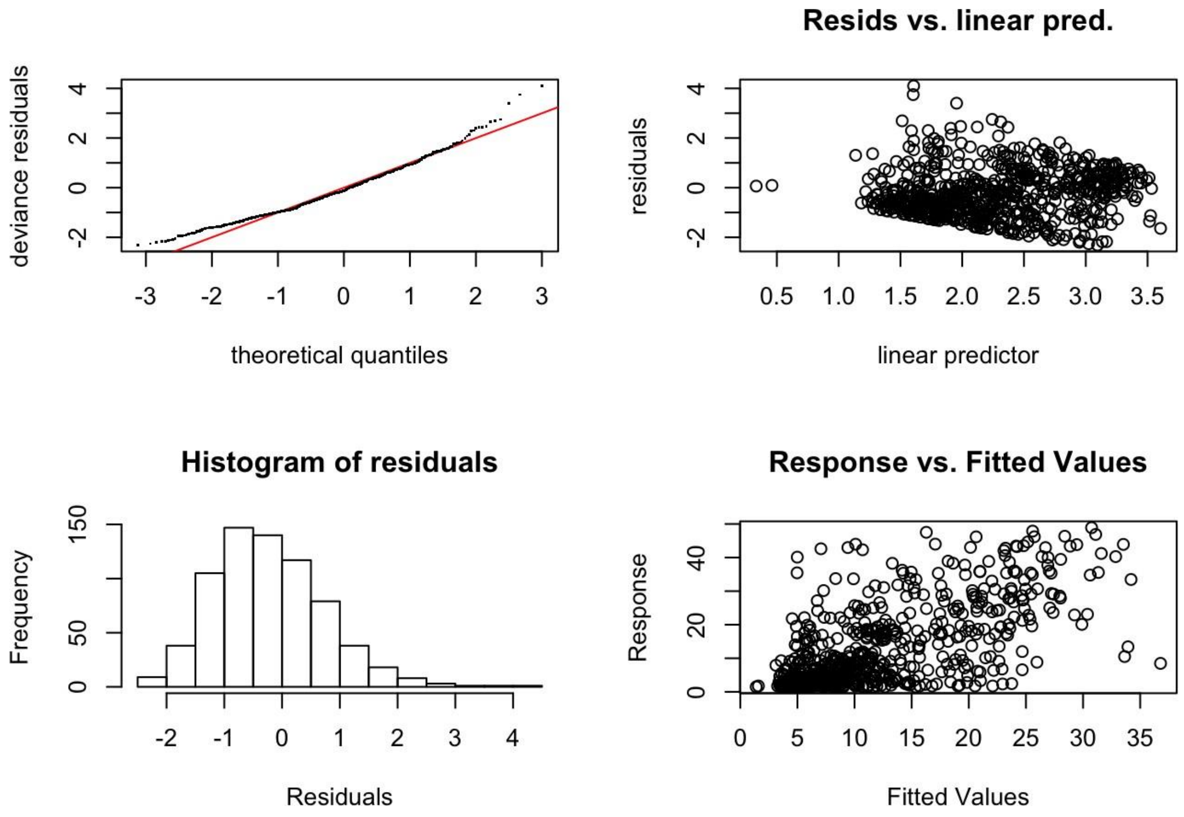


```
##
## Method: REML   Optimizer: outer newton
## full convergence after 3 iterations.
## Gradient range [-1.322651e-05,9.523495e-05]
## (score 2339.164 & scale 1).
## Hessian positive definite, eigenvalue range [0.1412794,231.8289].
## Model rank =  59 / 59
##
## Basis dimension (k) checking results. Low p-value (k-index<1) may
## indicate that k is too low, especially if edf is close to k'.
##
##                  k'  edf k-index p-value
## s(lat,lon)     49.0 32.9    0.84  <2e-16 ***
## s(chlorophyll)  9.0  3.0    0.93    0.29
## ---
## Signif. codes:  0 '***' 0.001 '**' 0.01 '*' 0.05 '.' 0.1 ' ' 1
```

```
summary(shallow.es50.chlorophyll)
```

```
##
## Family: Negative Binomial(2.132)
## Link function: log
##
## Formula:
## ES50 ~ s(lat, lon, bs = "sos") + s(chlorophyll)
##
## Parametric coefficients:
##             Estimate Std. Error z value Pr(>|z|)
## (Intercept)  2.27910    0.02892   78.81   <2e-16 ***
## ---
## Signif. codes:  0 '***' 0.001 '**' 0.01 '*' 0.05 '.' 0.1 ' ' 1
##
## Approximate significance of smooth terms:
##                   edf Ref.df Chi.sq p-value
## s(lat,lon)     32.934     49 390.67  <2e-16 ***
## s(chlorophyll)  2.999      9  12.88   9e-04 ***
## ---
## Signif. codes:  0 '***' 0.001 '**' 0.01 '*' 0.05 '.' 0.1 ' ' 1
##
## R-sq.(adj) =  0.418   Deviance explained = 42.8%
## -REML = 2339.2  Scale est. = 1         n = 705
```

```
shallow.es50.current <- gam(ES50 ~ s(lat, lon, bs = "sos") + s(current), data = s
hallow, family = "nb", method = "REML", select = TRUE)
gam.check(shallow.es50.current)
```

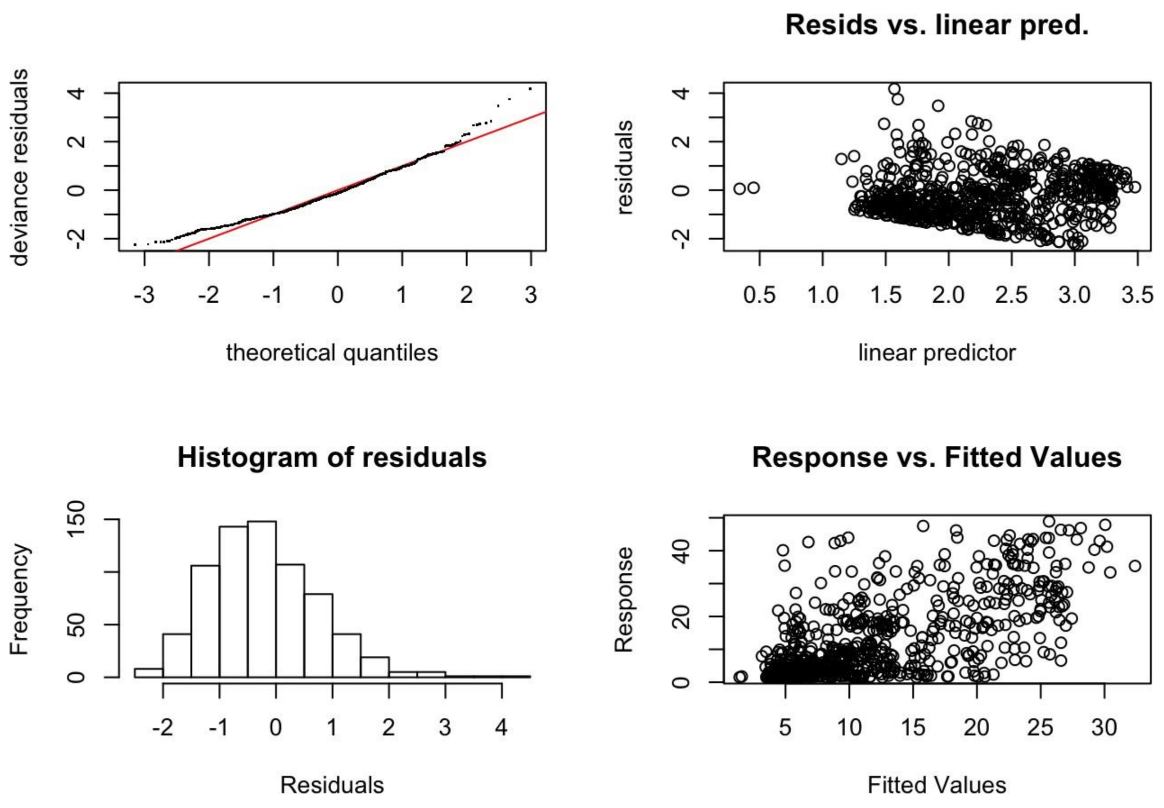

```
##
## Method: REML   Optimizer: outer newton
## full convergence after 6 iterations.
## Gradient range [-0.0006727976,0.000479441]
## (score 2341.073 & scale 1).
## Hessian positive definite, eigenvalue range [0.0006722106,234.1461].
## Model rank =  59 / 59
##
## Basis dimension (k) checking results. Low p-value (k-index<1) may
## indicate that k is too low, especially if edf is close to k'.
##
##                  k'    edf k-index p-value
## s(lat,lon) 49.000 33.585    0.84  <2e-16 ***
## s(current)  9.000  0.664    0.89   0.045 *
## ---
## Signif. codes:  0 '***' 0.001 '**' 0.01 '*' 0.05 '.' 0.1 ' ' 1
```

```
summary(shallow.es50.current)
```

```
##
## Family: Negative Binomial(2.105)
## Link function: log
##
## Formula:
## ES50 ~ s(lat, lon, bs = "sos") + s(current)
##
## Parametric coefficients:
##             Estimate Std. Error z value Pr(>|z|)
## (Intercept)  2.28275    0.02904   78.59   <2e-16 ***
## ---
## Signif. codes:  0 '***' 0.001 '**' 0.01 '*' 0.05 '.' 0.1 ' ' 1
##
## Approximate significance of smooth terms:
##               edf Ref.df  Chi.sq p-value
## s(lat,lon) 33.585     49 424.082  <2e-16 ***
## s(current)  0.664      9   1.992  0.0701 .
## ---
## Signif. codes:  0 '***' 0.001 '**' 0.01 '*' 0.05 '.' 0.1 ' ' 1
##
## R-sq.(adj) =   0.42   Deviance explained = 41.9%
## -REML = 2341.1  Scale est. = 1         n = 705
```

```
shallow.es50.o2.saturate <- gam(ES50 ~ s(lat, lon, bs = "sos") + s(o2.saturate),
data = shallow, family = "nb", method = "REML", select = TRUE)
gam.check(shallow.es50.o2.saturate)
```

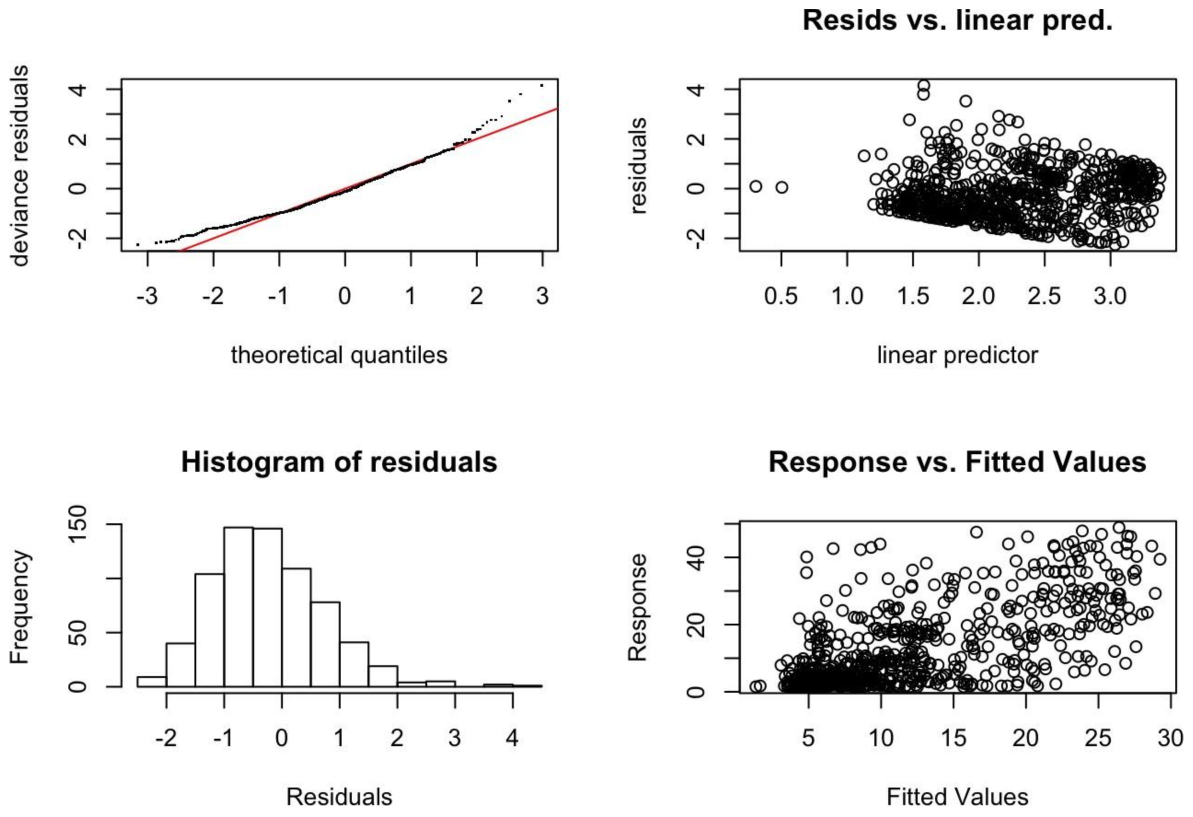


```
##
## Method: REML   Optimizer: outer newton
## full convergence after 3 iterations.
## Gradient range [-0.0006824379,0.0001868394]
## (score 2341.236 & scale 1).
## Hessian positive definite, eigenvalue range [0.0002624635,233.1806].
## Model rank =  59 / 59
##
## Basis dimension (k) checking results. Low p-value (k-index<1) may
## indicate that k is too low, especially if edf is close to k'.
##
##                    k'   edf k-index p-value
## s(lat,lon)      49.00 34.06    0.84  <2e-16 ***
## s(o2.saturate)   9.00  1.17    0.85  <2e-16 ***
## ---
## Signif. codes:  0 '***' 0.001 '**' 0.01 '*' 0.05 '.' 0.1 ' ' 1
```

```
summary(shallow.es50.o2.saturate)
```

```
##
## Family: Negative Binomial(2.113)
## Link function: log
##
## Formula:
## ES50 ~ s(lat, lon, bs = "sos") + s(o2.saturate)
##
## Parametric coefficients:
##              Estimate Std. Error z value Pr(>|z|)
## (Intercept)   2.28146    0.02901   78.65   <2e-16 ***
## ---
## Signif. codes:  0 '***' 0.001 '**' 0.01 '*' 0.05 '.' 0.1 ' ' 1
##
## Approximate significance of smooth terms:
##                  edf Ref.df  Chi.sq p-value
## s(lat,lon)     34.06     49 420.777  <2e-16 ***
## s(o2.saturate)  1.17      9   2.303  0.0988 .
## ---
## Signif. codes:  0 '***' 0.001 '**' 0.01 '*' 0.05 '.' 0.1 ' ' 1
##
## R-sq.(adj) =  0.418   Deviance explained = 42.2%
## -REML = 2341.2  Scale est. = 1        n = 705
```

```
shallow.es50.salinity <- gam(ES50 ~ s(lat, lon, bs = "sos") + s(salinity), data =
shallow, family = "nb", method = "REML", select = TRUE)
gam.check(shallow.es50.salinity)
```

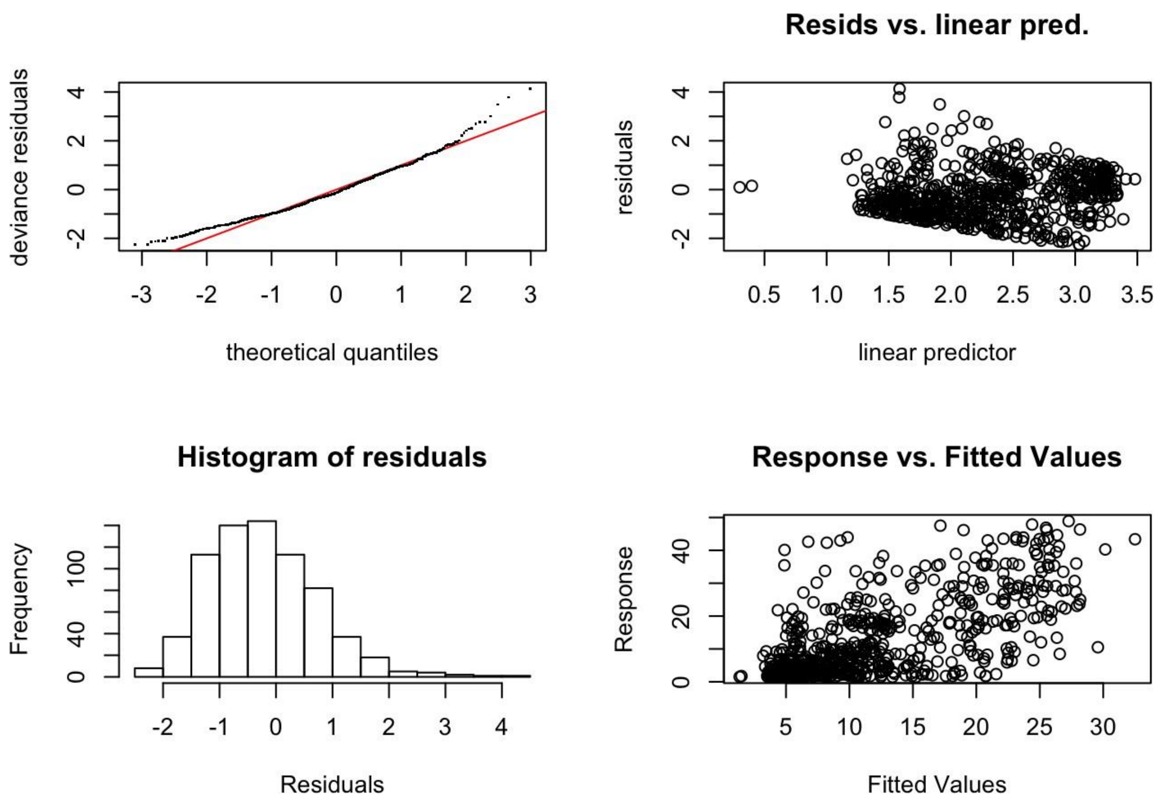

```
##
## Method: REML   Optimizer: outer newton
## full convergence after 6 iterations.
## Gradient range [-0.001116754,0.0001808395]
## (score 2340.619 & scale 1).
## Hessian positive definite, eigenvalue range [0.0405921,233.8519].
## Model rank =  59 / 59
##
## Basis dimension (k) checking results. Low p-value (k-index<1) may
## indicate that k is too low, especially if edf is close to k'.
##
##                  k'   edf k-index p-value
## s(lat,lon)    49.00 33.18    0.84  <2e-16 ***
## s(salinity)    9.00  1.09    0.91   0.075 .
## ---
## Signif. codes:  0 '***' 0.001 '**' 0.01 '*' 0.05 '.' 0.1 ' ' 1
```

```
summary(shallow.es50.salinity)
```

```
##
## Family: Negative Binomial(2.107)
## Link function: log
##
## Formula:
## ES50 ~ s(lat, lon, bs = "sos") + s(salinity)
##
## Parametric coefficients:
##             Estimate Std. Error z value Pr(>|z|)
## (Intercept)  2.28242    0.02904    78.6   <2e-16 ***
## ---
## Signif. codes:  0 '***' 0.001 '**' 0.01 '*' 0.05 '.' 0.1 ' ' 1
##
## Approximate significance of smooth terms:
##                edf Ref.df  Chi.sq p-value
## s(lat,lon)  33.177     49 335.316  <2e-16 ***
## s(salinity)  1.092      9   4.042  0.0188 *
## ---
## Signif. codes:  0 '***' 0.001 '**' 0.01 '*' 0.05 '.' 0.1 ' ' 1
##
## R-sq.(adj) =  0.418   Deviance explained =   42%
## -REML = 2340.6  Scale est. = 1         n = 705
```

```
shallow.es50.nitrate <- gam(ES50 ~ s(lat, lon, bs = "sos") + s(nitrate), data = s
hallow, family = "nb", method = "REML", select = TRUE)
gam.check(shallow.es50.nitrate)
```

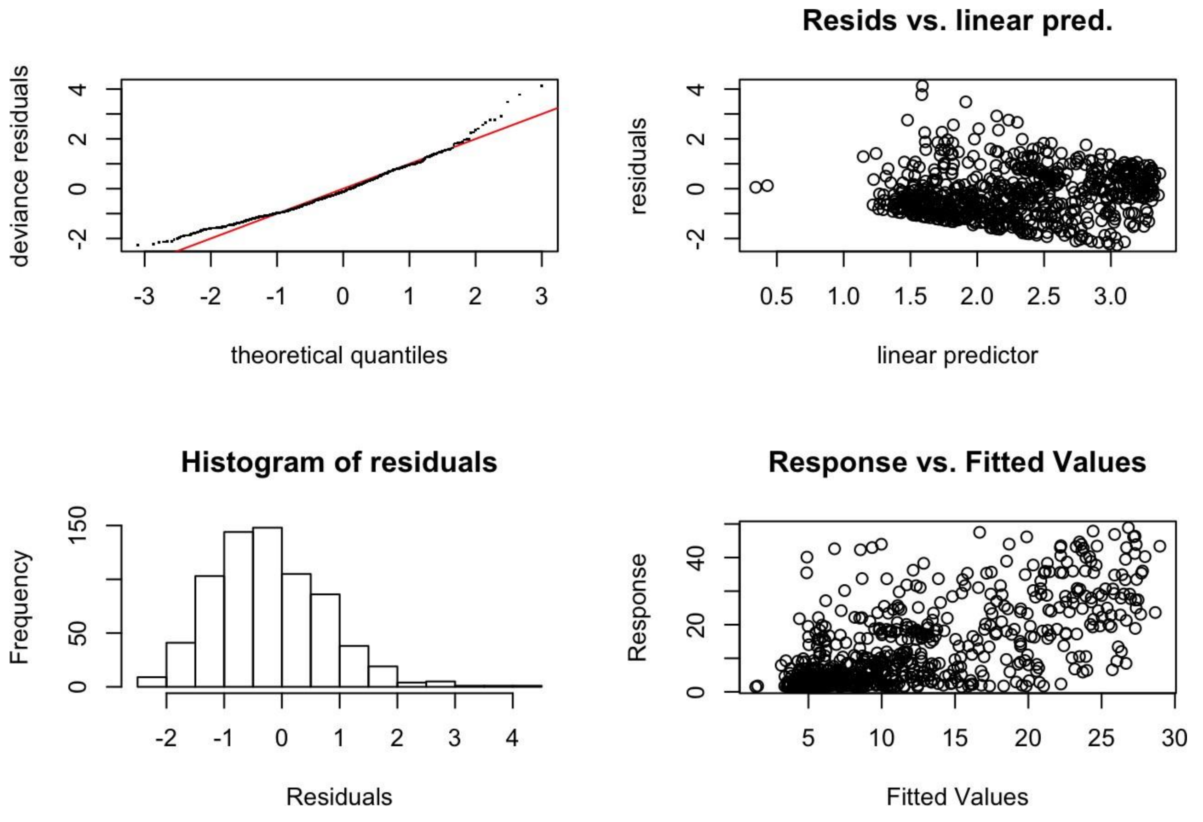


```
##
## Method: REML   Optimizer: outer newton
## full convergence after 6 iterations.
## Gradient range [-0.0008761779,0.0005350955]
## (score 2341.561 & scale 1).
## Hessian positive definite, eigenvalue range [1.174703e-05,234.1503].
## Model rank =  59 / 59
##
## Basis dimension (k) checking results. Low p-value (k-index<1) may
## indicate that k is too low, especially if edf is close to k'.
##
##                  k'     edf k-index p-value
## s(lat,lon) 49.0000 34.2330    0.84  <2e-16 ***
## s(nitrate)  9.0000  0.0019    0.90    0.13
## ---
## Signif. codes:  0 '***' 0.001 '**' 0.01 '*' 0.05 '.' 0.1 ' ' 1
```

```
summary(shallow.es50.nitrate)
```

```
##
## Family: Negative Binomial(2.106)
## Link function: log
##
## Formula:
## ES50 ~ s(lat, lon, bs =  "sos") +  s(nitrate)
##
## Parametric coefficients:
##                   Estimate Std. Error z value Pr(>|z|)
##  (Intercept)    2.28253     0.02904    78.59    <2e-16 ***
## ---
## Signif. codes:  0 '***' 0.001 '**' 0.01 '*' 0.05 '.' 0.1 ' ' 1
##
## Approximate significance of smooth terms:
##                          edf Ref.df Chi.sq  p-value
## s(lat,lon) 34.233015          49   436.6   <2e-16 ***
##  s(nitrate)   0.001901          9      0.0          1
## ---
## Signif. codes:  0 '***' 0.001 '**' 0.01 '*' 0.05 '.' 0.1 ' ' 1
##
##  R-sq.(adj)  =   0.415    Deviance  explained  =    42%
## -REML= 2341.6Scale  est.  =  1                   n = 705
```

```
shallow.es50.env <- gam(ES50 ~ s(lat, lon, bs = "sos") + s(temp) + s(dissolved.oxygen) + s(primary.productivity) + s(chlorophyll) + s(current) + s(o2.saturate) + s(salinity) + s(nitrate), data = shallow, family = "nb", method = "REML", select = TRUE)
gam.check(shallow.es50.env)
```

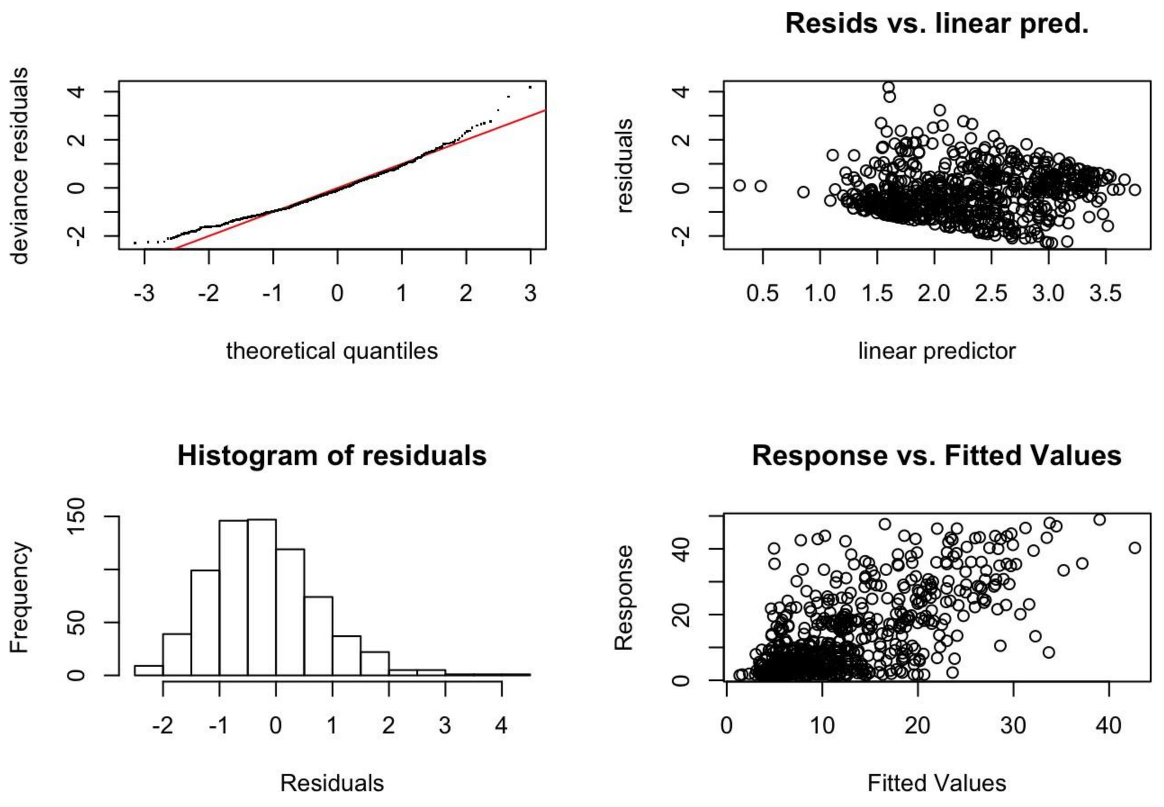

```
##
## Method: REML   Optimizer: outer newton
## full convergence after 9 iterations.
## Gradient range [-0.001211478,6.695132e-05]
## (score 2331.218 & scale 1).
## Hessian positive definite, eigenvalue range [1.934616e-05,225.6091].
## Model rank =  122 / 122
##
## Basis dimension (k) checking results. Low p-value (k-index<1) may
## indicate that k is too low, especially if edf is close to k'.
##
##                              k'      edf k-index p-value
## s(lat,lon)             4.90e+01 3.30e+01    0.85  <2e-16 ***
## s(temp)                9.00e+00 6.46e-04    0.99   0.850
## s(dissolved.oxygen)    9.00e+00 4.76e-01    0.95   0.515
## s(primary.productivity) 9.00e+00 3.37e+00    0.99   0.820
## s(chlorophyll)         9.00e+00 1.13e-03    0.93   0.300
## s(current)             9.00e+00 1.09e-03    0.90   0.075 .
## s(o2.saturate)         9.00e+00 4.17e-01    0.85   0.010 **
## s(salinity)            9.00e+00 8.96e-01    0.91   0.175
## s(nitrate)             9.00e+00 8.89e-02    0.91   0.100 .
## ---
## Signif. codes:  0 '***' 0.001 '**' 0.01 '*' 0.05 '.' 0.1 ' ' 1
```

```
summary(shallow.es50.env)
```

```
##
## Family: Negative Binomial(2.212)
## Link function: log
##
## Formula:
## ES50 ~ s(lat, lon, bs = "sos") + s(temp) + s(dissolved.oxygen) +
##       s(primary.productivity) + s(chlorophyll) +  s(current)  +  s(o2.saturate)  +
##       s(salinity) +  s(nitrate)
##
## Parametric coefficients:
##                  Estimate Std. Error z value Pr(>|z|)
##  (Intercept)   2.27029     0.02854    79.54    <2e-16 ***
## ---
## Signif. codes:  0 '***' 0.001 '**' 0.01 '*' 0.05 '.' 0.1 ' ' 1
##
## Approximate significance of smooth terms:
##                                  edf  Ref.df   Chi.sq  p-value
## s(lat,lon)                 32.976231      49  340.790  < 2e-16  ***
## s(temp)                     0.000646       9    0.000   0.2707
## s(dissolved.oxygen)         0.476287       9    0.577   0.1161
## s(primary.productivity)     3.367451       9   26.460  2.2e-07  ***
## s(chlorophyll)              0.001128       9    0.001   0.3742
## s(current)                  0.001091       9    0.001   0.4165
## s(o2.saturate)              0.417496       9    0.719   0.1609
## s(salinity)                 0.896068       9    2.753   0.0356  *
## s(nitrate)                  0.088862       9    0.089   0.2446
## ---
## Signif. codes:  0 '***' 0.001 '**' 0.01 '*' 0.05 '.' 0.1 ' ' 1
##
## R-sq.(adj) =   0.456     Deviance explained = 44.7%
## -REML = 2331.2 Scale  est. =  1                n =  705
```

Model selection for ES50, shallow water

```
shallow.es50.models <- list(shallow.es50.intercept = shallow.es50.intercept,
                            shallow.es50.latlon = shallow.es50.latlon,
                            shallow.es50.temp = shallow.es50.temp,
                            shallow.es50.oxygen = shallow.es50.oxygen,
                            shallow.es50.prod = shallow.es50.prod,
                            shallow.es50.chlorophyll = shallow.es50.chlorophyll,
                            shallow.es50.current = shallow.es50.current,
                            shallow.es50.o2.saturate = shallow.es50.o2.saturate,
                            shallow.es50.salinity = shallow.es50.salinity,
                            shallow.es50.nitrate = shallow.es50.nitrate,
                            shallow.es50.env = shallow.es50.env)
shallow.es50.aic.df <- data.frame(Model = names(shallow.es50.models),
                                  AIC = sapply(shallow.es50.models, function(x) x$aic),
                                  akaike.weights(sapply(shallow.es50.models,  function(x)
 x$aic)))

shallow.es50.aic.df <- shallow.es50.aic.df[order(shallow.es50.aic.df$AIC),]
shallow.es50.aic.df$Cumulative.Weight <- cumsum(shallow.es50.aic.df$weights)

kable(shallow.es50.aic.df, row.names = FALSE)
```

Model	AIC	deltaAIC	rel.LL	weights	Cumulative.Weight
shallow.es50.env	4598.298	0.0000000	1.0000000	0.5066030	0.5066030
shallow.es50.prod	4598.351	0.0529881	0.9738538	0.4933573	0.9999603
shallow.es50.chlorophyll	4617.762	19.4646455	0.0000593	0.0000301	0.9999904
shallow.es50.o2.saturate	4623.036	24.7379396	0.0000042	0.0000022	0.9999925
shallow.es50.oxygen	4623.785	25.4870104	0.0000029	0.0000015	0.9999940
shallow.es50.salinity	4624.051	25.7526841	0.0000026	0.0000013	0.9999953
shallow.es50.latlon	4624.161	25.8631607	0.0000024	0.0000012	0.9999965
shallow.es50.temp	4624.162	25.8644170	0.0000024	0.0000012	0.9999978
shallow.es50.nitrate	4624.164	25.8659651	0.0000024	0.0000012	0.9999990
shallow.es50.current	4624.537	26.2390899	0.0000020	0.0000010	1.0000000
shallow.es50.intercept	4951.158	352.8604444	0.0000000	0.0000000	1.0000000

```
write.csv(shallow.es50.aic.df, file = "shallow.es50.aic.csv")
```

Here we find strong support for the effects of the environment on ES50 in shallow water. Given that primary productivity alone does almost as good a job as all environmental predictors at once, we believe that the predictive power here is mostly coming from primary productivity. The relatively poor performance of the spatial autocorrelation-only model (delta AIC of 29.6) suggests that there is significant predictive power in the environmental variables over and above what is expected due to spatial autocorrelation alone.

Plots for ES50, shallow water

```
qplot(shallow$temp, predict(shallow.es50.temp, shallow)) + geom_smooth(method = "ga
m")
```

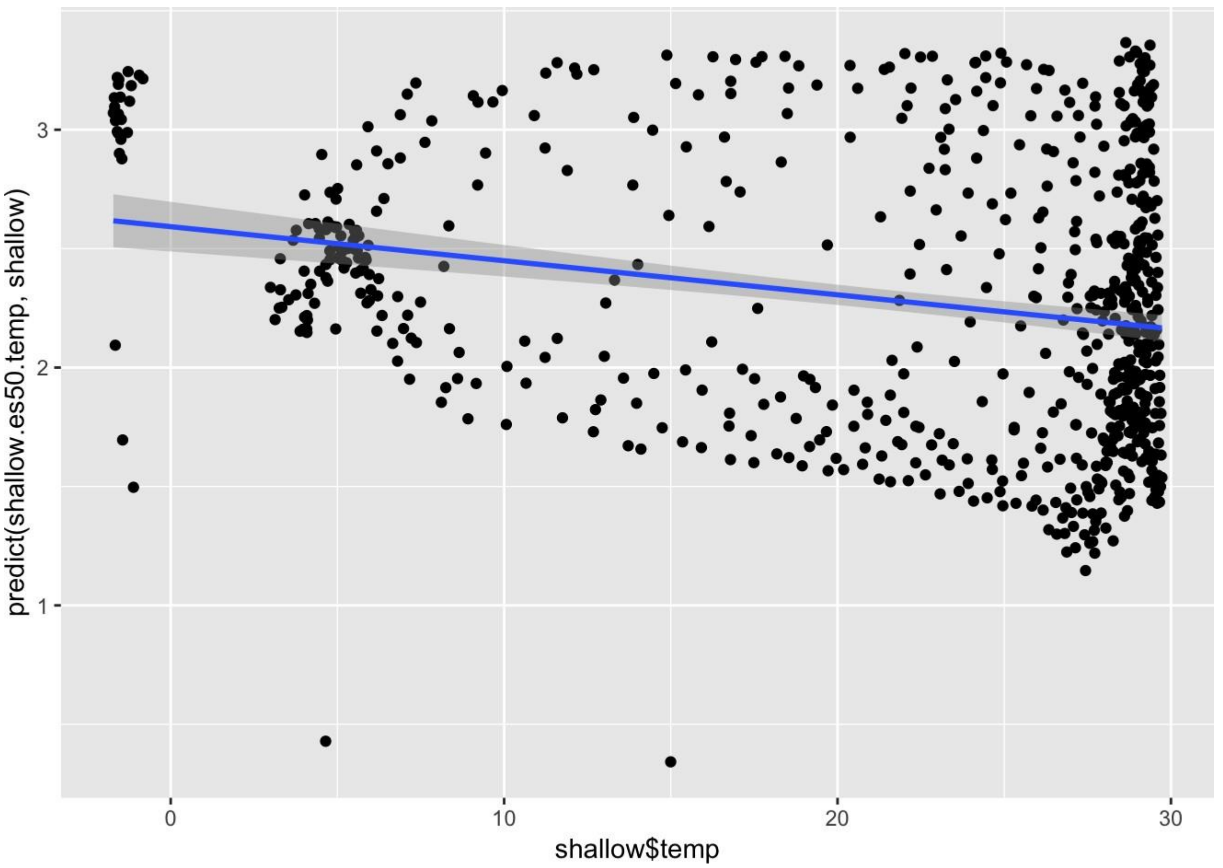


```
qplot(shallow$primary.productivity, predict(shallow.es50.prod, shallow)) + geom_smo
oth(method = "gam")
```

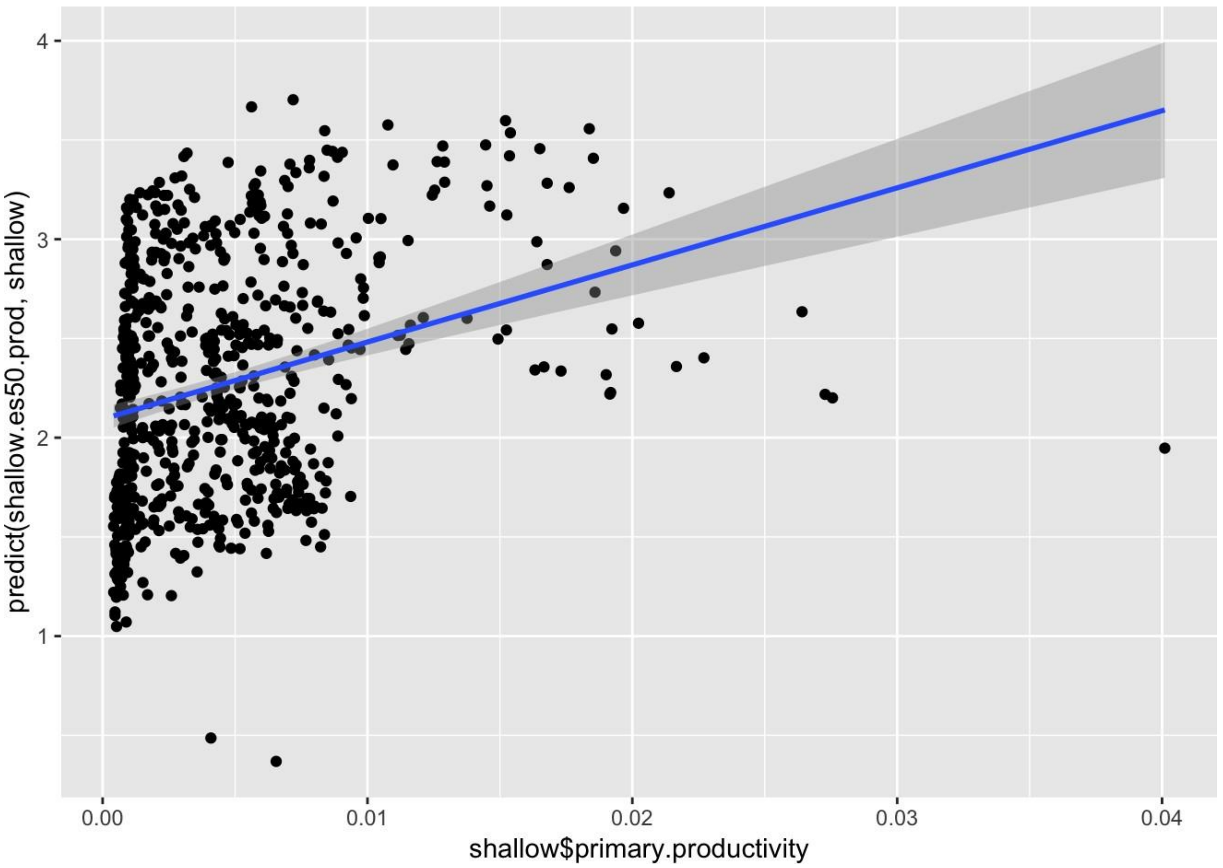


```
qplot(shallow$dissolved.oxygen, predict(shallow.es50.oxygen, shallow)) + geom_smoot
h(method = "gam")
```

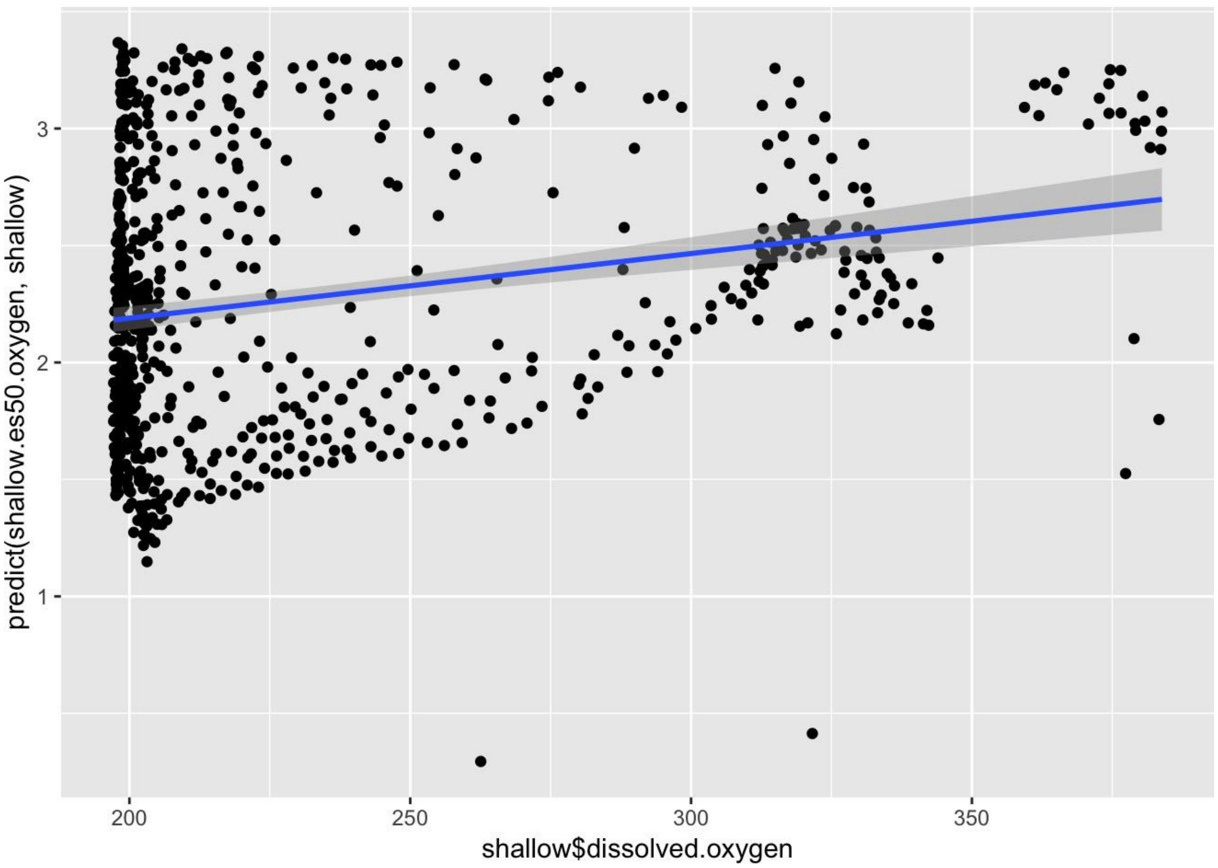


```
qplot(shallow$chlorophyll, predict(shallow.es50.chlorophyll, shallow)) + geom_smoot
h(method = "gam")
```

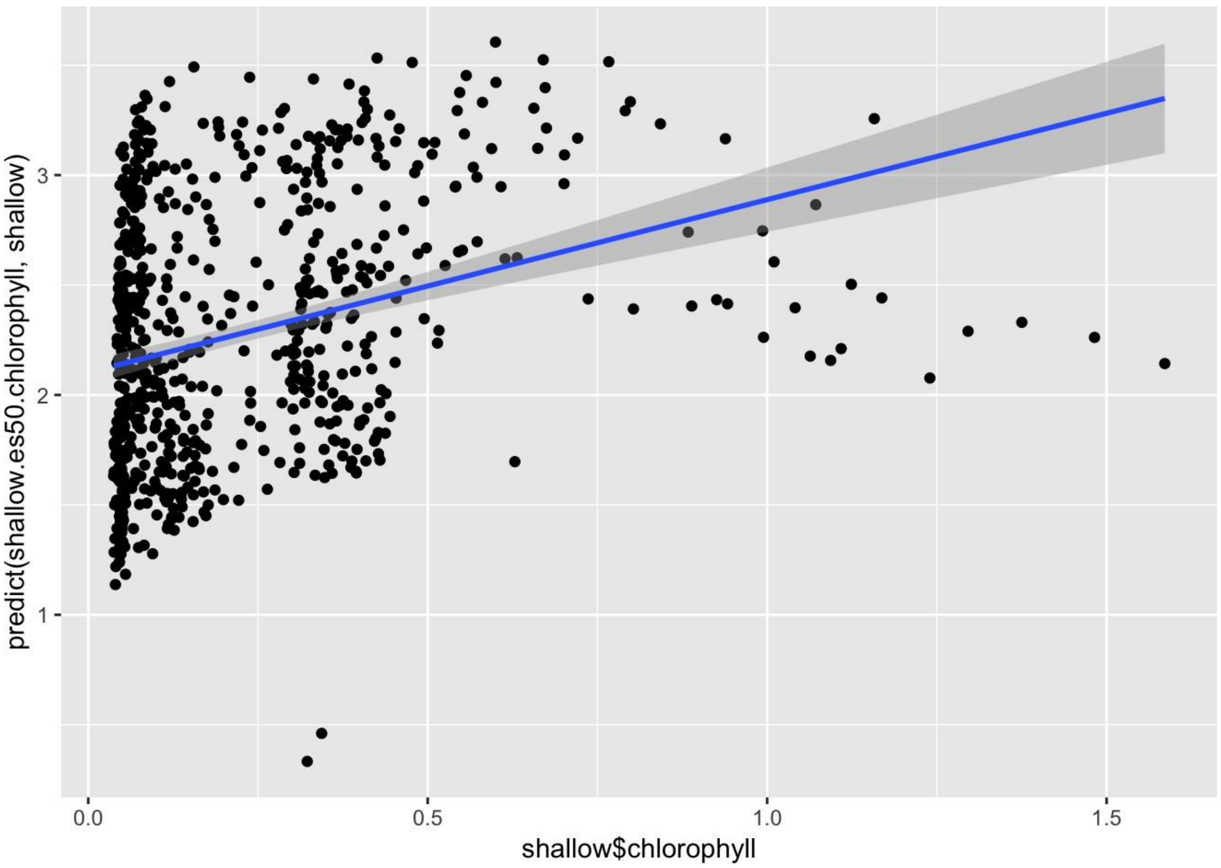


```
qplot(shallow$current, predict(shallow.es50.current, shallow)) + geom_smooth(method
= "gam")
```

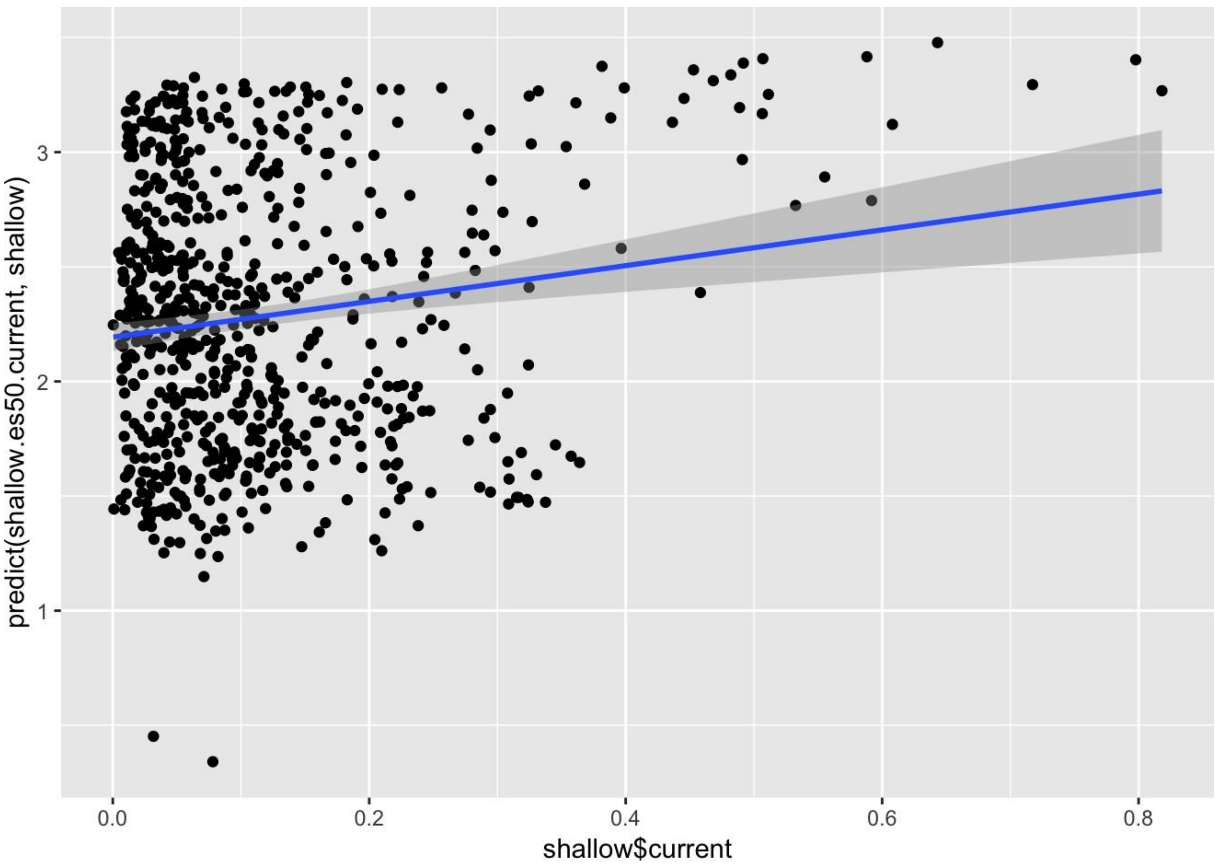


```
qplot(shallow$o2.saturate, predict(shallow.es50.o2.saturate, shallow)) + geom_smoot
h(method = "gam")
```

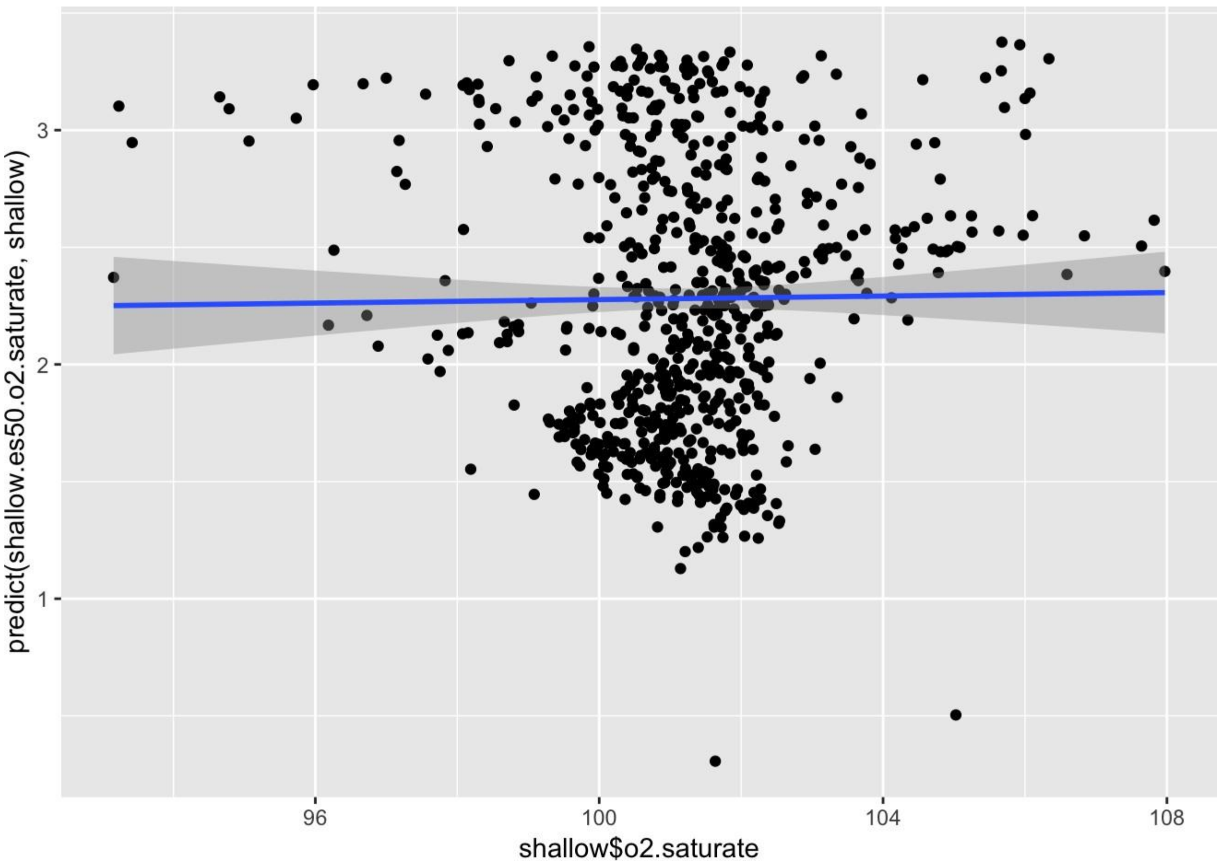


```
qplot(shallow$salinity,   predict(shallow.es50.salinity,   shallow))   +   geom_smooth(meth
od = "gam")
```

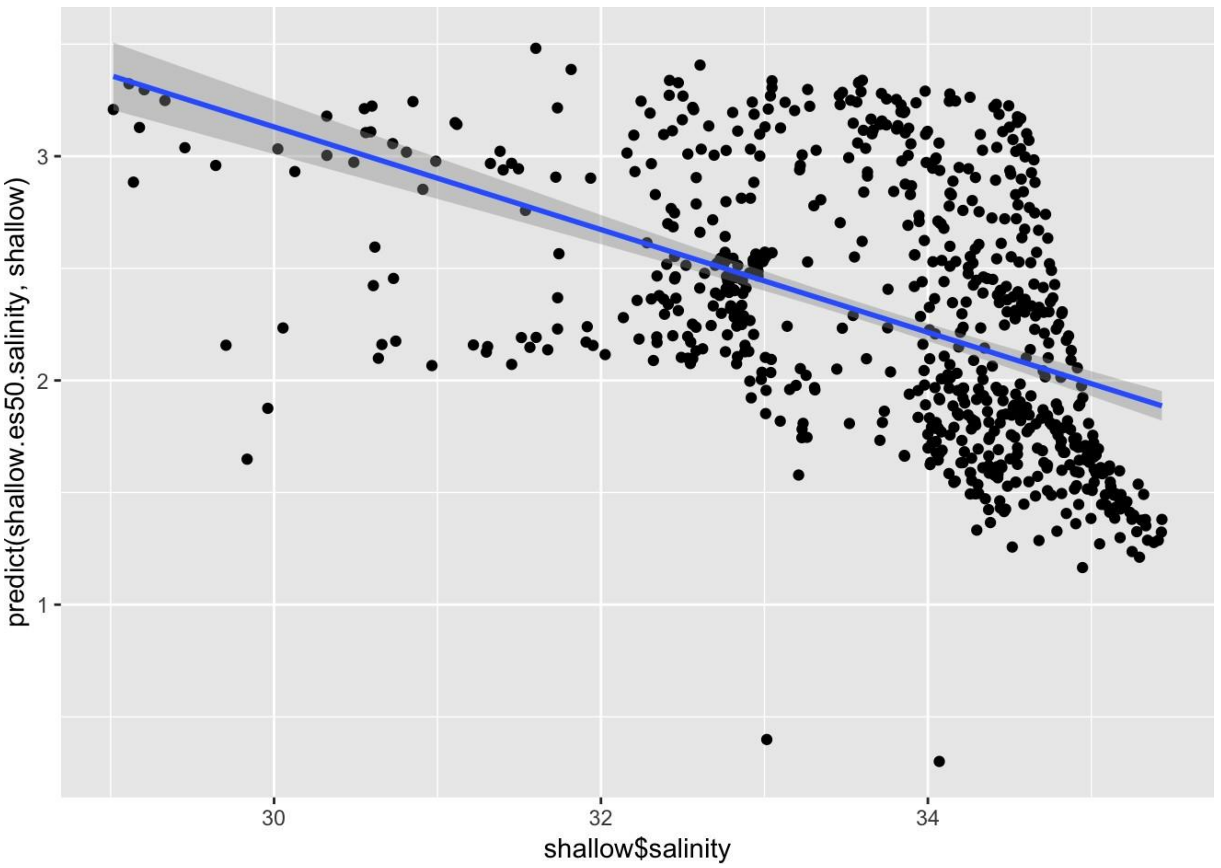


```
qplot(shallow$nitrate, predict(shallow.es50.nitrate, shallow)) + geom_smooth(method
= "gam")
```

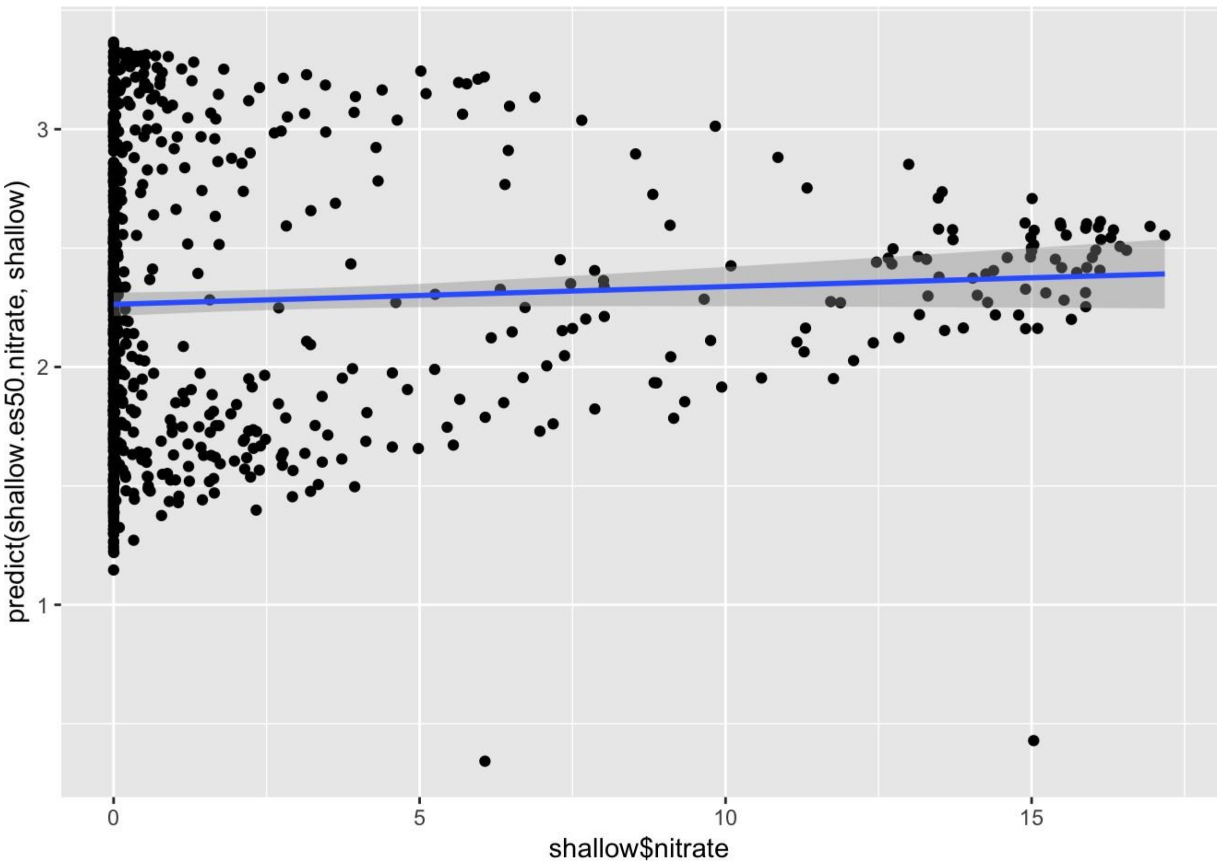

Deep water

Repeating the above analyses for deep-dwelling species.

Data load-in and visualization

```
deep <- read.csv("deep.csv")
analysis.cols <- c("lat", "lon", "area", "num.records", "num.species", "ES50", "dis
solved.oxygen", "temp", "chlorophyll", "current", "salinity", "nitrate")
deep <- deep[,analysis.cols]
deep <- deep[complete.cases(deep),]
corrplot(cor(deep))
```

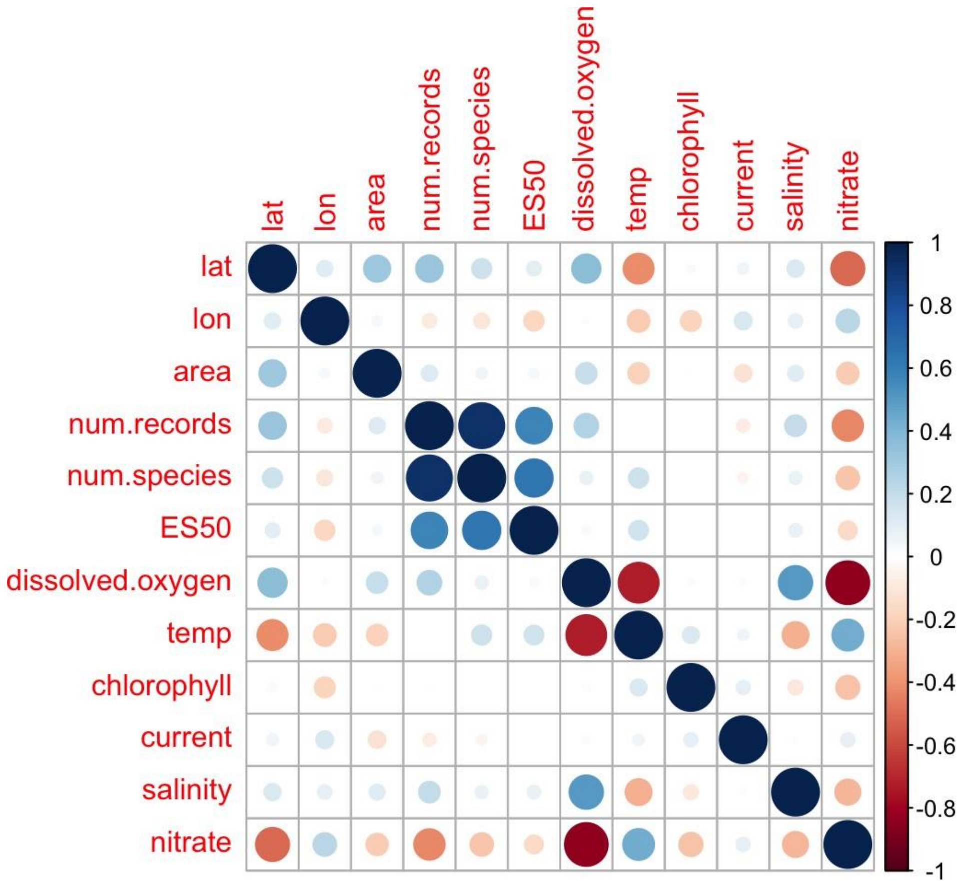

GAMs for number of species, deep water

As above, we’re going to develop a number of GAMs including one for “intercept”, one for “latlon”, one for all environmental predictors (“env”), and then one for each individual predictor.

```
deep.numsp.intercept <- gam(num.species ~ 1, data = deep, family = "nb", method =
"REML", select = TRUE)
gam.check(deep.numsp.intercept)
```

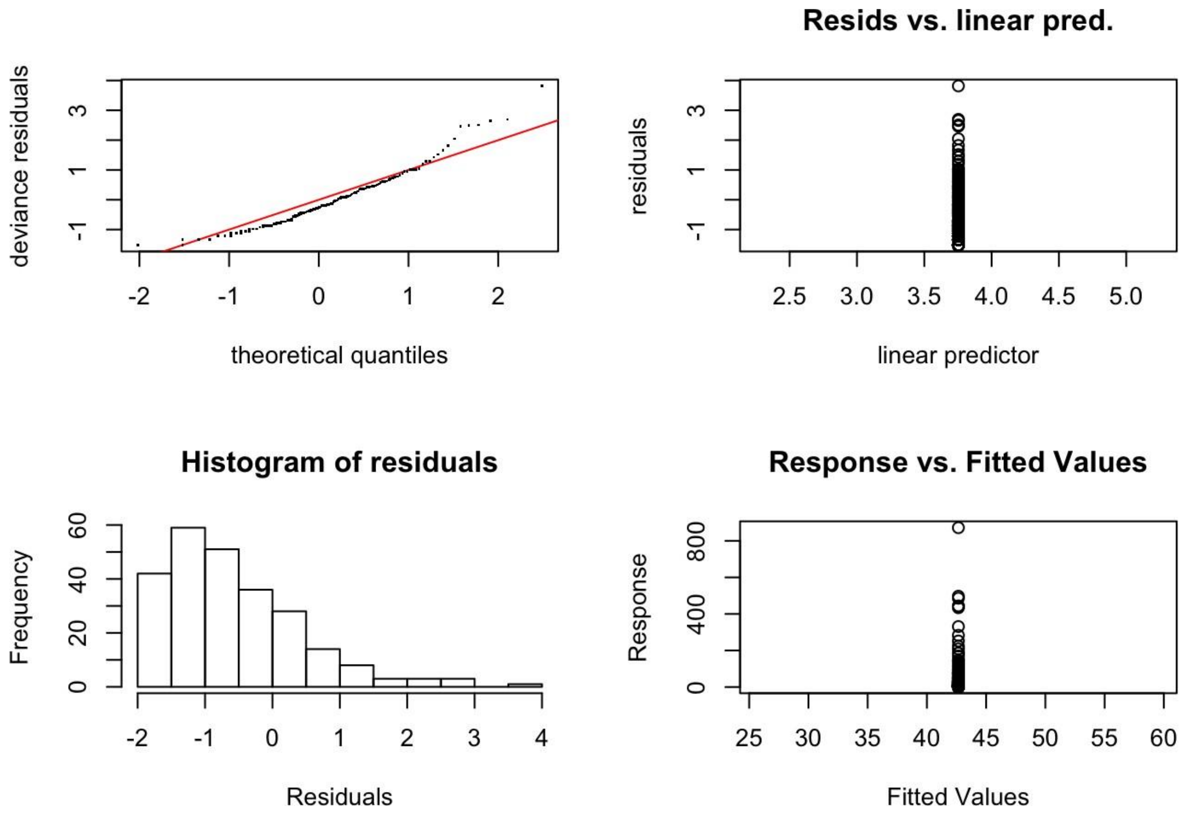


```
##
## Method: REML    Optimizer: outer newton
## full convergence after 4 iterations.
## Gradient range [1.064992e-07,1.064992e-07]
## (score 1115.547 & scale 1).
## Hessian positive definite, eigenvalue range [172.3917,172.3917].
## Model rank =  1 / 1
```

```
summary(deep.numsp.intercept)
```

```
##
## Family: Negative Binomial(0.446)
## Link function: log
##
## Formula:
## num.species ~ 1
##
## Parametric coefficients:
##             Estimate Std. Error z value Pr(>|z|)
## (Intercept)  3.75320    0.09555   39.28   <2e-16 ***
## ---
## Signif. codes:  0 '***' 0.001 '**' 0.01 '*' 0.05 '.' 0.1 ' ' 1
##
##
## R-sq.(adj) =  2.22e-16   Deviance explained = 1.42e-09%
## -REML = 1115.5  Scale est. = 1         n = 248
```

```
deep.numsp.numrec <- gam(num.species ~ s(num.records), data = deep, family = "nb",
 method = "REML", select = TRUE)
gam.check(deep.numsp.numrec)
```

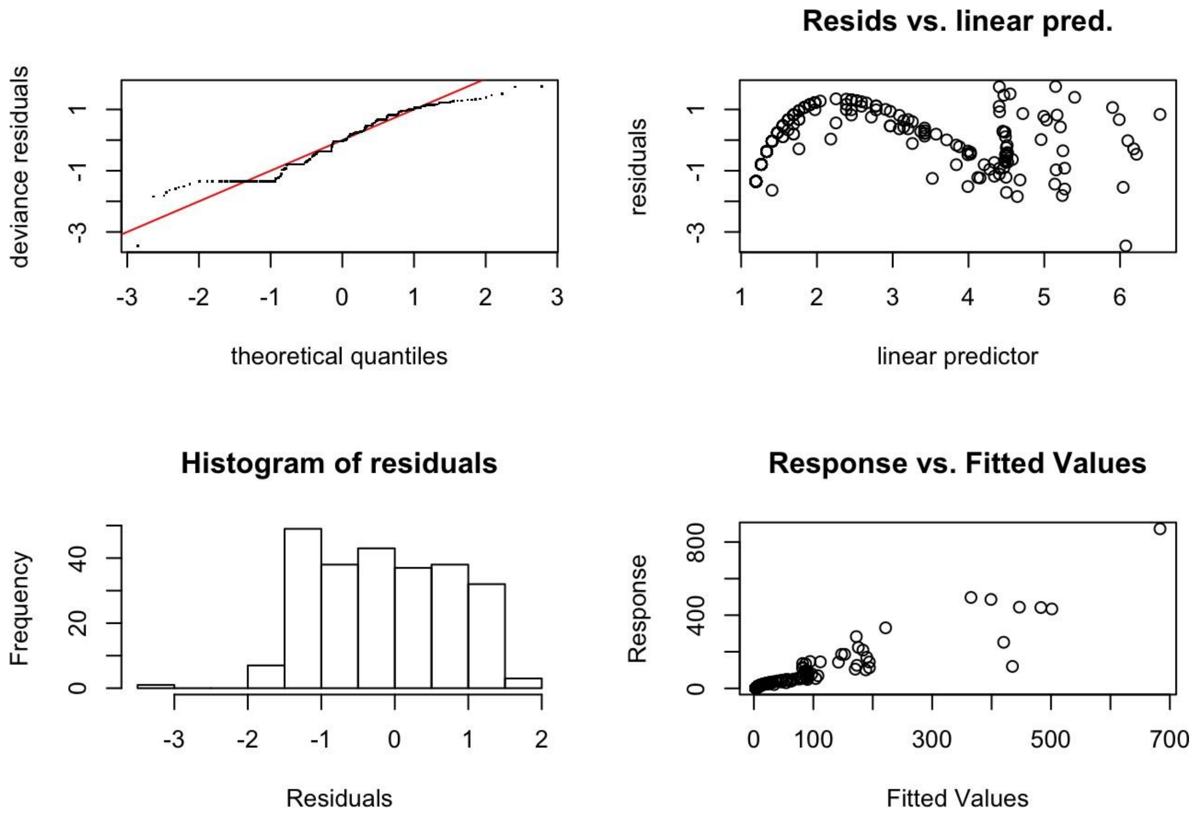


```
##
## Method: REML   Optimizer: outer newton
## full convergence after 5 iterations.
## Gradient range [-7.448098e-08,3.599384e-07]
## (score 789.128 & scale 1).
## Hessian positive definite, eigenvalue range [0.4809454,38.37389].
## Model rank =  10 / 10
##
## Basis dimension (k) checking results. Low p-value (k-index<1) may
## indicate that k is too low, especially if edf is close to k'.
##
##                  k'  edf k-index p-value
## s(num.records) 9.00 8.69    0.27  <2e-16 ***
## ---
## Signif. codes:  0 '***' 0.001 '**' 0.01 '*' 0.05 '.' 0.1 ' ' 1
```

```
summary(deep.numsp.numrec)
```

```
##
## Family: Negative Binomial(11.031)
## Link function: log
##
## Formula:
## num.species ~ s(num.records)
##
## Parametric coefficients:
##               Estimate Std. Error z value Pr(>|z|)
## (Intercept)   2.52278    0.03118   80.91   <2e-16 ***
## ---
## Signif. codes:  0 '***' 0.001 '**' 0.01 '*' 0.05 '.' 0.1 ' ' 1
##
## Approximate significance of smooth terms:
##                    edf Ref.df Chi.sq  p-value
## s(num.records) 8.691      9   3245  <2e-16 ***
## ---
## Signif. codes:  0 '***' 0.001 '**' 0.01 '*' 0.05 '.' 0.1 ' ' 1
##
## R-sq.(adj) =  0.872   Deviance explained = 95.3%
## -REML = 789.13  Scale est. = 1         n = 248
```

```
deep.numsp.latlon <- gam(num.species ~ s(lat, lon, bs = "sos") + s(num.records), da ta = deep, family = "nb", method = "REML", select = TRUE) gam.check(deep.numsp.latlon)
```

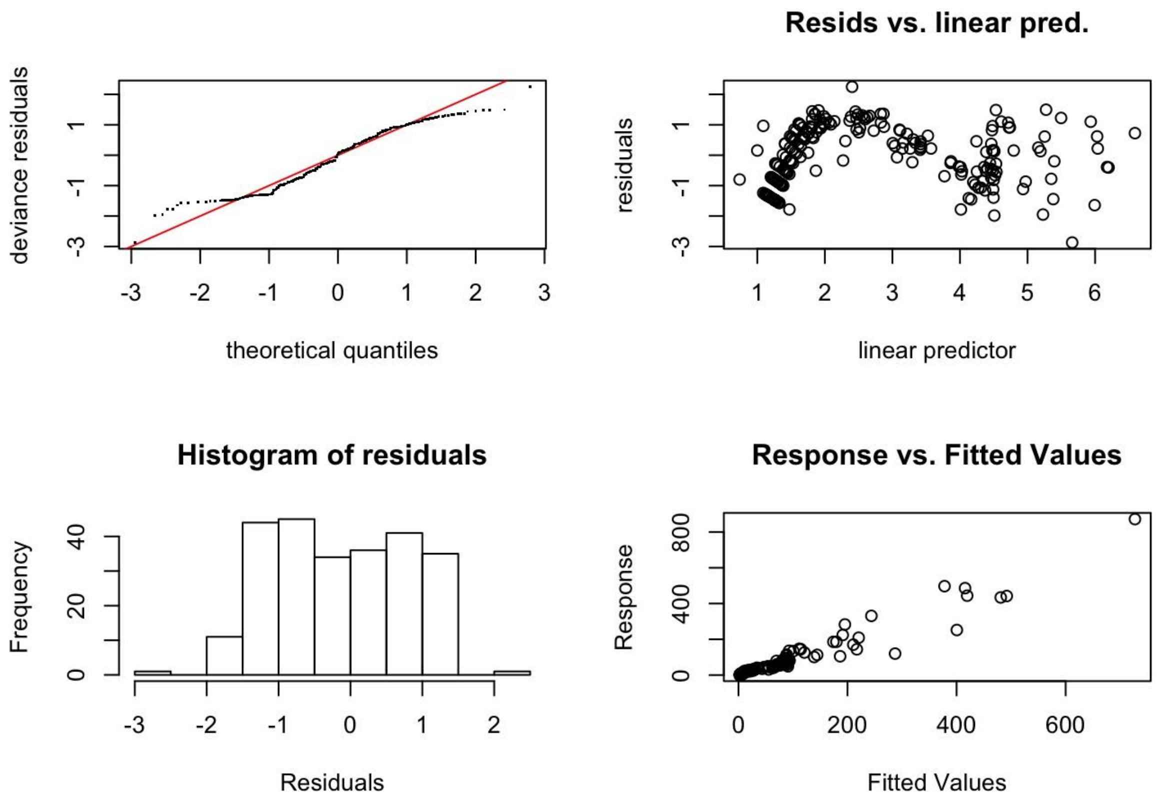

```
##
## Method: REML   Optimizer: outer newton
## full convergence after 13 iterations.
## Gradient range [-2.084442e-06,1.813092e-05]
## (score 783.3275 & scale 1).
## Hessian positive definite, eigenvalue range [0.4865975,26.20744].
## Model rank =  59 / 59
##
## Basis dimension (k) checking results. Low p-value (k-index<1) may
## indicate that k is too low, especially if edf is close to k'.
##
##                    k'  edf k-index p-value
## s(lat,lon)      49.00 6.48    0.97    0.39
## s(num.records)   9.00 8.72    0.21  <2e-16 ***
## ---
## Signif. codes:  0 '***' 0.001 '**' 0.01 '*' 0.05 '.' 0.1 ' ' 1
```

```
summary(deep.numsp.latlon)
```

```
##
## Family: Negative Binomial(15.245)
## Link function: log
##
## Formula:
## num.species ~ s(lat, lon, bs = "sos") + s(num.records)
##
## Parametric coefficients:
##             Estimate Std. Error z value Pr(>|z|)
## (Intercept)   2.5224     0.0294   85.81   <2e-16 ***
## ---
## Signif. codes:  0 '***' 0.001 '**' 0.01 '*' 0.05 '.' 0.1 ' ' 1
##
## Approximate significance of smooth terms:
##                  edf Ref.df   Chi.sq  p-value
## s(lat,lon)     6.482     49    28.61 4.42e-06 ***
## s(num.records) 8.721      9  3057.11  < 2e-16 ***
## ---
## Signif. codes:  0 '***' 0.001 '**' 0.01 '*' 0.05 '.' 0.1 ' ' 1
##
## R-sq.(adj) =  0.929   Deviance explained = 96.1%
## -REML = 783.33  Scale est. = 1         n = 248
```

```
deep.numsp.temp <- gam(num.species ~ s(lat, lon, bs = "sos") + s(num.records) + s(t
emp), data = deep, family = "nb", method = "REML", select = TRUE)
gam.check(deep.numsp.temp)
```

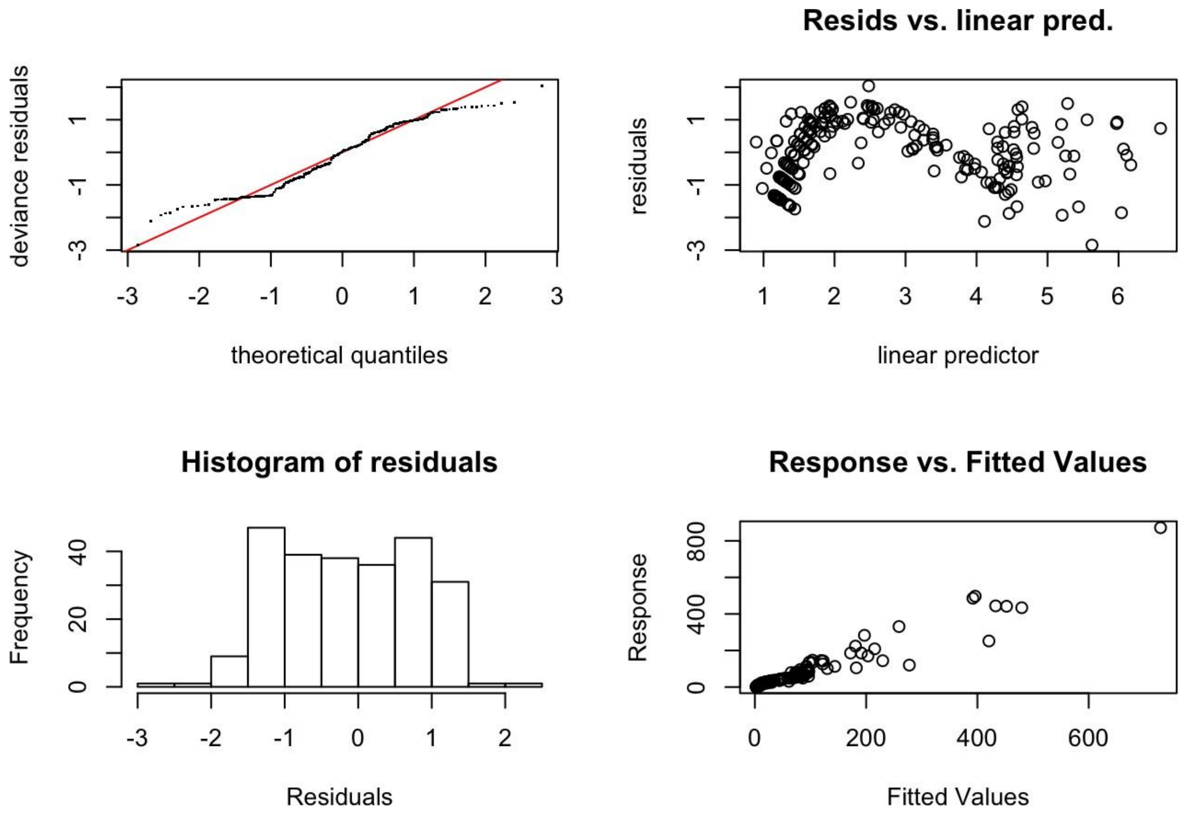


```
##
## Method: REML   Optimizer: outer newton
## full convergence after 16 iterations.
## Gradient range [-0.0001830443,0.0002958337]
## (score 779.4414 & scale 1).
## Hessian positive definite, eigenvalue range [0.1047609,25.92906].
## Model rank =  68 / 68
##
## Basis dimension (k) checking results. Low p-value (k-index<1) may
## indicate that k is too low, especially if edf is close to k'.
##
##                     k'   edf  k-index p-value
## s(lat,lon)       49.00  1.33     0.95    0.28
## s(num.records)    9.00  8.74     0.24  <2e-16 ***
## s(temp)           9.00  2.98     0.98    0.45
## ---
## Signif. codes:  0 '***' 0.001 '**' 0.01 '*' 0.05 '.' 0.1 ' ' 1
```

```
summary(deep.numsp.temp)
```

```
##
## Family: Negative Binomial(16.141)
## Link function: log
##
## Formula:
## num.species ~ s(lat, lon, bs = "sos") + s(num.records) + s(temp)
##
## Parametric coefficients:
##              Estimate Std. Error z value Pr(>|z|)
## (Intercept)   2.52107    0.02916   86.47   <2e-16 ***
## ---
## Signif. codes:  0 '***' 0.001 '**' 0.01 '*' 0.05 '.' 0.1 ' ' 1
##
## Approximate significance of smooth terms:
##                  edf Ref.df   Chi.sq  p-value
## s(lat,lon)     1.327     49    1.931    0.175
## s(num.records) 8.739      9 3286.489  < 2e-16 ***
## s(temp)        2.977      9   24.634 1.36e-06 ***
## ---
## Signif. codes:  0 '***' 0.001 '**' 0.01 '*' 0.05 '.' 0.1 ' ' 1
##
## R-sq.(adj) =  0.932   Deviance explained = 96.2%
## -REML=779.44 Scale est. = 1          n = 248
```

```
deep.numsp.oxygen <- gam(num.species ~ s(lat, lon, bs = "sos") + s(num.records) +
s(dissolved.oxygen), data = deep, family = "nb", method = "REML", select = TRUE)
gam.check(deep.numsp.oxygen)
```

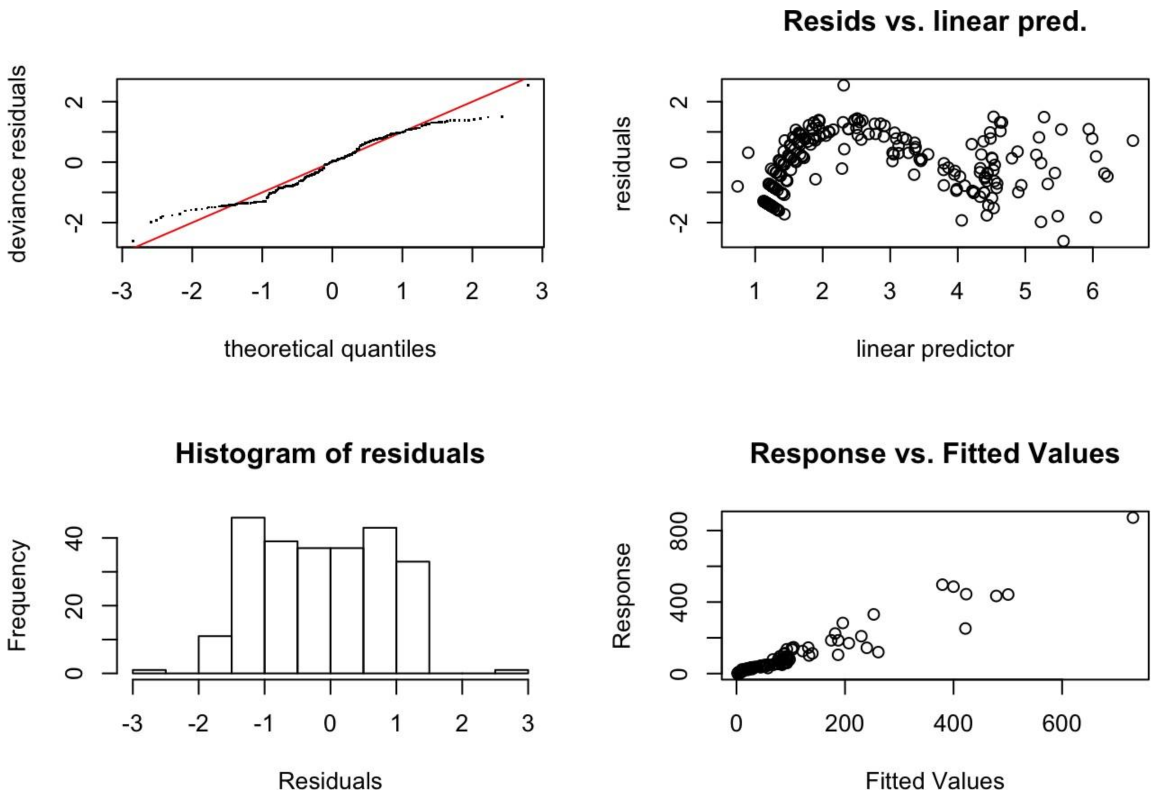

```
##
## Method: REML   Optimizer: outer newton
## full convergence after 15 iterations.
## Gradient range [-4.828611e-06,3.365376e-06]
## (score 778.8793 & scale 1).
## Hessian positive definite, eigenvalue range [0.07966418,27.44467].
## Model rank =  68 / 68
##
## Basis dimension (k) checking results. Low p-value (k-index<1) may
## indicate that k is too low, especially if edf is close to k'.
##
##                          k'   edf k-index p-value
## s(lat,lon)           49.000 0.899    0.96    0.36
## s(num.records)        9.000 8.740    0.21  <2e-16 ***
## s(dissolved.oxygen)   9.000 1.799    0.97    0.49
## ---
## Signif. codes:  0 '***' 0.001 '**' 0.01 '*' 0.05 '.' 0.1 ' ' 1
```

```
summary(deep.numsp.oxygen)
```

```
##
## Family: Negative Binomial(15.476)
## Link function: log
##
## Formula:
## num.species ~ s(lat, lon, bs = "sos") + s(num.records) + s(dissolved.oxygen)
##
## Parametric coefficients:
##             Estimate Std. Error z value Pr(>|z|)
## (Intercept)   2.5228     0.0293    86.1   <2e-16 ***
## ---
## Signif. codes:  0 '***' 0.001 '**' 0.01 '*' 0.05 '.' 0.1 ' ' 1
##
## Approximate significance of smooth terms:
##                        edf Ref.df   Chi.sq  p-value
## s(lat,lon)          0.8991     49    1.285    0.178
## s(num.records)      8.7404      9 3329.104  < 2e-16 ***
## s(dissolved.oxygen) 1.7995      9   23.743 2.24e-07 ***
## ---
## Signif. codes:  0 '***' 0.001 '**' 0.01 '*' 0.05 '.' 0.1 ' ' 1
##
## R-sq.(adj) =  0.929   Deviance explained = 96.1%
## -REML = 778.88  Scale est. = 1         n = 248
```

```
deep.numsp.chlorophyll <- gam(num.species ~ s(lat, lon, bs = "sos") + s(num.record
s) +  s(chlorophyll), data = deep, family = "nb", method = "REML", select = TRUE)
gam.check(deep.numsp.chlorophyll)
```

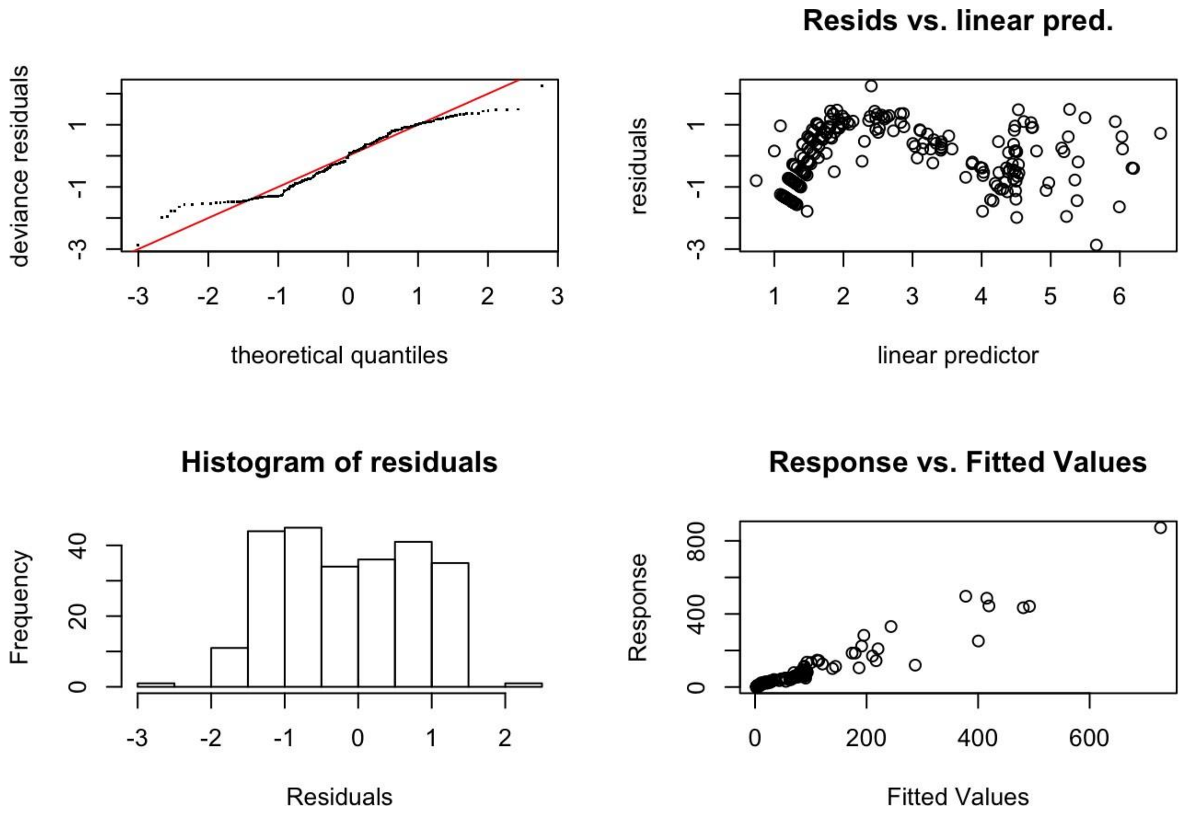


```
##
## Method: REML    Optimizer: outer newton
## full convergence after 14 iterations.
## Gradient range [-1.60812e-05,3.281463e-06]
## (score 783.3275 & scale 1).
## Hessian positive definite, eigenvalue range [1.238047e-05,26.20742].
## Model rank =  68 / 68
##
## Basis dimension (k) checking results. Low p-value (k-index<1) may
## indicate that k is too low, especially if edf is close to k'.
##
##                     k'      edf k-index p-value
## s(lat,lon)     4.90e+01 6.48e+00    0.97    0.40
## s(num.records) 9.00e+00 8.72e+00    0.21  <2e-16 ***
## s(chlorophyll) 9.00e+00 9.72e-05    0.99    0.54
## ---
## Signif. codes:  0 '***' 0.001 '**' 0.01 '*' 0.05 '.' 0.1 ' ' 1
```

```
summary(deep.numsp.chlorophyll)
```

```
##
## Family: Negative Binomial(15.245)
## Link function: log
##
## Formula:
## num.species ~ s(lat, lon, bs = "sos") +  s(num.records) +  s(chlorophyll)
##
## Parametric coefficients:
##                  Estimate Std. Error z value Pr(>|z|)
##  (Intercept)     2.5224      0.0294    85.81    <2e-16 ***
## ---
## Signif. codes:  0 '***' 0.001 '**' 0.01 '*' 0.05 '.' 0.1 ' ' 1
##
## Approximate significance of smooth terms:
##                          edf  Ref.df   Chi.sq   p-value
## s(lat,lon)        6.482e+00       49     28.61 4.42e-06 ***
## s(num.records) 8.721e+00        9  3057.11  <  2e-16  ***
##  s(chlorophyll)  9.717e-05        9 0.00  0.535
## ---
## Signif. codes:  0 '***' 0.001 '**' 0.01 '*' 0.05 '.' 0.1 ' ' 1
##
##  R-sq.(adj)  =   0.929    Deviance explained = 96.1%
## -REML = 783.33Scale  est. = 1                 n =  248
```

```
deep.numsp.current <- gam(num.species ~ s(lat, lon, bs = "sos") + s(num.records) +
s(current), data = deep, family = "nb", method = "REML", select = TRUE)
gam.check(deep.numsp.current)
```

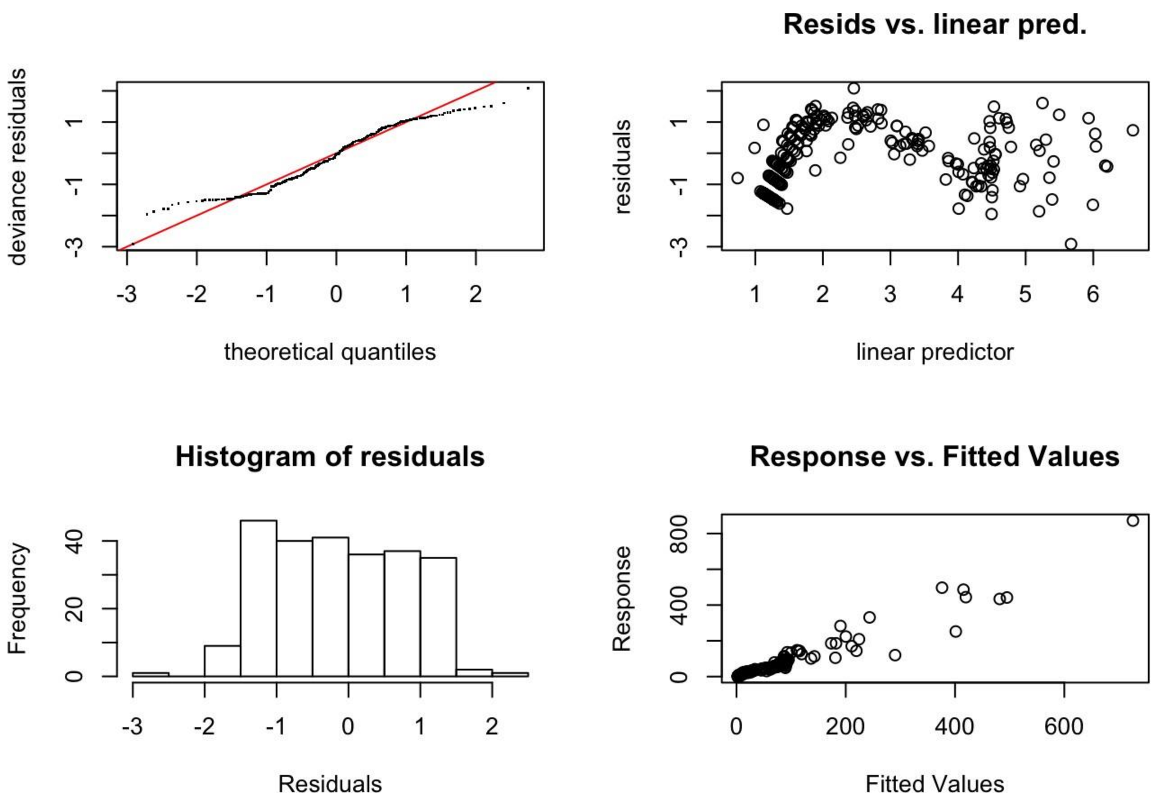

```
##
## Method: REML   Optimizer: outer newton
## full convergence after 12 iterations.
## Gradient range [-7.866149e-05,0.0006824098]
## (score 783.122 & scale 1).
## Hessian positive definite, eigenvalue range [3.296297e-05,26.16876].
## Model rank =  68 / 68
##
## Basis dimension (k) checking results. Low p-value (k-index<1) may
## indicate that k is too low, especially if edf is close to k'.
##
##                     k'    edf k-index p-value
## s(lat,lon)      49.000  6.353    0.97    0.35
## s(num.records)   9.000  8.720    0.21  <2e-16 ***
## s(current)       9.000  0.708    0.98    0.54
## ---
## Signif. codes:  0 '***' 0.001 '**' 0.01 '*' 0.05 '.' 0.1 ' ' 1
```

```
summary(deep.numsp.current)
```

```
##
## Family: Negative Binomial(15.378)
## Link function: log
##
## Formula:
## num.species ~ s(lat, lon, bs = "sos") + s(num.records) + s(current)
##
## Parametric coefficients:
##             Estimate Std. Error z value Pr(>|z|)
## (Intercept)  2.52214    0.02936   85.91   <2e-16 ***
## ---
## Signif. codes:  0 '***' 0.001 '**' 0.01 '*' 0.05 '.' 0.1 ' ' 1
##
## Approximate significance of smooth terms:
##                   edf Ref.df   Chi.sq  p-value
## s(lat,lon)     6.3530     49   28.396 3.48e-06 ***
## s(num.records) 8.7198      9 3072.017  < 2e-16 ***
## s(current)     0.7081      9    1.351    0.139
## ---
## Signif. codes:  0 '***' 0.001 '**' 0.01 '*' 0.05 '.' 0.1 ' ' 1
##
##  R-sq.(adj) =  0.928   Deviance explained = 96.2%
## -REML = 783.12  Scale est. = 1         n = 248
```

```
deep.numsp.salinity <- gam(num.species ~ s(lat, lon, bs = "sos") + s(num.records) +
s(salinity), data = deep, family = "nb", method = "REML", select = TRUE)
gam.check(deep.numsp.salinity)
```

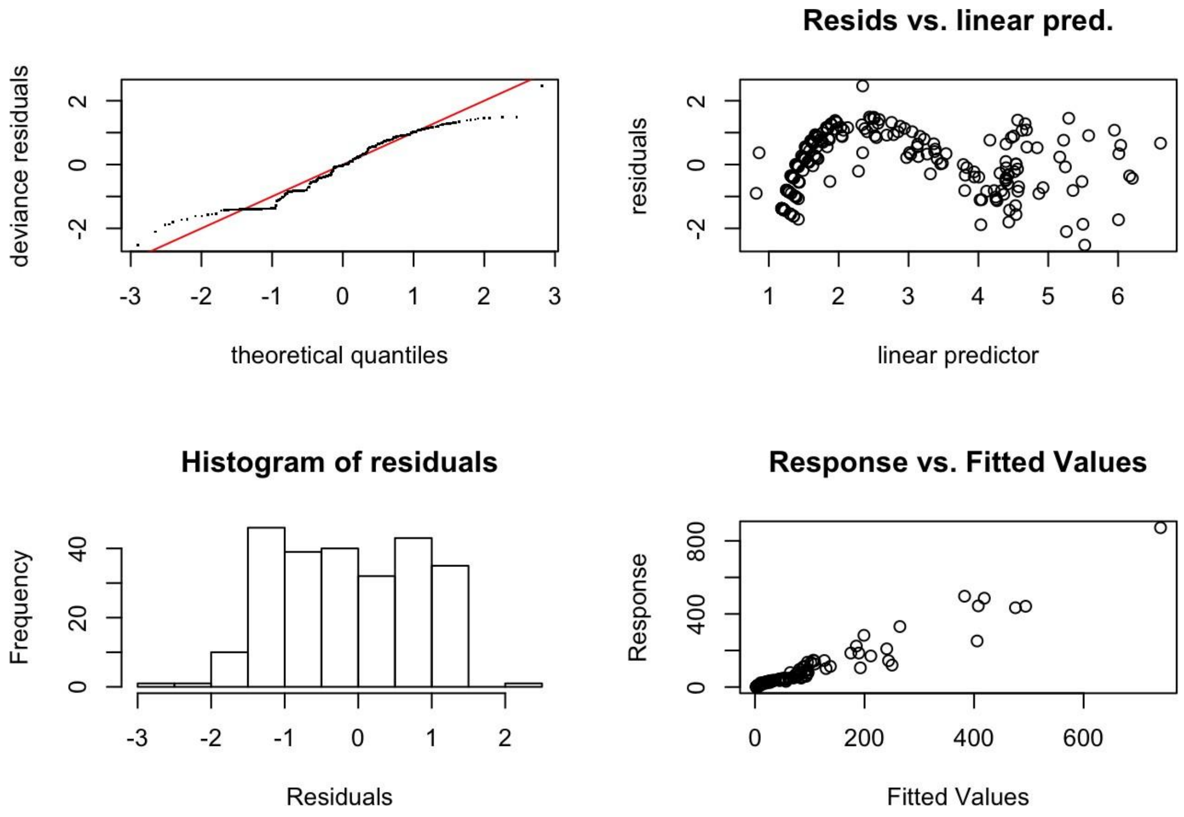


```
##
## Method: REML   Optimizer: outer newton
## full convergence after 19 iterations.
## Gradient range [-4.890114e-05,6.436646e-05]
## (score 778.6788 & scale 1).
## Hessian positive definite, eigenvalue range [4.896408e-05,26.32655].
## Model rank =  68 / 68
##
## Basis dimension (k) checking results. Low p-value (k-index<1) may
## indicate that k is too low, especially if edf is close to k'.
##
##                      k'     edf k-index p-value
## s(lat,lon)     49.00000 0.00062    0.95    0.32
## s(num.records)  9.00000 8.74747    0.19  <2e-16 ***
## s(salinity)     9.00000 2.76874    0.92    0.20
## ---
## Signif. codes:  0 '***' 0.001 '**' 0.01 '*' 0.05 '.' 0.1 ' ' 1
```

```
summary(deep.numsp.salinity)
```

```
##
## Family: Negative Binomial(16.098)
## Link function: log
##
## Formula:
## num.species ~ s(lat, lon, bs = "sos") + s(num.records) + s(salinity)
##
## Parametric coefficients:
##                Estimate Std. Error z value Pr(>|z|)
## (Intercept)    2.52442    0.02907   86.83   <2e-16 ***
## ---
## Signif. codes:  0 '***' 0.001 '**' 0.01 '*' 0.05 '.' 0.1 ' ' 1
##
## Approximate significance of smooth terms:
##                        edf Ref.df   Chi.sq  p-value
## s(lat,lon)       0.0006202     49    0.001   0.391
## s(num.records) 8.7474679      9 3390.383  < 2e-16 ***
## s(salinity)      2.7687403      9   35.507 6.42e-09 ***
## ---
## Signif. codes:  0 '***' 0.001 '**' 0.01 '*' 0.05 '.' 0.1 ' ' 1
##
## R-sq.(adj) =  0.936   Deviance explained = 96.2%
## -REML = 778.68  Scale est. = 1         n = 248
```

```
deep.numsp.nitrate <- gam(num.species ~ s(lat, lon, bs = "sos") + s(num.records) +
s(nitrate), data = deep, family = "nb", method = "REML", select = TRUE)
gam.check(deep.numsp.nitrate)
```

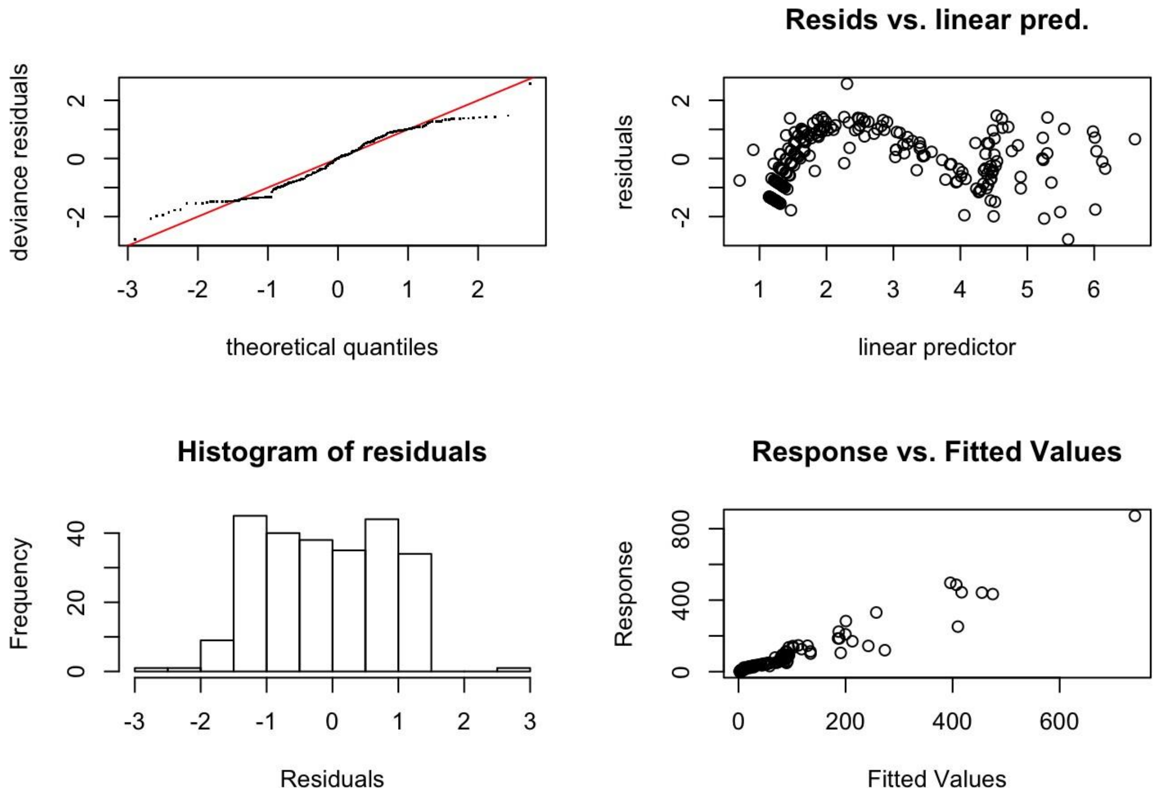

```
##
## Method: REML   Optimizer: outer newton
## full convergence after 15 iterations.
## Gradient range [-0.000171565,7.103026e-05]
## (score 778.9375 & scale 1).
## Hessian positive definite, eigenvalue range [0.000171518,26.49127].
## Model rank =  68 / 68
##
## Basis dimension (k) checking results. Low p-value (k-index<1) may
## indicate that k is too low, especially if edf is close to k'.
##
##                      k'   edf k-index p-value
## s(lat,lon)       49.000 2.016    0.96    0.28
## s(num.records)    9.000 8.742    0.22  <2e-16 ***
## s(nitrate)        9.000 0.946    0.93    0.22
## ---
## Signif. codes:  0 '***' 0.001 '**' 0.01 '*' 0.05 '.' 0.1 ' ' 1
```

```
summary(deep.numsp.nitrate)
```

```
##
## Family: Negative Binomial(15.952)
## Link function: log
##
## Formula:
## num.species ~ s(lat, lon, bs = "sos") + s(num.records) + s(nitrate)
##
## Parametric coefficients:
##             Estimate Std. Error z value Pr(>|z|)
## (Intercept)  2.52313    0.02915   86.56   <2e-16 ***
## ---
## Signif. codes:  0 '***' 0.001 '**' 0.01 '*' 0.05 '.' 0.1 ' ' 1
##
## Approximate significance of smooth terms:
##                   edf Ref.df   Chi.sq  p-value
## s(lat,lon)     2.0160     49    4.562   0.0495 *
## s(num.records) 8.7424      9 3467.571  < 2e-16 ***
## s(nitrate)     0.9461      9   17.430 1.87e-06 ***
## ---
## Signif. codes:  0 '***' 0.001 '**' 0.01 '*' 0.05 '.' 0.1 ' ' 1
##
## R-sq.(adj) =  0.935   Deviance explained = 96.2%
## -REML = 778.94  Scale est. = 1         n = 248
```

```
deep.numsp.env <- gam(num.species ~ s(lat, lon, bs = "sos") + s(num.records) + s(te
mp) + s(dissolved.oxygen) + s(chlorophyll) + s(current) + s(salinity) + s(nitrat e),
data = deep, family = "nb", method = "REML", select = TRUE)
gam.check(deep.numsp.env)
```

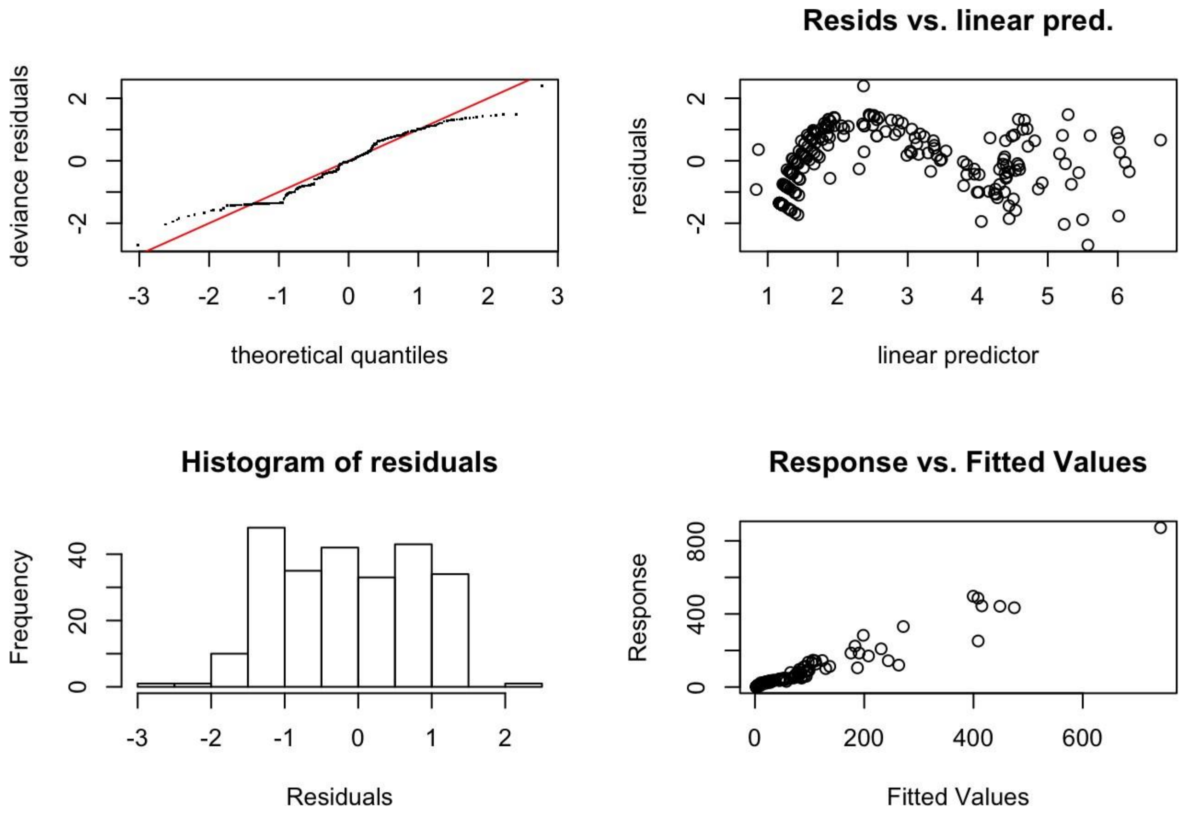


```
##
## Method: REML   Optimizer: outer newton
## full convergence after 13 iterations.
## Gradient range [-0.0003393487,0.0001655968]
## (score 778.3568 & scale 1).
## eigenvalue range [-3.960114e-05,26.01022].
## Model rank =  113 / 113
##
## Basis dimension (k) checking results. Low p-value (k-index<1) may
## indicate that k is too low, especially if edf is close to k'.
##
##                           k'      edf k-index p-value
## s(lat,lon)          4.90e+01 5.61e-01    0.96    0.33
## s(num.records)      9.00e+00 8.74e+00    0.21  <2e-16 ***
## s(temp)             9.00e+00 1.29e+00    0.99    0.51
## s(dissolved.oxygen) 9.00e+00 2.50e-04    0.99    0.55
## s(chlorophyll)      9.00e+00 2.23e-04    1.00    0.63
## s(current)          9.00e+00 1.33e-01    0.96    0.37
## s(salinity)         9.00e+00 2.34e+00    0.93    0.18
## s(nitrate)          9.00e+00 3.97e-04    0.94    0.30
## ---
## Signif. codes:  0 '***' 0.001 '**' 0.01 '*' 0.05 '.' 0.1 ' ' 1
```

```
summary(deep.numsp.env)
```

```
##
## Family: Negative Binomial(16.363)
## Link function: log
##
## Formula:
## num.species ~ s(lat, lon, bs = "sos") + s(num.records) + s(temp) +
##     s(dissolved.oxygen) + s(chlorophyll) +  s(current)  +  s(salinity)  +
##     s(nitrate)
##
## Parametric coefficients:
##             Estimate Std. Error z value Pr(>|z|)
##  (Intercept)   2.52257    0.02904   86.86   <2e-16 ***
## ---
## Signif. codes:  0 '***' 0.001 '**' 0.01 '*' 0.05 '.' 0.1 ' ' 1
##
## Approximate significance of smooth terms:
##                          edf  Ref.df    Chi.sq   p-value
##  s(lat,lon)            0.5611688      49     0.730  0.206615
##  s(num.records)        8.7441673       9  3279.706   < 2e-16  ***
##  s(temp)               1.2898582       9     3.262  0.048569  *
##  s(dissolved.oxygen) 0.0002496        9     0.000  0.479536
##  s(chlorophyll)        0.0002232       9     0.000  0.751210
##  s(current)            0.1331032       9     0.150  0.277828
##  s(salinity)           2.3436500       9    12.671  0.000285  ***
##  s(nitrate)            0.0003972       9     0.000  0.507870
##  ---
## Signif. codes:  0 '***' 0.001 '**' 0.01 '*' 0.05 '.' 0.1 ' ' 1
##
##  R-sq.(adj) =   0.938    Deviance explained = 96.3%
## -REML = 778.36Scale  est. =  1              n =  248
```

Model selection for number of species, deep water

```
deep.numsp.models <- list(deep.numsp.intercept = deep.numsp.intercept,
                          deep.numsp.numrec = deep.numsp.numrec,
                       deep.numsp.latlon = deep.numsp.latlon,
                       deep.numsp.temp = deep.numsp.temp,
                       deep.numsp.oxygen = deep.numsp.oxygen,
                       deep.numsp.chlorophyll = deep.numsp.chlorophyll,
                       deep.numsp.current = deep.numsp.current,
                       deep.numsp.salinity = deep.numsp.salinity,
                       deep.numsp.nitrate = deep.numsp.nitrate,
                       deep.numsp.env = deep.numsp.env)
deep.numsp.aic.df <- data.frame(Model = names(deep.numsp.models),
                              AIC = sapply(deep.numsp.models, function(x) x$aic),
                             akaike.weights(sapply(deep.numsp.models, function(x) x
$aic)))

deep.numsp.aic.df <- deep.numsp.aic.df[order(deep.numsp.aic.df$AIC),]
deep.numsp.aic.df$Cumulative.Weight <- cumsum(deep.numsp.aic.df$weights)

kable(deep.numsp.aic.df, row.names = FALSE)
```

Model	AIC	deltaAIC	rel.LL	weights	Cumulative.Weight
deep.numsp.env	1503.752	0.0000000	1.0000000	0.3916404	0.3916404
deep.numsp.salinity	1504.519	0.7670844	0.6814433	0.2668807	0.6585211
deep.numsp.temp	1505.273	1.5213809	0.4673436	0.1830306	0.8415517
deep.numsp.oxygen	1507.051	3.2996823	0.1920804	0.0752264	0.9167781
deep.numsp.nitrate	1507.071	3.3192946	0.1902061	0.0744924	0.9912705
deep.numsp.current	1513.226	9.4747427	0.0087616	0.0034314	0.9947019
deep.numsp.latlon	1513.744	9.9923088	0.0067639	0.0026490	0.9973510
deep.numsp.chlorophyll	1513.744	9.9924113	0.0067636	0.0026489	0.9999998
deep.numsp.numrec	1533.111	29.3590199	0.0000004	0.0000002	1.0000000
deep.numsp.intercept	2230.235	726.4832924	0.0000000	0.0000000	1.0000000

```
write.csv(deep.numsp.aic.df, file = "deep.numsp.aic.csv")
```

We find that the best model is the one that contains all environmental predictor variables. The second best model is one that contains only the effects of oxygen, but the delta AIC between that model and the best model is 7.18, indicating that the oxygen-only model is a relatively poor fit to the data. We find strong evidence that the “env” model outperforms the model using spatial autocorrelation alone (delta AIC = 10.18).

Plots for number of species, deep water

```
qplot(deep$temp, predict(deep.numsp.temp, deep)) + geom_smooth(method = "gam")
```

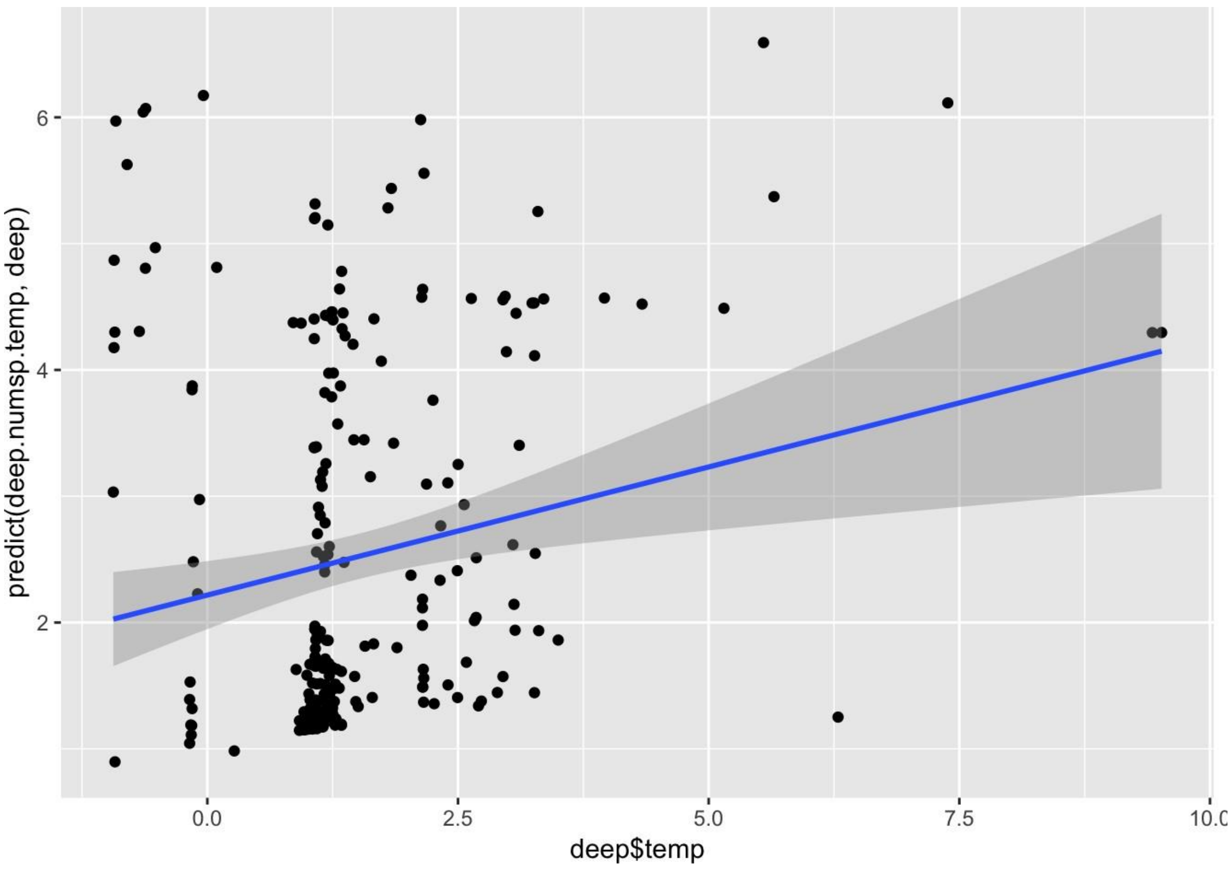


```
qplot(deep$dissolved.oxygen, predict(deep.numsp.oxygen, deep)) + geom_smooth(method
= "gam")
```

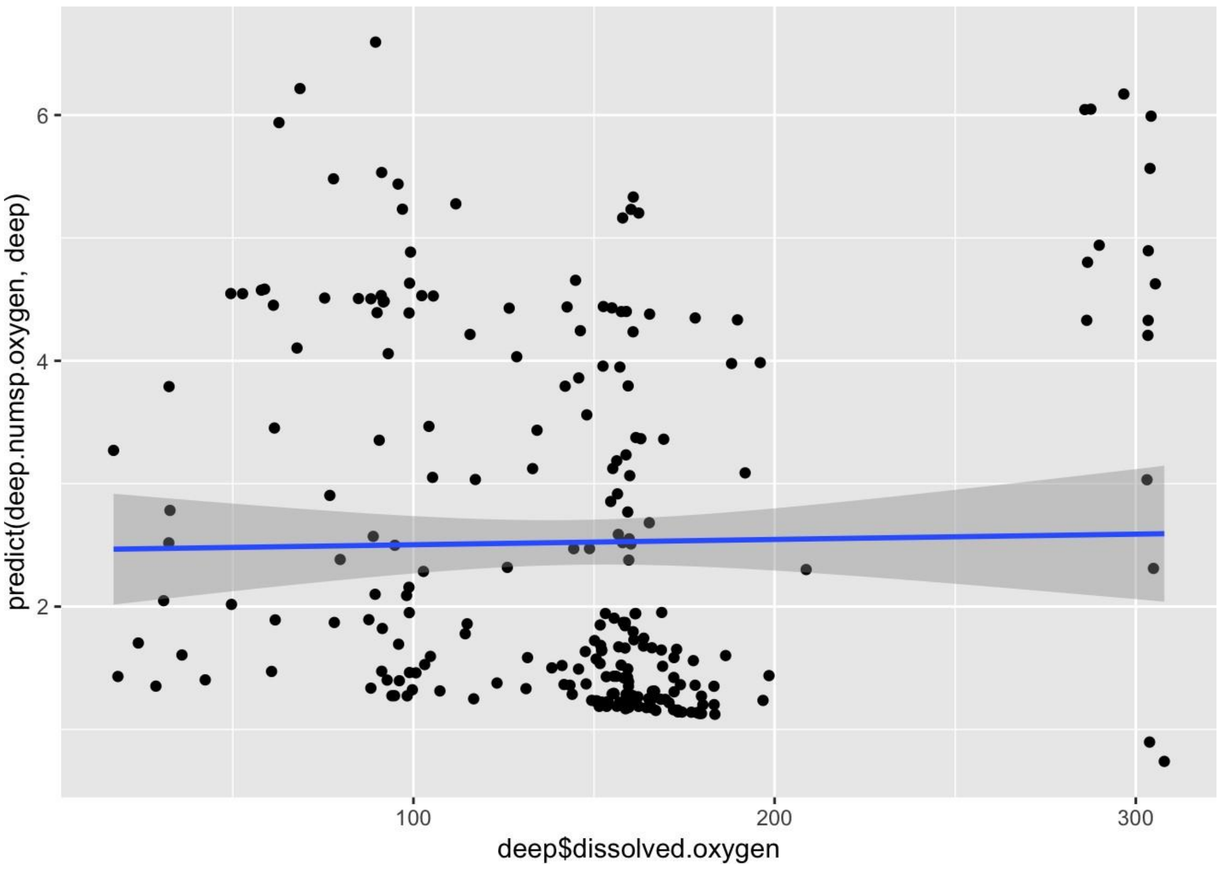


```
qplot(deep$chlorophyll, predict(deep.numsp.chlorophyll, deep)) + geom_smooth(method
= "gam")
```

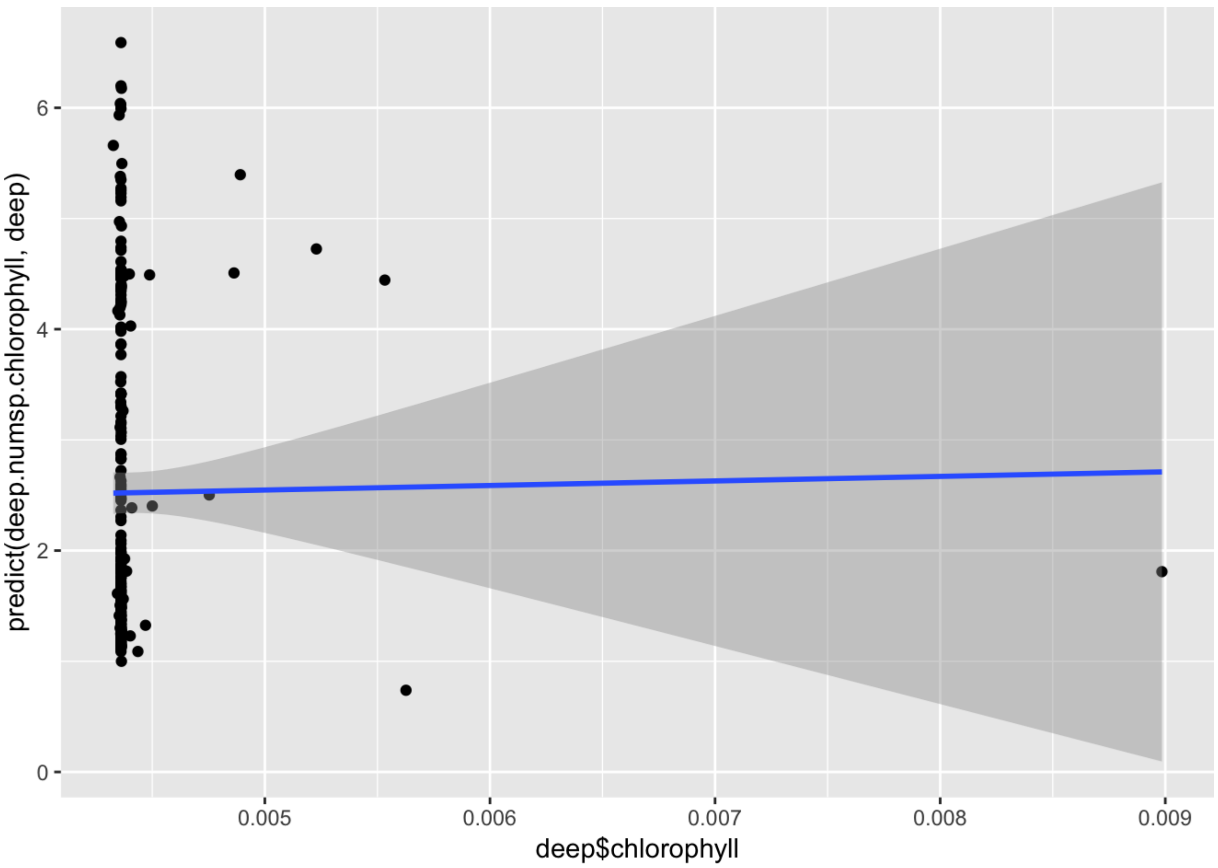


```
qplot(deep$current, predict(deep.numsp.current, deep)) + geom_smooth(method = "gam"
)
```

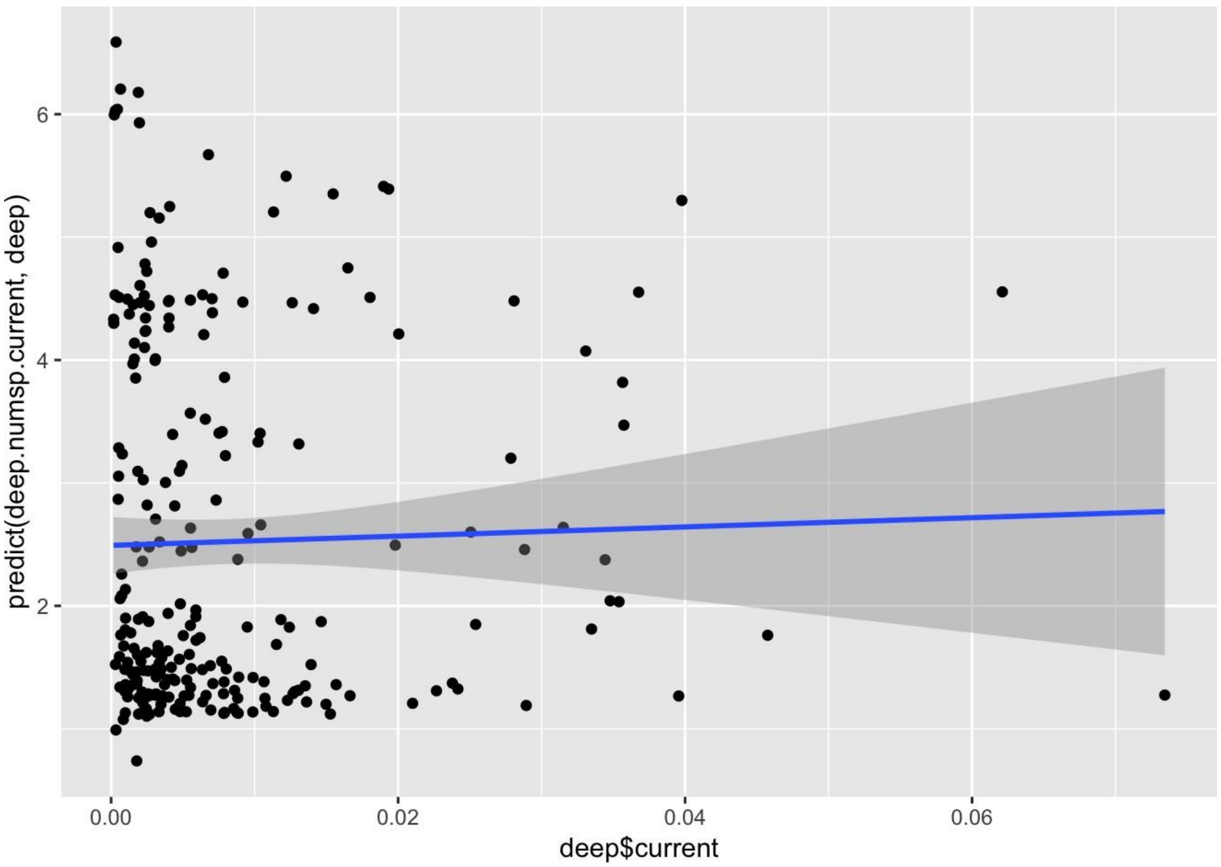


```
qplot(deep$salinity, predict(deep.numsp.salinity, deep)) + geom_smooth(method  =  "ga
m")
```

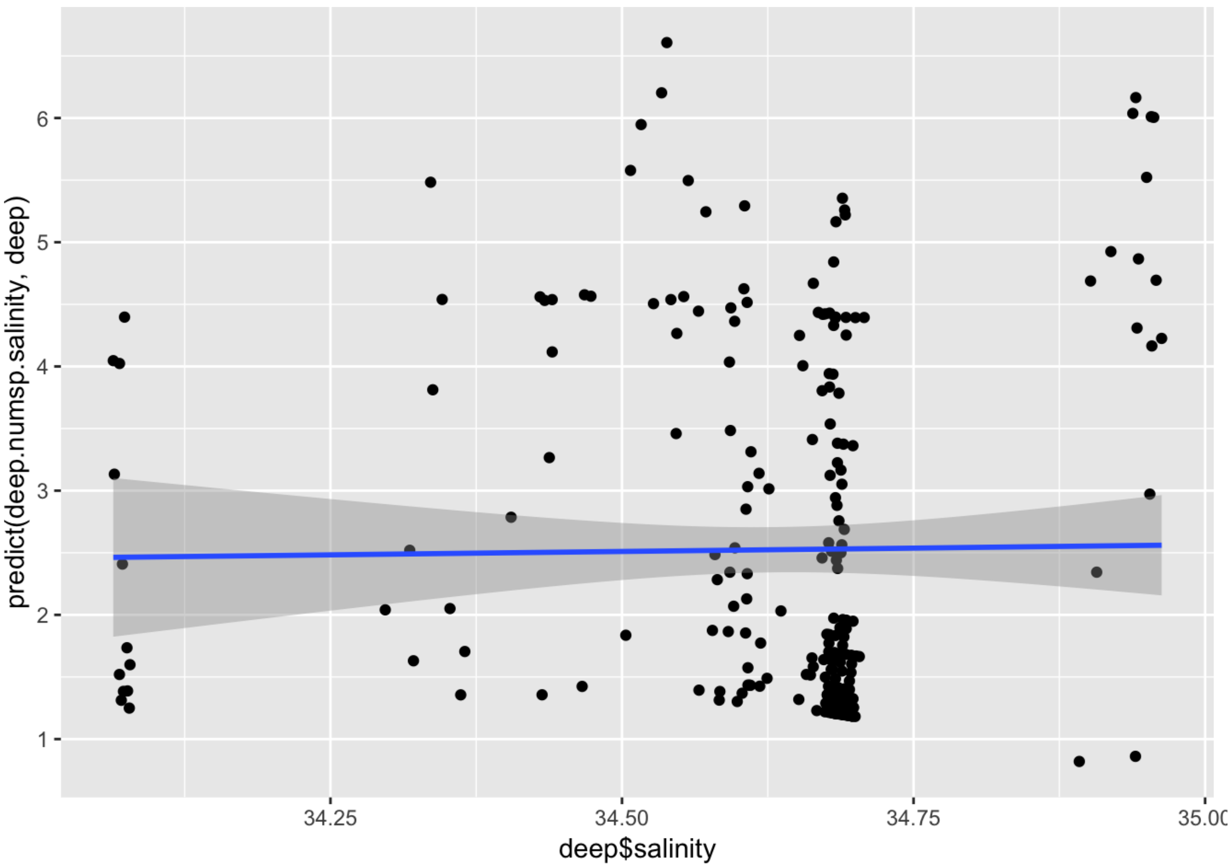


```
qplot(deep$nitrate, predict(deep.numsp.nitrate, deep)) + geom_smooth(method = "gam"
)
```

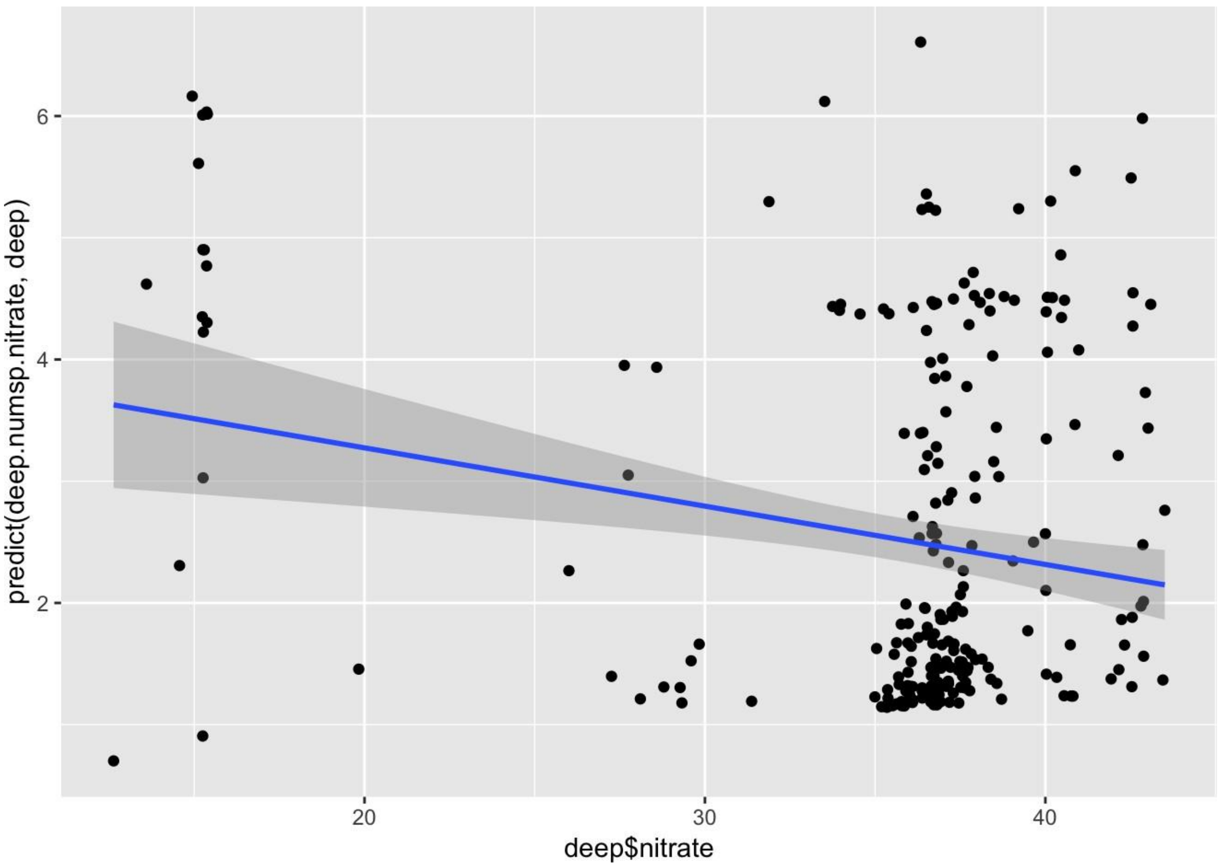

GAMs for ES50, deep water

We're now doing GAMs for the effects of the environment on the number of species estimated from rarefaction analyses. As above, we remove the "no.records" term because it is already included in the calculation of ES50.

```
deep.es50.intercept <- gam(ES50 ~ 1, data = deep, family = "nb", method = "REML", s
elect = TRUE)
gam.check(deep.es50.intercept)
```

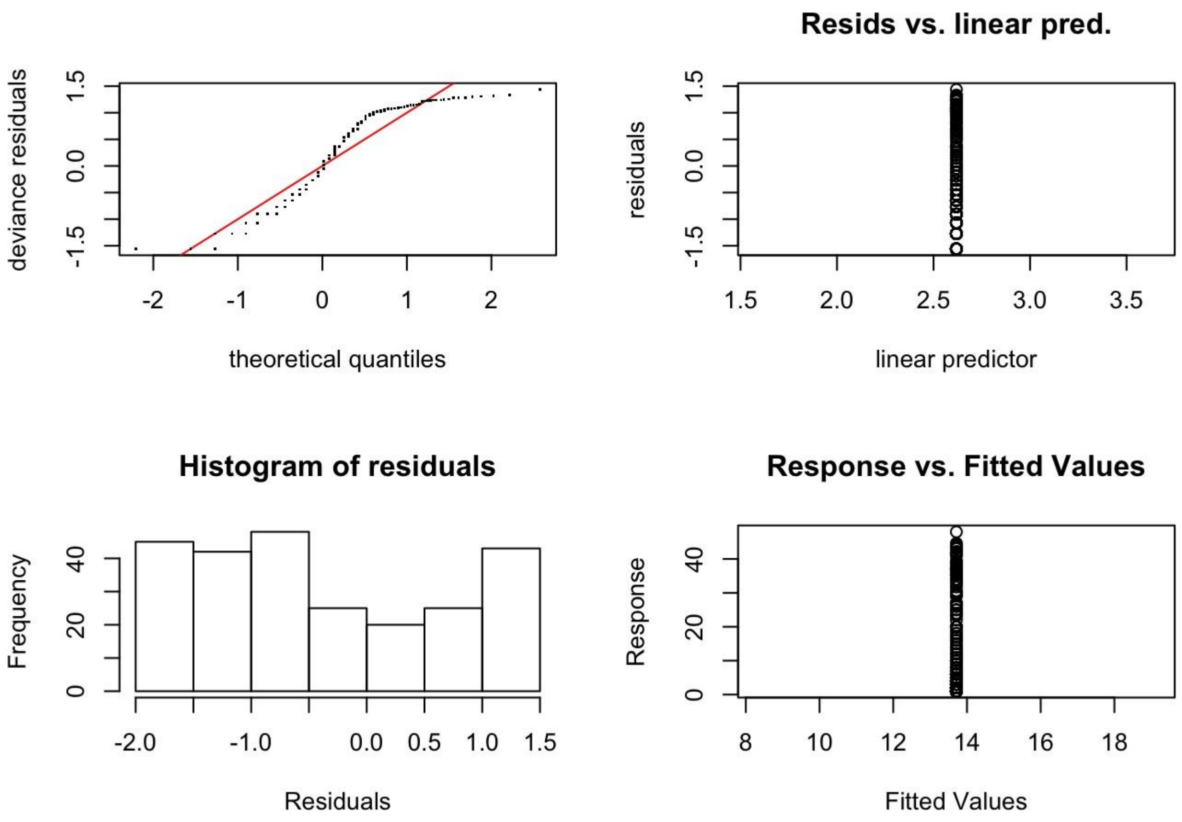


```
##
## Method: REML   Optimizer: outer newton
## full convergence after 3 iterations.
## Gradient range [2.716585e-10,2.716585e-10]
## (score 906.5347 & scale 1).
## Hessian positive definite, eigenvalue range [130.3561,130.3561].
## Model rank =  1 / 1
```

```
summary(deep.es50.intercept)
```

```
##
## Family: Negative Binomial(0.863)
## Link function: log
##
##   Formula:
## ES50 ~ 1
##
## Parametric coefficients:
##                Estimate Std. Error z value Pr(>|z|)
## (Intercept)    2.61829    0.07046   37.16  <2e-16 ***
## ---
## Signif. codes:   0 '***' 0.001 '**' 0.01 '*' 0.05 '.' 0.1 ' ' 1
##
##
## R-sq.(adj) =        0    Deviance explained = 1.93e-08%
## -REML = 906.53   Scale est. = 1          n = 248
```

```
deep.es50.latlon <- gam(ES50 ~ s(lat, lon, bs = "sos") , data = deep, family = "nb"
, method = "REML", select = TRUE)
gam.check(deep.es50.latlon)
```

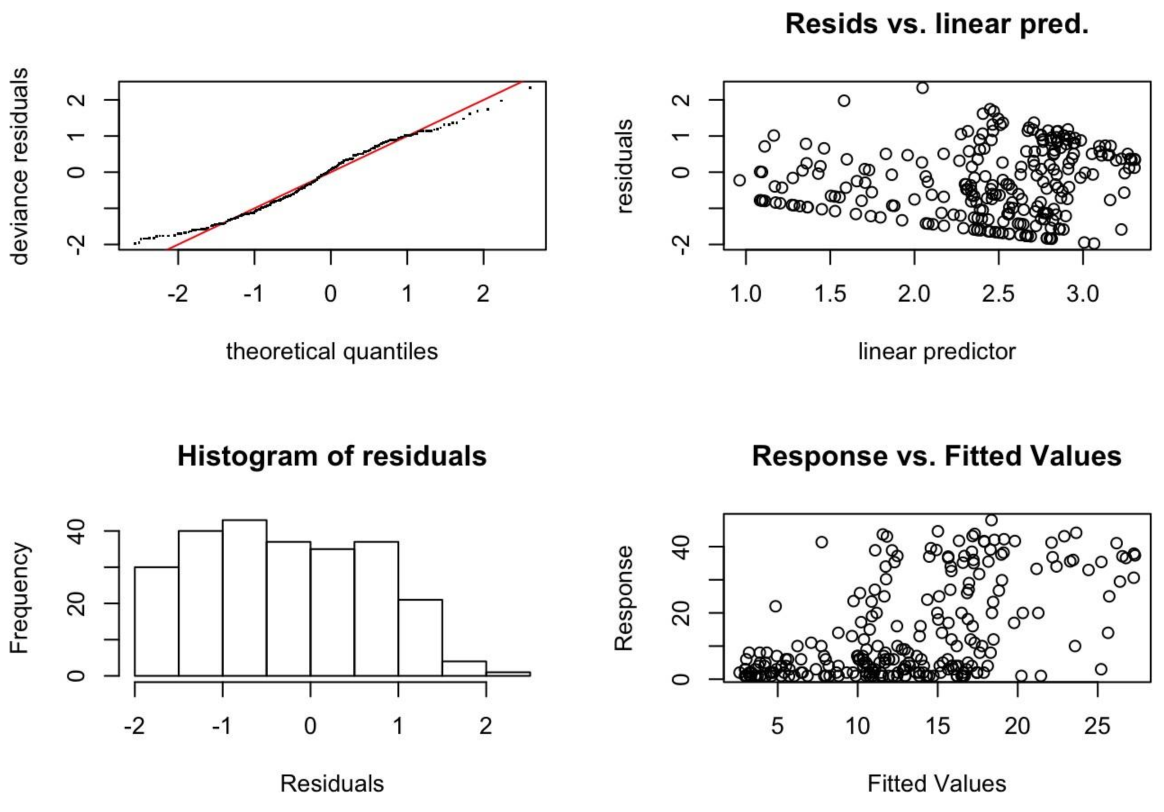

```
##
## Method: REML   Optimizer: outer newton
## full convergence after 4 iterations.
## Gradient range [-1.143111e-06,4.094961e-07]
## (score 889.619 & scale 1).
## Hessian positive definite, eigenvalue range [2.532821,104.0371].
## Model rank =  50 / 50
##
## Basis dimension (k) checking results. Low p-value (k-index<1) may
## indicate that k is too low, especially if edf is close to k'.
##
##              k' edf k-index p-value
## s(lat,lon)  49  17    0.86    0.13
```

```
summary(deep.es50.latlon)
```

```
##
## Family: Negative Binomial(1.121)
## Link function: log
##
## Formula:
## ES50 ~ s(lat, lon, bs = "sos")
##
## Parametric coefficients:
##             Estimate Std. Error z value Pr(>|z|)
## (Intercept)  2.43192    0.06339   38.36   <2e-16 ***
## ---
## Signif. codes:  0 '***' 0.001 '**' 0.01 '*' 0.05 '.' 0.1 ' ' 1
##
## Approximate significance of smooth terms:
##              edf Ref.df Chi.sq  p-value
## s(lat,lon) 17.04     49  89.19 1.66e-15 ***
## ---
## Signif. codes:  0 '***' 0.001 '**' 0.01 '*' 0.05 '.' 0.1 ' ' 1
##
## R-sq.(adj) =  0.229   Deviance explained =   28%
## -REML = 889.62  Scale est. = 1         n = 248
```

```
deep.es50.temp <- gam(ES50 ~ s(lat, lon, bs = "sos") + s(temp), data = deep, famil y
= "nb", method = "REML", select = TRUE)
gam.check(deep.es50.temp)
```

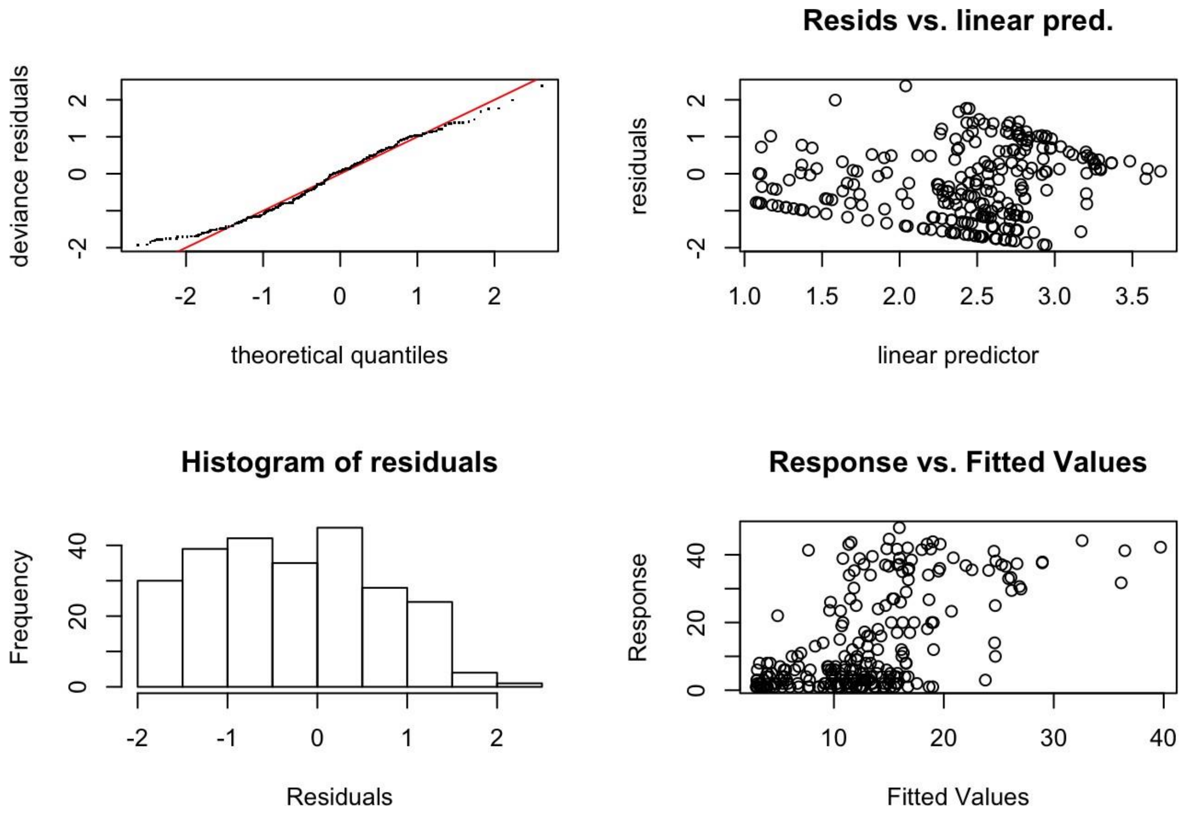


```
##
## Method: REML   Optimizer: outer newton
## full convergence after 9 iterations.
## Gradient range [-0.0004680909,0.0003360787]
## (score 887.7525 & scale 1).
## Hessian positive definite, eigenvalue range [0.0004680402,102.684].
## Model rank =  59 / 59
##
## Basis dimension (k) checking results. Low p-value (k-index<1) may
## indicate that k is too low, especially if edf is close to k'.
##
##                 k'    edf k-index p-value
## s(lat,lon) 49.000 16.799    0.89    0.34
## s(temp)     9.000  0.847    0.77    0.01 **
## ---
## Signif. codes:  0 '***' 0.001 '**' 0.01 '*' 0.05 '.' 0.1 ' ' 1
```

```
summary(deep.es50.temp)
```

```
##
## Family: Negative Binomial(1.146)
## Link function: log
##
## Formula:
## ES50 ~ s(lat, lon, bs = "sos") + s(temp)
##
## Parametric coefficients:
##              Estimate Std. Error z value Pr(>|z|)
## (Intercept)    2.4218     0.0628   38.56   <2e-16 ***
## ---
## Signif. codes:  0 '***' 0.001 '**' 0.01 '*' 0.05 '.' 0.1 ' ' 1
##
## Approximate significance of smooth terms:
##                 edf Ref.df Chi.sq  p-value
## s(lat,lon) 16.7991     49 89.108 9.68e-16***
## s(temp)     0.8467      9  5.169  0.00824 **
## ---
## Signif. codes:  0 '***' 0.001 '**' 0.01 '*' 0.05 '.' 0.1 ' ' 1
##
##  R-sq.(adj) =  0.278   Deviance explained=29.6%
## -REML=887.75Scale est. = 1          n = 248
```

```
deep.es50.oxygen <- gam(ES50 ~ s(lat, lon, bs= "sos") + s(dissolved.oxygen), dat a = deep, family = "nb", method = "REML", select = TRUE) gam.check(deep.es50.oxygen)
```

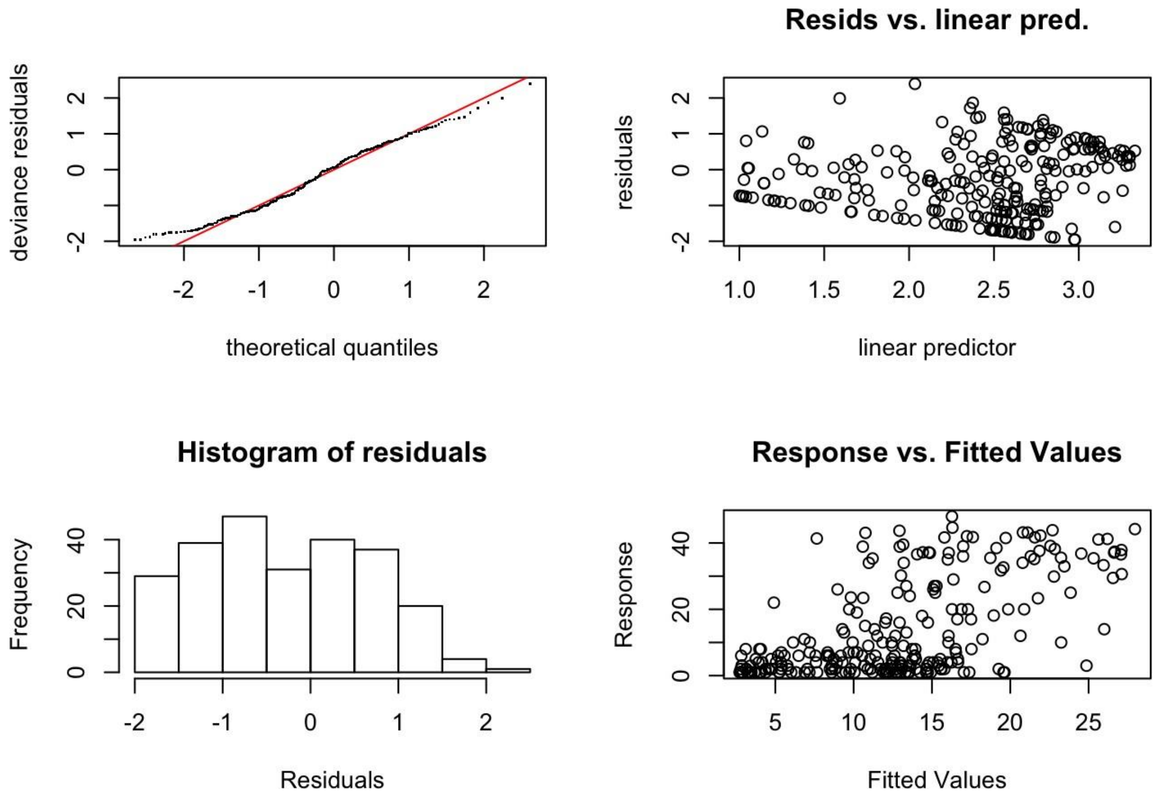

```
##
## Method: REML   Optimizer: outer newton
## full convergence after 4 iterations.
## Gradient range [-0.0001959342,0.0001265859]
## (score 888.3044 & scale 1).
## Hessian positive definite, eigenvalue range [0.0001958078,100.738].
## Model rank =  59 / 59
##
## Basis dimension (k) checking results. Low p-value (k-index<1) may
## indicate that k is too low, especially if edf is close to k'.
##
##                         k'   edf k-index p-value
## s(lat,lon)           49.00 18.08    0.90    0.32
## s(dissolved.oxygen)   9.00  1.28    0.94    0.67
```

```
summary(deep.es50.oxygen)
```

```
##
## Family: Negative Binomial(1.159)
## Link function: log
##
## Formula:
## ES50 ~ s(lat, lon, bs = "sos") + s(dissolved.oxygen)
##
## Parametric coefficients:
##             Estimate Std. Error z value Pr(>|z|)
## (Intercept)  2.41289    0.06254   38.58   <2e-16 ***
## ---
## Signif. codes:  0 '***' 0.001 '**' 0.01 '*' 0.05 '.' 0.1 ' ' 1
##
## Approximate significance of smooth terms:
##                        edf Ref.df Chi.sq  p-value
## s(lat,lon)          18.080     49 82.598 2.87e-14 ***
## s(dissolved.oxygen)  1.282      9  4.827   0.0105 *
## ---
## Signif. codes:  0 '***' 0.001 '**' 0.01 '*' 0.05 '.' 0.1 ' ' 1
##
## R-sq.(adj) =  0.276   Deviance explained = 30.9%
## -REML =  888.3  Scale est. = 1         n = 248
```

```
deep.es50.chlorophyll <- gam(ES50 ~ s(lat, lon, bs = "sos") + s(chlorophyll), dat a
= deep, family = "nb", method = "REML", select = TRUE)
gam.check(deep.es50.chlorophyll)
```

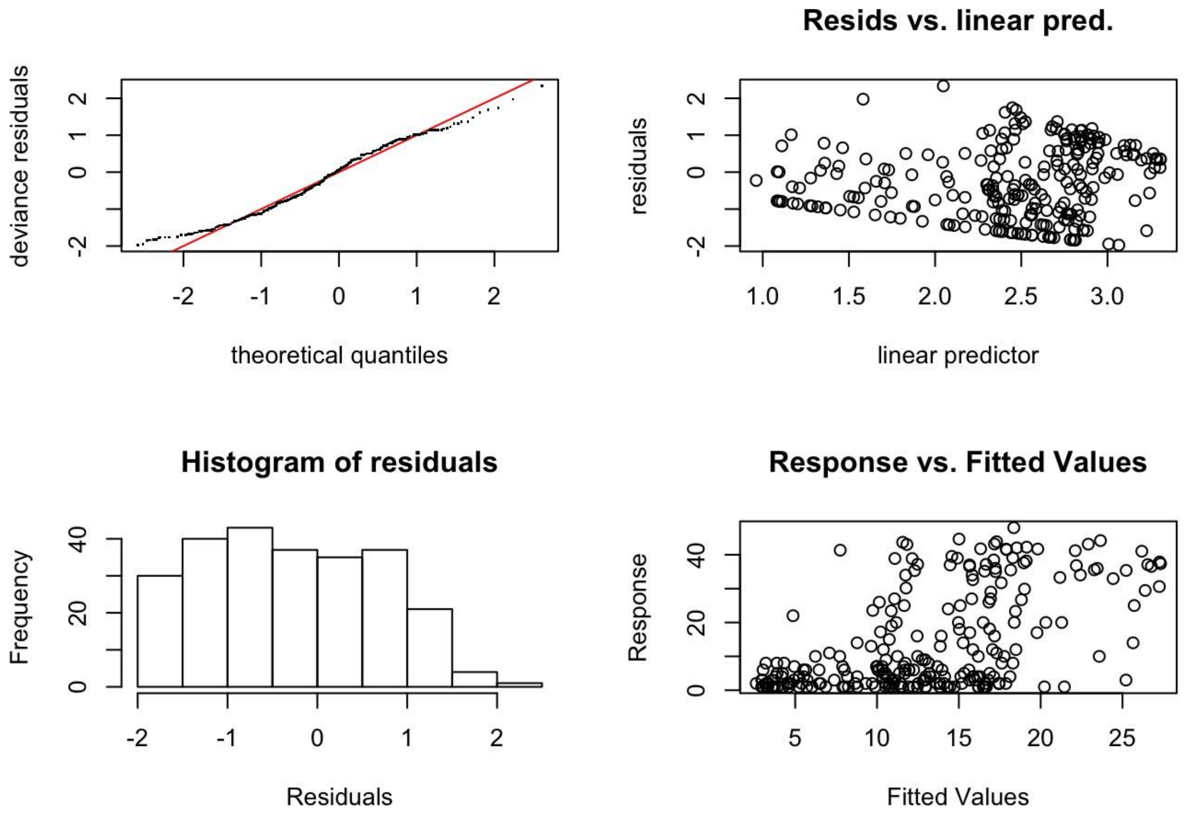


```
##
## Method: REML   Optimizer: outer newton
## full convergence after 6 iterations.
## Gradient range [-0.0003322345,0.0002701815]
## (score 889.6193 & scale 1).
## Hessian positive definite, eigenvalue range [2.314382e-05,104.0372].
## Model rank =  59 / 59
##
## Basis dimension (k) checking results. Low p-value (k-index<1) may
## indicate that k is too low, especially if edf is close to k'.
##
##                      k'      edf k-index p-value
## s(lat,lon)      4.90e+01 1.70e+01    0.86    0.12
## s(chlorophyll) 9.00e+00 9.76e-04    0.94    0.71
```

```
summary(deep.es50.chlorophyll)
```

```
##
## Family: Negative Binomial(1.121)
## Link function: log
##
## Formula:
## ES50 ~ s(lat, lon, bs = "sos") + s(chlorophyll)
##
## Parametric coefficients:
##              Estimate Std. Error z value Pr(>|z|)
## (Intercept)   2.43192    0.06339   38.36   <2e-16 ***
## ---
## Signif. codes:  0 '***' 0.001 '**' 0.01 '*' 0.05 '.' 0.1 ' ' 1
##
## Approximate significance of smooth terms:
##                      edf Ref.df Chi.sq  p-value
## s(lat,lon)     1.704e+01     49  89.18 1.66e-15 ***
## s(chlorophyll) 9.758e-04      9   0.00    0.824
## ---
## Signif. codes:  0 '***' 0.001 '**' 0.01 '*' 0.05 '.' 0.1 ' ' 1
##
## R-sq.(adj) =  0.229   Deviance explained =   28%
## -REML=889.62Scale est. = 1          n = 248
```

```
deep.es50.current <- gam(ES50 ~ s(lat, lon, bs = "sos") + s(current), data = dee
p, family = "nb", method = "REML", select = TRUE)
gam.check(deep.es50.current)
```

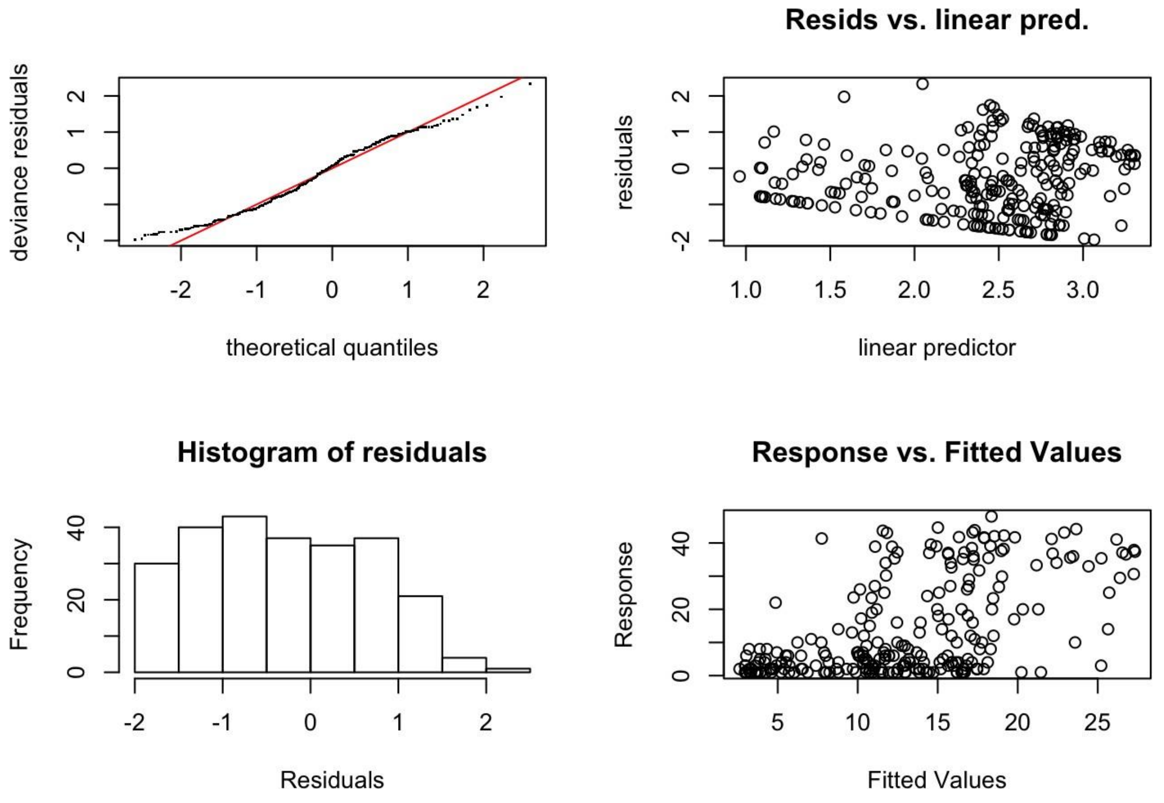

```
##
## Method: REML   Optimizer: outer newton
## full convergence after 7 iterations.
## Gradient range [-0.0004904038,0.0002815296]
## (score 889.6195 & scale 1).
## Hessian positive definite, eigenvalue range [5.346121e-06,104.0371].
## Model rank =  59 / 59
##
## Basis dimension (k) checking results. Low p-value (k-index<1) may
## indicate that k is too low, especially if edf is close to k'.
##
##                  k'      edf k-index p-value
## s(lat,lon) 49.00000 17.03618    0.86   0.145
## s(current)  9.00000  0.00136    0.83   0.085 .
## ---
## Signif. codes:  0 '***' 0.001 '**' 0.01 '*' 0.05 '.' 0.1 ' ' 1
```

```
summary(deep.es50.current)
```

```
##
## Family: Negative Binomial(1.121)
## Link function: log
##
## Formula:
## ES50 ~ s(lat, lon, bs = "sos") + s(current)
##
## Parametric coefficients:
##             Estimate Std. Error z value Pr(>|z|)
## (Intercept)  2.43192    0.06339   38.36   <2e-16 ***
## ---
## Signif. codes:  0 '***' 0.001 '**' 0.01 '*' 0.05 '.' 0.1 ' ' 1
##
## Approximate significance of smooth terms:
##                  edf Ref.df Chi.sq  p-value
## s(lat,lon) 17.036183     49  89.18 1.66e-15 ***
## s(current)  0.001356      9   0.00     0.72
## ---
## Signif. codes:  0 '***' 0.001 '**' 0.01 '*' 0.05 '.' 0.1 ' ' 1
##
## R-sq.(adj) =  0.229   Deviance explained =   28%
## -REML = 889.62  Scale est. = 1         n = 248
```

```
deep.es50.salinity <- gam(ES50 ~ s(lat, lon, bs = "sos") + s(salinity), data = de
ep, family = "nb", method = "REML", select = TRUE)
gam.check(deep.es50.salinity)
```

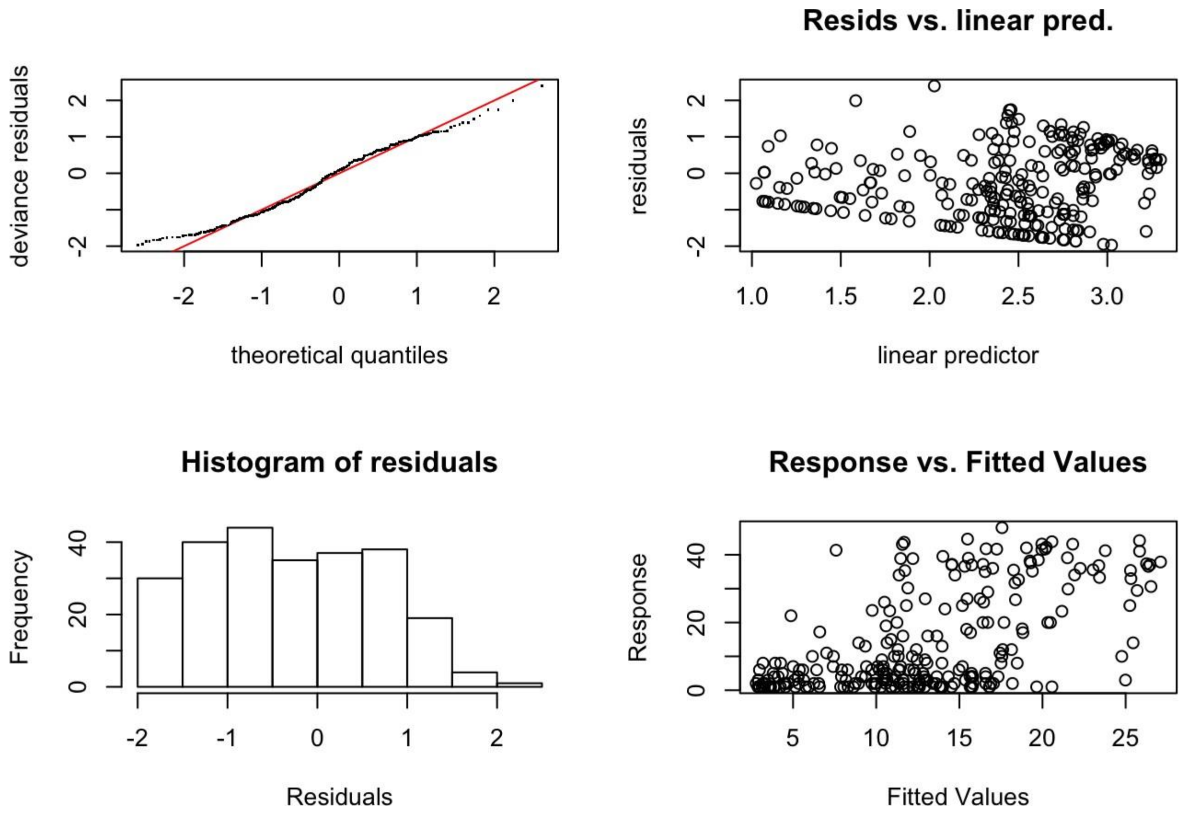


```
##
## Method: REML   Optimizer: outer newton
## full convergence after 4 iterations.
## Gradient range [-0.0004229958,0.0001480549]
## (score 889.1231 & scale 1).
## Hessian positive definite, eigenvalue range [5.59459e-05,101.5801].
## Model rank =  59 / 59
##
## Basis dimension (k) checking results. Low p-value (k-index<1) may
## indicate that k is too low, especially if edf is close to k'.
##
##                   k'   edf k-index p-value
## s(lat,lon)     49.00 17.11    0.88    0.24
## s(salinity)     9.00  1.62    0.93    0.59
```

```
summary(deep.es50.salinity)
```

```
##
## Family: Negative Binomial(1.145)
## Link function: log
##
## Formula:
## ES50 ~ s(lat, lon, bs = "sos") + s(salinity)
##
## Parametric coefficients:
##              Estimate Std. Error z value Pr(>|z|)
## (Intercept)   2.41959    0.06284    38.5   <2e-16 ***
## ---
## Signif. codes:  0 '***' 0.001 '**' 0.01 '*' 0.05 '.' 0.1 ' ' 1
##
## Approximate significance of smooth terms:
##                edf Ref.df Chi.sq  p-value
## s(lat,lon)   17.11     49 86.501 1.16e-15 ***
## s(salinity)   1.62      9  4.004   0.0373 *
## ---
## Signif. codes:  0 '***' 0.001 '**' 0.01 '*' 0.05 '.' 0.1 ' ' 1
##
## R-sq.(adj) =  0.261   Deviance explained = 29.9%
## -REML = 889.12  Scale est. = 1         n = 248
```

```
deep.es50.nitrate <- gam(ES50 ~ s(lat, lon, bs = "sos") + s(nitrate), data = dee
p, family = "nb", method = "REML", select = TRUE)
gam.check(deep.es50.nitrate)
```

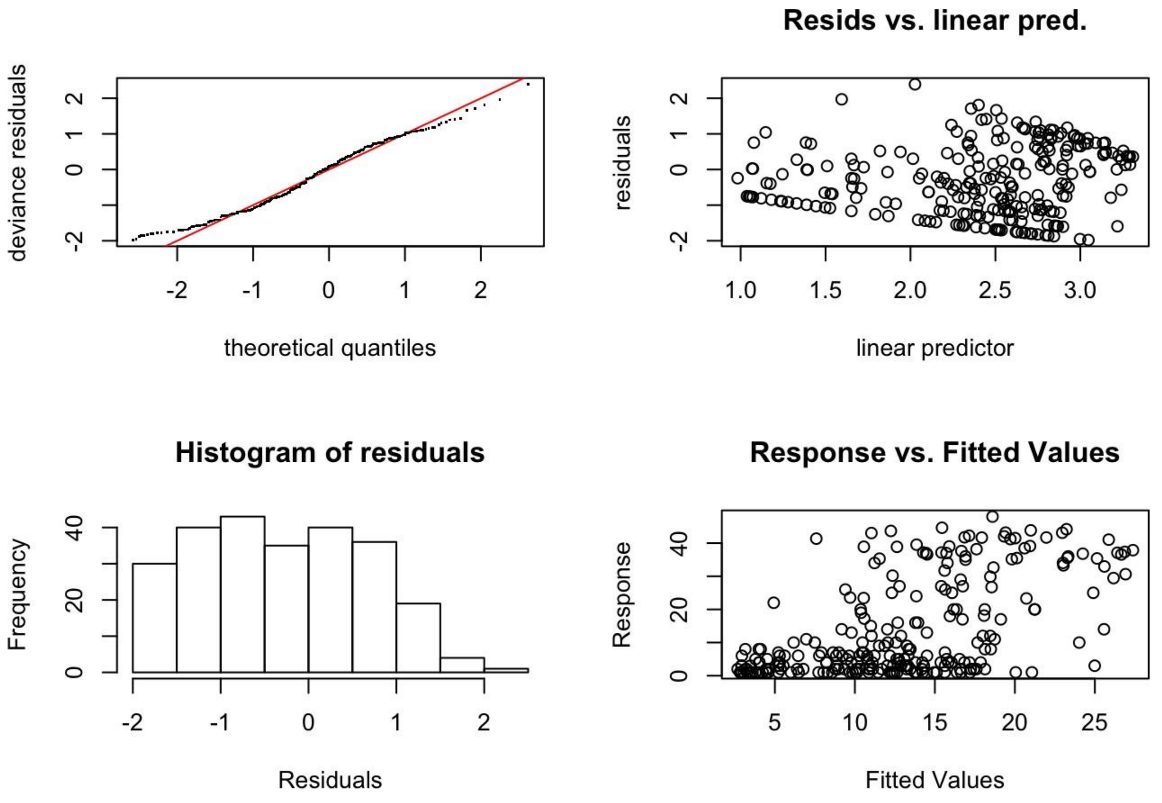

```
##
## Method: REML   Optimizer: outer newton
## full convergence after 4 iterations.
## Gradient range [-0.0001644731,7.83587e-05]
## (score 889.0222 & scale 1).
## Hessian positive definite, eigenvalue range [0.0001644127,102.0879].
## Model rank =  59 / 59
##
## Basis dimension (k) checking results. Low p-value (k-index<1) may
## indicate that k is too low, especially if edf is close to k'.
##
##                 k'   edf k-index p-value
##  s(lat,lon) 49.00 17.34    0.88    0.19
##  s(nitrate)  9.00  1.15    0.85    0.10
```

```
summary(deep.es50.nitrate)
```

```
##
## Family: Negative Binomial(1.141)
## Link function: log
##
## Formula:
## ES50 ~ s(lat, lon, bs = "sos") + s(nitrate)
##
## Parametric coefficients:
##             Estimate Std. Error z value Pr(>|z|)
##  (Intercept)  2.42141    0.06295   38.46   <2e-16 ***
## ---
## Signif. codes:  0 '***' 0.001 '**' 0.01 '*' 0.05 '.' 0.1 ' ' 1
##
## Approximate significance of smooth terms:
##               edf Ref.df Chi.sq  p-value
## s(lat,lon) 17.344     49 81.402 2.79e-14 ***
## s(nitrate)  1.149      9  2.899    0.041 *
## ---
## Signif. codes:  0 '***' 0.001 '**' 0.01 '*' 0.05 '.' 0.1 ' ' 1
##
##  R-sq.(adj) =   0.25   Deviance explained = 29.6%
## -REML = 889.02  Scale est. = 1         n = 248
```

```
deep.es50.env <- gam(ES50 ~ s(lat, lon, bs = "sos") + s(temp) + s(dissolved.oxyge
n) + s(chlorophyll) + s(current) + s(salinity) + s(nitrate), data = deep, family =
"nb", method = "REML", select = TRUE)
gam.check(deep.es50.env)
```

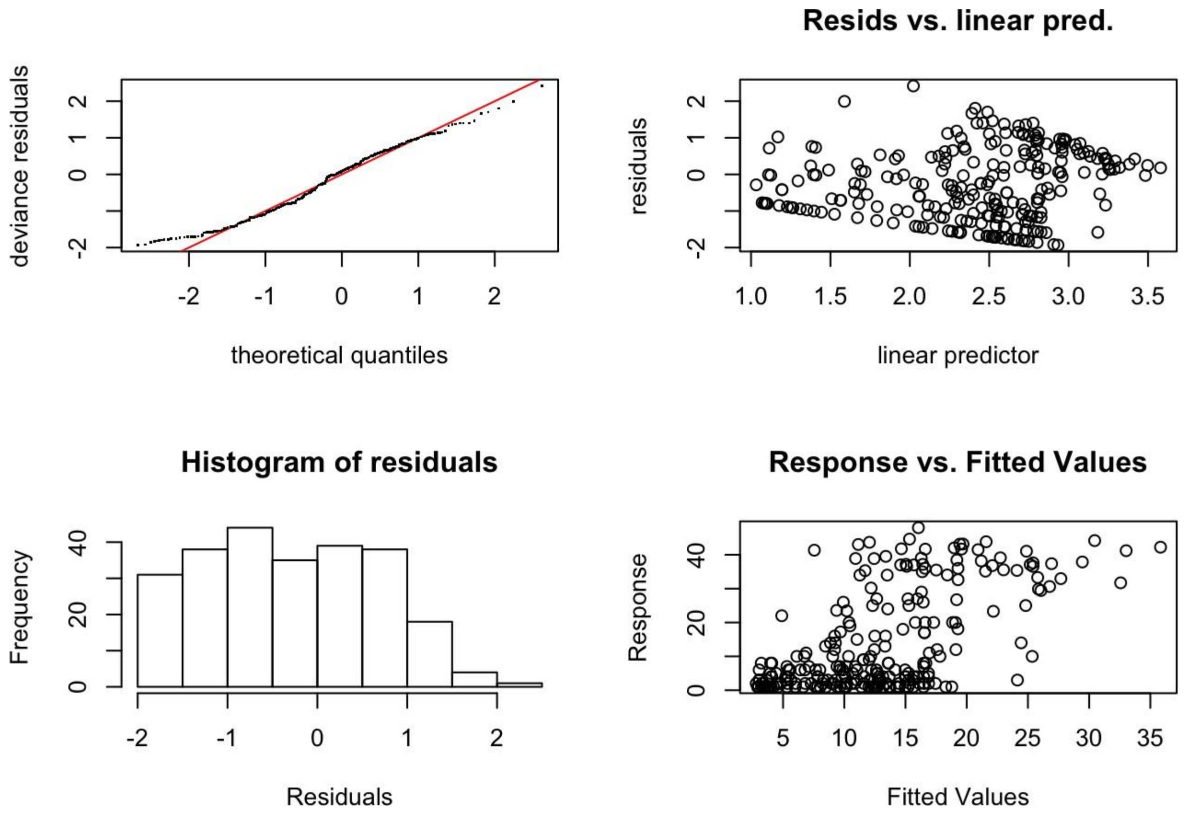


```
##
## Method: REML   Optimizer: outer newton
## full convergence after 13 iterations.
## Gradient range [-0.0002630044,0.0001745135]
## (score 886.9873 & scale 1).
## Hessian positive definite, eigenvalue range [3.801833e-06,101.7219].
## Model rank =  104 / 104
##
## Basis dimension (k) checking results. Low p-value (k-index<1) may
## indicate that k is too low, especially if edf is close to k'.
##
##                          k'      edf k-index p-value
## s(lat,lon)         4.90e+01 1.59e+01    0.90   0.315
## s(temp)            9.00e+00 8.60e-01    0.79   0.025 *
## s(dissolved.oxygen) 9.00e+00 4.99e-05   0.93   0.580
## s(chlorophyll)     9.00e+00 3.76e-05    0.95   0.635
## s(current)         9.00e+00 3.27e-05    0.85   0.100 .
## s(salinity)        9.00e+00 6.62e-01    0.93   0.565
## s(nitrate)         9.00e+00 8.86e-01    0.89   0.270
## ---
## Signif. codes:  0 '***' 0.001 '**' 0.01 '*' 0.05 '.' 0.1 ' ' 1
```

```
summary(deep.es50.env)
```

```
##
## Family: Negative Binomial(1.159)
## Link function: log
##
## Formula:
## ES50 ~ s(lat, lon, bs = "sos") + s(temp) + s(dissolved.oxygen) +
##     s(chlorophyll) + s(current) + s(salinity) + s(nitrate)
##
## Parametric coefficients:
##             Estimate Std. Error z value Pr(>|z|)
## (Intercept)  2.41592    0.06252   38.64   <2e-16 ***
## ---
## Signif. codes:  0 '***' 0.001 '**' 0.01 '*' 0.05 '.' 0.1 ' ' 1
##
## Approximate significance of smooth terms:
##                           edf Ref.df Chi.sq  p-value
## s(lat,lon)          1.588e+01     49 74.255  1.6e-13 ***
## s(temp)             8.596e-01      9  5.854  0.00488 **
## s(dissolved.oxygen) 4.994e-05      9  0.000  0.43457
## s(chlorophyll)      3.756e-05      9  0.000  0.94571
## s(current)          3.274e-05      9  0.000  1.00000
## s(salinity)         6.615e-01      9  2.046  0.03428 *
## s(nitrate)          8.861e-01      9  1.547  0.10430
## ---
## Signif. codes:  0 '***' 0.001 '**' 0.01 '*' 0.05 '.' 0.1 ' ' 1
##
## R-sq.(adj) =  0.287   Deviance explained = 30.5%
## -REML = 886.99  Scale est. = 1         n = 248
```

Model selection for ES50, deep water

```
deep.es50.models <- list(deep.es50.intercept = deep.es50.intercept,
                         deep.es50.latlon = deep.es50.latlon,
                         deep.es50.temp = deep.es50.temp,
                         deep.es50.oxygen = deep.es50.oxygen,
                         deep.es50.chlorophyll = deep.es50.chlorophyll,
                         deep.es50.current = deep.es50.current,
                         deep.es50.salinity = deep.es50.salinity,
                         deep.es50.nitrate = deep.es50.nitrate,
                         deep.es50.env = deep.es50.env)
deep.es50.aic.df <- data.frame(Model = names(deep.es50.models),
                               AIC = sapply(deep.es50.models, function(x) x$aic),
                              akaike.weights(sapply(deep.es50.models, function(x) x$a
ic)))

deep.es50.aic.df <- deep.es50.aic.df[order(deep.es50.aic.df$AIC),]
deep.es50.aic.df$Cumulative.Weight <- cumsum(deep.es50.aic.df$weights)

kable(deep.es50.aic.df, row.names = FALSE)
```

Model	AIC	deltaAIC	rel.LL	weights	Cumulative.Weight
deep.es50.env	1751.424	0.0000000	1.0000000	0.3917528	0.3917528
deep.es50.oxygen	1752.042	0.6181978	0.7341082	0.2875889	0.6793417

Model	AIC	deltaAIC	rel.LL	weights	Cumulative.Weight
deep.es50.temp	1753.522	2.0980682	0.3502759	0.1372216	0.8165632
deep.es50.salinity	1754.545	3.1215021	0.2099783	0.0822596	0.8988228
deep.es50.nitrate	1755.165	3.7416483	0.1539967	0.0603286	0.9591514
deep.es50.latlon	1758.141	6.7175613	0.0347776	0.0136242	0.9727757
deep.es50.chlorophyll	1758.143	6.7191041	0.0347508	0.0136137	0.9863894
deep.es50.current	1758.143	6.7195658	0.0347428	0.0136106	1.0000000
deep.es50.intercept	1811.602	60.1779873	0.0000000	0.0000000	1.0000000

```
write.csv(deep.es50.aic.df, file = "deep.es50.aic.csv")
```

We find that model selection supports the importance of the environment in explaining the number of species in a community. These results are only moderately strong, however; the delta AIC between the "env" model and the one for spatial autocorrelation is only 3.65. We also note that there is no strong signal indicating which predictor variable is important in explaining ES50; both oxygen and salinity are within delta AIC of 2 compared to the full model.

Plots for ES50, deep water

```
qplot(deep$temp, predict(deep.es50.temp, deep)) + geom_smooth(method = "gam")
```

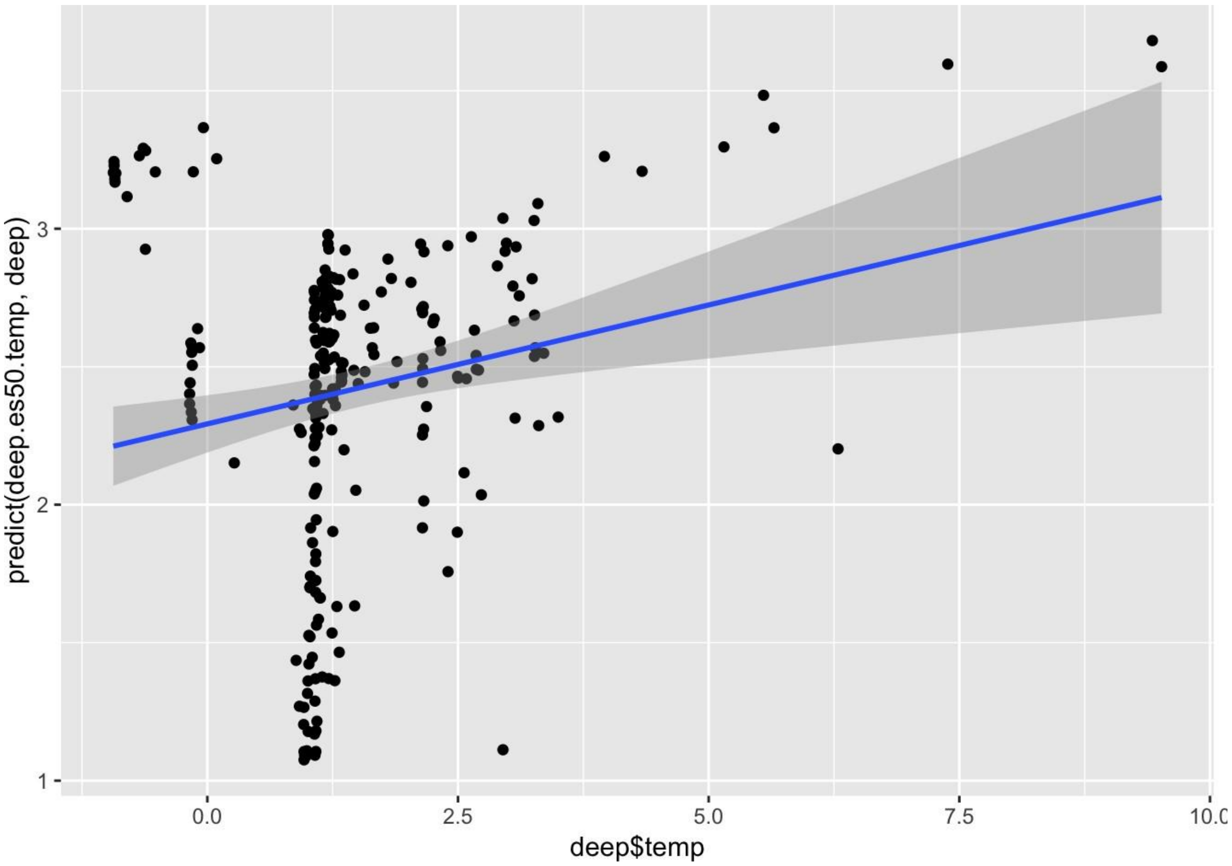


```
qplot(deep$dissolved.oxygen, predict(deep.es50.oxygen, deep)) + geom_smooth(method
= "gam")
```

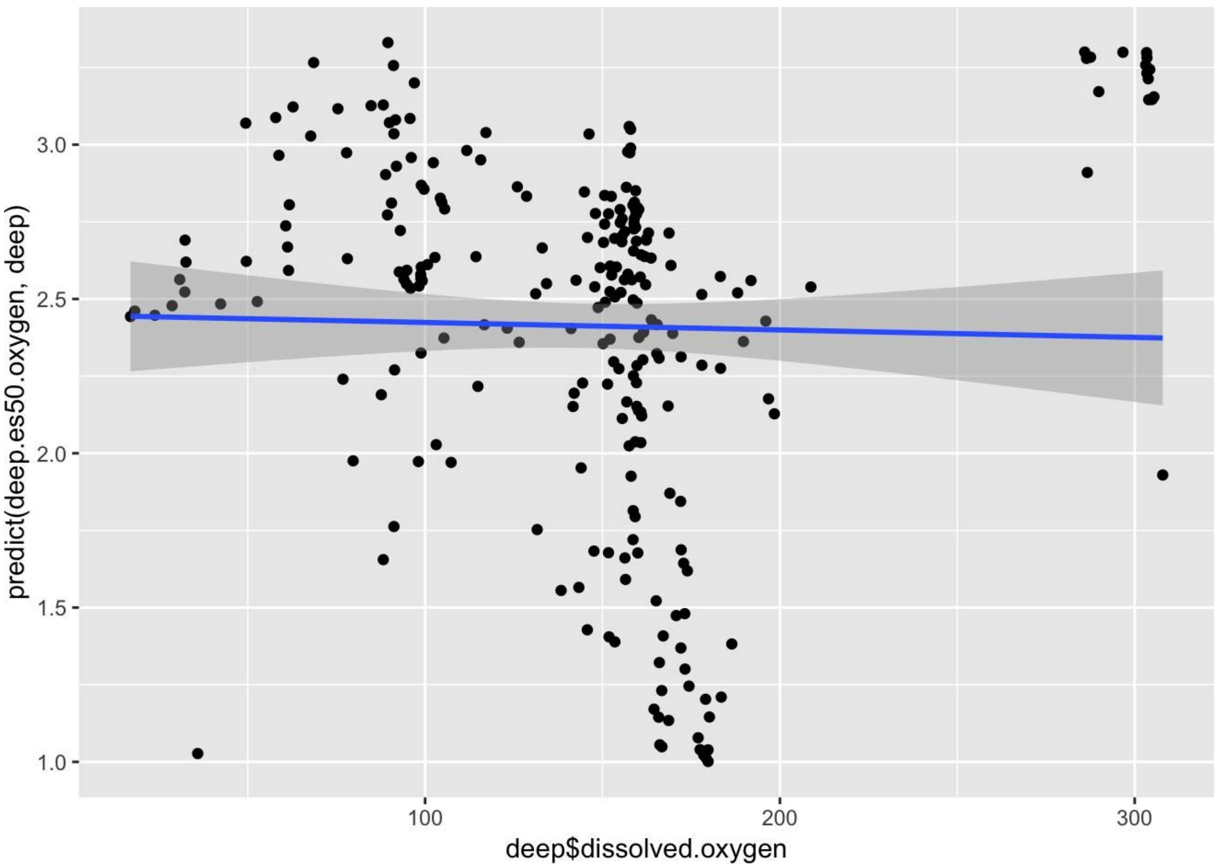


```
qplot(deep$chlorophyll, predict(deep.es50.chlorophyll, deep)) + geom_smooth(method
= "gam")
```

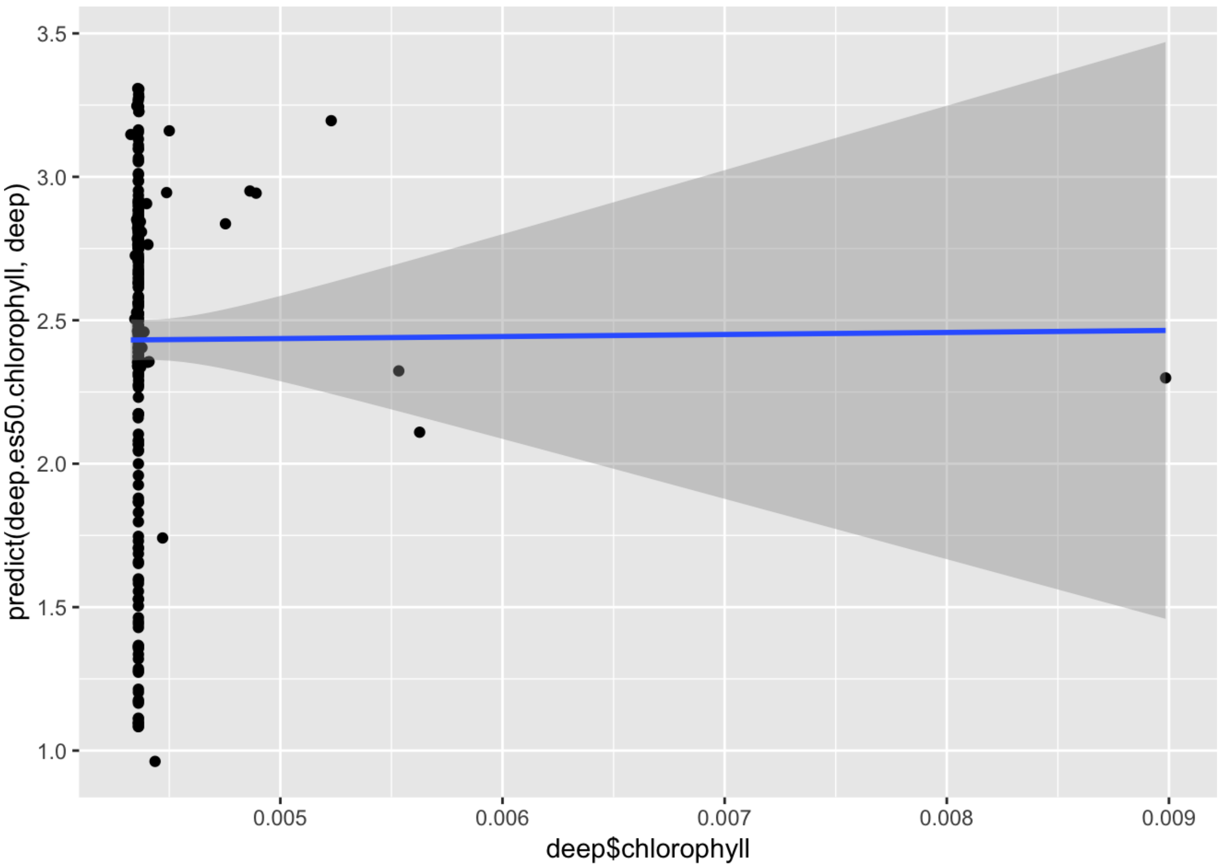


```
qplot(deep$current, predict(deep.es50.current, deep)) + geom_smooth(method = "gam")
```

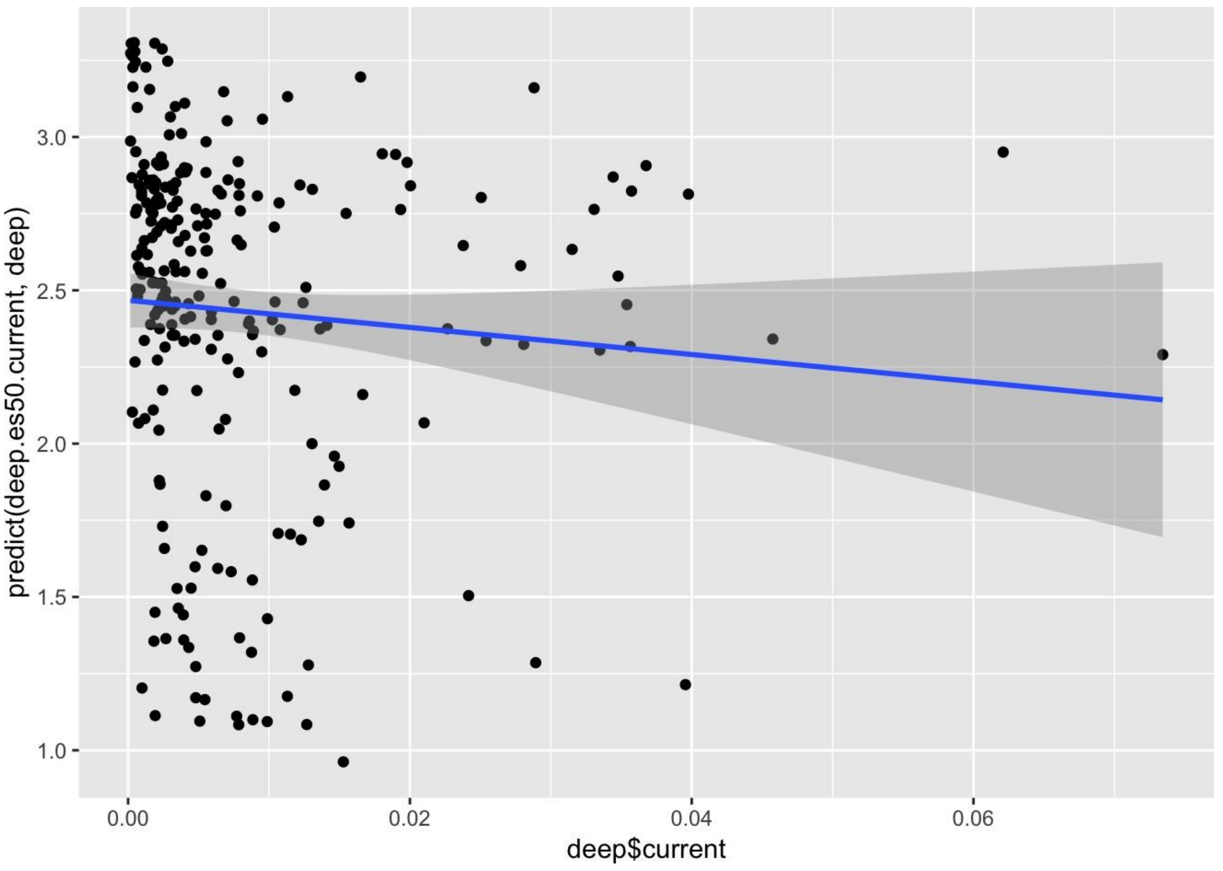


```
qplot(deep$salinity, predict(deep.es50.salinity, deep)) +  geom_smooth(method  =  "ga
m")
```

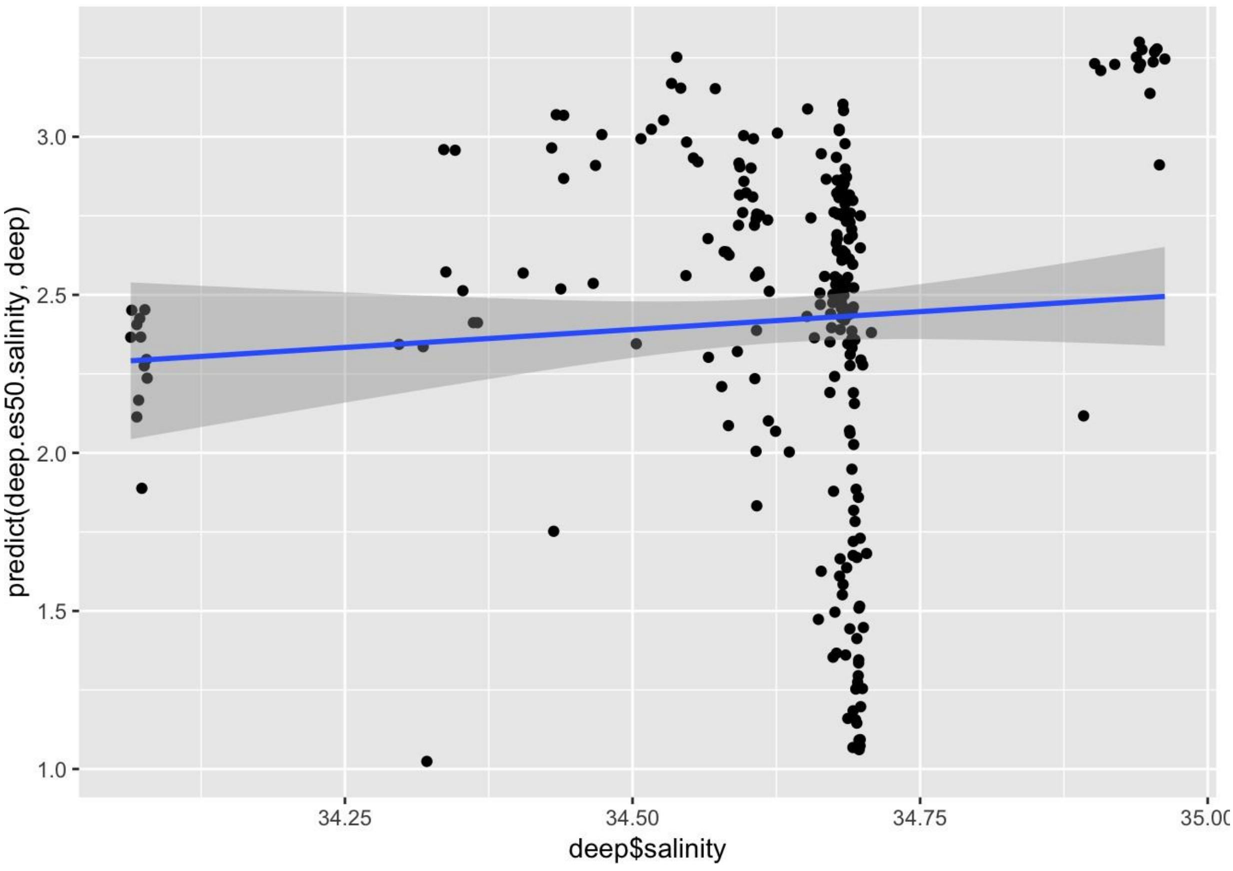


```
qplot(deep$nitrate, predict(deep.es50.nitrate, deep)) + geom_smooth(method = "gam")
```

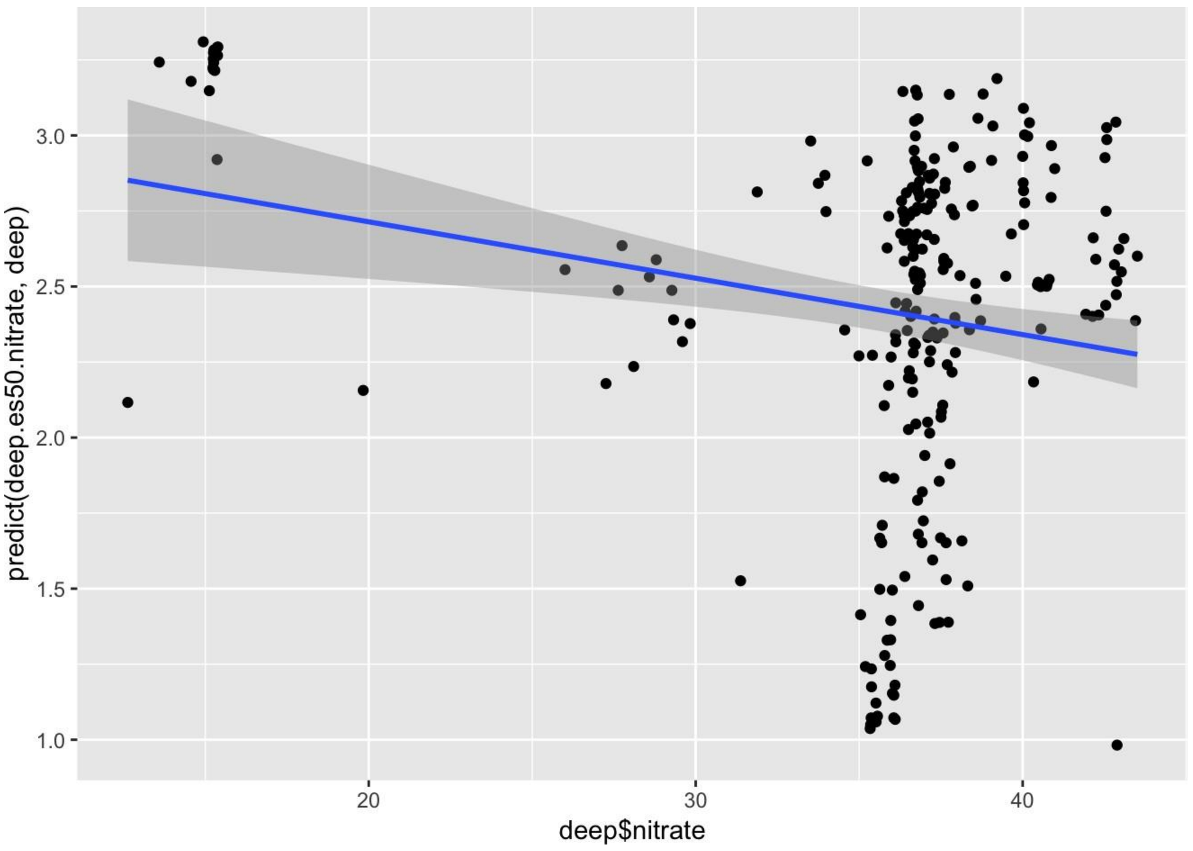
3.0
2.5
2.0
1.5
1.0
predict(deep.es50.nitrate, deep)
20
30
40
deep$nitrate

S2. Species Counts and Environment Based on 5° Latitudinal Bands.

Setup

Shallow water

Deep water

Species Counts and Environment, 5 degree bands

Setup

Shallow water

Data load-in and visualization

First we're going to load in our data and then trim the data frame down to just the columns we need.

```
shallow <- read.csv("shallow.5.degree.csv")
analysis.cols <- c("area", "num.records", "num.species", "ES50", "dissolved.oxygen"
, "temp", "primary.productivity", "chlorophyll", "current", "o2.saturate", "salinit
y", "nitrate")
shallow <- shallow[,analysis.cols]
shallow <- shallow[complete.cases(shallow),]
corrplot(cor(shallow))
```

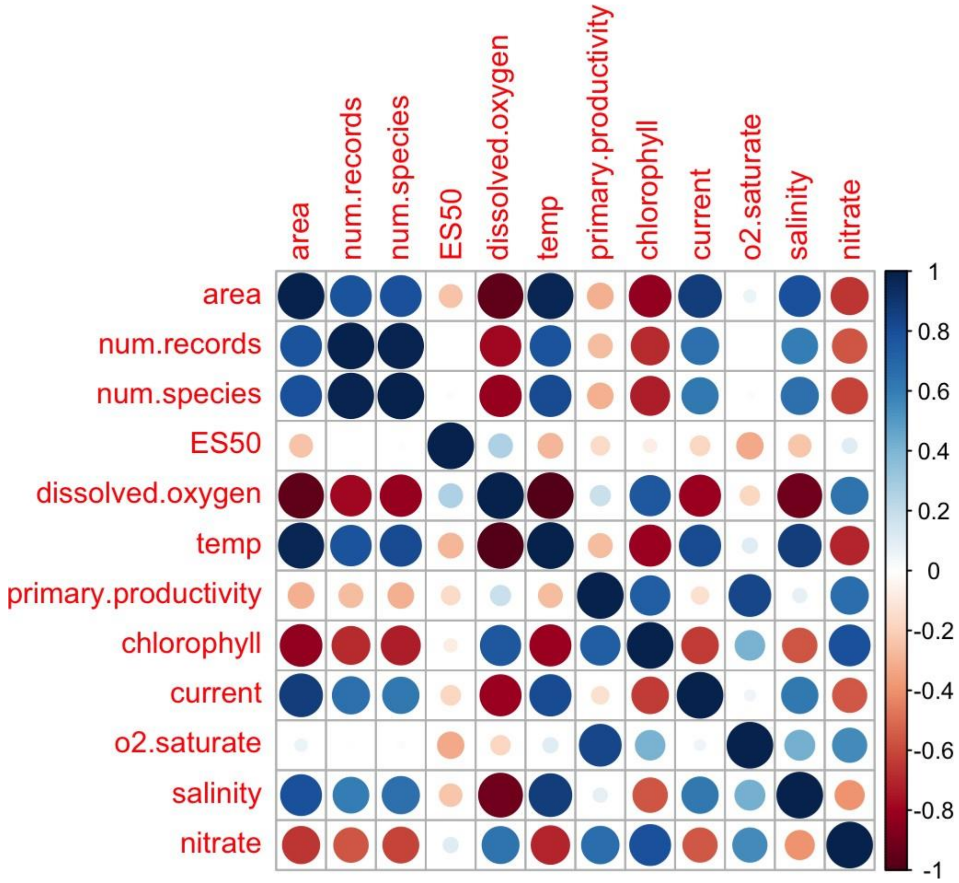

GLMs for number of species, shallow water

We're going to develop a number of GLMs here. The "intercept" glm represents the fit of a model that assumes no relationship between the species counts and the environment and no spatial autocorrelation. The "numrec" model represents the fit of a model that only contains sampling bias. The remainder of the models ("temp", "oxygen", etc.) estimate the effects of a single predictor at a time, controlling for number of records.

```
shallow.numsp.intercept <- glm(num.species ~ 1, family = "poisson", data = shallow)
summary(shallow.numsp.intercept)
```

```
##
## Call:
## glm(formula = num.species ~ 1, family = "poisson", data = shallow)
##
## Deviance Residuals:
##      Min        1Q    Median       3Q      Max
## -119.171   -66.422    -9.743   36.253  137.422
##
## Coefficients:
##              Estimate Std. Error z value Pr(>|z|)
## (Intercept) 8.871672   0.002961    2996   <2e-16 ***
## ---
## Signif. codes:  0 '***' 0.001 '**' 0.01 '*' 0.05 '.' 0.1 ' ' 1
##
## (Dispersion parameter for poisson family taken to be 1)
##
##     Null deviance: 77725  on 15  degrees of freedom
## Residual deviance: 77725  on 15  degrees of freedom
## AIC: 77885
##
## Number of Fisher Scoring iterations: 5
```

```
shallow.numsp.numrec <- glm(num.species ~ num.records, family = "poisson", data = s
hallow)
summary(shallow.numsp.numrec)
```

```
##
## Call:
## glm(formula = num.species ~ num.records, family = "poisson",
##     data = shallow)
##
## Deviance Residuals:
##     Min       1Q   Median       3Q      Max
## -76.324  -35.422    4.397   16.433   50.351
##
## Coefficients:
##              Estimate Std. Error z value Pr(>|z|)
## (Intercept) 7.985e+00  5.350e-03    1492   <2e-16 ***
## num.records 7.009e-05  2.770e-07     253   <2e-16 ***
## ---
## Signif. codes:  0 '***' 0.001 '**' 0.01 '*' 0.05 '.' 0.1 ' ' 1
##
## (Dispersion parameter for poisson family taken to be 1)
##
##     Null deviance: 77725  on 15  degrees of freedom
## Residual deviance: 22879  on 14  degrees of freedom
## AIC: 23041
##
## Number of Fisher Scoring iterations: 5
```

```
shallow.numsp.temp <- glm(num.species ~ num.records + temp, family = "poisson", dat
a = shallow)
summary(shallow.numsp.temp)
```

```
##
## Call:
## glm(formula = num.species ~ num.records + temp, family = "poisson",
##     data = shallow)
##
## Deviance Residuals:
##     Min       1Q   Median       3Q      Max
## -61.993  -25.333   -0.182   14.483   41.945
##
## Coefficients:
##              Estimate Std. Error z value Pr(>|z|)
## (Intercept) 7.613e+00  7.513e-03 1013.22   <2e-16 ***
## num.records 4.501e-05  3.955e-07  113.82   <2e-16 ***
## temp        3.653e-02  4.051e-04   90.17   <2e-16 ***
## ---
## Signif. codes:  0 '***' 0.001 '**' 0.01 '*' 0.05 '.' 0.1 ' ' 1
##
## (Dispersion parameter for poisson family taken to be 1)
##
##     Null deviance: 77725  on 15  degrees of freedom
## Residual deviance: 14661  on 13  degrees of freedom
## AIC: 14825
##
## Number of Fisher Scoring iterations: 5
```

```
shallow.numsp.oxygen <- glm(num.species ~ num.records + dissolved.oxygen, family
= "poisson", data = shallow)
summary(shallow.numsp.oxygen)
```

```
##
## Call:
## glm(formula = num.species ~ num.records + dissolved.oxygen, family = "poisson",
##     data = shallow)
##
## Deviance Residuals:
##     Min       1Q   Median       3Q      Max
## -55.481  -21.004    2.088   17.144   37.645
##
## Coefficients:
##                    Estimate Std. Error z value Pr(>|z|)
## (Intercept)       1.021e+01  2.223e-02  459.27   <2e-16 ***
## num.records       4.296e-05  3.932e-07  109.25   <2e-16 ***
## dissolved.oxygen -7.578e-03  7.637e-05  -99.22   <2e-16 ***
## ---
## Signif. codes:  0 '***' 0.001 '**' 0.01 '*' 0.05 '.' 0.1 ' ' 1
##
## (Dispersion parameter for poisson family taken to be 1)
##
##     Null deviance: 77725  on 15  degrees of freedom
## Residual deviance: 12441  on 13  degrees of freedom
## AIC: 12605
##
## Number of Fisher Scoring iterations: 5
```

```
shallow.numsp.prod <- glm(num.species ~ num.records + primary.productivity, famil
y = "poisson", data = shallow)
summary(shallow.numsp.prod)
```

```
##
## Call:
## glm(formula = num.species ~ num.records + primary.productivity,
##     family = "poisson", data = shallow)
##
## Deviance Residuals:
##     Min       1Q   Median      3Q     Max
## -79.472  -40.316   7.588  18.270  50.049
##
## Coefficients:
##                        Estimate Std. Error z value Pr(>|z|)
## (Intercept)           8.142e+00  8.451e-03  963.39   <2e-16 ***
## num.records           6.768e-05  2.936e-07  230.54   <2e-16 ***
## primary.productivity -2.639e+01  1.138e+00  -23.18   <2e-16 ***
## ---
## Signif. codes:  0 '***' 0.001 '**' 0.01 '*' 0.05 '.' 0.1 ' ' 1
##
## (Dispersion parameter for poisson family taken to be 1)
##
##     Null deviance: 77725  on 15  degrees of freedom
## Residual deviance: 22337  on 13  degrees of freedom
## AIC: 22501
##
## Number of Fisher Scoring iterations: 5
```

```
shallow.numsp.chlorophyll <- glm(num.species ~ num.records + chlorophyll, family
= "poisson", data = shallow)
summary(shallow.numsp.chlorophyll)
```

```
##
## Call:
## glm(formula = num.species ~ num.records + chlorophyll, family = "poisson",
##     data = shallow)
##
## Deviance Residuals:
##     Min       1Q   Median       3Q      Max
## -64.497  -29.226   -8.027   15.647   52.590
##
## Coefficients:
##               Estimate Std. Error z value Pr(>|z|)
## (Intercept)  8.662e+00  9.461e-03  915.52   <2e-16 ***
## num.records  5.093e-05  3.639e-07  139.95   <2e-16 ***
## chlorophyll -1.610e+00  1.991e-02  -80.86   <2e-16 ***
## ---
## Signif. codes:  0 '***' 0.001 '**' 0.01 '*' 0.05 '.' 0.1 ' ' 1
##
## (Dispersion parameter for poisson family taken to be 1)
##
##     Null deviance: 77725  on 15  degrees of freedom
## Residual deviance: 16006  on 13  degrees of freedom
## AIC: 16170
##
## Number of Fisher Scoring iterations: 5
```

```
shallow.numsp.current <- glm(num.species ~  num.records + current, family = "poisso
n", data = shallow)
summary(shallow.numsp.current)
```

```
##
## Call:
## glm(formula = num.species ~ num.records + current, family = "poisson",
##     data = shallow)
##
## Deviance Residuals:
##     Min       1Q   Median       3Q      Max
## -71.570  -28.902   -1.892   16.834   54.011
##
## Coefficients:
##              Estimate Std. Error z value Pr(>|z|)
## (Intercept) 7.802e+00  7.112e-03 1097.03   <2e-16 ***
## num.records 6.242e-05  3.321e-07  187.94   <2e-16 ***
## current     2.769e+00  6.449e-02   42.94   <2e-16 ***
## ---
## Signif. codes:  0 '***' 0.001 '**' 0.01 '*' 0.05 '.' 0.1 ' ' 1
##
## (Dispersion parameter for poisson family taken to be 1)
##
##     Null deviance: 77725  on 15  degrees of freedom
## Residual deviance: 21151  on 13  degrees of freedom
## AIC: 21315
##
## Number of Fisher Scoring iterations: 5
```

```
shallow.numsp.o2.saturate <- glm(num.species ~ num.records + o2.saturate, family
= "poisson", data = shallow)
summary(shallow.numsp.o2.saturate)
```

```
##
## Call:
## glm(formula = num.species ~ num.records + o2.saturate, family = "poisson",
##     data = shallow)
##
## Deviance Residuals:
##     Min       1Q   Median       3Q      Max
## -76.524  -35.726    4.632   16.408   50.325
##
## Coefficients:
##               Estimate Std. Error z value Pr(>|z|)
## (Intercept)  8.225e+00  2.504e-01  32.847   <2e-16 ***
## num.records  7.008e-05  2.773e-07 252.696   <2e-16 ***
## o2.saturate -2.373e-03  2.474e-03  -0.959    0.337
## ---
## Signif. codes:  0 '***' 0.001 '**' 0.01 '*' 0.05 '.' 0.1 ' ' 1
##
## (Dispersion parameter for poisson family taken to be 1)
##
##     Null deviance: 77725  on 15  degrees of freedom
## Residual deviance: 22878  on 13  degrees of freedom
## AIC: 23042
##
## Number of Fisher Scoring iterations: 5
```

```
shallow.numsp.salinity <- glm(num.species ~ num.records + salinity, family = "poi
sson", data = shallow)
summary(shallow.numsp.salinity)
```

```
##
## Call:
## glm(formula = num.species ~ num.records + salinity, family = "poisson",
##     data = shallow)
##
## Deviance Residuals:
##      Min        1Q    Median        3Q       Max
## -54.003   -14.680     4.552    11.755    34.503
##
## Coefficients:
##              Estimate Std. Error z value Pr(>|z|)
## (Intercept) -3.139e+00  1.063e-01  -29.52   <2e-16 ***
## num.records  6.123e-05  3.193e-07  191.77   <2e-16 ***
## salinity     3.356e-01  3.166e-03  106.01   <2e-16 ***
## ---
## Signif. codes:  0 '***' 0.001 '**' 0.01 '*' 0.05 '.' 0.1 ' ' 1
##
## (Dispersion parameter for poisson family taken to be 1)
##
##     Null deviance: 77725.1  on 15  degrees of freedom
## Residual deviance:  9843.3  on 13  degrees of freedom
## AIC: 10008
##
## Number of Fisher Scoring iterations: 5
```

```
shallow.numsp.nitrate <-glm(num.species ~ num.records + nitrate, family = "poiss
on", data = shallow)
summary(shallow.numsp.nitrate)
```

```
##
## Call:
## glm(formula = num.species ~ num.records + nitrate, family = "poisson",
##     data = shallow)
##
## Deviance Residuals:
##      Min        1Q    Median        3Q       Max
## -79.989   -29.903     3.061    19.810    45.032
##
## Coefficients:
##               Estimate Std. Error z value Pr(>|z|)
## (Intercept)  8.312e+00  7.430e-03 1118.66   <2e-16 ***
## num.records  5.822e-05  3.415e-07  170.50   <2e-16 ***
## nitrate     -5.945e-02  1.016e-03  -58.55   <2e-16 ***
## ---
## Signif. codes:  0 '***' 0.001 '**' 0.01 '*' 0.05 '.' 0.1 ' ' 1
##
## (Dispersion parameter for poisson family taken to be 1)
##
##     Null deviance: 77725  on 15  degrees of freedom
## Residual deviance: 19093  on 13  degrees of freedom
## AIC: 19257
##
## Number of Fisher Scoring iterations: 5
```

Model selection for number of species, shallow water

Here we use AIC to compare models for goodness of fit while penalizing for overparameterization. Models are ranked in order of AIC score, with lowest scoring model (model with the best compromise between fit and complexity) first in the list. The delta AIC represents how much the AIC score for each model differs from the top model, an can be used as an estimate of the relative support for each model. A delta AIC of 2 is considered to be potentially a significantly better fit, with higher delta AICs between models indicating increasingly larger differences in model fit while correcting for the number of parameters.

```
shallow.numsp.models <- list(shallow.numsp.intercept = shallow.numsp.intercept,
                             shallow.numsp.temp = shallow.numsp.temp,
                             shallow.numsp.oxygen = shallow.numsp.oxygen,
                             shallow.numsp.prod = shallow.numsp.prod,
                             shallow.numsp.chlorophyll = shallow.numsp.chlorophyll,
                             shallow.numsp.numrec = shallow.numsp.numrec,
                             shallow.numsp.current = shallow.numsp.current,
                             shallow.numsp.o2.saturate = shallow.numsp.o2.saturate,
                             shallow.numsp.salinity = shallow.numsp.salinity,
                             shallow.numsp.nitrate = shallow.numsp.nitrate)
shallow.numsp.aic.df <- data.frame(Model = names(shallow.numsp.models),
                                   AIC = sapply(shallow.numsp.models, function(x) AICc
(x)),
                                  akaike.weights(sapply(shallow.numsp.models, function(x)
AICc(x))))


shallow.numsp.aic.df <-shallow.numsp.aic.df[order(shallow.numsp.aic.df$AIC),]
shallow.numsp.aic.df$Cumulative.Weight  <-  cumsum(shallow.numsp.aic.df$weights)

kable(shallow.numsp.aic.df, row.names = FALSE)
```

Model	AIC	deltaAIC	rel.LL	weights	Cumulative.Weight
shallow.numsp.salinity	10009.54	0.000	1	1	1
shallow.numsp.oxygen	12606.98	2597.441	0	0	1
shallow.numsp.temp	14827.07	4817.526	0	0	1
shallow.numsp.chlorophyll	16172.16	6162.624	0	0	1
shallow.numsp.nitrate	19259.05	9249.507	0	0	1
shallow.numsp.current	21316.87	11307.335	0	0	1
shallow.numsp.prod	22502.95	12493.406	0	0	1
shallow.numsp.numrec	23042.10	13032.562	0	0	1
shallow.numsp.o2.saturate	23044.26	13034.719	0	0	1
shallow.numsp.intercept	77885.64	67876.100	0	0	1

```
write.csv(shallow.numsp.aic.df, file = "shallow.5.degree.numsp.aic.csv")
```

Here we see that the model containing salinity is the best fit, but many other models are almost as good. It is particularly noteworthy that the model containing only the effects of sampling (the “numrec” model) has a delta AIC of < 2 compared to the best-performing model. This indicates that a model that contains only sampling effort is for all practical purposes as good as any model that contains an environmental predictor. As such, we cannot with confidence say that any environmental predictor is useful in explaining the number of species.

Plots for number of species, shallow water

Here we plot the effects of each environmental predictor from its respective single-variable model.

```
qplot(shallow$temp, predict(shallow.numsp.temp, shallow)) + geom_smooth(method = "g
lm")
```

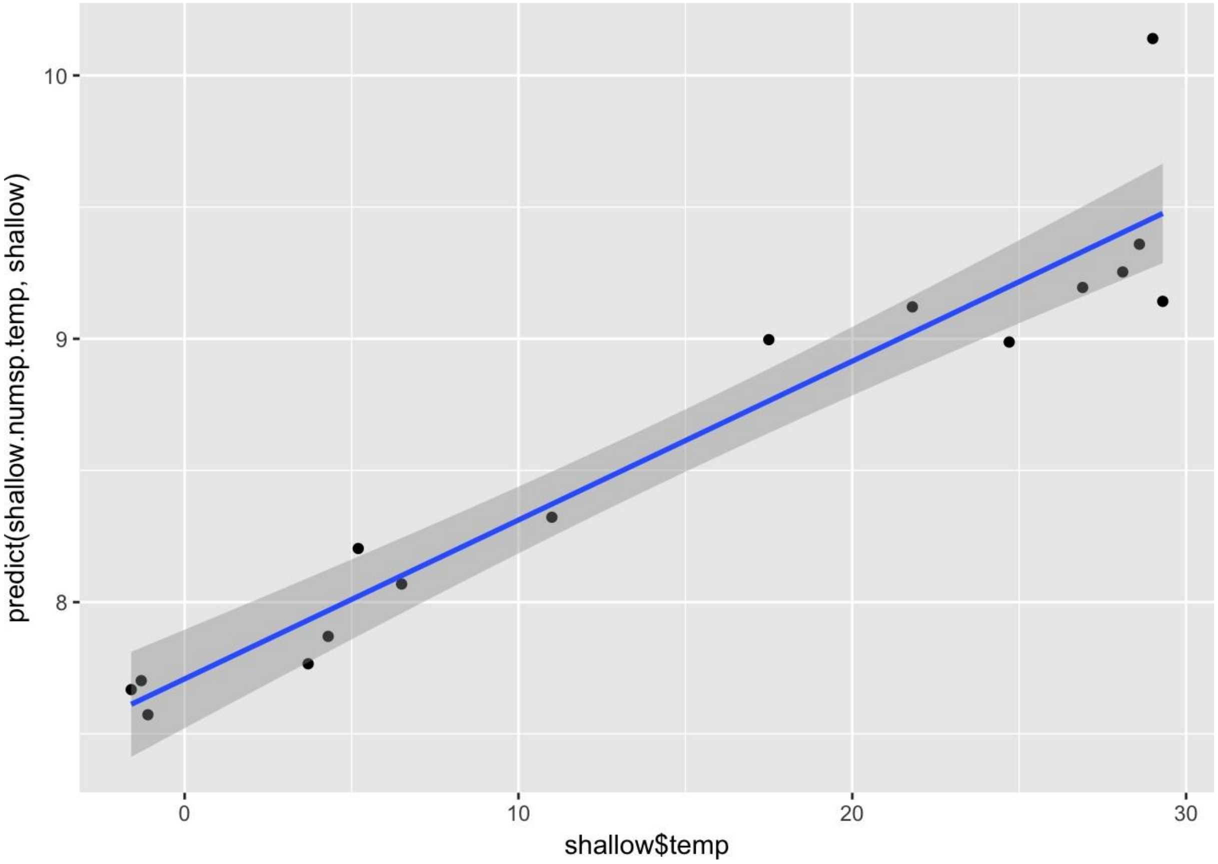


```
qplot(shallow$num.species, predict(shallow.numsp.numrec, shallow)) + geom_smooth(me
thod = "glm")
```

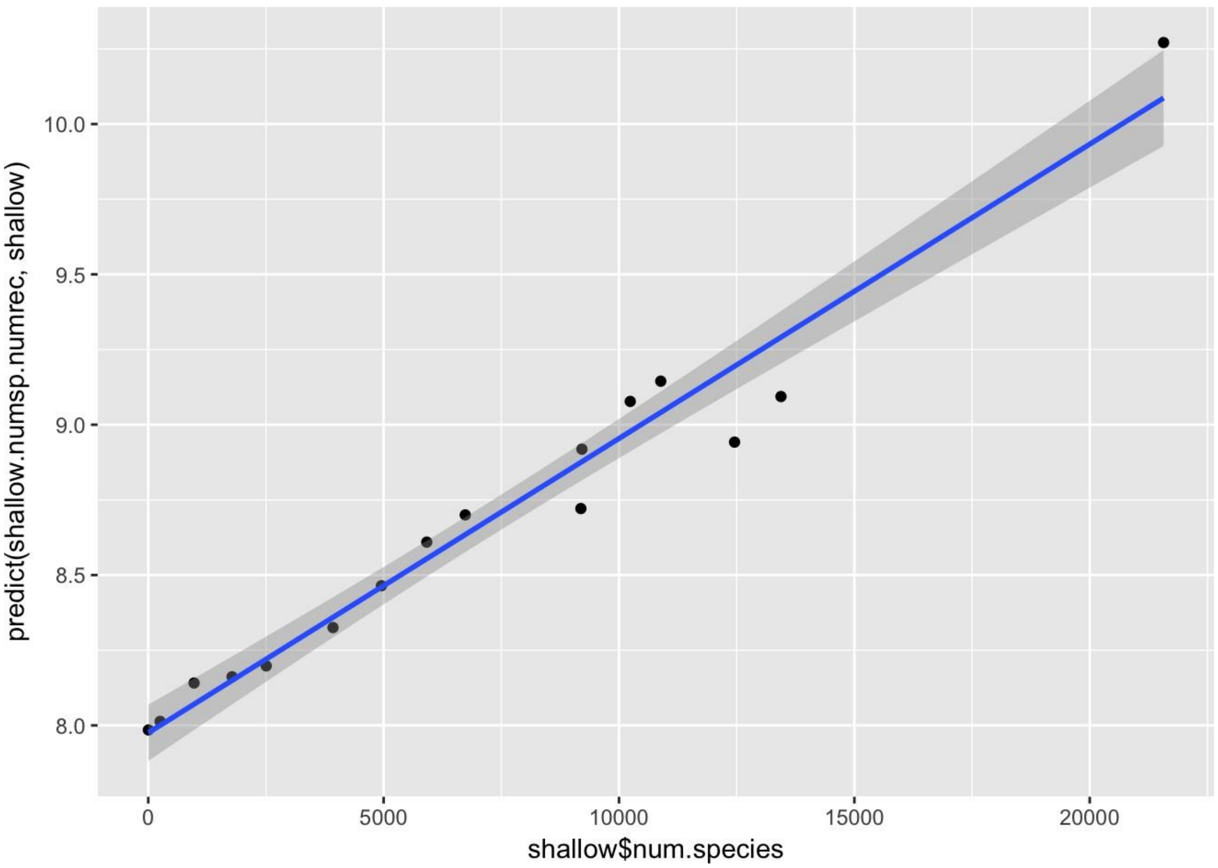


```
qplot(shallow$primary.productivity, predict(shallow.numsp.prod, shallow)) + geom_sm
ooth(method = "glm")
```

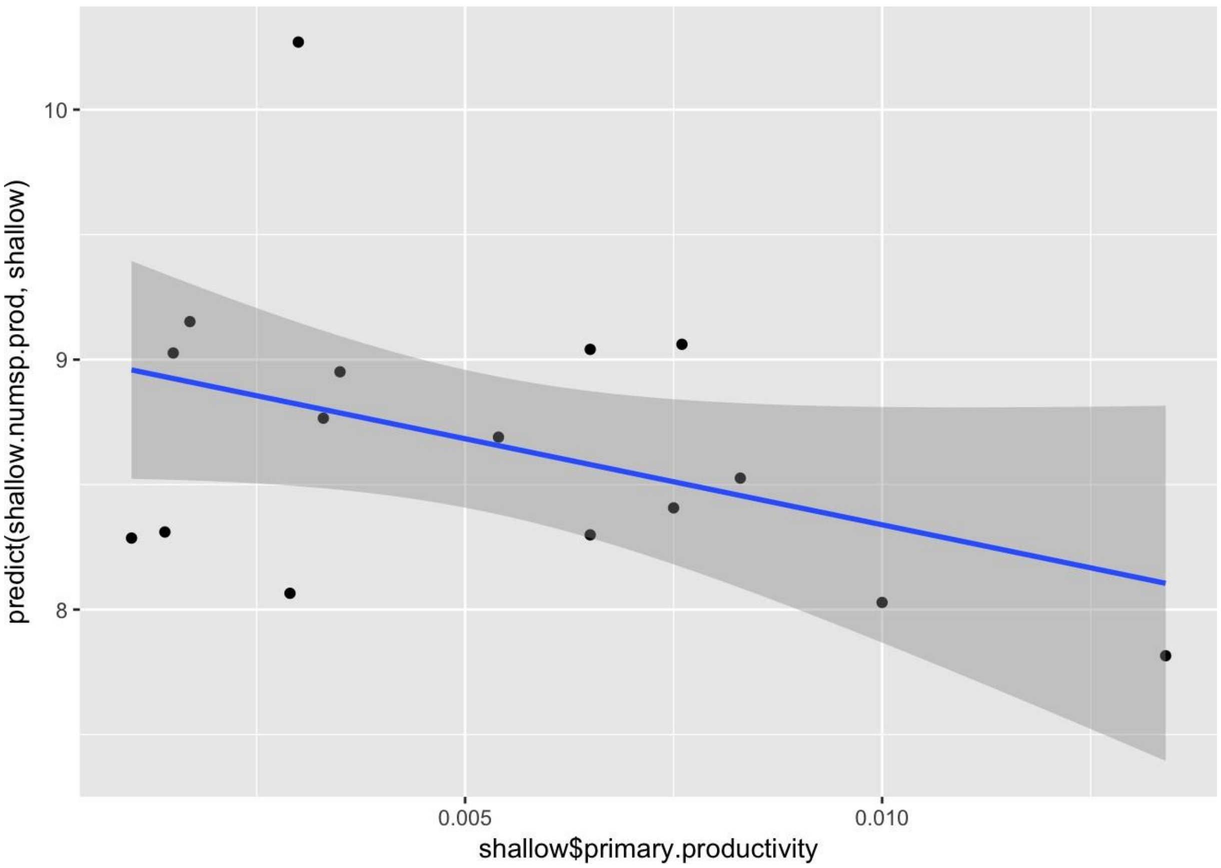


```
qplot(shallow$dissolved.oxygen, predict(shallow.numsp.oxygen, shallow)) + geom_smoo
th(method = "glm")
```

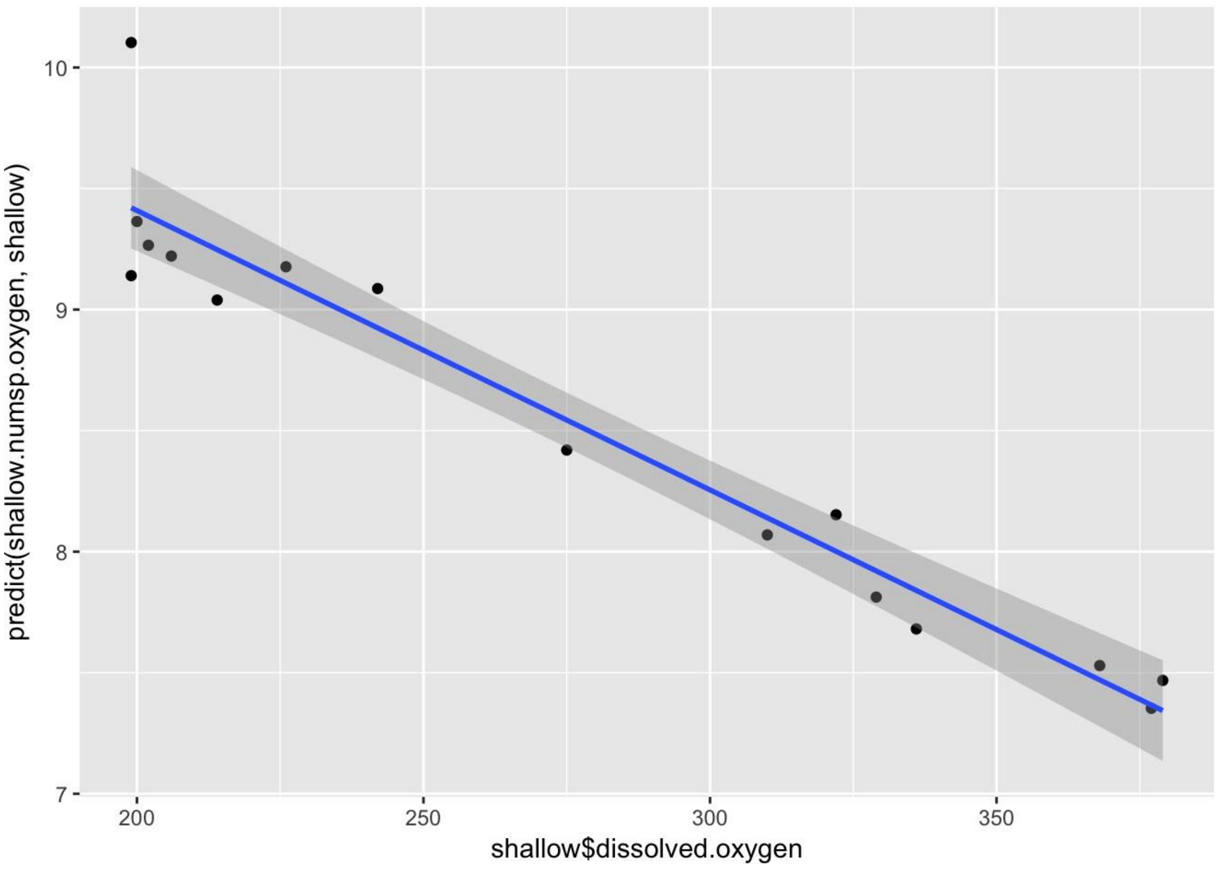


```
qplot(shallow$chlorophyll, predict(shallow.numsp.chlorophyll, shallow)) + geom_smoo
th(method = "glm")
```

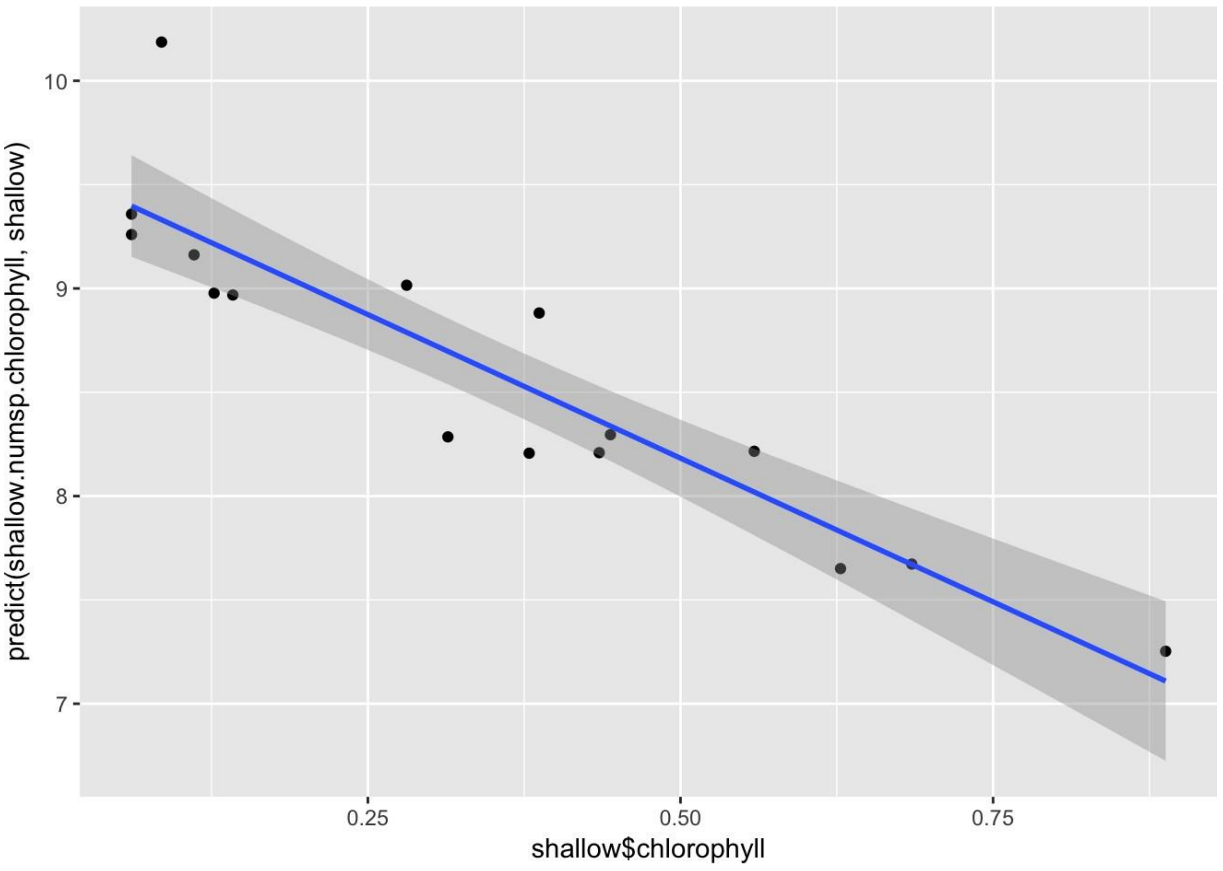


```
qplot(shallow$current, predict(shallow.numsp.current, shallow)) + geom_smooth(metho
d = "glm")
```

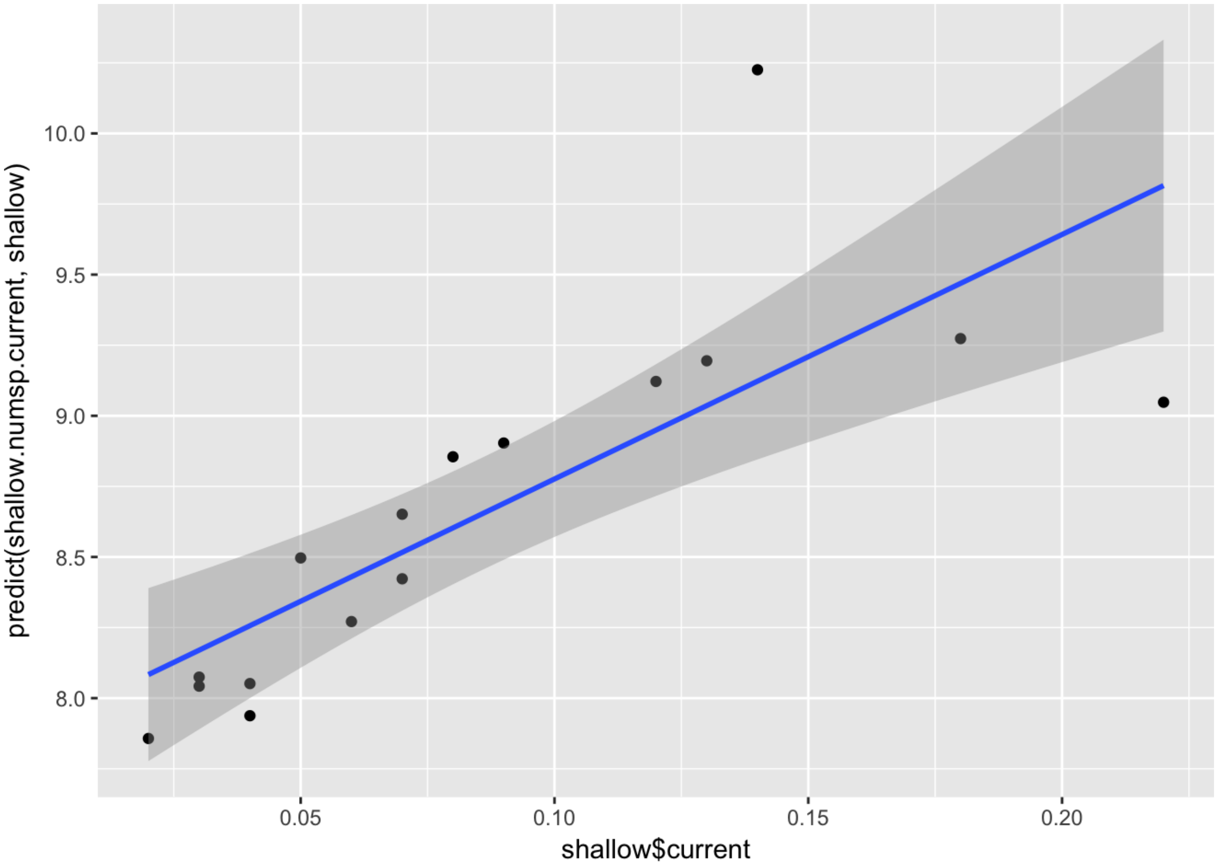


```
qplot(shallow$o2.saturate, predict(shallow.numsp.o2.saturate, shallow)) + geom_smoo
th(method = "glm")
```

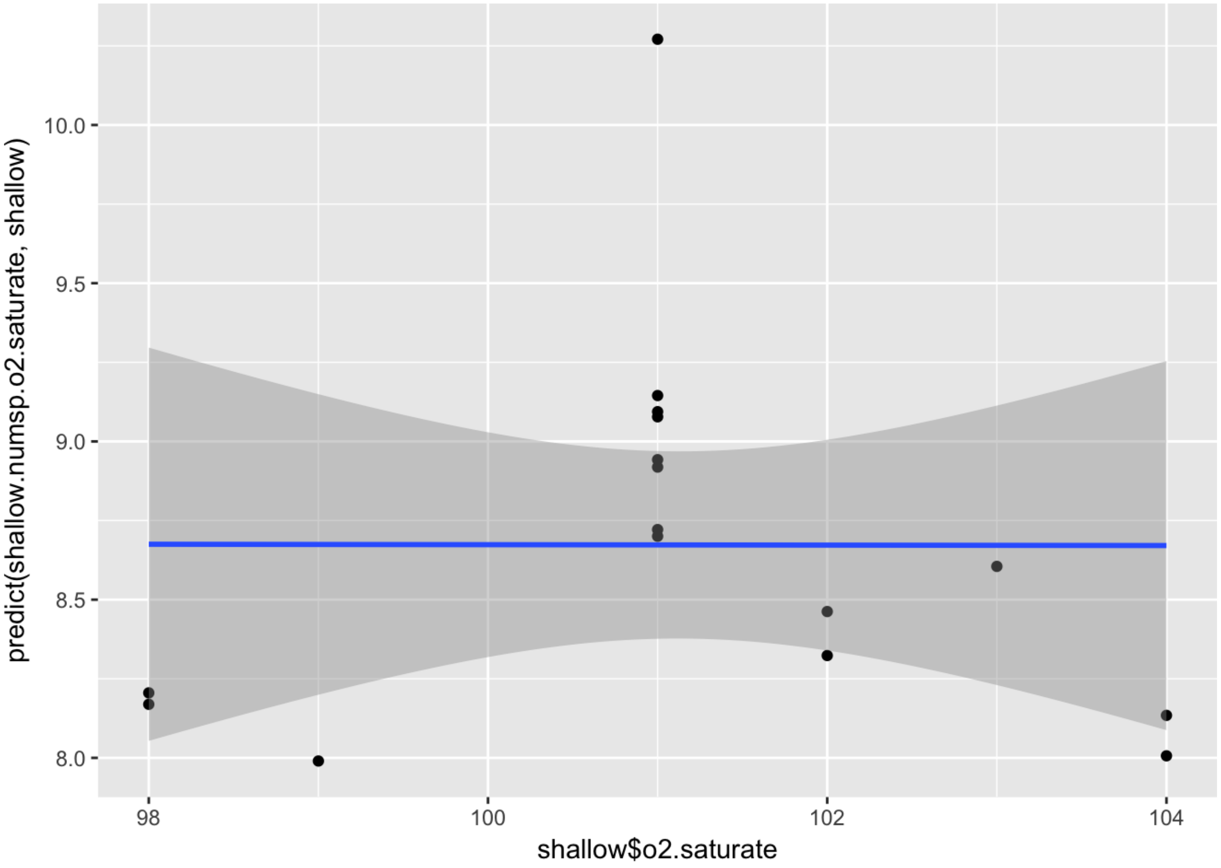


```
qplot(shallow$salinity, predict(shallow.numsp.salinity, shallow)) + geom_smooth(met
hod = "glm")
```

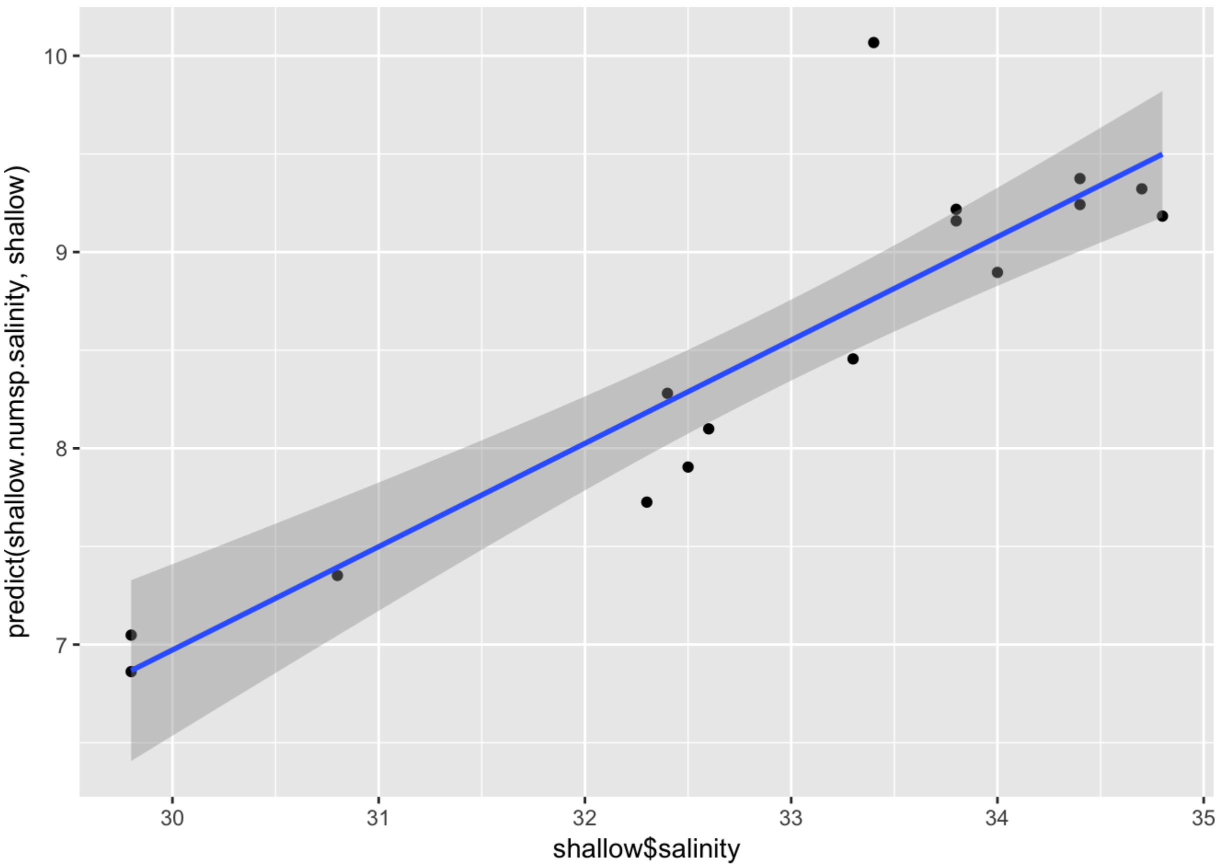


```
qplot(shallow$nitrate, predict(shallow.numsp.nitrate, shallow)) + geom_smooth(metho
d = "glm")
```

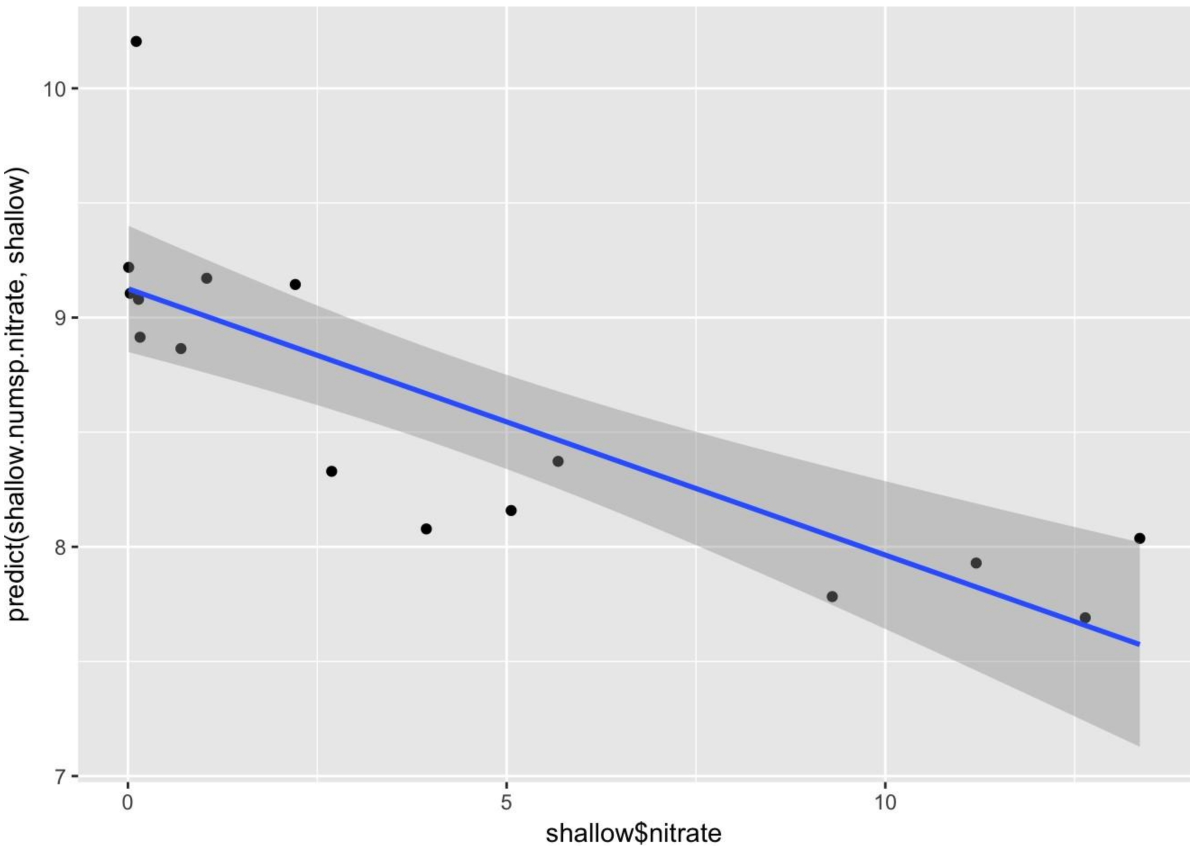

GLMs for ES50, shallow water

These models are identical to the above, except that the response variable is the species richness estimated from the rarefaction curves. These are meant to be estimates of species richness if all sites were sampled equally. Since these measurements are intended to take sampling effort into account, we exclude the “num.records” term for these models.

```
shallow.es50.intercept <- glm(ES50 ~ 1, family = "poisson", data = shallow)
summary(shallow.es50.intercept)
```

```
##
## Call:
## glm(formula = ES50 ~ 1, family = "poisson", data = shallow)
##
## Deviance Residuals:
##      Min        1Q    Median        3Q       Max
## -3.6830   -0.5683   -0.1248    0.4342    3.1278
##
## Coefficients:
##             Estimate Std. Error z value Pr(>|z|)
## (Intercept)  2.52072    0.07089   35.56   <2e-16 ***
## ---
## Signif. codes:  0 '***' 0.001 '**' 0.01 '*' 0.05 '.' 0.1 ' ' 1
##
## (Dispersion parameter for poisson family taken to be 1)
##
##     Null deviance: 30.953  on 15  degrees of freedom
## Residual deviance: 30.953  on 15  degrees of freedom
## AIC: 101.37
##
## Number of Fisher Scoring iterations: 4
```

```
shallow.es50.temp <- glm(ES50 ~ temp, family = "poisson", data = shallow)
summary(shallow.es50.temp)
```

```
##
## Call:
## glm(formula = ES50 ~ temp, family = "poisson", data = shallow)
##
## Deviance Residuals:
##      Min        1Q    Median        3Q       Max
## -4.0864   -0.4847   0.0168    0.4444    2.5418
##
## Coefficients:
##              Estimate Std. Error z value Pr(>|z|)
## (Intercept)  2.648781   0.106161  24.951   <2e-16 ***
## temp        -0.009224   0.005973  -1.544    0.122
## ---
## Signif. codes:  0 '***' 0.001 '**' 0.01 '*' 0.05 '.' 0.1 ' ' 1
##
## (Dispersion parameter for poisson family taken to be 1)
##
##     Null deviance: 30.953  on 15  degrees of freedom
## Residual deviance: 28.559  on 14  degrees of freedom
## AIC: 100.98
##
## Number of Fisher Scoring iterations: 4
```

```
shallow.es50.oxygen <- glm(ES50 ~ dissolved.oxygen, family = "poisson", data = shal
low)
summary(shallow.es50.oxygen)
```

```
##
## Call:
## glm(formula = ES50 ~ dissolved.oxygen, family = "poisson", data = shallow)
##
## Deviance Residuals:
##      Min        1Q    Median        3Q       Max
## -4.1101   -0.4847  -0.0217    0.5021    2.5835
##
## Coefficients:
##                  Estimate Std. Error z value Pr(>|z|)
## (Intercept)      2.113708   0.297882   7.096 1.29e-12 ***
## dissolved.oxygen 0.001467   0.001030   1.424    0.154
## ---
## Signif. codes:  0 '***' 0.001 '**' 0.01 '*' 0.05 '.' 0.1 ' ' 1
##
## (Dispersion parameter for poisson family taken to be 1)
##
##     Null deviance: 30.953  on 15  degrees of freedom
## Residual deviance: 28.934  on 14  degrees of freedom
## AIC: 101.36
##
## Number of Fisher Scoring iterations: 5
```

```
shallow.es50.prod <- glm(ES50 ~ primary.productivity, family = "poisson", data = sh
allow)
summary(shallow.es50.prod)
```

```
##
## Call:
## glm(formula = ES50 ~ primary.productivity, family = "poisson",
##     data = shallow)
##
## Deviance Residuals:
##     Min       1Q   Median       3Q      Max
## -3.7981  -0.6577   0.1055   0.4421   2.8239
##
## Coefficients:
##                      Estimate Std. Error z value Pr(>|z|)
## (Intercept)            2.6137     0.1272  20.542   <2e-16 ***
## primary.productivity -18.1957    21.0964  -0.863    0.388
## ---
## Signif. codes:  0 '***' 0.001 '**' 0.01 '*' 0.05 '.' 0.1 ' ' 1
##
## (Dispersion parameter for poisson family taken to be 1)
##
##     Null deviance: 30.953  on 15  degrees of freedom
## Residual deviance: 30.197  on 14  degrees of freedom
## AIC: 102.62
##
## Number of Fisher Scoring iterations: 4
```

```
shallow.es50.chlorophyll <- glm(ES50 ~ chlorophyll, family = "poisson", data = shal
low)
summary(shallow.es50.chlorophyll)
```

```
##
## Call:
## glm(formula = ES50 ~ chlorophyll, family = "poisson", data = shallow)
##
## Deviance Residuals:
##     Min       1Q   Median       3Q      Max
## -3.5850  -0.6251  -0.0227   0.3515   3.1121
##
## Coefficients:
##             Estimate Std. Error z value Pr(>|z|)
## (Intercept)   2.5634     0.1236  20.746   <2e-16 ***
## chlorophyll  -0.1234     0.2959  -0.417    0.677
## ---
## Signif. codes:  0 '***' 0.001 '**' 0.01 '*' 0.05 '.' 0.1 ' ' 1
##
## (Dispersion parameter for poisson family taken to be 1)
##
##     Null deviance: 30.953  on 15  degrees of freedom
## Residual deviance: 30.778  on 14  degrees of freedom
## AIC: 103.2
##
## Number of Fisher Scoring iterations: 4
```

```
shallow.es50.current <- glm(ES50 ~ current, family = "poisson", data = shallow)
summary(shallow.es50.current)
```

```
##
## Call:
## glm(formula = ES50 ~ current, family = "poisson", data = shallow)
##
## Deviance Residuals:
##      Min        1Q    Median        3Q       Max
## -3.9141   -0.4697    0.0144    0.5715    2.8536
##
## Coefficients:
##             Estimate Std. Error z value Pr(>|z|)
## (Intercept)   2.6260     0.1285  20.436   <2e-16 ***
## current      -1.2585     1.3101  -0.961    0.337
## ---
## Signif. codes:  0 '***' 0.001 '**' 0.01 '*' 0.05 '.' 0.1 ' ' 1
##
## (Dispersion parameter for poisson family taken to be 1)
##
##     Null deviance: 30.953  on 15  degrees of freedom
## Residual deviance: 30.008  on 14  degrees of freedom
## AIC: 102.43
##
## Number of Fisher Scoring iterations: 4
```

```
shallow.es50.o2.saturate <- glm(ES50 ~ o2.saturate, family = "poisson", data = shal
low)
summary(shallow.es50.o2.saturate)
```

```
##
## Call:
## glm(formula = ES50 ~ o2.saturate, family = "poisson", data = shallow)
##
## Deviance Residuals:
##      Min        1Q    Median        3Q       Max
## -4.1129   -0.4209    0.1394    0.4739    2.2156
##
## Coefficients:
##              Estimate Std. Error z value Pr(>|z|)
## (Intercept)   9.87123    4.18524   2.359   0.0183 *
## o2.saturate  -0.07276    0.04147  -1.755   0.0793 .
## ---
## Signif. codes:  0 '***' 0.001 '**' 0.01 '*' 0.05 '.' 0.1 ' ' 1
##
## (Dispersion parameter for poisson family taken to be 1)
##
##     Null deviance: 30.953  on 15  degrees of freedom
## Residual deviance: 27.895  on 14  degrees of freedom
## AIC: 100.32
##
## Number of Fisher Scoring iterations: 4
```

```
shallow.es50.salinity <- glm(ES50 ~ salinity, family = "poisson", data = shallow)
summary(shallow.es50.salinity)
```

```
##
## Call:
## glm(formula = ES50 ~ salinity, family = "poisson", data = shallow)
##
## Deviance Residuals:
##      Min        1Q    Median        3Q       Max
## -4.1826   -0.3624    0.0295    0.5597    2.6606
##
## Coefficients:
##              Estimate Std. Error z value Pr(>|z|)
## (Intercept)   4.34860    1.44031   3.019  0.00253 **
## salinity     -0.05563    0.04388  -1.268  0.20484
## ---
## Signif. codes:  0 '***' 0.001 '**' 0.01 '*' 0.05 '.' 0.1 ' ' 1
##
## (Dispersion parameter for poisson family taken to be 1)
##
##     Null deviance: 30.953  on 15  degrees of freedom
## Residual deviance: 29.379  on 14  degrees of freedom
## AIC: 101.8
##
## Number of Fisher Scoring iterations: 5
```

```
shallow.es50.nitrate <- glm(ES50 ~ nitrate, family = "poisson", data = shallow)
summary(shallow.es50.nitrate)
```

```
##
## Call:
## glm(formula = ES50 ~ nitrate, family = "poisson", data = shallow)
##
## Deviance Residuals:
##      Min        1Q    Median        3Q       Max
## -3.6731   -0.4479   -0.1586    0.4444    3.1042
##
## Coefficients:
##             Estimate Std. Error z value Pr(>|z|)
## (Intercept) 2.484316   0.097391  25.509   <2e-16 ***
## nitrate     0.008352   0.015006   0.557    0.578
## ---
## Signif. codes:  0 '***' 0.001 '**' 0.01 '*' 0.05 '.' 0.1 ' ' 1
##
## (Dispersion parameter for poisson family taken to be 1)
##
##     Null deviance: 30.953  on 15  degrees of freedom
## Residual deviance: 30.646  on 14  degrees of freedom
## AIC: 103.07
##
## Number of Fisher Scoring iterations: 4
```

Model selection for ES50, shallow water

Here we use AIC to compare models for goodness of fit while penalizing for overparameterization. Models are ranked in order of AIC score, with lowest scoring model (model with the best compromise between fit and complexity) first in the list. The delta AIC represents how much the

AIC score for each model differs from the top model, an can be used as an estimate of the relative support for each model. A delta AIC of 2 is considered to be potentially a significantly better fit, with higher delta AICs between models indicating increasingly larger differences in model fit while correcting for the number of parameters.

```
shallow.es50.models <- list(shallow.es50.intercept = shallow.es50.intercept,
                            shallow.es50.temp = shallow.es50.temp,
                            shallow.es50.oxygen = shallow.es50.oxygen,
                            shallow.es50.prod = shallow.es50.prod,
                            shallow.es50.chlorophyll = shallow.es50.chlorophyll,
                            shallow.es50.current = shallow.es50.current,
                            shallow.es50.o2.saturate = shallow.es50.o2.saturate,
                            shallow.es50.salinity = shallow.es50.salinity,
                            shallow.es50.nitrate = shallow.es50.nitrate)
shallow.es50.aic.df <- data.frame(Model = names(shallow.es50.models),
                                  AIC = sapply(shallow.es50.models, function(x) AICc
(x)),
                                  akaike.weights(sapply(shallow.es50.models,  function(x)
 AICc(x))))

shallow.es50.aic.df <- shallow.es50.aic.df[order(shallow.es50.aic.df$AIC),]
shallow.es50.aic.df$Cumulative.Weight <- cumsum(shallow.es50.aic.df$weights)

kable(shallow.es50.aic.df, row.names = FALSE)
```

Model	AIC	deltaAIC	rel.LL	weights	Cumulative.Weight
shallow.es50.o2.saturate	101.2391	0.0000000	1.0000000	0.2104438	0.2104438
shallow.es50.intercept	101.6597	0.4206558	0.8103185	0.1705265	0.3809704
shallow.es50.temp	101.9027	0.6636585	0.7176098	0.1510166	0.5319869
shallow.es50.oxygen	102.2781	1.0390737	0.5947960	0.1251711	0.6571581
shallow.es50.salinity	102.7235	1.4843970	0.4760661	0.1001852	0.7573432
shallow.es50.current	103.3525	2.1133812	0.3476043	0.0731512	0.8304944
shallow.es50.prod	103.5417	2.3026027	0.3162250	0.0665476	0.8970420
shallow.es50.nitrate	103.9905	2.7513899	0.2526639	0.0531716	0.9502136
shallow.es50.chlorophyll	104.1220	2.8829527	0.2365782	0.0497864	1.0000000

```
write.csv(shallow.es50.aic.df, file = "shallow.5.degree.es50.aic.csv")
```

Here we see that the model containing only the intercept is the best fit, indicating no support for any environmental variable as a useful predictor of ES50 in shallow water.

Plots for ES50, shallow water

Here we plot the effects of each environmental predictor from its respective single-variable model.

```
qplot(shallow$temp, shallow$ES50) + geom_smooth(method = "glm")
```

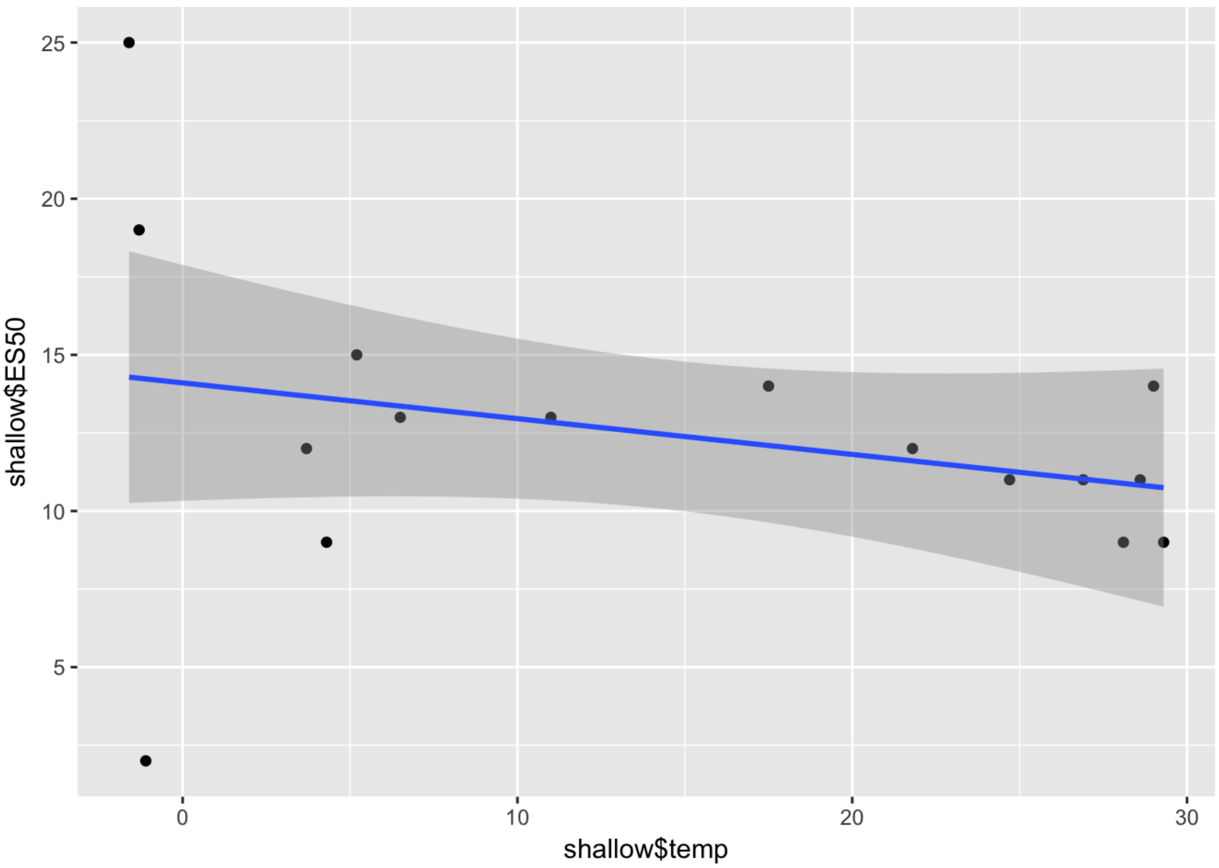


```
qplot(shallow$num.records, shallow$ES50) + geom_smooth(method = "glm")
```

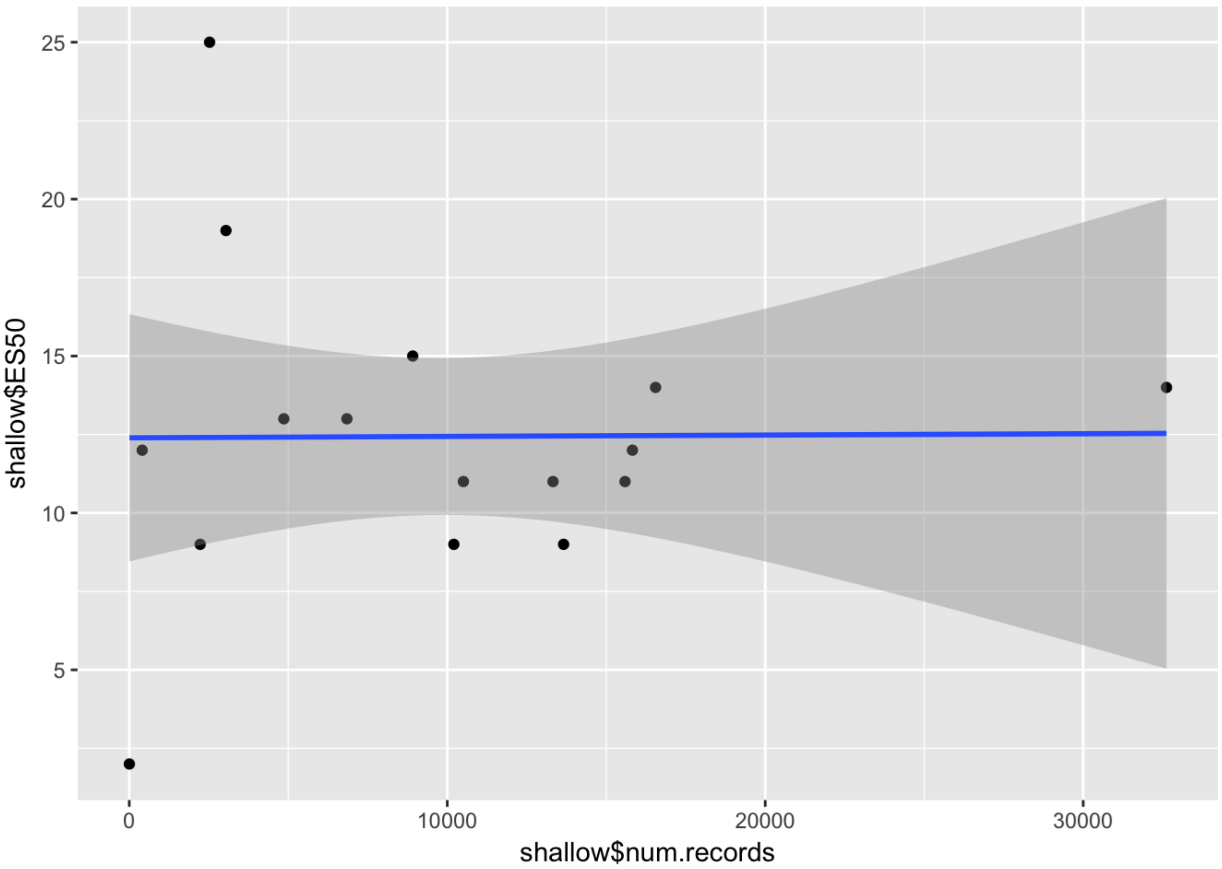


```
qplot(shallow$primary.productivity, shallow$ES50) + geom_smooth(method = "glm")
```

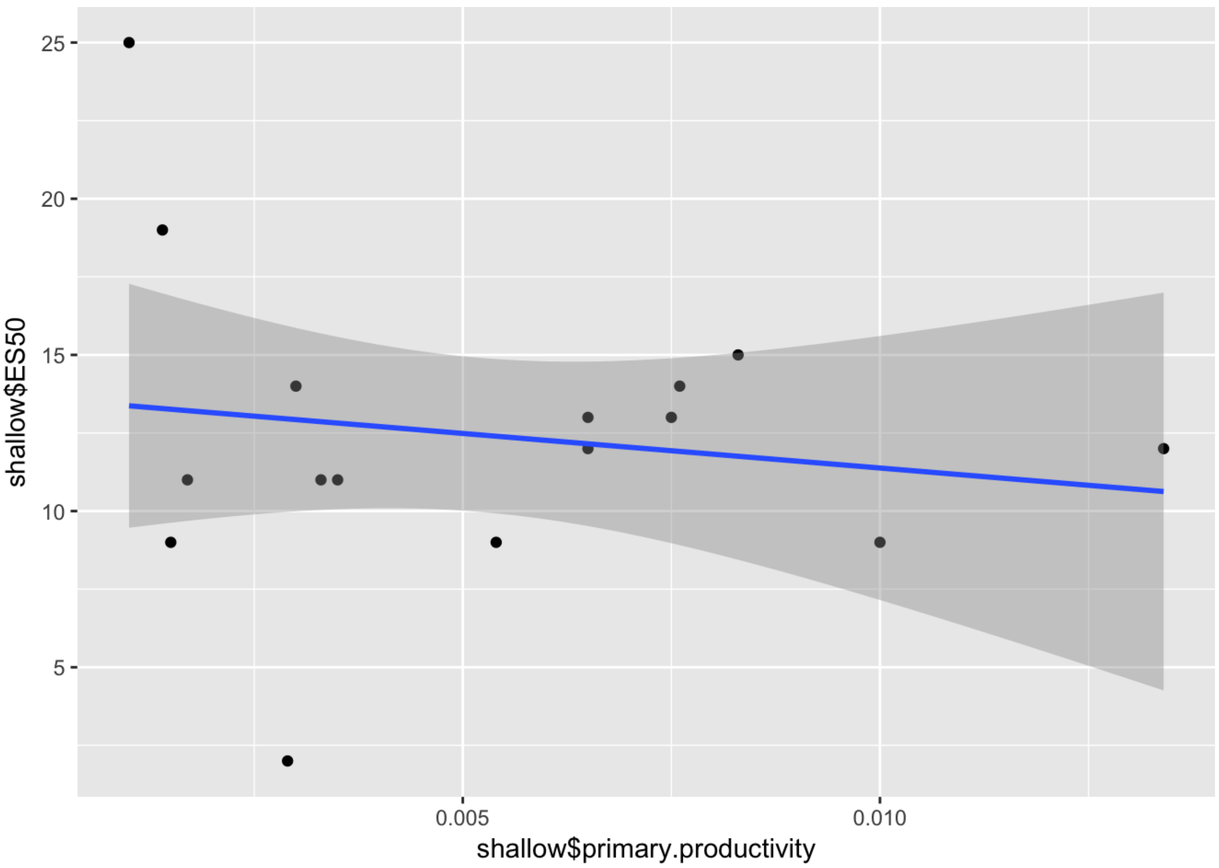


```
qplot(shallow$dissolved.oxygen, shallow$ES50) + geom_smooth(method = "glm")
```

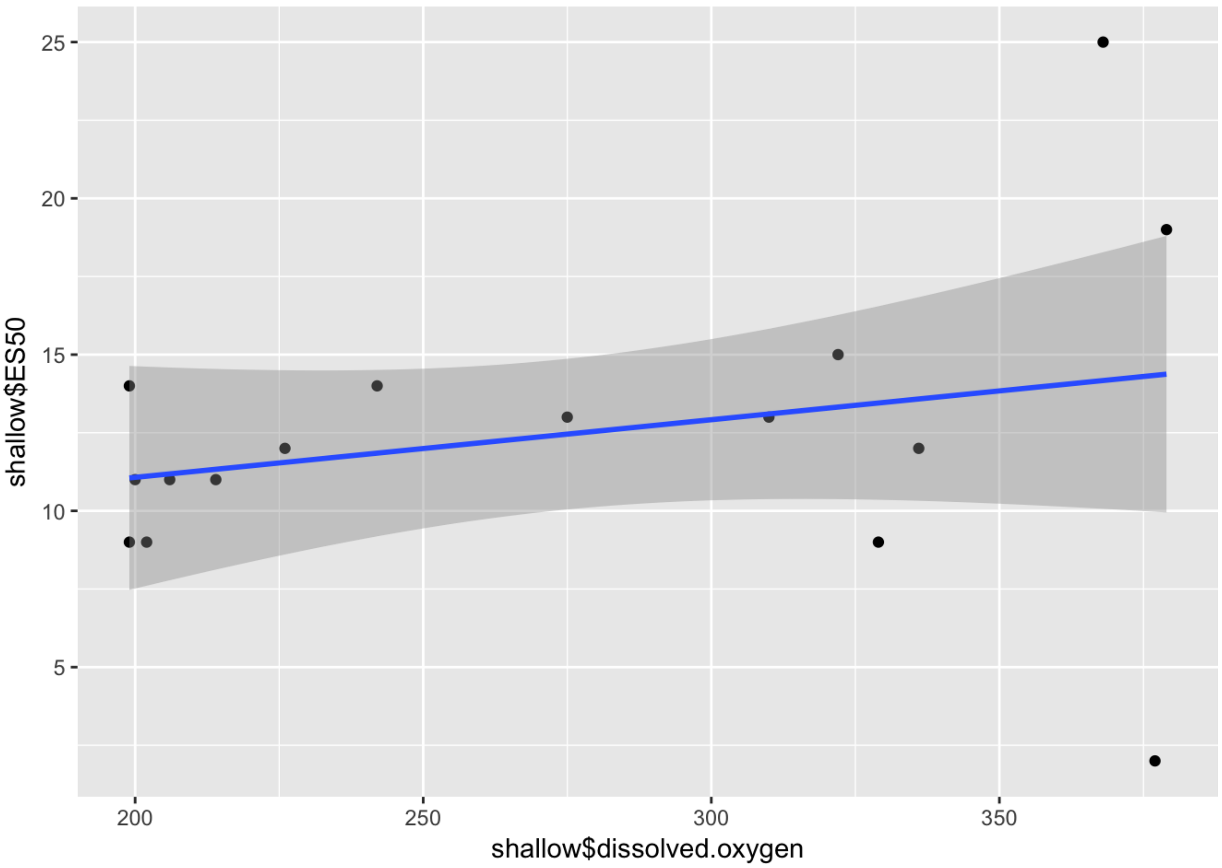


```
qplot(shallow$chlorophyll, shallow$ES50) + geom_smooth(method = "glm")
```

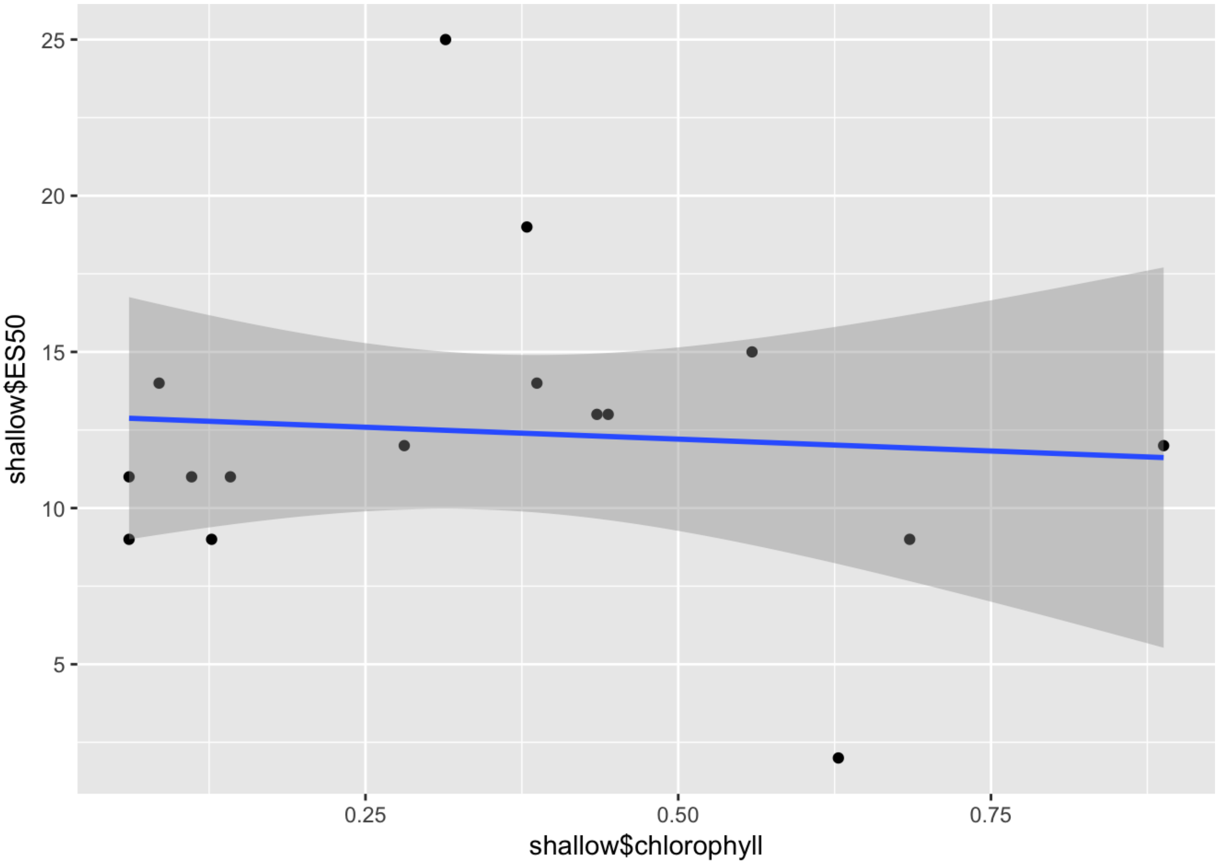


```
qplot(shallow$current, shallow$ES50) + geom_smooth(method = "glm")
```

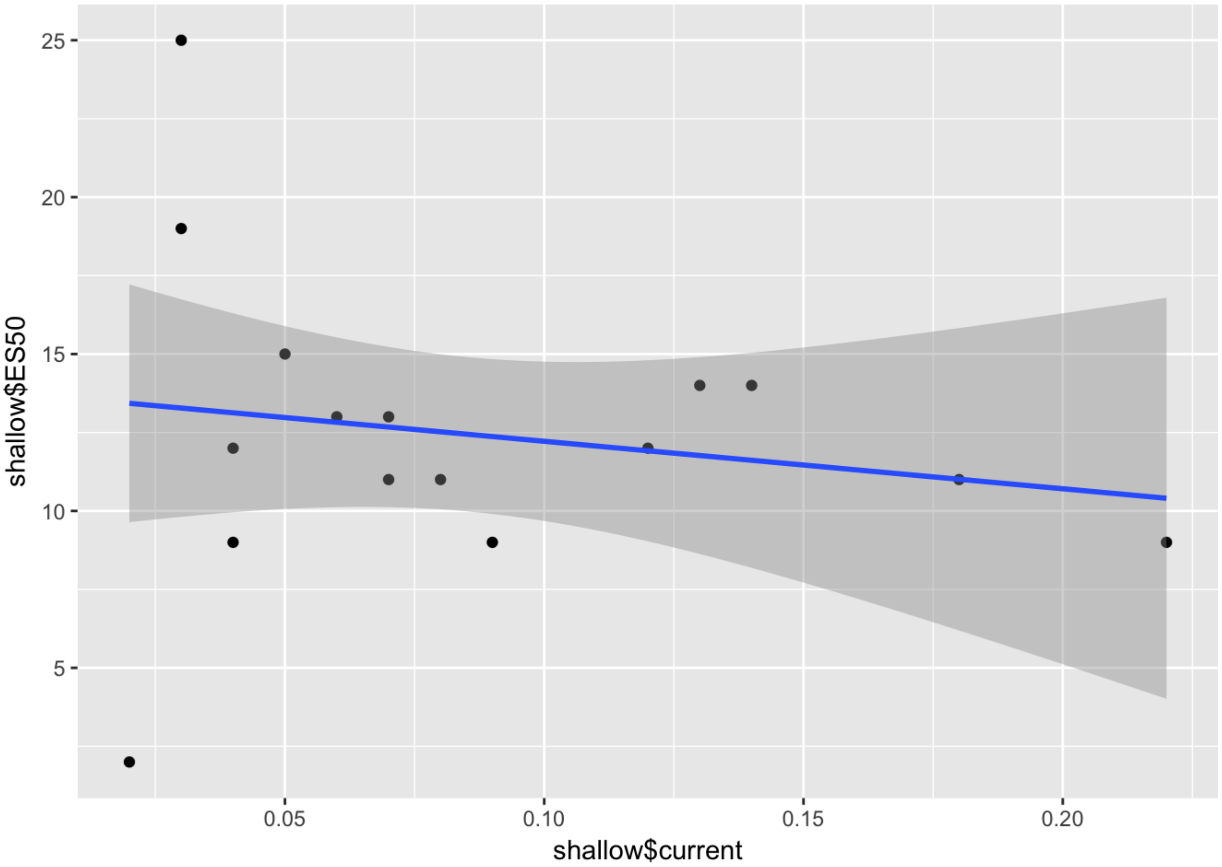


```
qplot(shallow$o2.saturate, shallow$ES50) + geom_smooth(method = "glm")
```

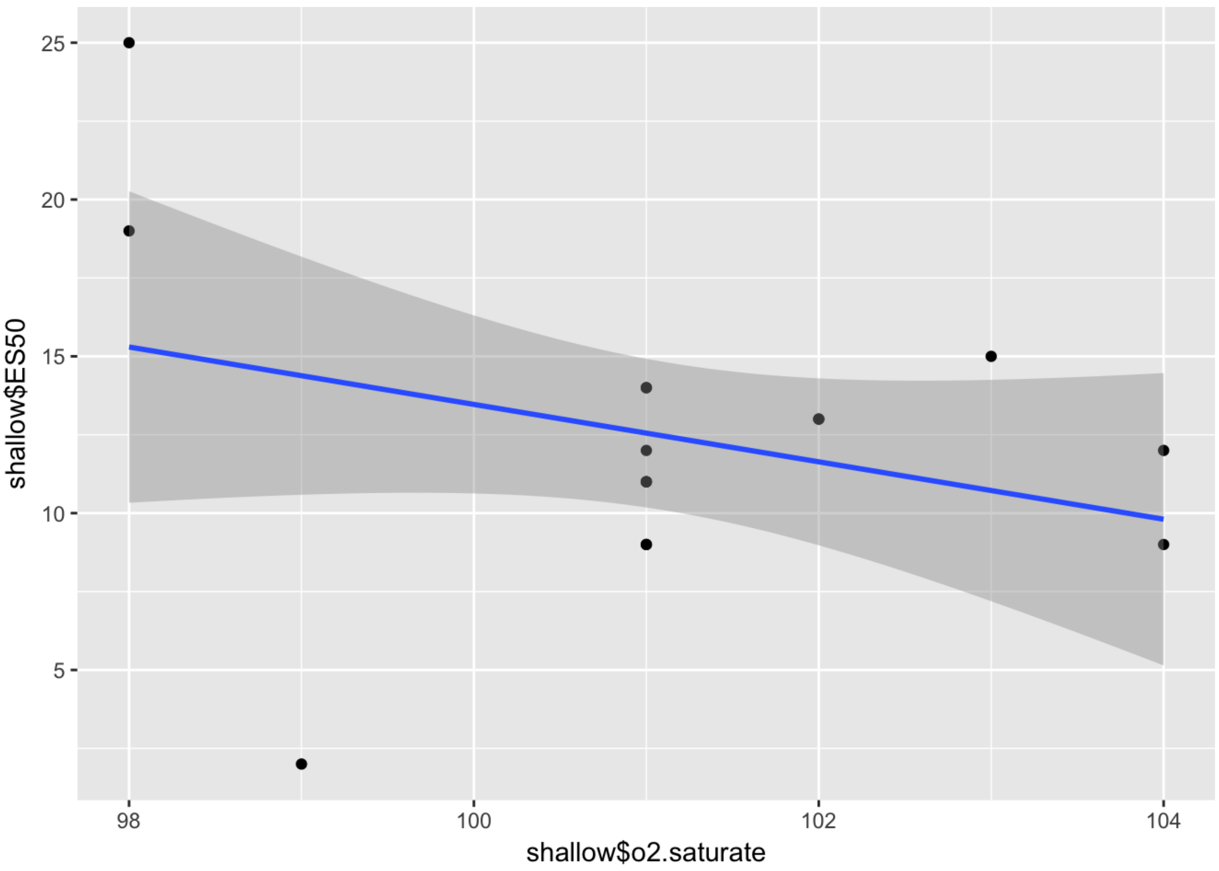


```
qplot(shallow$salinity, shallow$ES50) + geom_smooth(method = "glm")
```

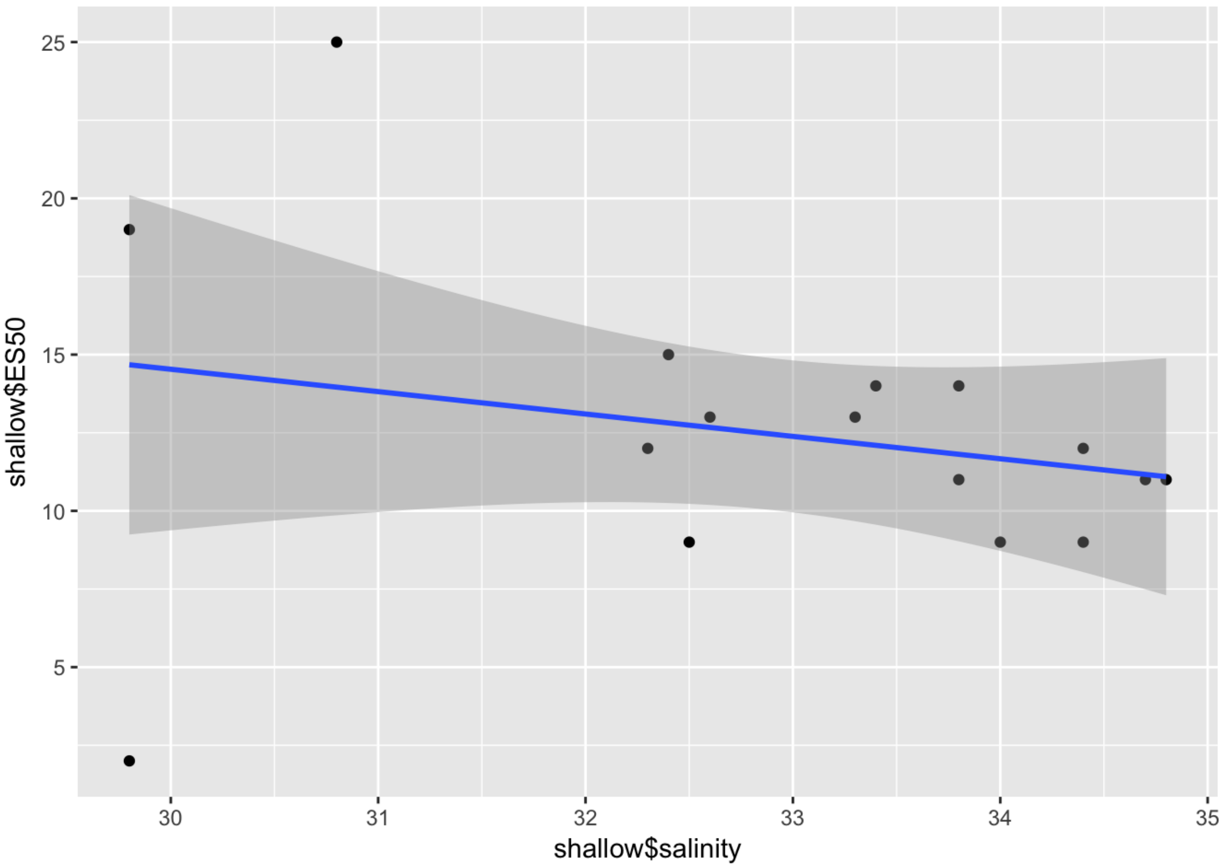


```
qplot(shallow$nitrate, shallow$ES50) + geom_smooth(method = "glm")
```

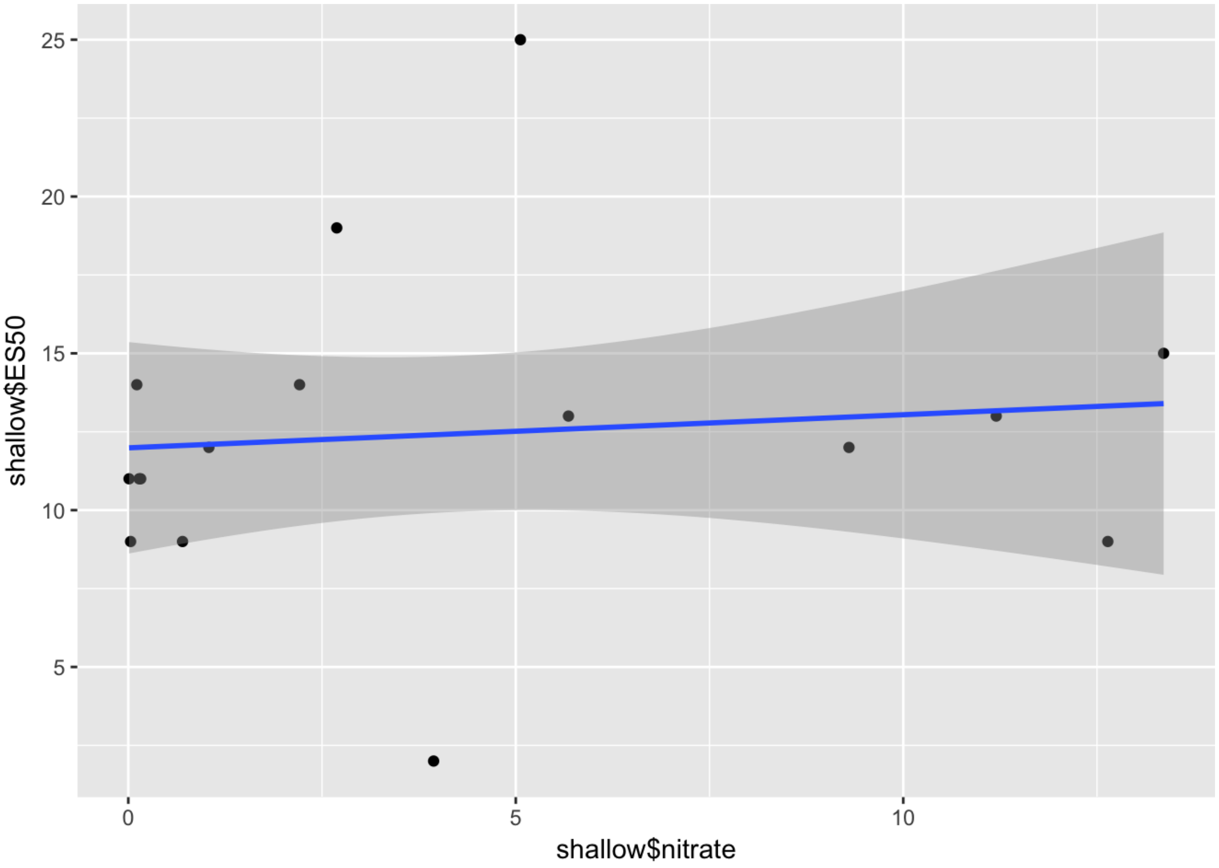

Deep water

Data load-in and visualization

First we're going to load in our data and then trim the data frame down to just the columns we need.

```
deep <- read.csv("deep.5.degree.csv")
analysis.cols <- c("area", "num.records", "num.species", "ES50", "dissolved.oxygen"
, "temp", "chlorophyll", "current", "salinity", "nitrate")
deep <- deep[,analysis.cols]
deep <- deep[complete.cases(deep),]
corrplot(cor(deep))
```

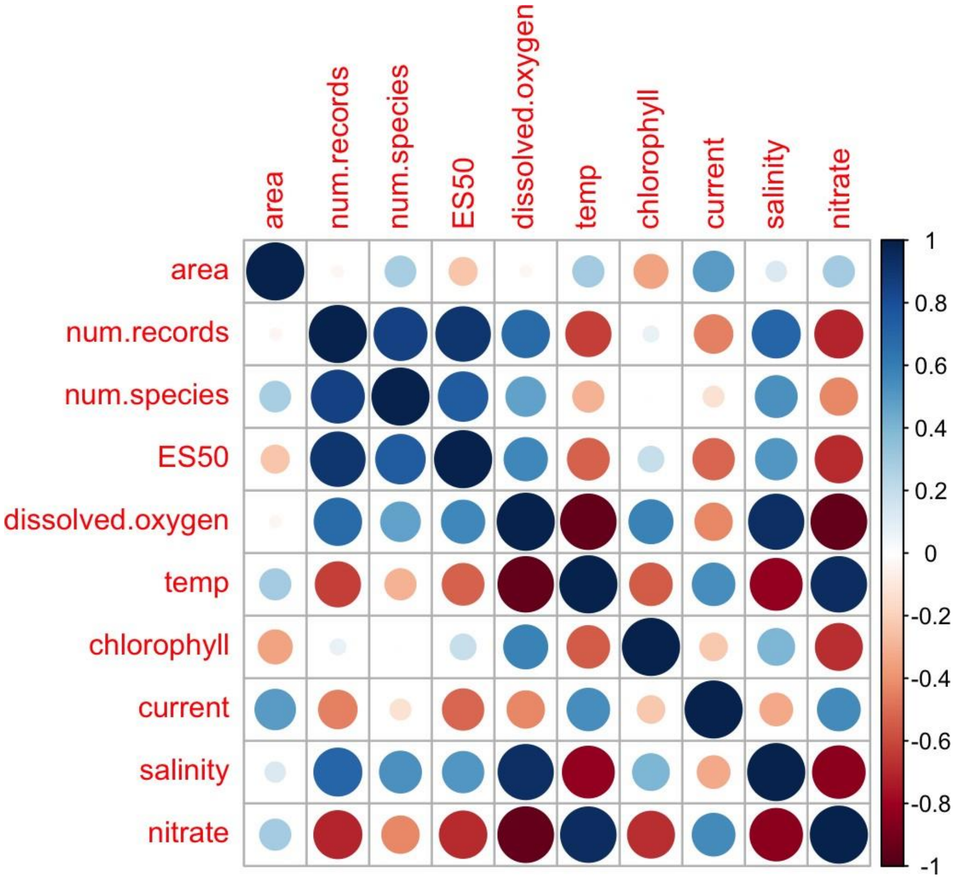

GLMs for number of species, deep water

We're going to develop a number of GLMs here. The "intercept" glm represents the fit of a model that assumes no relationship between the species counts and the environment and no spatial autocorrelation. The "latlon" model represents the fit of a model that fits spatial autocorrelation, but no environmental effects. The "env" model represents the combined effects of all environmental predictors, and the remainder of the models ("temp", "oxygen", etc.) estimate the effects of a single predictor at a time.

```
deep.numsp.intercept <- glm(num.species ~ 1, family = "poisson", data = deep)
summary(deep.numsp.intercept)
```

```
##
## Call:
## glm(formula = num.species ~ 1, family = "poisson", data = deep)
##
## Deviance Residuals:
##      Min        1Q    Median        3Q       Max
## -34.368   -14.677    -8.063    11.747    43.628
##
## Coefficients:
##             Estimate Std. Error z value Pr(>|z|)
## (Intercept) 6.621139   0.009423   702.7   <2e-16 ***
## ---
## Signif. codes:  0 '***' 0.001 '**' 0.01 '*' 0.05 '.' 0.1 ' ' 1
##
## (Dispersion parameter for poisson family taken to be 1)
##
##     Null deviance: 6924.5  on 14  degrees of freedom
## Residual deviance: 6924.5  on 14  degrees of freedom
## AIC: 7047.2
##
## Number of Fisher Scoring iterations: 5
```

```
deep.numsp.numrec <- glm(num.species ~ num.records, family = "poisson", data = dee
p)
summary(deep.numsp.numrec)
```

```
##
## Call:
## glm(formula = num.species ~ num.records, family = "poisson",
##     data = deep)
##
## Deviance Residuals:
##      Min        1Q    Median        3Q       Max
## -24.113   -10.134    -4.026     6.374    26.968
##
## Coefficients:
##              Estimate Std. Error z value Pr(>|z|)
## (Intercept) 6.041e+00  1.443e-02  418.60   <2e-16 ***
## num.records 4.357e-04  6.318e-06   68.97   <2e-16 ***
## ---
## Signif. codes:  0 '***' 0.001 '**' 0.01 '*' 0.05 '.' 0.1 ' ' 1
##
## (Dispersion parameter for poisson family taken to be 1)
##
##     Null deviance: 6924.5  on 14  degrees of freedom
## Residual deviance: 3013.2  on 13  degrees of freedom
## AIC: 3138
##
## Number of Fisher Scoring iterations: 5
```

```
deep.numsp.temp <- glm(num.species ~ num.records + temp, family = "poisson", data =
deep)
summary(deep.numsp.temp)
```

```
##
## Call:
## glm(formula = num.species ~ num.records + temp, family = "poisson",
##     data = deep)
##
## Deviance Residuals:
##     Min       1Q   Median       3Q      Max
## -27.583   -8.959   -1.936    9.030   15.068
##
## Coefficients:
##              Estimate Std. Error z value Pr(>|z|)
## (Intercept) 5.386e+00  2.904e-02  185.44   <2e-16 ***
## num.records 6.214e-04  9.376e-06   66.28   <2e-16 ***
## temp        3.713e-01  1.367e-02   27.16   <2e-16 ***
## ---
## Signif. codes:  0 '***' 0.001 '**' 0.01 '*' 0.05 '.' 0.1 ' ' 1
##
## (Dispersion parameter for poisson family taken to be 1)
##
##     Null deviance: 6924.5  on 14  degrees of freedom
## Residual deviance: 2303.3  on 12  degrees of freedom
## AIC: 2430
##
## Number of Fisher Scoring iterations: 5
```

```
deep.numsp.oxygen <- glm(num.species ~ num.records + dissolved.oxygen, family =
"poisson", data = deep)
summary(deep.numsp.oxygen)
```

```
##
## Call:
## glm(formula = num.species ~ num.records + dissolved.oxygen, family = "poisson",
##     data = deep)
##
## Deviance Residuals:
##     Min       1Q   Median       3Q      Max
## -27.384   -9.683   -3.208   10.448   20.957
##
## Coefficients:
##                    Estimate Std. Error z value Pr(>|z|)
## (Intercept)       6.307e+00  2.787e-02  226.29   <2e-16 ***
## num.records       5.319e-04  1.096e-05   48.53   <2e-16 ***
## dissolved.oxygen -2.340e-03  2.154e-04  -10.87   <2e-16 ***
## ---
## Signif. codes:  0 '***' 0.001 '**' 0.01 '*' 0.05 '.' 0.1 ' ' 1
##
## (Dispersion parameter for poisson family taken to be 1)
##
##     Null deviance: 6924.5  on 14  degrees of freedom
## Residual deviance: 2891.7  on 12  degrees of freedom
## AIC: 3018.4
##
## Number of Fisher Scoring iterations: 5
```

```
deep.numsp.chlorophyll <- glm(num.species ~ num.records + chlorophyll, family =
"poisson", data = deep)
summary(deep.numsp.chlorophyll)
```

```
##
## Call:
## glm(formula = num.species ~ num.records + chlorophyll, family = "poisson",
##     data = deep)
##
## Deviance Residuals:
##      Min       1Q   Median       3Q      Max
## -24.058  -10.067   -3.955    6.157   27.056
##
## Coefficients:
##              Estimate Std. Error z value Pr(>|z|)
## (Intercept) 5.184e+00  8.279e-01   6.262 3.81e-10 ***
## num.records 4.362e-04  6.342e-06  68.776  < 2e-16 ***
## chlorophyll 1.939e+02  1.874e+02   1.035    0.301
## ---
## Signif. codes:  0 '***' 0.001 '**' 0.01 '*' 0.05 '.' 0.1 ' ' 1
##
## (Dispersion parameter for poisson family taken to be 1)
##
##     Null deviance: 6924.5  on 14  degrees of freedom
## Residual deviance: 3012.2  on 12  degrees of freedom
## AIC: 3138.9
##
## Number of Fisher Scoring iterations: 5
```

```
deep.numsp.current <- glm(num.species ~ num.records + current, family = "poisson",
data = deep)
summary(deep.numsp.current)
```

```
##
## Call:
## glm(formula = num.species ~ num.records + current, family = "poisson",
##     data = deep)
##
## Deviance Residuals:
##      Min        1Q    Median        3Q       Max
## -20.1499  -11.5189    0.9779    6.6915   17.6137
##
## Coefficients:
##              Estimate Std. Error z value Pr(>|z|)
## (Intercept) 4.747e+00  4.264e-02  111.33   <2e-16 ***
## num.records 6.496e-04  9.417e-06   68.98   <2e-16 ***
## current     1.208e+02  3.541e+00   34.13   <2e-16 ***
## ---
## Signif. codes:  0 '***' 0.001 '**' 0.01 '*' 0.05 '.' 0.1 ' ' 1
##
## (Dispersion parameter for poisson family taken to be 1)
##
##     Null deviance: 6924.5  on 14  degrees of freedom
## Residual deviance: 1915.3  on 12  degrees of freedom
## AIC: 2042
##
## Number of Fisher Scoring iterations: 4
```

```
deep.numsp.salinity <- glm(num.species ~ num.records + salinity, family = "poisso
n", data = deep)
summary(deep.numsp.salinity)
```

```
##
## Call:
## glm(formula = num.species ~ num.records + salinity, family = "poisson",
##     data = deep)
##
## Deviance Residuals:
##     Min       1Q   Median       3Q      Max
## -27.744   -9.292   -3.172    7.553   23.695
##
## Coefficients:
##               Estimate Std. Error z value Pr(>|z|)
## (Intercept)  3.348e+01  3.423e+00   9.780  < 2e-16 ***
## num.records  5.147e-04  1.183e-05  43.513  < 2e-16 ***
## salinity    -7.938e-01  9.905e-02  -8.014 1.11e-15 ***
## ---
## Signif. codes:  0 '***' 0.001 '**' 0.01 '*' 0.05 '.' 0.1 ' ' 1
##
## (Dispersion parameter for poisson family taken to be 1)
##
##     Null deviance: 6924.5  on 14  degrees of freedom
## Residual deviance: 2949.8  on 12  degrees of freedom
## AIC: 3076.5
##
## Number of Fisher Scoring iterations: 5
```

```
deep.numsp.nitrate <- glm(num.species ~  num.records +  nitrate, family =  "poisson"
, data = deep)
summary(deep.numsp.nitrate)
```

```
##
## Call:
## glm(formula = num.species ~  num.records  +  nitrate, family  =  "poisson",
##       data = deep)
##
## Deviance Residuals:
##      Min        1Q    Median        3Q       Max
## -25.694    -9.033    -2.933    10.986    20.035
##
## Coefficients:
##                Estimate Std. Error z value  Pr(>|z|)
## (Intercept) 4.348e+00   7.881e-02   55.16   <2e-16 ***
##  num.records  6.670e-04   1.227e-05   54.37   <2e-16 ***
## nitrate       4.146e-02   1.871e-03   22.16   <2e-16 ***
## ---
## Signif. codes:  0 '***' 0.001 '**' 0.01 '*' 0.05 '.' 0.1 ' ' 1
##
## (Dispersion parameter for poisson family taken to be 1)
##
##   Null  deviance:  6924.5   on  14   degrees  of  freedom
## Residual deviance: 2473.9  on 12  degrees of freedom
## AIC: 2600.7
##
## Number of Fisher Scoring iterations: 5
```

Model selection for number of species, deep water

Here we use AIC to compare models for goodness of fit while penalizing for overparameterization. Models are ranked in order of AIC score, with lowest scoring model (model with the best compromise between fit and complexity) first in the list. The delta AIC represents how much the AIC score for each model differs from the top model, an can be used as an estimate of the relative support for each model. A delta AIC of 2 is considered to be potentially a significantly better fit, with higher delta AICs between models indicating increasingly larger differences in model fit while correcting for the number of parameters.

```
deep.numsp.models <- list(deep.numsp.intercept = deep.numsp.intercept,
                          deep.numsp.temp = deep.numsp.temp,
                          deep.numsp.numrec = deep.numsp.numrec,
                          deep.numsp.oxygen = deep.numsp.oxygen,
                          deep.numsp.chlorophyll = deep.numsp.chlorophyll,
                          deep.numsp.current = deep.numsp.current,
                          deep.numsp.salinity = deep.numsp.salinity,
                          deep.numsp.nitrate = deep.numsp.nitrate)
deep.numsp.aic.df <- data.frame(Model = names(deep.numsp.models),
                                AIC = sapply(deep.numsp.models, function(x) AICc(x)),
                               akaike.weights(sapply(deep.numsp.models, function(x) AI
Cc(x))))

deep.numsp.aic.df <- deep.numsp.aic.df[order(deep.numsp.aic.df$AIC),]
deep.numsp.aic.df$Cumulative.Weight <- cumsum(deep.numsp.aic.df$weights)

kable(deep.numsp.aic.df, row.names = FALSE)
```

Model	AIC	deltaAIC	rel.LL	weights	Cumulative.Weight
deep.numsp.current	2044.206	0.0000	1	1	1
deep.numsp.temp	2432.181	387.9754	0	0	1
deep.numsp.nitrate	2602.863	558.6573	0	0	1
deep.numsp.oxygen	3020.607	976.4008	0	0	1
deep.numsp.salinity	3078.730	1034.5245	0	0	1
deep.numsp.numrec	3138.989	1094.7832	0	0	1
deep.numsp.chlorophyll	3141.111	1096.9052	0	0	1
deep.numsp.intercept	7047.531	5003.3255	0	0	1

```
write.csv(deep.numsp.aic.df, file = "deep.5.degree.numsp.aic.csv")
```

Here we see that the models containing temperature and current have the best fit, with everything else having a delta AIC over 2. Notably the “numrec” model has a delta AIC over that threshold, indicating that temperature and current have some level of explanatory power for number of species.

Plots for number of species, deep water

Here we plot the effects of each environmental predictor from its respective single-variable model.

```
qplot(deep$temp, predict(deep.numsp.temp, deep)) + geom_smooth(method = "glm")
```

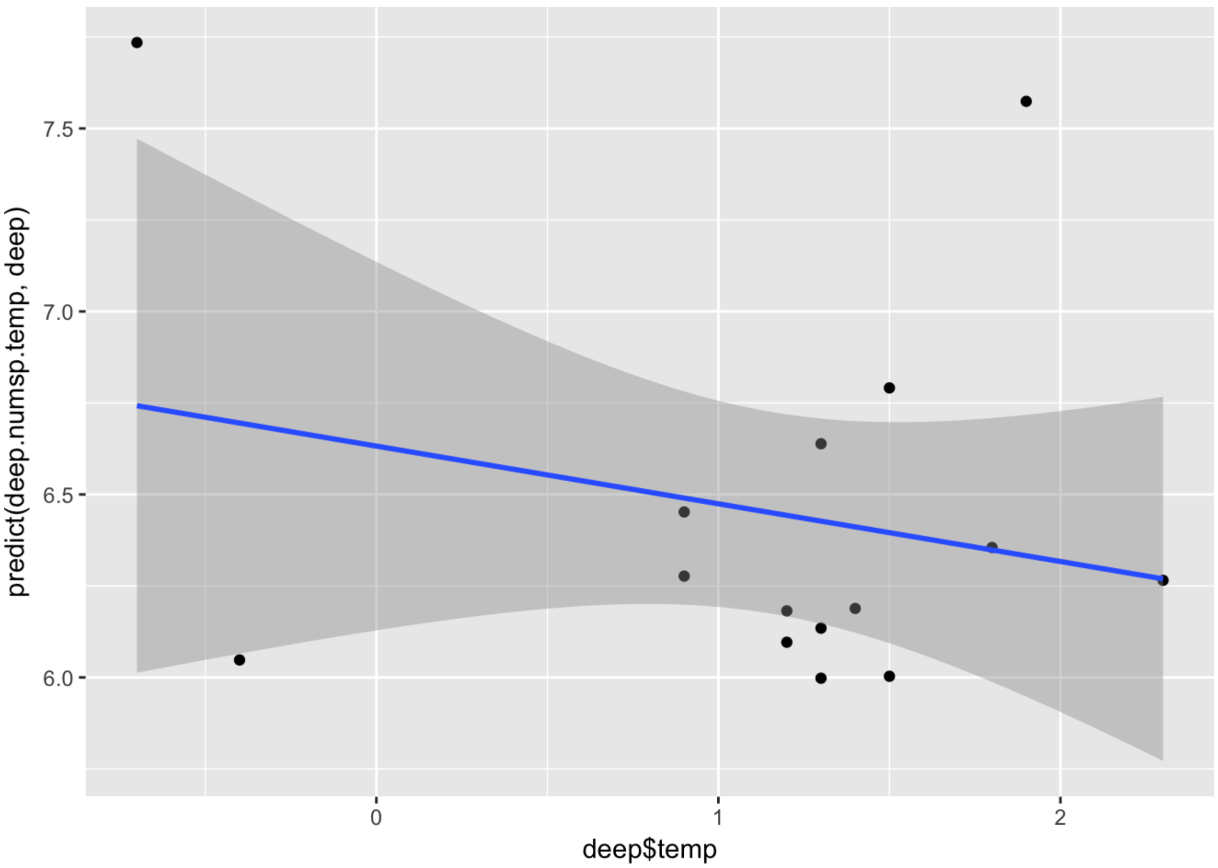


```
qplot(deep$num.species, predict(deep.numsp.numrec, deep)) + geom_smooth(method = "g
lm")
```

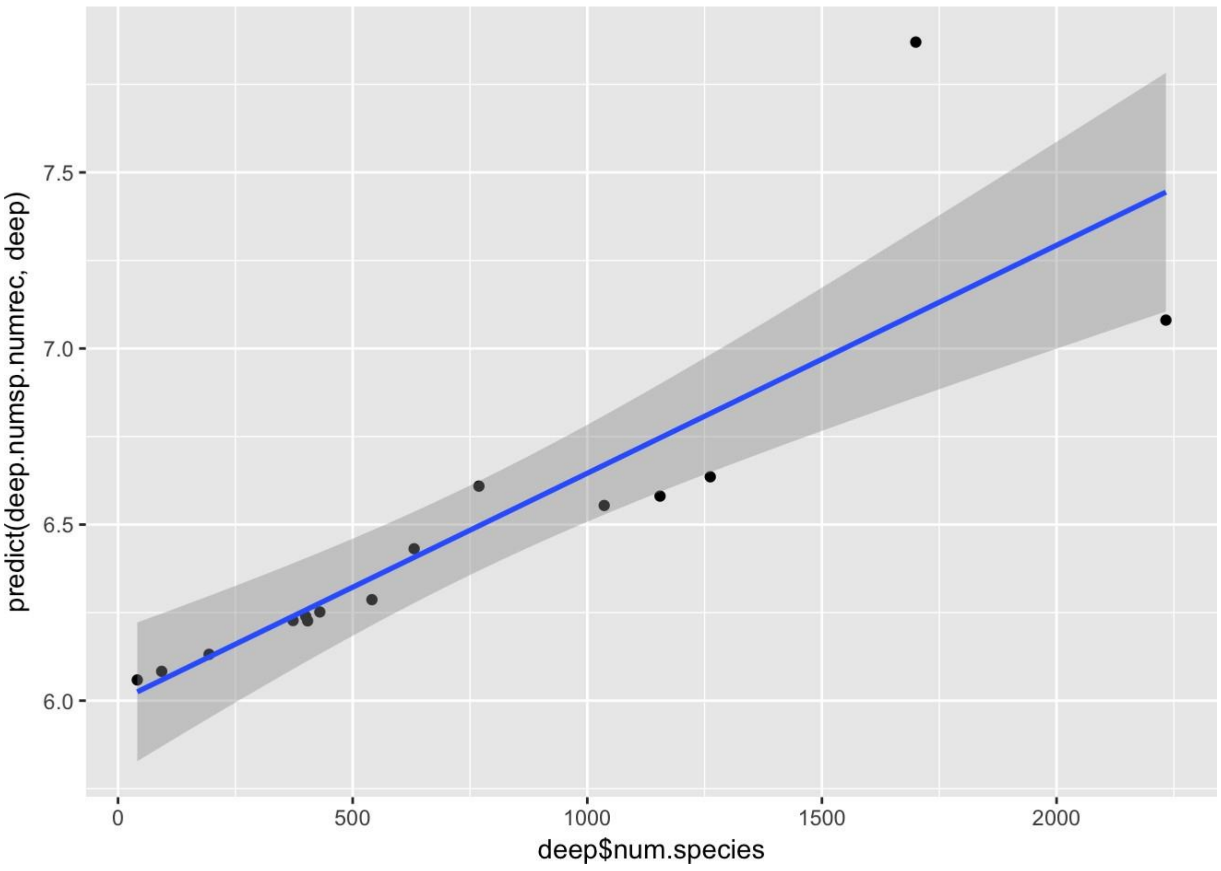


```
qplot(deep$dissolved.oxygen, predict(deep.numsp.oxygen, deep)) + geom_smooth(method
= "glm")
```

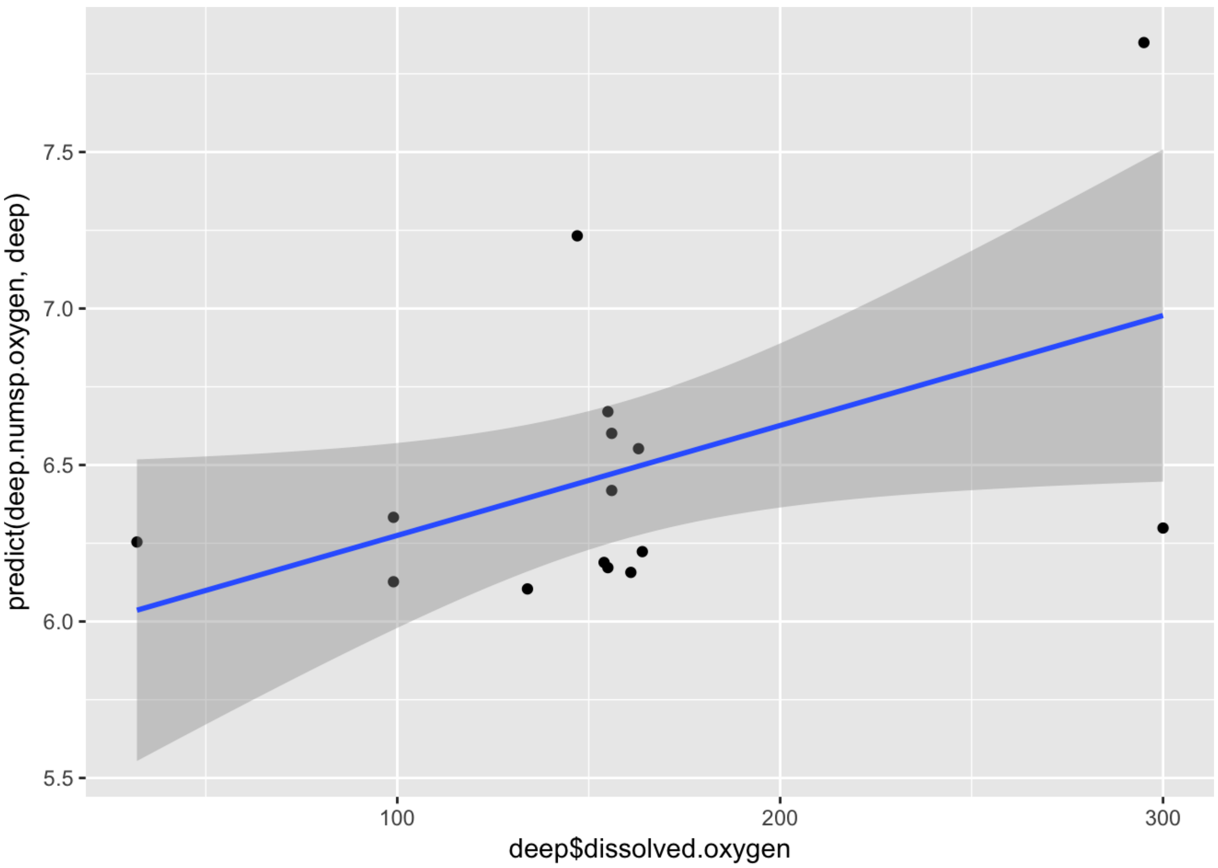


```
qplot(deep$chlorophyll, predict(deep.numsp.chlorophyll, deep)) +  geom_smooth(method
= "glm")
```

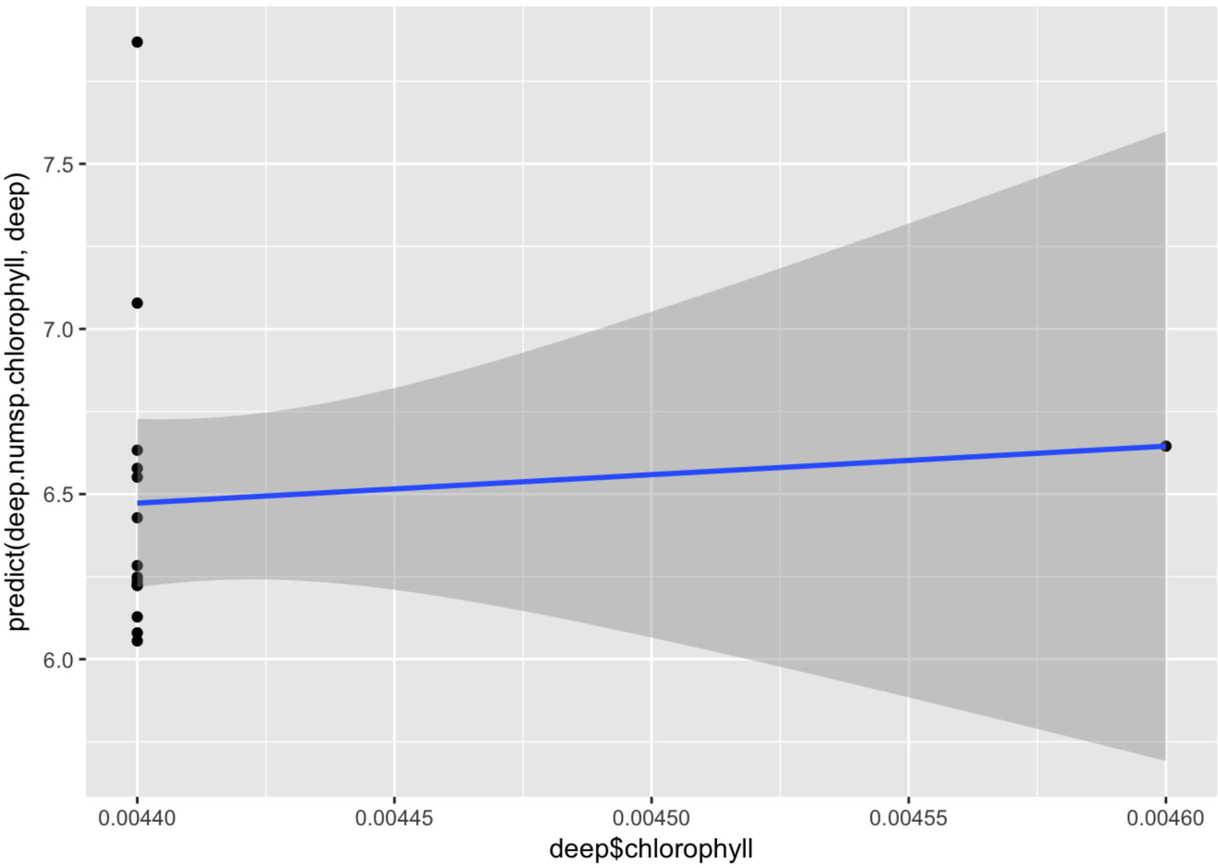


```
qplot(deep$current, predict(deep.numsp.current, deep)) + geom_smooth(method = "glm"
)
```

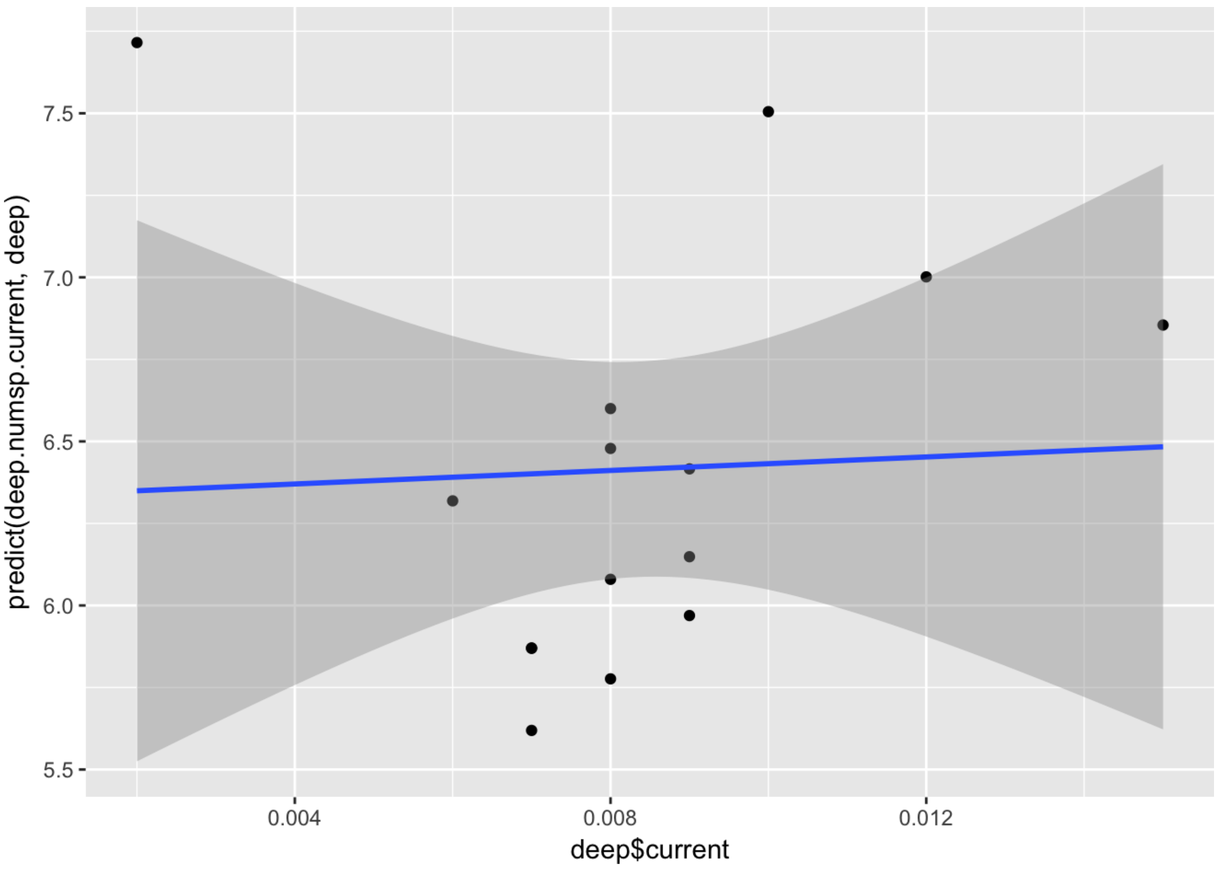


```
qplot(deep$salinity, predict(deep.numsp.salinity, deep)) + geom_smooth(method = "gl
m")
```

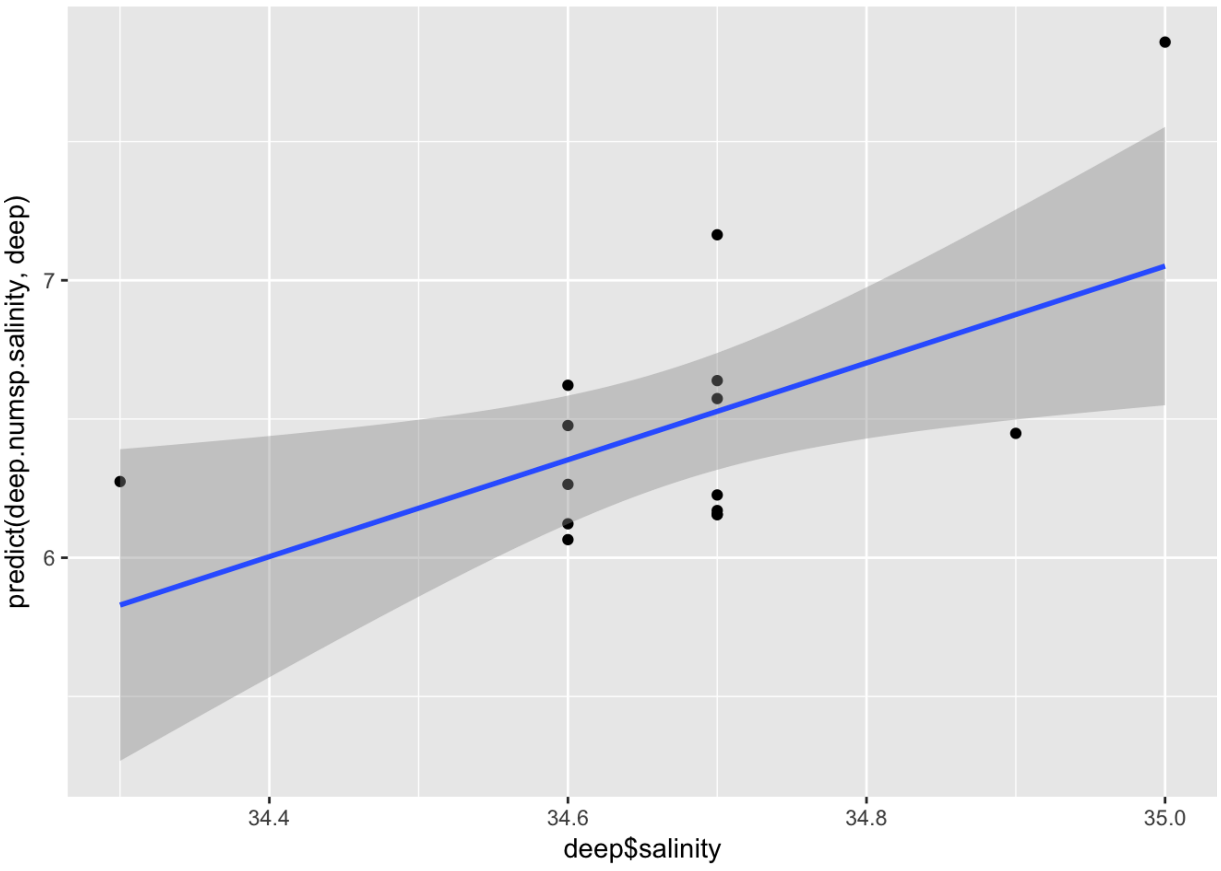


```
qplot(deep$nitrate, predict(deep.numsp.nitrate, deep)) + geom_smooth(method =  "glm"
)
```

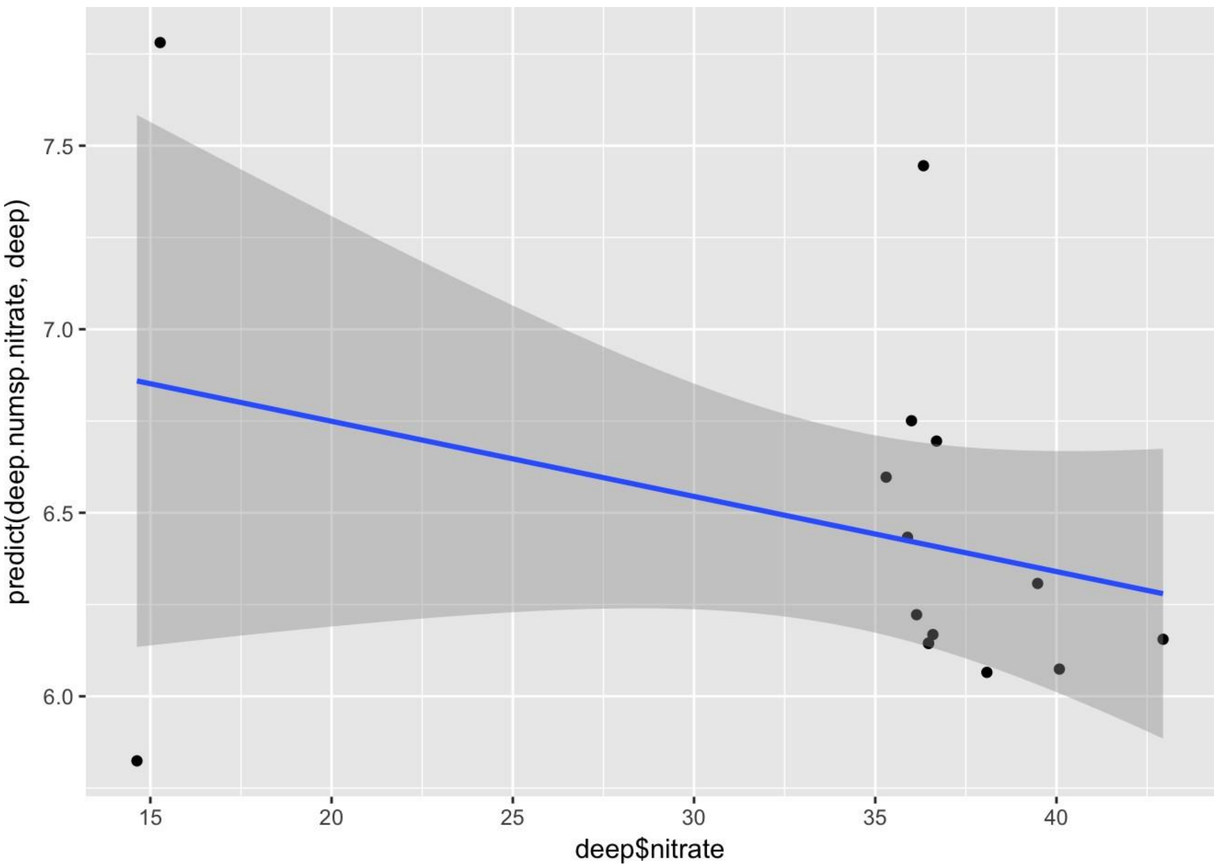

GLMs for ES50, deep water

These models are identical to the above, except that the response variable is the species richness estimated from the rarefaction curves. These are meant to be estimates of species richness if all sites were sampled equally. Since these measurements are intended to take sampling effort into account, we exclude the "num.records" term for these models.

```
deep.es50.intercept <- glm(ES50 ~ 1, family = "poisson", data = deep)
summary(deep.es50.intercept)
```

```
##
## Call:
## glm(formula = ES50 ~ 1, family = "poisson", data = deep)
##
## Deviance Residuals:
##     Min       1Q   Median       3Q      Max
## -1.8427  -1.0814  -0.3752   0.1541   3.9880
##
## Coefficients:
##             Estimate Std. Error z value Pr(>|z|)
## (Intercept)  2.66723    0.06804    39.2   <2e-16 ***
## ---
## Signif. codes:  0 '***' 0.001 '**' 0.01 '*' 0.05 '.' 0.1 ' ' 1
##
## (Dispersion parameter for poisson family taken to be 1)
##
##     Null deviance: 35.111  on 14  degrees of freedom
## Residual deviance: 35.111  on 14  degrees of freedom
## AIC: 103.75
##
## Number of Fisher Scoring iterations: 4
```

```
deep.es50.temp <- glm(ES50 ~ temp, family = "poisson", data = deep)
summary(deep.es50.temp)
```

```
##
## Call:
## glm(formula = ES50 ~ temp, family = "poisson", data = deep)
##
## Deviance Residuals:
##     Min       1Q   Median       3Q      Max
## -1.7290  -0.8220  -0.2853   0.0377   3.1912
##
## Coefficients:
##             Estimate Std. Error z value Pr(>|z|)
## (Intercept)  2.95448    0.10410  28.381  < 2e-16 ***
## temp        -0.26680    0.07986  -3.341 0.000835 ***
## ---
## Signif. codes:  0 '***' 0.001 '**' 0.01 '*' 0.05 '.' 0.1 ' ' 1
##
## (Dispersion parameter for poisson family taken to be 1)
##
##     Null deviance: 35.111  on 14  degrees of freedom
## Residual deviance: 24.730  on 13  degrees of freedom
## AIC: 95.373
##
## Number of Fisher Scoring iterations: 4
```

```
deep.es50.oxygen <- glm(ES50 ~ dissolved.oxygen, family = "poisson", data = deep)
summary(deep.es50.oxygen)
```

```
##
## Call:
## glm(formula = ES50 ~ dissolved.oxygen, family = "poisson", data = deep)
##
## Deviance Residuals:
##      Min        1Q    Median        3Q       Max
## -1.78608  -0.96314  -0.24994   0.05043   2.57468
##
## Coefficients:
##                   Estimate Std. Error z value Pr(>|z|)
## (Intercept)      2.0903096  0.1815253  11.515  < 2e-16 ***
## dissolved.oxygen 0.0034819  0.0009681   3.597 0.000322 ***
## ---
## Signif. codes:  0 '***' 0.001 '**' 0.01 '*' 0.05 '.' 0.1 ' ' 1
##
## (Dispersion parameter for poisson family taken to be 1)
##
##     Null deviance: 35.111  on 14  degrees of freedom
## Residual deviance: 22.866  on 13  degrees of freedom
## AIC: 93.509
##
## Number of Fisher Scoring iterations: 4
```

```
deep.es50.chlorophyll <- glm(ES50 ~ chlorophyll, family = "poisson", data = deep)
summary(deep.es50.chlorophyll)
```

```
##
## Call:
## glm(formula = ES50 ~ chlorophyll, family = "poisson", data = deep)
##
## Deviance Residuals:
##     Min       1Q   Median       3Q      Max
## -1.7628  -0.9985  -0.2894  -0.0095   4.0896
##
## Coefficients:
##             Estimate Std. Error z value Pr(>|z|)
## (Intercept)   -3.962      5.307  -0.747    0.455
## chlorophyll 1501.463   1201.121   1.250    0.211
##
## (Dispersion parameter for poisson family taken to be 1)
##
##     Null deviance: 35.111  on 14  degrees of freedom
## Residual deviance: 33.671  on 13  degrees of freedom
## AIC: 104.31
##
## Number of Fisher Scoring iterations: 4
```

```
deep.es50.current <- glm(ES50 ~ current, family = "poisson", data = deep)
summary(deep.es50.current)
```

```
##
## Call:
## glm(formula = ES50  ~ current, family = "poisson", data = deep)
##
## Deviance Residuals:
##       Min          1Q     Median         3Q        Max
## -2.1418   -0.8155   -0.1136    0.5595    2.9705
##
## Coefficients:
##                  Estimate Std. Error z value Pr(>|z|)
## (Intercept)      3.3315       0.2045   16.293   <  2e-16  ***
## current        -82.7742      24.9834  -3.313  0.000922 ***
## ---
## Signif. codes:  0 '***' 0.001 '**' 0.01 '*' 0.05 '.'  0.1 ' ' 1
##
## (Dispersion parameter for poisson family taken to be 1)
##
##   Null deviance: 35.111  on 14  degrees of freedom
## Residual deviance: 24.136  on 13  degrees of freedom
## AIC: 94.779
##
## Number of Fisher Scoring iterations: 4
```

```
deep.es50.salinity <- glm(ES50 ~ salinity, family = "poisson", data = deep)
summary(deep.es50.salinity)
```

```
##
## Call:
## glm(formula = ES50 ~ salinity, family = "poisson", data = deep)
##
## Deviance Residuals:
##       Min          1Q     Median         3Q        Max
## -1.8960   -1.0256   -0.1811    0.2596    2.4965
##
## Coefficients:
##                  Estimate Std. Error z value  Pr(>|z|)
## (Intercept) -49.9163      15.9064   -3.138 0.001700 **
## salinity          1.5158       0.4583    3.307 0.000942 ***
## ---
## Signif. codes:  0 '***' 0.001 '**' 0.01 '*' 0.05 '.'  0.1 ' '  1
##
## (Dispersion parameter for poisson family taken to be 1)
##
##     Null deviance: 35.111    on 14    degrees of freedom
## Residual deviance: 24.250   on 13   degrees of freedom
## AIC: 94.893
##
## Number of Fisher Scoring iterations: 4
```

```
deep.es50.nitrate <- glm(ES50 ~ nitrate, family = "poisson", data = deep)
summary(deep.es50.nitrate)
```

```
##
## Call:
## glm(formula = ES50 ~ nitrate, family = "poisson", data = deep)
##
## Deviance Residuals:
##      Min        1Q    Median        3Q       Max
## -1.51928  -0.95869  -0.07878   0.16286   2.67794
##
## Coefficients:
##              Estimate Std. Error z value Pr(>|z|)
## (Intercept)  3.675190   0.237422  15.480  < 2e-16 ***
## nitrate     -0.030235   0.007086  -4.267 1.98e-05 ***
## ---
## Signif. codes:  0 '***' 0.001 '**' 0.01 '*' 0.05 '.' 0.1 ' ' 1
##
## (Dispersion parameter for poisson family taken to be 1)
##
##     Null deviance: 35.111  on 14  degrees of freedom
## Residual deviance: 18.927  on 13  degrees of freedom
## AIC: 89.57
##
## Number of Fisher Scoring iterations: 4
```

Model selection for ES50, deep water

Here we use AIC to compare models for goodness of fit while penalizing for overparameterization. Models are ranked in order of AIC score, with lowest scoring model (model with the best compromise between fit and complexity) first in the list. The delta AIC represents how much the AIC score for each model differs from the top model, an can be used as an estimate of the relative support for each model. A delta AIC of 2 is considered to be potentially a significantly better fit, with higher delta AICs between models indicating increasingly larger differences in model fit while correcting for the number of parameters.

```
deep.es50.models <- list(deep.es50.intercept = deep.es50.intercept,
                         deep.es50.temp = deep.es50.temp,
                         deep.es50.oxygen = deep.es50.oxygen,
                         deep.es50.chlorophyll = deep.es50.chlorophyll,
                         deep.es50.current = deep.es50.current,
                         deep.es50.salinity = deep.es50.salinity,
                         deep.es50.nitrate = deep.es50.nitrate)
deep.es50.aic.df <- data.frame(Model = names(deep.es50.models),
                               AIC = sapply(deep.es50.models, function(x) AICc(x)),
                              akaike.weights(sapply(deep.es50.models, function(x) AIC
c(x))))

deep.es50.aic.df <- deep.es50.aic.df[order(deep.es50.aic.df$AIC),]
deep.es50.aic.df$Cumulative.Weight <- cumsum(deep.es50.aic.df$weights)

kable(deep.es50.aic.df, row.names = FALSE)
```

Model	AIC	deltaAIC	rel.LL	weights	Cumulative.Weight
deep.es50.nitrate	90.57036	0.000000	1.0000000	0.7462194	0.7462194
deep.es50.oxygen	94.50935	3.938991	0.1395273	0.1041179	0.8503373

Model	AIC	deltaAIC	rel.LL	weights	Cumulative.Weight
deep.es50.current	95.77905	5.208689	0.0739516	0.0551841	0.9055214
deep.es50.salinity	95.89287	5.322507	0.0698606	0.0521313	0.9576527
deep.es50.temp	96.37322	5.802862	0.0549445	0.0410007	0.9986534
deep.es50.intercept	104.06178	13.491417	0.0011759	0.0008775	0.9995309
deep.es50.chlorophyll	105.31431	14.743947	0.0006286	0.0004691	1.0000000

```
write.csv(deep.es50.aic.df, file = "deep.5.degree.es50.aic.csv")
```

We find that the model containing nitrate is the best fit, with other models having a delta AIC over 2. This indicates that nitrate is the best predictor for ES50 in the deep ocean.

Plots for ES50, deep water

Here we plot the effects of each environmental predictor from its respective single-variable model.

```
qplot(deep$temp, deep$ES50) + geom_smooth(method = "glm")
```

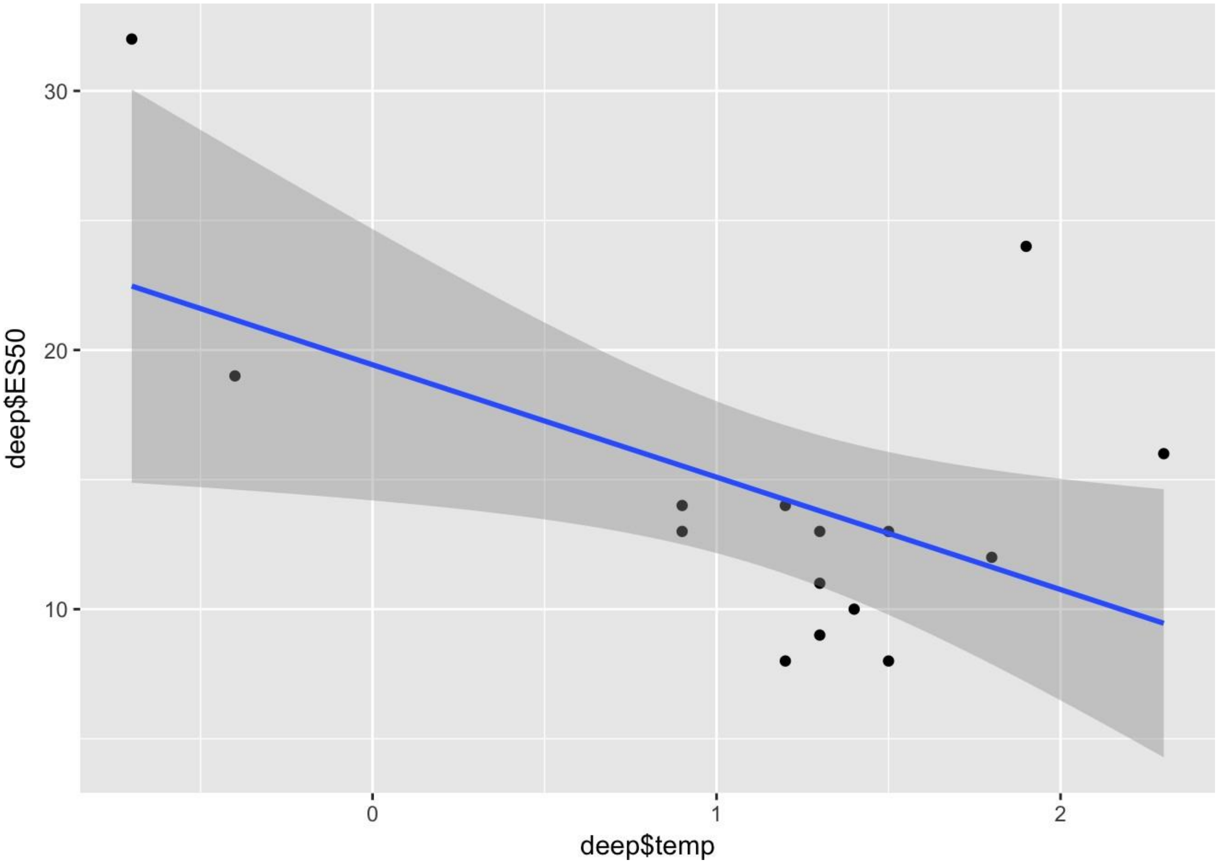


```
qplot(deep$num.records, deep$ES50) + geom_smooth(method = "glm")
```

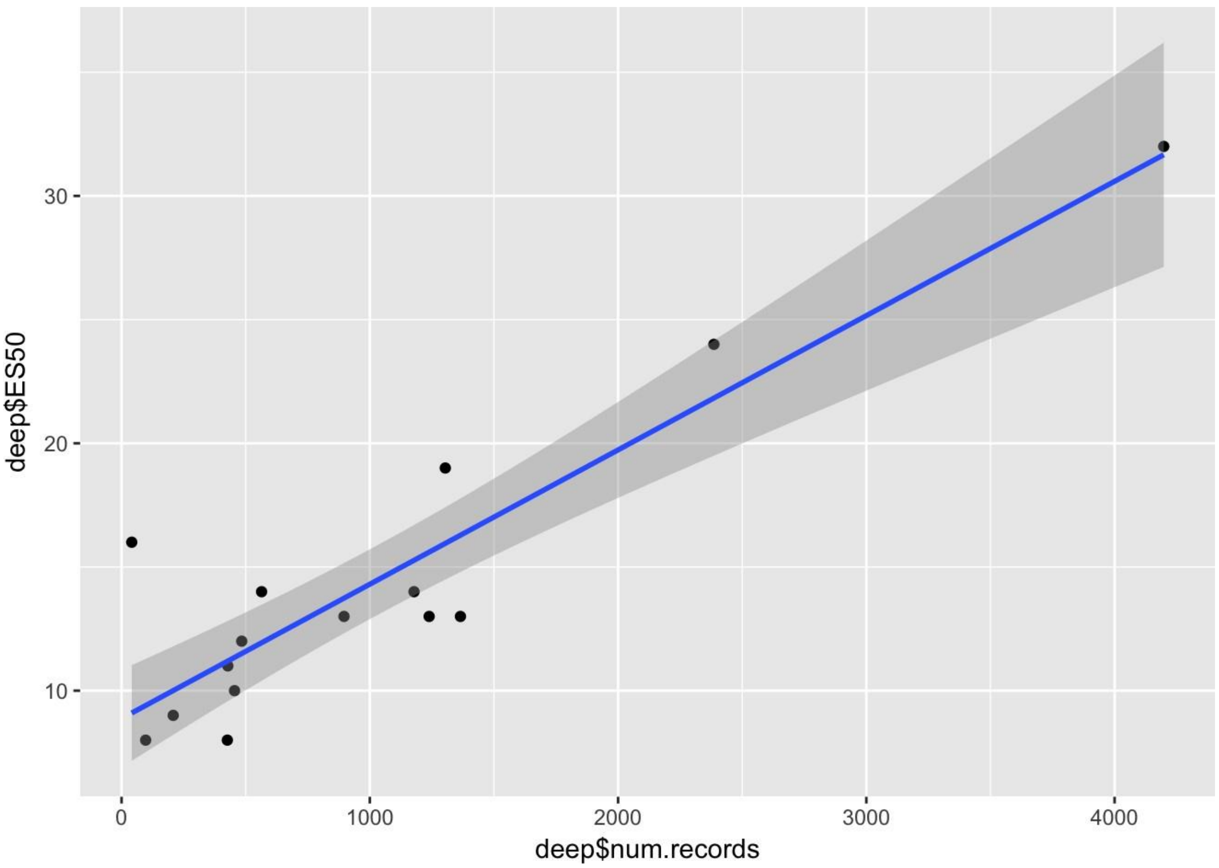


```
qplot(deep$dissolved.oxygen, deep$ES50) + geom_smooth(method = "glm")
```

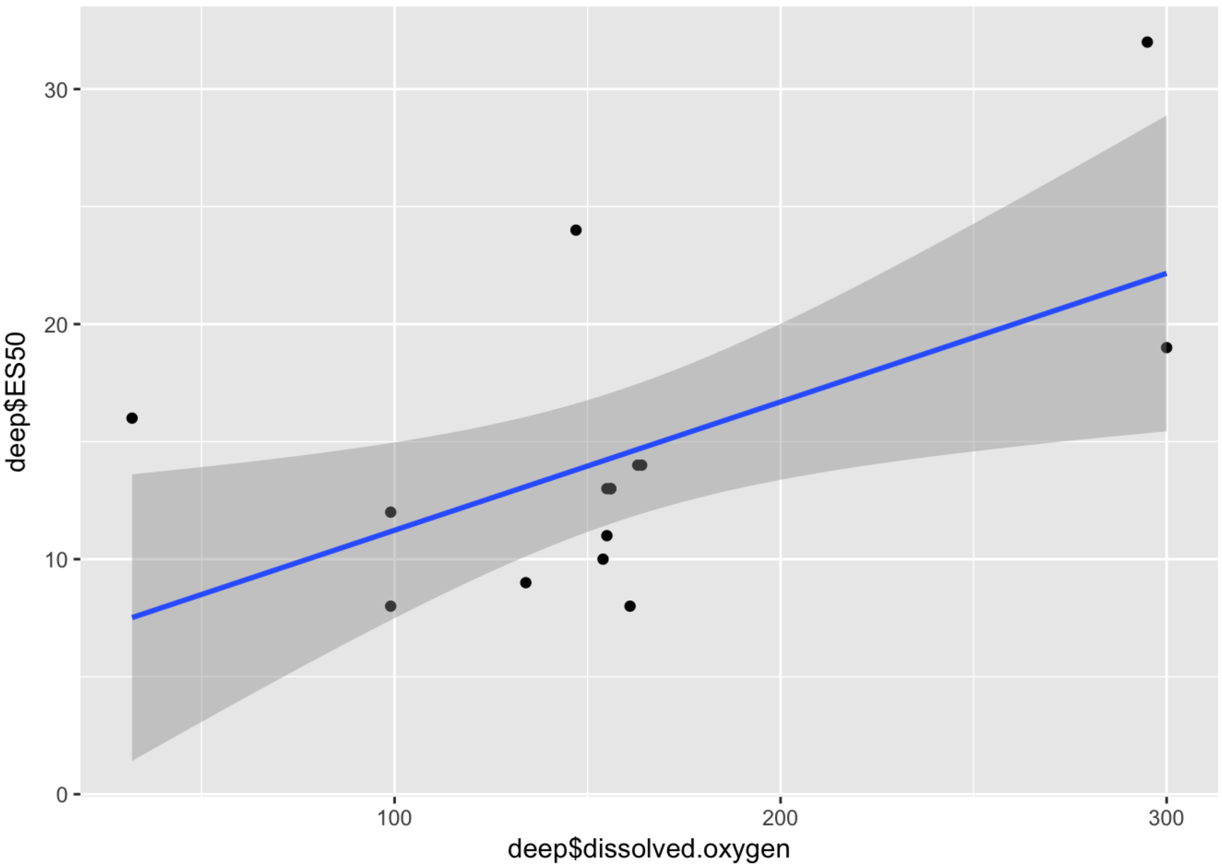


```
qplot(deep$chlorophyll, deep$ES50) + geom_smooth(method = "glm")
```

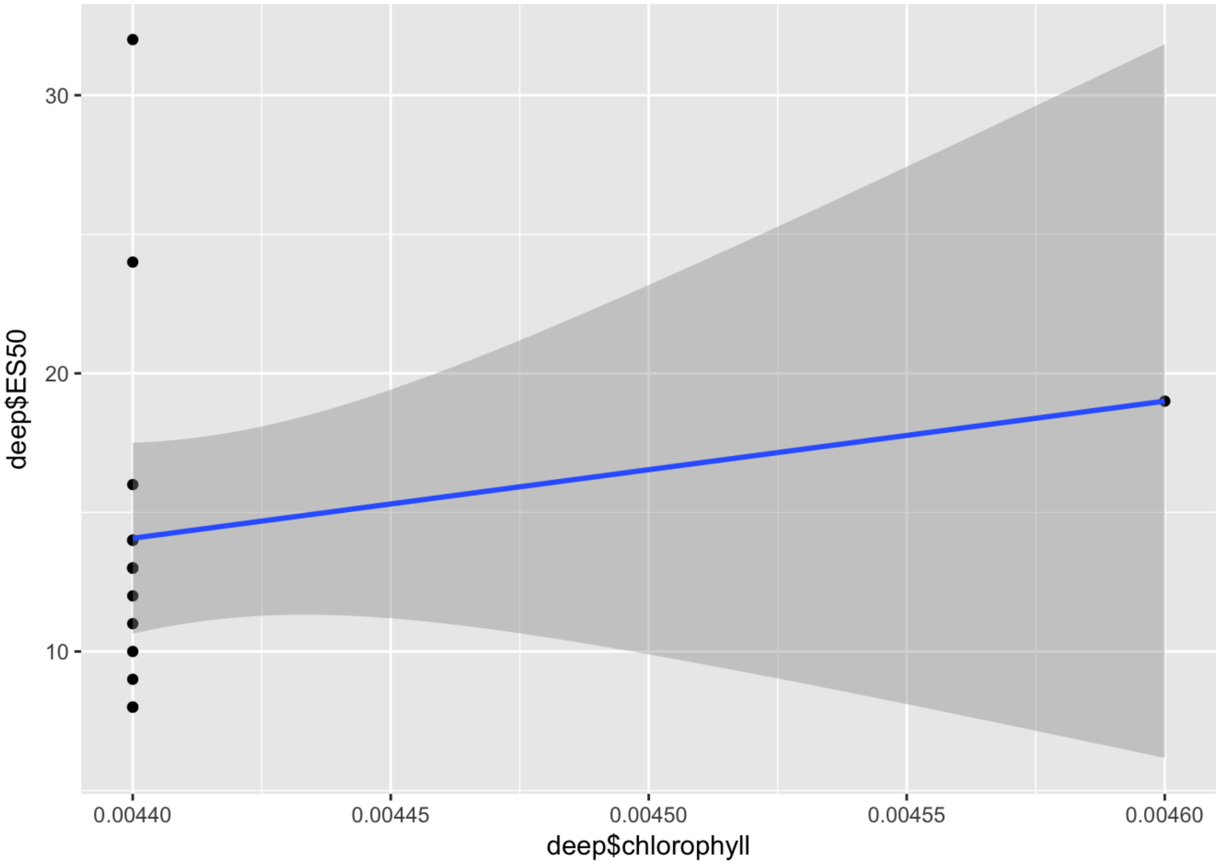


```
qplot(deep$current, deep$ES50) + geom_smooth(method = "glm")
```

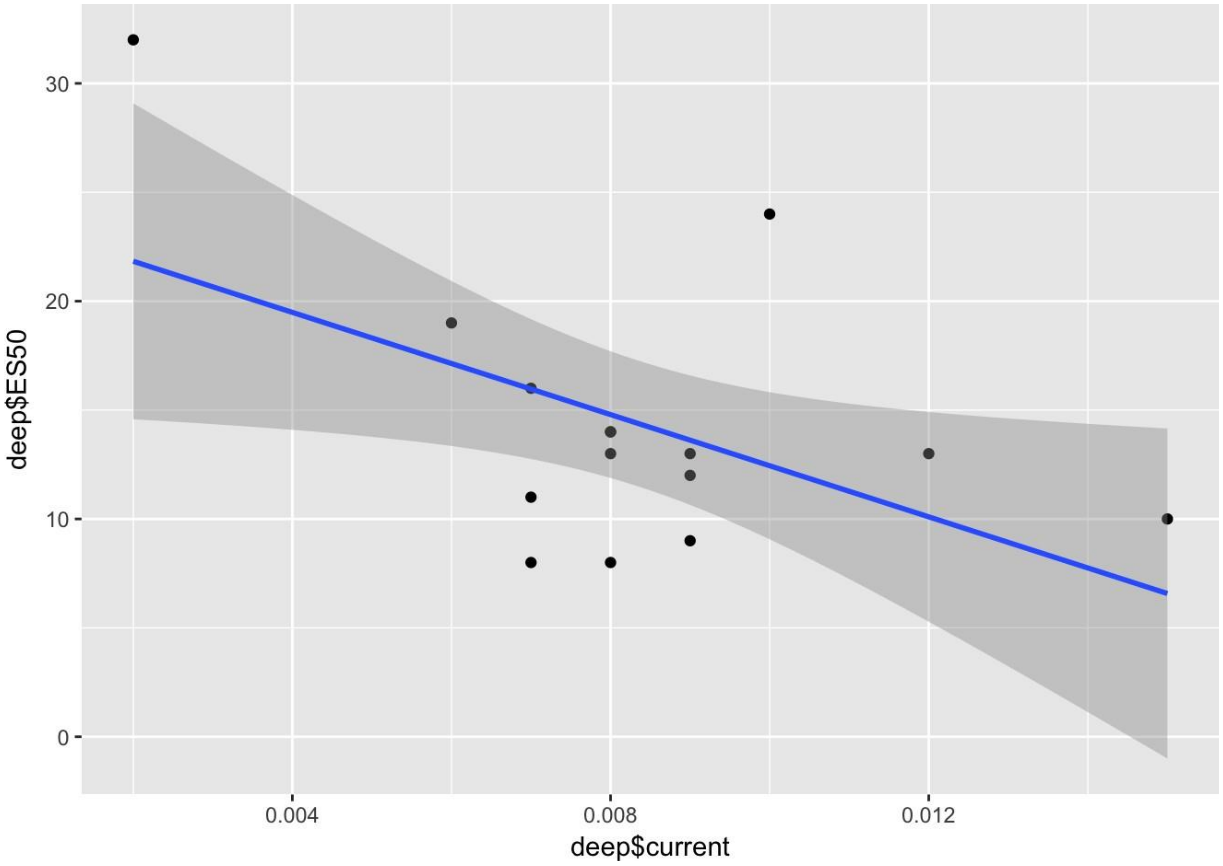


```
qplot(deep$salinity, deep$ES50) + geom_smooth(method = "glm")
```

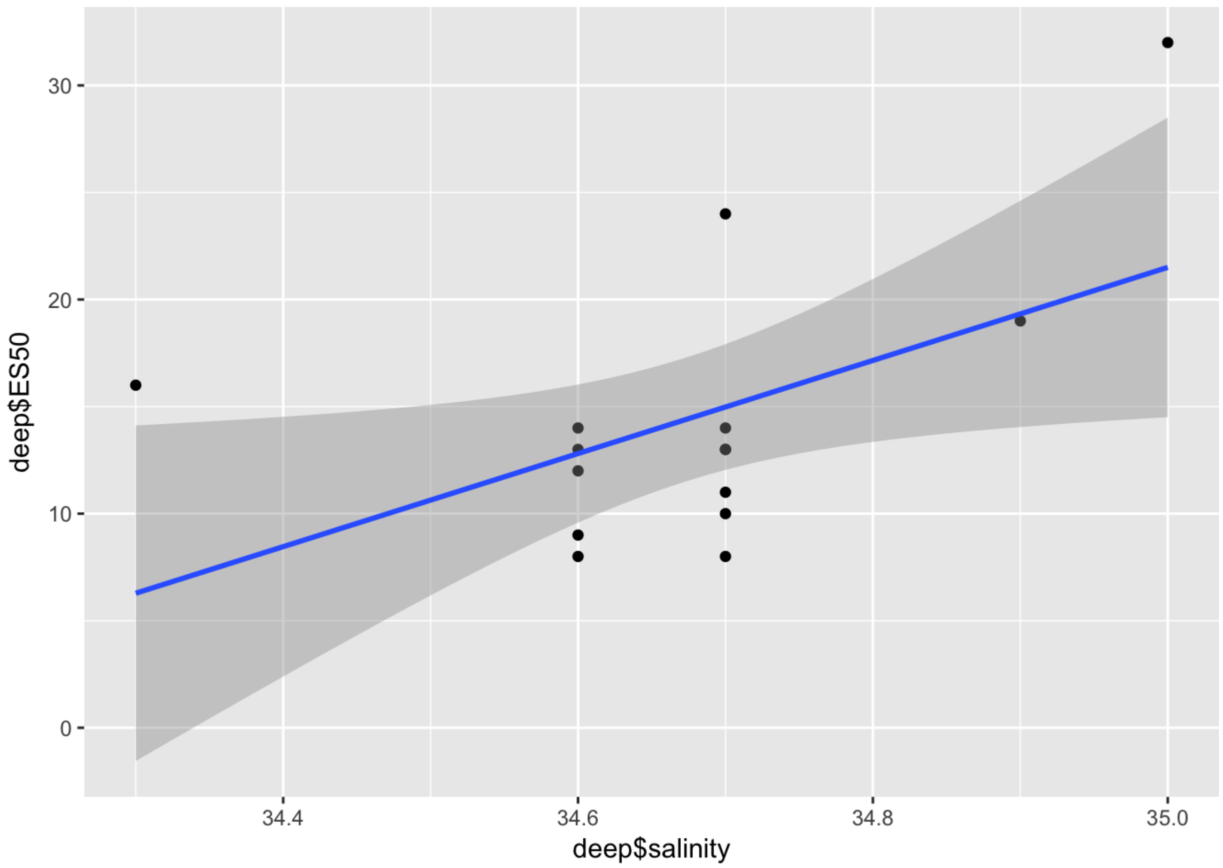


```
qplot(deep$nitrate, deep$ES50) + geom_smooth(method = "glm")
```

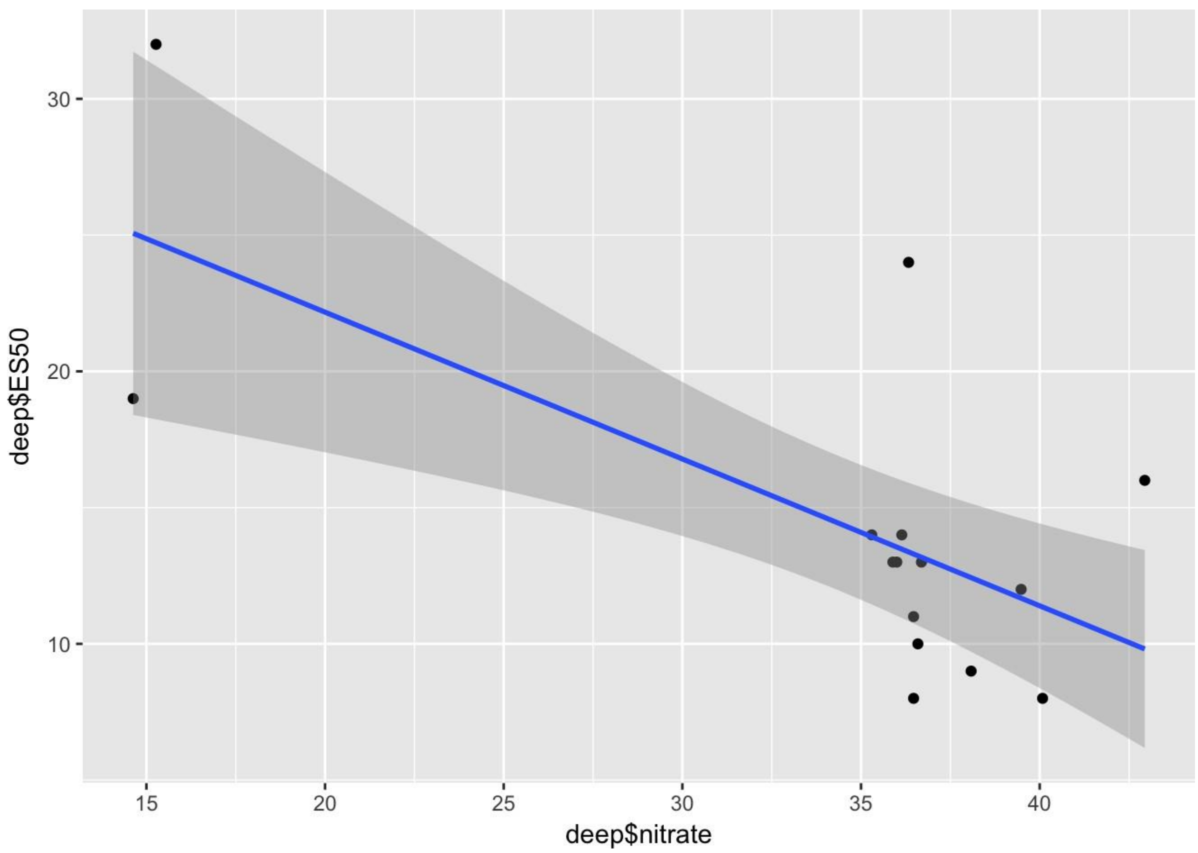

Supplement: Supplementary file 1 — SI [file 41598_2019_45813_MOESM1_ESM.pdf]
